# Iodine-Catalyzed Prins Cyclization of Homoallylic Alcohols and Aldehydes

**DOI:** 10.3390/molecules180911100

**Published:** 2013-09-10

**Authors:** Kachi R. Kishore Kumar Reddy, Iara M. L. Rosa, Antônio C. Doriguetto, Erick L. Bastos, Luiz F. Silva

**Affiliations:** 1 Instituto de Química - Universidade de São Paulo, Av. Prof. Lineu Prestes, 748, CP 26077, CEP 05513-970 São Paulo SP, Brazil; E-Mails: kishorereddyk@gmail.com (K.R.K.K.R.); elbastos@iq.usp.br (E.L.B.); 2 Laboratório de Cristalografia, Instituto de Química, Universidade Federal de Alfenas, Rua Gabriel Monteiro da Silva 714, 37130-000 Alfenas, Minas Gerais, Brazil; E-Mails: cristalografia.landre@gmail.com (I.M.L.R.); doriguetto@unifal-mg.edu.br (A.C.D.)

**Keywords:** isochromene, pyrans, prins cyclization, iodine, DFT calculations

## Abstract

The iodine-catalyzed Prins cyclization of homoallylic alcohols and aldehydes was investigated under metal-free conditions and without additives. Anhydrous conditions and inert atmosphere are not required. The reaction of 2-(3,4-dihydronaphthalen-1-yl)propan-1-ol and 21 aldehydes (aliphatic and aromatic) in CH_2_Cl_2_ in the presence of 5 mol % of iodine gave 1,4,5,6-tetrahydro-2*H*-benzo[*f*]isochromenes in 54%–86% yield. Under similar conditions, the Prins cyclization of six alcohols containing an endocyclic double bond (primary, secondary, or tertiary) led to dihydropyrans in 52%–91% yield. The acyclic homoallylic alcohols gave 4-iodo-tetrahydropyran in 29%–41% yield in the presence of 50 mol % of iodine. This type of substrate is the main limitation of the methodology. The relative configuration of the products was assigned by NMR and X-ray analysis. The mechanism and the ratio of the products are discussed, based on DFT calculations.

## 1. Introduction

**Scheme 1:**
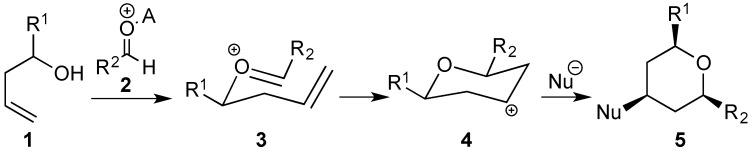
General mechanism for Prins cyclization.

The Prins cyclization is a powerful method for the synthesis of hydropyrans [1,2,3,4,5,6,7,8,9,10,11,12,13,14,15,16,17,18,19]. Several natural products were obtained using this reaction as an important step [7,8,20,21,22,23,24,25]. Typically, this transformation is carried out treating a mixture of a homoallylic alcohol and a carbonyl compound in the presence of an acid (Bronsted or a Lewis) (Scheme 1). One of the possible Lewis acids for Prins cyclization is iodine [26,27,28], which was used in stoichiometric amount in the presence of excess of homoallylic alcohols [29,30], Herein, we describe that a series of new pyrans can be obtained through Prins cyclization using 5 mol % of iodine and equimolar amounts of homoallylic alcohols and aldehydes in an efficient manner [31]. Anhydrous conditions and inert atmosphere are not required in this metal-free protocol.

## 2. Results and Discussion

### 2.1. Discovering the Iodine-Catalyzed Prins Cyclization

**Scheme 2:**
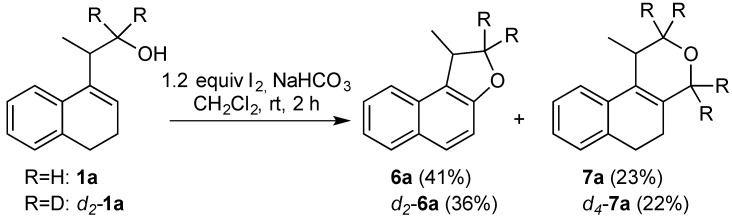
Reaction of homoallylic alcohol **1a** with Iodine.

**Scheme 3:**
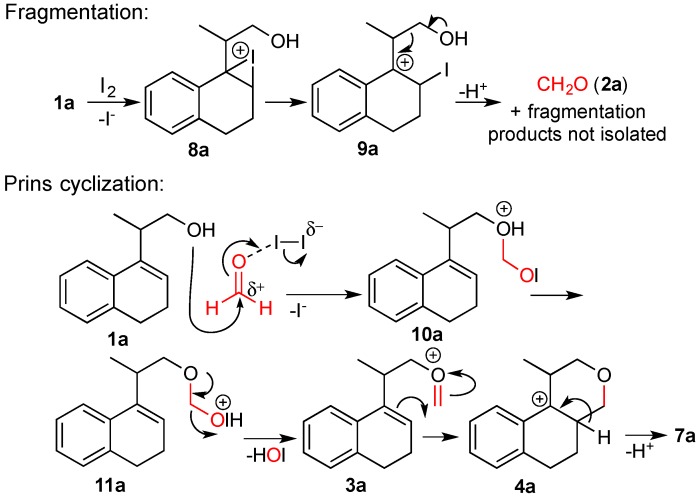
Fragmentation and Prins cyclization of homoallylic alcohol **1a**.

Aiming to synthesize *O*-heterocyclic compounds, we decided to investigate the reaction of the homoallylic alcohol **1a** with iodine in the presence of NaHCO_3_. Under these conditions, naphthalene **6a** and the benzo[*f*]isochromene **7a** were isolated (Scheme 2). The cyclic ether **6a** is formed from an overall 5-*endo*-*trig* iodocyclization [32,33,34,35,36], followed by aromatization. The compound **7a** is formed by an iodine-induced fragmentation of **1a** [35,37,38,39], which generates formaldehyde. The Prins cyclization of **1a** and formaldehyde gives the isochromene **7a** (Scheme 3) [29,30]. To give further evidence for these mechanisms, *d_2_*-**1a** was prepared and submitted to the same reaction conditions, giving *d_2_*-**6a** and *d_4_*-**7a** in 36 and 22% yield, respectively (Scheme 2).

### 2.2. Scope of the Iodine-Catalyzed Prins Cyclization: Aromatic Aldehydes

The Prins cyclization of **1a** and *p*-anisaldehyde (**2b**) was investigated in detail to optimize the preparation of **7a** (Table 1) [31]. We found that the desired product **7a** can be obtained in 75% yield using 5 mol % of I_2_ (entry 4). This condition was used in the Prins cyclization of **1a** and other aldehydes. The reaction of HI and HOI, which are formed in the reaction medium, gives I_2_ and H_2_O, thus explaining the catalytic use of I_2_ [40]. The regeneration of I_2_ is possible because iodide does not act as nucleophile, *i.e.*, is not incorporated in the product, differing from the work of Yadav and co-workers [29,30]. However, we cannot exclude the participation of HI and of HOI to promote the Prins cyclization.

**Table 1:** Prins cyclization of **1a** and *p*-anisaldehyde (**2b**) ^a^. 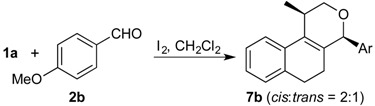

Entry	2b (equiv)	I_2_ (equiv)	Yield of 7b
1	2.3	1.1	54% ^b^
2	2.1	0.5	71% ^c^
3	1.0	0.2	81%
4	1.0	0.05	75%
5	1.0	0	-- ^d^

^a^ Ratio estimated by NMR. Relative configuration assigned by NOESY. ^b^ aldehyde recovered: 31%. ^c^ aldehyde recovered: 67%. ^d^ No reaction.

Once optimized conditions were found, the reactions of **1a** with a broad selection of aromatic aldehydes were performed. Prins cyclization products were obtained in 60%–86% yield (Table 2). Besides the expected isochromene **7**, the isomeric alkenes **12** were also formed, typically as a minor component. The distribution between the pyrans **7** and **12** is discussed in the mechanism section below. The reaction tolerates the presence of electron donating (Me, OMe and NHAc) and electron withdrawing groups (Br and NO_2_). It can also be performed with aldehydes bearing substituents in the *ortho* position (entries 4, 10 and 13), including the sterically demanding aldehyde **2g**, although in this case the major product is the pyran **12g** (entry 5).

**Table 2:** Iodine-catalyzed Prins cyclization of **1a** and aromatic aldehydes ^a,b^. 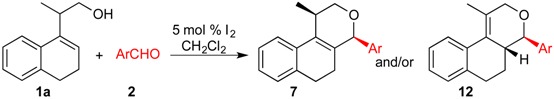

Entry	Aldehyde	Product (Yield)
1	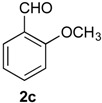	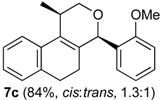
2	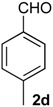	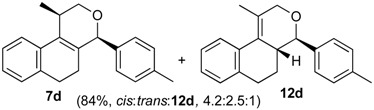
3	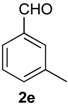	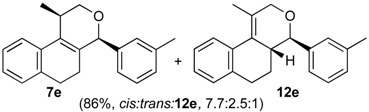
4	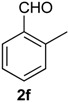	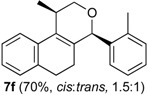
5	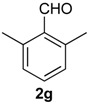	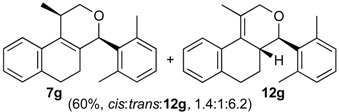
6	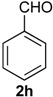	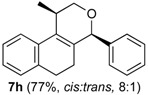
7	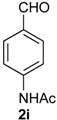	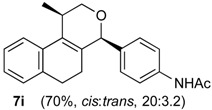
8	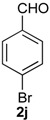	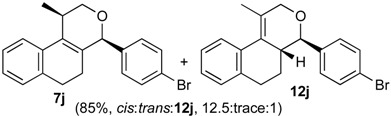
9	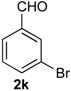	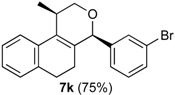
10	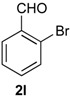	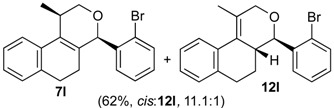
11	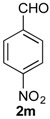	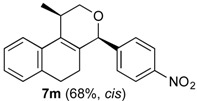
12	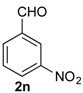	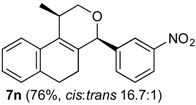
13	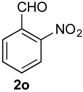	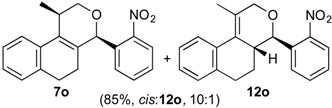

^a^ **1a** (1.0 equiv), aldehyde (1.0 equiv), I_2_ (5 mol %), CH_2_Cl_2_. ^b^ ratio estimated by NMR.

In all cases, the two groups in the isochromenes **7** possess a *cis* relationship with respect to the pyran ring. The relative configurations were assigned by NMR analysis, including NOESY experiments. X-ray analysis of the bromo derivative **7k** gave further evidence for the relative configuration of isochromenes **7**.

**Figure 1:**
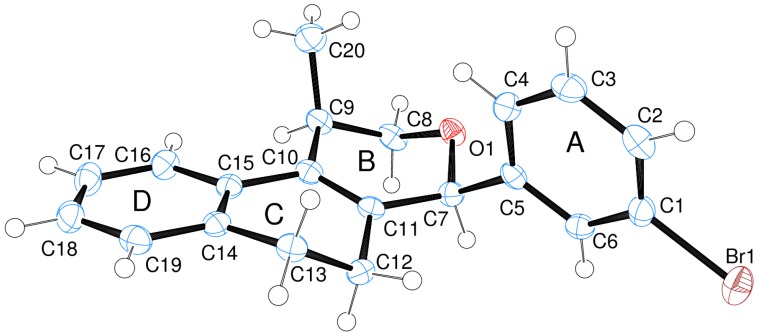
ORTEP-3 view of **7k** showing the atom labeling, configuration of the chiral atoms and 50% probability ellipsoids. H atoms are shown as spheres of arbitrary radii.

Figure 1 is an ORTEP-3 view of **7k**, which was solved in the space group P2_1_2_1_2_1_. Since **7k** is a chiral molecule crystallized in a chiral space group containing just one molecule in the asymmetric unit its crystal structure contains a pure enantiomer [41]. Moreover, due to the presence of the bromine atom, which has an anomalous scattering large enough to permit the refinement of the Flack parameter [42], the absolute structure of **7k** was unambiguously determined in this study. Thus, the chiral atoms present the following configurations: C7(*S*), C9(*R*).

### 2.3. Scope of the Iodine-Catalyzed Prins Cyclization: Aliphatic Aldehydes

The iodine-catalyzed Prins cyclization of homoallylic alcohol **1a** and several aliphatic aldehydes was next investigated (Table 3). Depolymerization of paraformaldehyde occurs *in situ* and the Prins cyclization of the formaldehyde formed *in situ* with **1a** gave **7a** (entry 1).

**Table 3:** Iodine-catalyzed Prins cyclization of **1a** and aliphatic aldehydes ^a^.

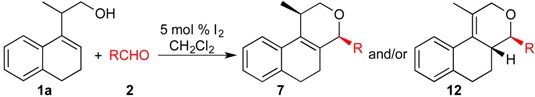		
Entry	Aldehyde	Product (Yield)
1	(CH_2_O)_n_ **2a**	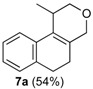
2	MeCHO **2p**	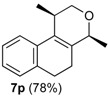
3	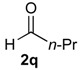	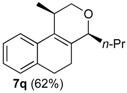
4	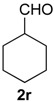	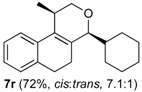
5	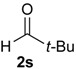	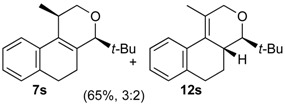
6	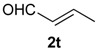	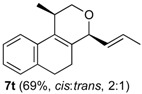
7	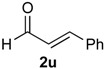	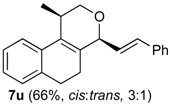

^a^ **1a** (1.0 equiv), aldehyde (1.0 equiv), I_2_ (5 mol %), CH_2_Cl_2_.

The desired products were also obtained using several aliphatic aldehydes, including sterically demanding ones, such as **2q**–**s** (entries 3–5), albeit for **2s** a significant amount of **12s** was formed. Treatment of **1a** with the α,β-unsaturated aldehydes **2t** and **2u** gave the desired isochromenes **7t** and **7u**, respectively, in good yield (entries 6 and 7).

### 2.4. Scope of the Iodine-Catalyzed Prins Cyclization: Homoallylic Alcohols

After studying the reaction of several aliphatic and aromatic aldehydes, the behavior of different homoallylic alcohols was investigated using *p*-anisaldehyde (**2b**), as the carbonyl component. The reaction of endocyclic homoallylic alcohols was first studied (Table 4).

**Table 4:** Prins cyclization of homoallylic alcohols bearing endocyclic double bonds with **2b**.

Entry	Alcohol	Product (Yield)
1	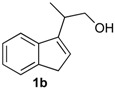	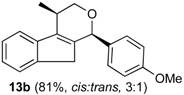
2	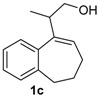	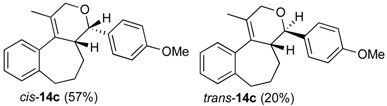
3	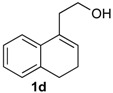	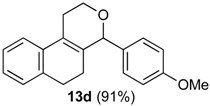
4	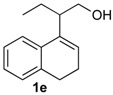	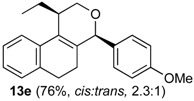
5^b^	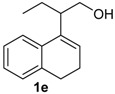	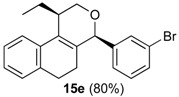
6	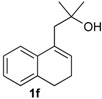	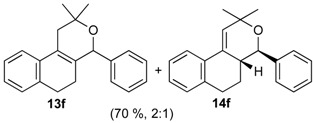
7	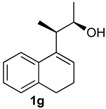	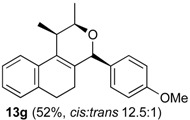

^a^ alcohol **1** (1.0 equiv), aldehyde (1.0 equiv), I_2_ (5 mol %), CH_2_Cl_2_. ^b^ aldehyde **2k**. ^c^ aldehyde **2h**.

**Figure 2:**
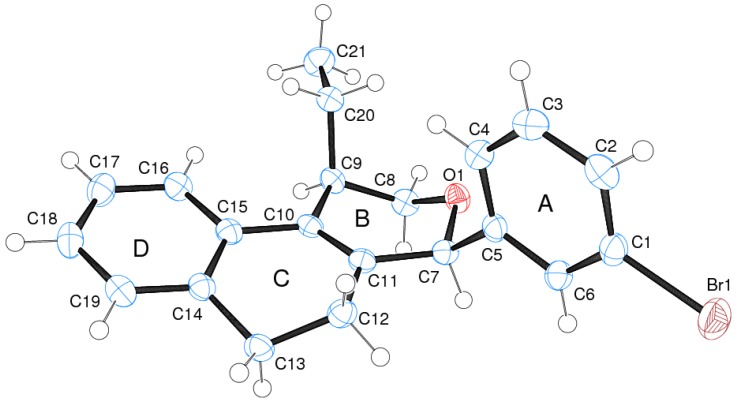
ORTEP-3 view of **15e** (1*R*,4*S*-conformer) showing the atom labeling, configuration of the chiral atoms and 50% probability ellipsoids. H atoms are shown as spheres of arbitrary radii.

The iodine-catalyzed Prins cyclization can also be performed with endocyclic homoallylic alcohols bearing the double bond in five and seven-membered rings (entries 1 and 2). Homoallylic alcohols with different side chains can be used as substrate (entries 3–5), including tertiary (entry 6) and secondary alcohols (entry 7). The bromo derivative **15e** (entry 5) was isolated as nice crystals and its structure was confirmed by X-ray analysis (Figure 2, see Supplementary Information for details), supporting the relative configuration assigned by NMR. Compound **15e** is a chiral molecule crystallized in a centrosymmetric space group, P2_1_/c. Therefore, its crystal structure is an 50:50 equimolar mixture of a pair of enantiomers (1*R*,4*S*/1*S*,4*R*) in a well-defined arrangement. Figure 2 shows the *1R,4S*-enantiomer structure C7(*S*) and C9(*R*) of **15e**.

Analyzing the intramolecular geometry, it is observed that the individual rings in **7k** and **15e** assume similar geometries: (a) rings A and D are, as expected, very planar; (b) ring B is in half-chair conformation, with atoms O1 and C8 in the flap positions; (c) ring C is in twist-boat conformation. Another similarity is observed comparing the ring B substituents: In both structures the methyl (or ethyl in **15e**) and bromophenyl groups are in axial and bisectional positions respectively related to ring B.

**Figure 3:**
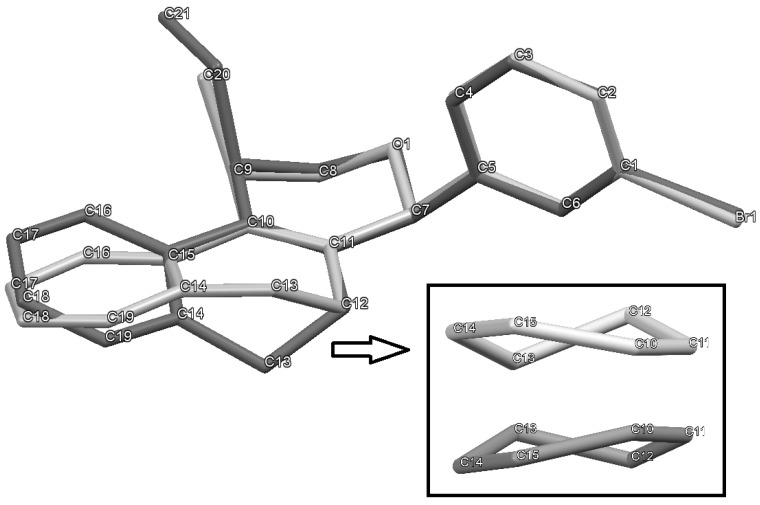
MERCURY view showing the superposition of equivalent conformers of **7k** (light gray) and **15e** (dark gray). The insert shows the rings C in a same view, which rotated 90° considering a vertical axis through center ring. The hydrogen atoms were hidden for the representation clarity.

In Figure 3, the equivalent 1*R*,4*S*-enantiomers of **7k** and **15e** are superimposed in a capped stick fashion. The overlay of molecular backbones clearly shows the conformational similarity between homologous atoms in rings A and B and their first neighbor atoms. Indeed, **7k** and **15e** adopt a very similar conformation in terms of the torsion angles about the bonds by which the bromophenyl ring links the polycyclic three ring system: C6-C5-C7-C11 = 119.0(2) and C4-C5-C7-O1 = 62.5(2)° for **7k**, and 120.2(2) and 63.3(3)°, respectively, for **15e**.

**Figure 4:**
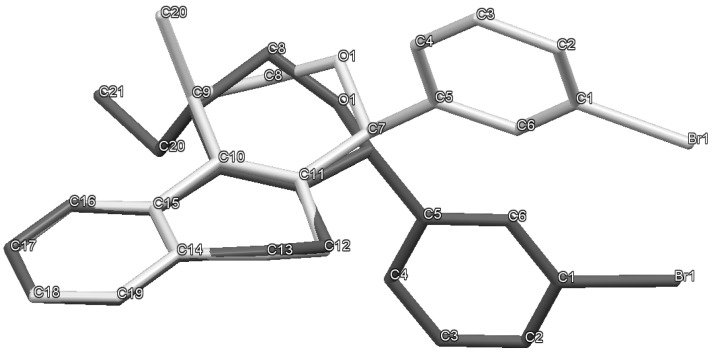
MERCURY view showing the superposition of opposite conformers of **7k** (light gray) and **15e** (dark gray). The hydrogen atoms were hidden for the representation clarity.

On the other hand, the molecular overlay also shows that the homologous atoms in rings C and D do not match. In fact, despite having the same 6-member ring shape, *i.e*., a twist-boat conformation, the ring C orientation in **7k** strongly differs from that one found in 1R,4S-enantiomer of **15e**. As consequence the D rings also do not match themselves. The insert of Figure 3 illustrates the rotated conformation of ring C comparing **7k** and **15e**. Interestingly, the superimposition of the pure 1*R*,4*S*-enantiomer of S15 with the opposite one of S97 (1*S*,4*R*) shows that rings D and C match (Figure 4).

The molecular geometries were studied through MOGUL [43], a knowledge base that takes a molecule submitted either manually or by another computer program via an instruction-file interface and perform substructure searches of the Cambridge Structural Database (CSD) for, typically, all its bond, angles and torsion angles. In both structures, all bond lengths and angles are in agreement with the expected ones, when compared with the similar structures and considering a good refinement.

**Figure 5:**
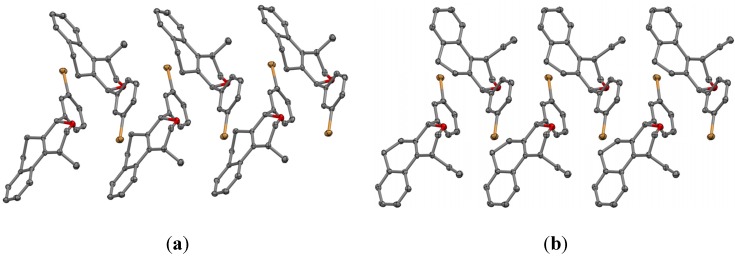
A partial packing diagram for (a) **7k** and (b) **15e**, showing the double chain formed along the respective unit cell *b* axes. The hydrogen atoms were hidden for representation clarity.

**Figure 6:**
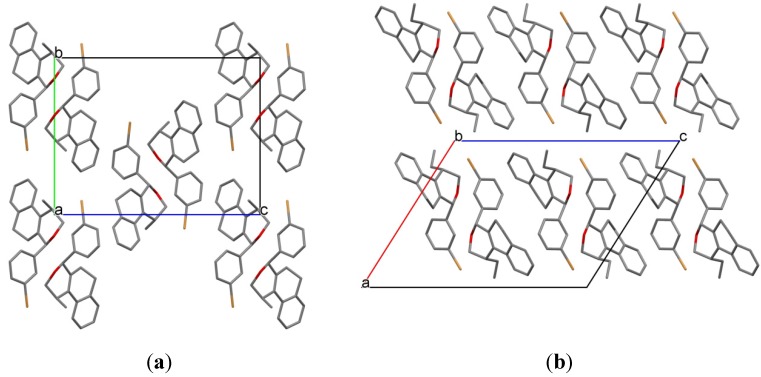
The crystal packing illustration of (a) **7k** and (b) **15e** onto the ac plane. Hydrogen atoms were omitted for clarity.

The crystal packing in **7k** and **15e** is dominated by Van der Waals close contacts. In both structures the molecules are self-assembled generating a double chain along the unit cell *b* axis involving molecules related to 2_1_-fold screw axis (Figure 5). Surprisingly, despite the chemical, molecular conformational, and space group symmetry differences comparing **7k** and **15e**, the 1-D structure along its respective unit cell *b* axis are very similar (Figure 5). This supramolecular synthon is itself linked by Van der Waals forces along unit cell *a* and *c* axis completing the 3-D network of the two structures (Figure 6).

The final stage to understand the scope of the iodine-catalyzed Prins cyclization was the investigation of the reactivity of a series of acyclic homoallylic alcohols with *p*-anisaldehyde (**2b**) (Table 5).

**Table 5:** Iodine-catalyzed Prins cyclization of acyclic homoallylic alcohols with **2b** ^a^.

Entry	Alcohol	Product (Yield)
1	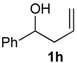	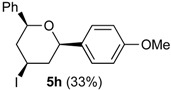
2	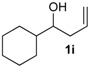	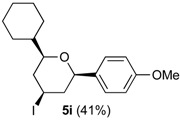
3	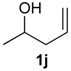	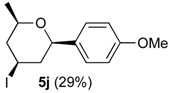
4	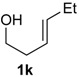	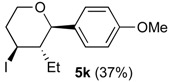
5	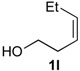	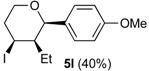
6^b^	**1l**	**5l** (81%)
7^c^	**1l**	**5l** (84%)

^a^ I_2_ (50 mol %), CH_2_Cl_2_. ^b^ 1 equiv of I_2_. ^c^ 1 equiv of I_2_ and 2 equiv of alcohol [30].

In the reaction of alcohols **1h**–**l** the carbocation intermediate **4** (*cf.* Scheme 1) reacts with iodide giving 4-iodo-tetrahydropyrans **5h**–**l**, as product, instead of dihydropyran. Iodine is not regenerated in the medium and the catalytic cycle is interrupted. Thus, as expected, the yields of the reaction are clearly related to the amount of iodine. Using 0.5 equiv of iodine the yields were 33%–40% (entries 1–5), whereas using 1 equiv. [29,30], the yield jumped to 81%–84% (entries 6–7). Using 0.2 equiv of iodine, compound **5h** was obtained in 5% yield. The 4-iodo-hydropyrans **5h**–**l** were isolated as a single diastereomers, corresponding to the equatorial attack of the iodide [5,44].

**Scheme 4:**
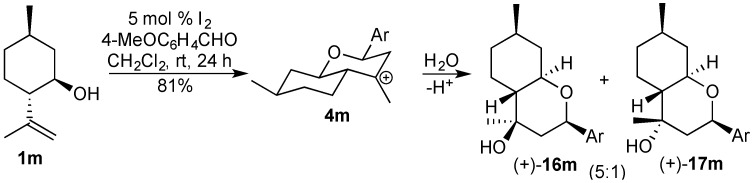
Iodine-catalyzed Prins cyclization of isopulegol (**1m**).

In the Prins cyclization with the monoterpene isopulegol (**1m**), the carbocation intermediate **4m** reacts with water (formed *in situ*) giving the alcohols (+)-**16m** and (+)-**17m**, in a 5:1 mixture, respectively. These products were obtained in very good yield (81%), using 5 mol % of iodine. The attack of the water to the carbocation intermediate **4m** occurs at the equatorial position preferentially, as expected (Scheme 4) [5,44].

### 2.5. Mechanistic Insights Based on DFT Calculations

The mechanism of the Prins cyclization of aldehydes and homoallylic alcohols was investigated by DFT theoretical calculations. The following topics were addressed: (*i*) the preferential formation of the *cis* diastereomer of compound **7** (Table 2 and Table 3); and (*ii*) the preferential formation of **7** instead of **12** (Table 2).

The formation of compound **7h** (Table 2, Entry 6, 8:1 *cis*:*trans*) was selected as the model reaction for the theoretical study. The *cis* diastereoselectivity has been rationalized assuming the preferential formation of the intermediate (*E*)-**3** as well as repulsive steric interactions that compromise the rotation around the dihedral angle formed between the 1,2-dihydronaphthalene ring and the benzylidene(propyl)oxonium moiety (*i.e.*, Me–CH–(C=C), φ_1_) [31,45]. To verity this hypothesis, the structure of the intermediate carbocations (*E*)- and (*Z*)-**3h** in two different twist-boat conformations of the 1,2-dihydronaphtalene ring (namely A and B) were optimized in the gas phase and the rotational barrier around the dihedral angle φ_1_ was determined.

**Scheme 5:**
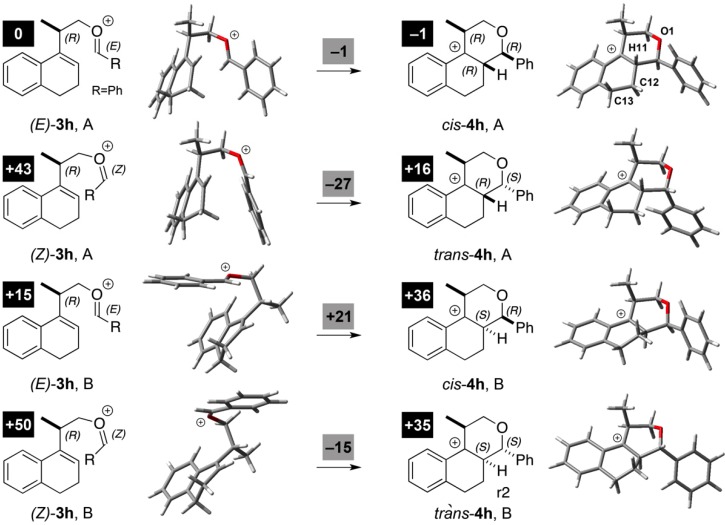
Formation of *cis*- and *trans*-**4h** after the non-stereospecific formation of the oxonium ion **3h**. Optimized minimum geometries and relative ΔG° (in kJ mol^–1^, highlighted) were obtained at the B3LYP/6-31+G(d,p) level.

The barrier for the *E*/*Z* isomerization of **3h** is around 40 kJ mol^–1^ (Scheme 5), corroborating the preferential formation of (*E*)*-***3h**, despite the fact that the non-stereospecific nucleophilic attack of the alcohol **1a** on the carbonyl moiety may give, *a priori*, both *E*- and *Z*- oxocarbenium ions **3h**. Furthermore, the potential barrier for the rotation of the dihedral angle φ_1_ is around 60 kJ mol^–1^ (Figure S1), supporting our initial hypothesis that the repulsive steric interaction between the methyl group and the aromatic hydrogen (H16) compromises the rotation around the dihedral angle φ_1_ [31,45]. However, the *E*/*Z* diastereomers of **3h** still can undergo the electrophilic addition step from both faces of the 1,2-dihydronaphtalene ring in two different half-chair conformations of ring C. Scheme 5 provides an outline of the reaction paths leading to oxonium intermediates of type **4h** in which the C12–C11 is staggered. Electrophilic addition leading to cyclization is spontaneous in all cases, due to the formation of a more stable carbocation **4h**, compared to oxocarbenium ions **3h**. As discussed above, (*E*)-**3h** is more stable than (*Z*)-**3h** in both conformers selected. However, conformer A of (11*R*)-*cis*-**4h** is in average 25 kJ mol^–1^ more stable than other diastereomers. Consequently, the favorable conversion of (*E*)-**3h** into (11*R*)-*cis*-**4h** seems to be the cause for the preferential formation of *cis*-**7h**. However, considering the activation barriers for the formation of (11*R*)-*cis*-**4h** and (11*R*)-*trans*-**4h**, (19 kJ mol^–1^ and 14 kJ mol^–1^, respectively) and the more exergonic formation of the *trans* diastereomer, one cannot rule out the possible preferential formation of the *trans* diastereomer depending on the substitution pattern of **3** (Table S1). Animations showing the reaction coordinate (imaginary frequency) for electrophilic addition step giving both *cis* and *trans* isomers are available in the *SI*.

**Figure 7:**
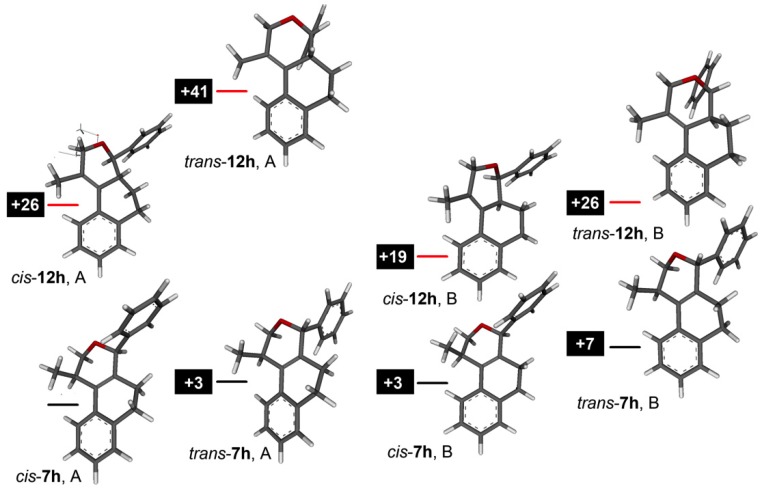
Energy diagram comparing products **7h** and **12h** in two different conformations of ring C.

The elimination reaction involving *cis*- or *trans*-**4** was investigated determining the relative stability of products **7** and **12**. First, the elimination of **4s** was selected as model system since **7s** and **12s** are produced by elimination in roughly equimolar amounts (**7s**/**12s** ratio = 1.5). The Boltzmann ratio **7s**/**12s** is 1.6 (ΔG° = 1.2 kJ mol^–1^, Table S1), which (although is in the limit of accuracy of the method) is in very good agreement with the experimental values. Next, the exclusive formation of **7h** instead of **12h** in the elimination reaction of **4h** was investigated comparing the relative stability of both products. Figure 7 shows that both *cis*- and *trans*-**7h** are more stable than the corresponding isomers **12h**, independent on the conformation of the ring C.

**Figure 8:**
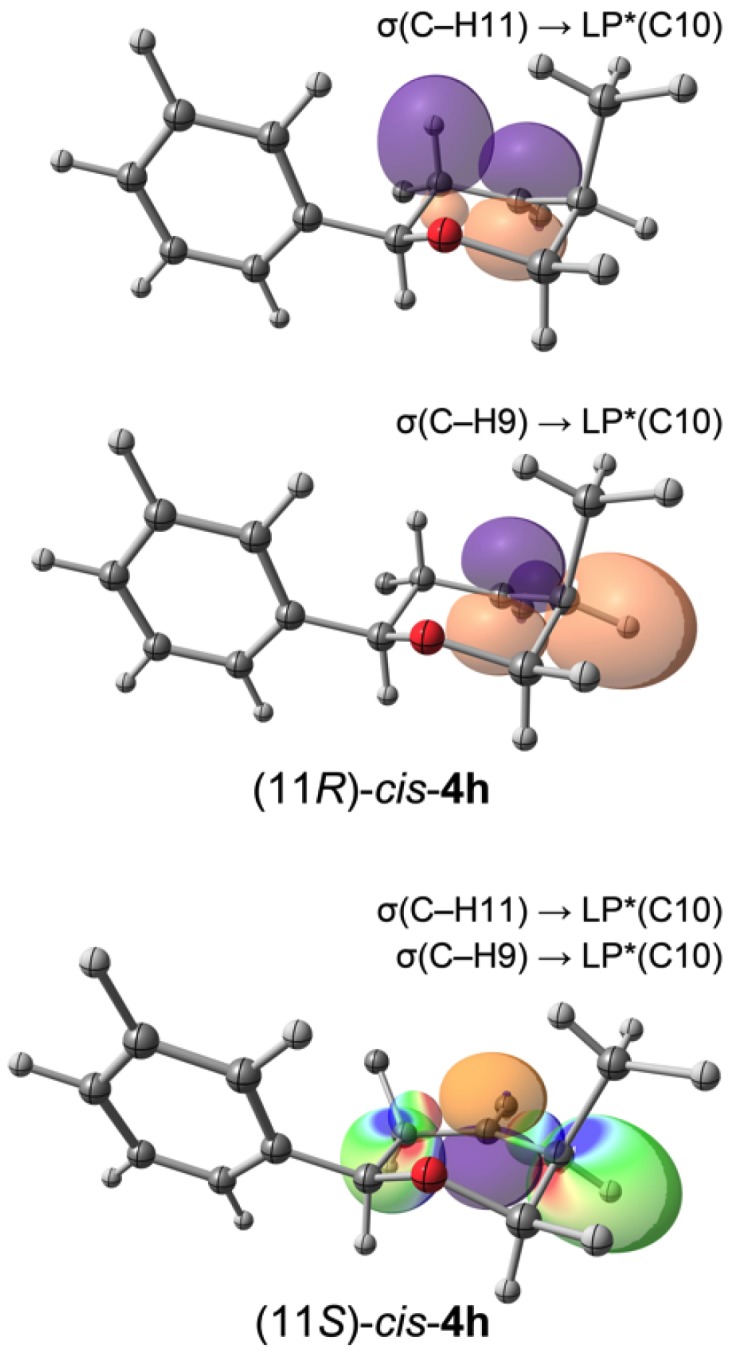
Relevant NBOs for the stablization of *cis*-**4h**. Some atoms were removed for clarity. Color mapped surfaces indicate orbital superposition in red and blue; contour value: 0.062.

Stereoelectronic effects in the elimination step (from **4** to **7**, Scheme 3) were investigated by natural bond orbital (NBO) analysis. The second order perturbation theory analysis of the NBOs indicate that the σ(C–H11) is a much better electron donor to the *p*-type antibonding lone pair (LP*) at the C10 than the σ(C–H9) for all diastereomers of **4h**, *i.e.*, stabilization energy of the σ(C–H11) → LP*(C10) is 35 kJ mol^−1^, whereas for σ(C–H9) the stabilization is negligible. However, for (11*S*)-*cis*-**4h** both donor-acceptor interactions σ(C–H11) → LP*(C10) and σ(C–H9) → LP*(C10) are stabilizing (40 kJ mol^−1^ and 16 kJ mol^−1^, respectively). The occupancy of the LP*(C10) NBO of (11*R*)-*cis*-**4h** is 0.633 *vs.* 0.579 for (11*S*)-*cis*-**4h**; furthermore, atomic charges determined by natural population analysis (NPA) indicate charges of 0.346 and 0.400 for (11*R*)- and (11*S*)-*cis*-**4h**, respectively. Figure 8 depicts relevant NBOs for (11*R*)- and (11*S*)-*cis*-**4h**.

Briefly, results from theoretical calculations indicate that in the case of **3h**, the formation of the *E* isomer is, as predicted before [31,45], preferable to other isomers because rotation around the φ_1_ dihedral angle and *E*/*Z* isomerization of oxocarbenion ions **3** are both not thermodynamic favorable (Table S1 and Scheme 5). The relative stability of one of the *E* isomers favors the formation of the (11*R*)-*cis*-**4h** carbocation compared to (11*S*)-*cis*-**4h**, which originates from the electrophilic attack from the opposite face of the 1,2-dihydronaphtalene ring. The activation barrier for the formation of the *cis*- and *trans*-**4h** indicates an earlier transition state in the case of the conversion of (*Z*)-**3h** to *trans*-**4h**. Therefore, if the substitution pattern favors the *Z* isomer of the oxocarbenium ion **3**, the preferential formation of the *trans* product would be possible. The formation of the product **7** is related to its higher stability when compared to **12**. Furthermore, NBO analysis indicate that between the two possible *cis*-**4h** intermediates, the 11*R* has the H11 in a better alignment with the *p*-type LP*(C10), reducing the overall charge at the carbon and favoring the formation of product *cis*-**7h** by elimination.

## 3. Experimental

### 3.1. Materials and Methods

All commercially available reagents were used without further purification unless otherwise noted. Commercially available isopulegol was purified by flash column chromatography (15% AcOEt in hexanes) and other homoallylic alcohols **1a**–**l** were synthesized as previously reported [36,46]. **1m** is commercially available. THF and Benzene were freshly distilled from sodium/benzophenone. CH_2_Cl_2_ was freshly distilled over CaH_2_. TLC analyses were performed in silica gel plates, using UV and/or *p*-anisaldehyde solution for visualization. Flash column chromatography was performed using silica gel 200–400 mesh (Aldrich, St. Louis, USA). Melting points are uncorrected. All NMR analyses were recorded using CDCl_3_ as solvent and TMS as internal pattern in Bruker (AC200) or Varian (INOVA300) spectrometers. IR spectra were measured on a Perkin-Elmer 1750-FT. HRMS analysis were performed on a Bruker Daltonics Microtof Eletrospray. The experimental procedures for the preparation of compounds **6a**, **7a**–**b** were previously reported [31].

### 3.2. Prins Cyclizations

*Prins cyclization of* **1a** *and* **2c***. General Procedure for Iodine-Catalyzed Prins cyclization*. To a stirred solution of **1a** (0.078 g, 0.414 mmol) and **2c** (0.050 mL, 0.41 mmol) in CH_2_Cl_2_ (5 mL), was added I_2_ (0.0052, 0.021 mmol). The mixture was refluxed for 3 h. Na_2_SO_3_ (0.0030 g, 0.021 mmol) and H_2_O (10 mL) were added. The aqueous phase was extracted with AcOEt (3 × 5 mL). The combined organic was washed with brine (5 mL) and dried over anhydrous MgSO_4_. The solvent was removed under reduced pressure. The crude product was purified by flash column chromatography (5% AcOEt in hexane), affording **7c** (*cis:trans* = 1.3:1, 0.11 g, 0.35 mmol, 84%) as colorless viscous oil. The relative configuration was assigned by NMR analysis, including NOESY experiments of enriched samples of *cis*-**7c** and *trans-***7c** that were obtained after successive purifications of the product by flash column chromatography (1% AcOEt in hexanes).

*(±)-cis-2,4,5,6-Tetrahydro-4-(2-methoxyphenyl)-1-methyl-1H-benzo[f]isochromene* (*cis*-**7c**). IR (film): 3063, 2960, 2936, 2836, 1599, 1491, 1462, 756, 736 cm^−1^. ^1^H-NMR (300 MHz, CDCl_3_) δ: 1.41 (d, *J* = 6.9 Hz, 3H), 1.77–1.87 (m, 1H), 1.94–2.05 (m, 1H), 2.67 (t, *J* = 7.9 Hz, 2H), 2.76–2.78 (m, 1H), 3.83–3.87 (m, 1H), 3.87 (s, 3H), 3.98 (dd, *J* = 10.8, 3.3 Hz, 1H), 5.70 (s, 1H), 6.91–6.97 (m, 2H), 7.08–7.16 (m, 2H), 7.21–7.27 (m, 1H), 7.29–7.38 (m, 3H). ^13^C-NMR (75 MHz, CDCl_3_) δ: 18.5, 24.4, 28.0, 28.8, 55.6, 70.2, 73.0, 110.9, 120.7, 122.1, 126.4, 127.6, 128.5, 129.3, 131.8, 133.7, 134.0, 135.8, 157.9. LRMS *m/z* (rel. int.): 306 (M^+∙^, 25), 264 (17), 245 (13), 231 (10), 199 (11), 141 (17), 135 (100). HRMS [ESI(+)] calcd. for [C_21_H_22_O_2_+Na^+^] 329.1517, found 329.1494.

(*±)-trans-2,4,5,6-Tetrahydro-4-(2-methoxyphenyl)-1-methyl-1H-benzo[f]-isochromene* (*trans*-**7c**). IR (film): 3060, 3018, 2959, 2929, 2835, 1599, 1587, 1489, 1462, 756, 736 cm^−1^. ^1^H-NMR (300 MHz, CDCl_3_) δ: 1.26 (d, *J* = 7.0 Hz, 3H), 1.81–2.04 (m, 2H), 2.62–2.81 (m, 3H), 3.54 (dd, *J* = 11.2, 2.6 Hz, 1H), 3.81–3.95 (m, 1H), 3.90 (s, 3H), 5.75 (s, 1H), 6.86–6.96 (m, 2H), 7.11–7.39 (m, 6H). ^13^C-NMR (75 MHz, CDCl_3_) δ: 18.2, 25.8, 28.1, 28.3, 55.6, 66.1, 71.5, 110.8, 119.7, 122.3, 126.3, 126.7, 127.6, 129.3, 129.6, 132.3, 132.8, 133.7, 136.3, 158.3. LRMS *m/z* (rel. int.): 306 (M^+∙^, 32), 264 (18), 245 (16), 231 (8), 199 (11), 141 (19), 135 (100). HRMS [ESI(+)] calcd. for [C_21_H_22_O_2_+Na^+^] 329.1517, found 329.1511.

*Prins cyclization of* **1a** *and* **2d**. The reaction was performed following the general procedure, but using **1a** (0.096 g, 0.51 mmol), **2d** (0.060 mL, 0.41 mmol), CH_2_Cl_2_ (5 mL), I_2_ (0.0065, 0.025 mmol). A mixture of **7d** and **12d** (*cis:trans:***12d** = 4.2:2.5:1, 0.12 g, 0.43 mmol, 84%) was obtained as a colorless viscous oil. This mixture was subjected to another flash column chromatography (1% AcOEt in hexanes). Partially pure samples could be obtained for characterization separately.

*(±)-cis-2,4,5,6-Tetrahydro-1-methyl-4-p-tolyl-1H-benzo[f]isochromene* (***cis*-7d**). IR (film): 3058, 3024, 2954, 2919, 2849, 1272, 1178, 1109, 766, 754 cm^−1^. ^1^H-NMR (200 MHz, CDCl_3_) δ: 1.43 (d, *J* = 6.8 Hz, 3H), 1.68–2.05 (m, 2H), 2.35 (s, 3H), 2.67 (t, *J* = 7.6 Hz, 2H), 2.73–2.78 (m, 1H), 3.88 (dd, *J* = 10.9, 1.8 Hz, 1H), 3.97 (dd, *J* = 10.8, 2.8 Hz, 1H), 5.09 (s, 3H), 7.08–7.19 (m, 3H), 7.21–7.29 (m, 3H), 7.30–7.47 (m, 2H). ^13^C-NMR (50 MHz, CDCl_3_) δ: 18.7, 21.2, 24.5 28.0, 28.9, 70.0, 80.5, 122.1, 126.5, 126.6, 127.7, 128.6, 129.2, 131.9, 133.1, 133.8, 135.7, 137.7, 138.0. LRMS *m/z* (rel. int.): 290 (M^+∙^, 18), 276 (14), 275 (72), 257 (8), 247 (25), 229 (10), 215 (8), 203 (13), 171 (9), 155 (11), 128 (29), 127 (22), 119 (93), 91 (88), 65 (51), 43 (100). HRMS [ESI(+)] calcd. for [C_21_H_22_O+Na]^+^ 313.1563, found 313.1535.

*(±)-(4R,4aR)-4,4a,5,6-Tetrahydro-1-methyl-4-p-tolyl-2H-benzo[f]isochromene* (**12d**) and *(±)-trans-2,4,5,6-tetrahydro-1-methyl-4-p-tolyl-1H-benzo[f]isochromene* (***trans*-7d**). IR (film): 3059, 3020, 2925, 2873, 1716, 1452, 1273, 1103, 1038, 816, 760 cm^−1^. ^1^H-NMR (200 MHz, CDCl_3_) δ: **12d**: 1.21–1.81 (m, 2H), 1.92 (s, 3H), 2.53 (s, 3H), 2.60–2.87 (m , 3H), 4.10 (d, *J* = 9.6 Hz, 1H), 4.24 (d, *J* = 17.2 Hz, 1H), 4.35 (d, *J* = 16.6 Hz, 1H), 7.11–7.41 (m, 7H), 7.44 (d, *J* = 3.0 Hz, 1H), *trans*-**7d**: 1.22 (d, *J* = 6.8 Hz, 3H), 2.34 (s, 3H), 3.50 (dd, *J* = 11.3, 3.3 Hz, 1H), 3.99 (d, *J* = 11.3, 3.7 Hz, 1H), 5.17 (s, 1H). Other signals overlap with the major diastereomer. ^13^C-NMR (75 MHz, CDCl_3_) δ: **12d**: 16.6, 21.2, 26.1, 28.3, 40.7, 71.3, 83.3, 125.0, 126.3, 126.5, 126.6, 127.6, 129.1, 128.4, 128.9, 129.1, 132.6, 136.2, 137.7, 137.9, 138.0, 138.1. *trans*-**7d**: 17.9, 21.2, 26.0, 28.2, 28.2, 66.7, 78.8, 122.6, 127.0, 127.3, 127.6, 129.2, 129.6, 129.7, 130.2, 132.6, 133.5, 133.7, 134.8, 136.2, 136.3. LRMS *m/z* (rel. int.): 290 (M^+∙^, 17), 275 (72), 247 (25), 229 (10), 215 (8), 203 (13), 155 (11), 128 (29), 127 (21), 119 (93), 91 (88), 65 (51), 43 (100). HRMS [ESI(+)] calcd. for [C_21_H_22_O+Na]^+^ 313.1563, found 313.1532.

*Prins cyclization of* **1a** *and* **2e**. The reaction was performed following the general procedure, but using **1a** (0.080 g, 0.42 mmol), **2e** (0.050 mL, 0.42 mmol), CH_2_Cl_2_ (5 mL), I_2_ (0.0054, 0.021 mmol). A mixture of **7e** and **12e** (*cis:trans:***12e** = 7.7:2.5:1, 0.11 g, 0.37 mmol, 86%) was obtained as colorless viscous oil. This mixture was subjected to another flash column chromatography (1% AcOEt in hexanes) for characterization.

*(±)-2,4,5,6-Tetrahydro-1-methyl-4-m-tolyl-1H-benzo[f]isochromene* (**7e**). IR (film): 3060, 3022, 2963, 2930, 1718, 1488, 1459, 1451, 1278, 1200, 1115, 766, 746 cm^−1^. ^1^H-NMR (200 MHz, CDCl_3_) δ: *cis*-**7e**: 1.45 (d, *J* = 6.8 Hz, 3H), 1.69–2.06 (m, 2H), 2.34 (s, 3H), 2.67 (t, *J* = 8.0 Hz, 2H), 2.74–2.78 (m, 1H), 3.97 (dd, *J* = 10.8, 2.8 Hz, 1H), 3.88 (dd, *J* = 10.9, 1.8 Hz, 1H), 5.09 (s, 3H), 7.08–7.19 (m, 3H), 7.21–7.29 (m, 3H), 7.30–7.47 (m, 2H). *trans*-**7e**: 1.22 (d, *J* = 6.8 Hz, 3H), 2.37 (s, 3H), 3.51 (dd, *J* = 11.2, 4 Hz, 1H), 4.41 (dd, *J* = 10.5, 2.8 Hz, 1H). Other signals overlap with the major diastereomer. ^13^C-NMR (50 MHz, CDCl_3_) δ: *cis*-**7e:** 18.8, 21.4, 24.4, 28.0, 28.9, 70.4, 80.8, 122.1, 125.3, 125.8, 126.5, 126.6, 127.7, 128.4, 128.5, 129.0, 129.3, 131.9, 133.0, 133.8, 135.7, 138.2, 140.5. LRMS *m/z* (rel. int.): 290 (M^+∙^, 37), 249 (11), 248 (69), 247 (39), 233 (25), 215 (11), 155 (17), 141 (15), 129 (33), 119 (100). HRMS [ESI(+)] calcd. for [C_21_H_22_O+Na]^+^ 313.1563, found 313.1541.

*(±)-(4R,4aR)-4,4a,5,6-Tetrahydro-1-methyl-4-m-tolyl-2H-benzo[f]isochromene* (**12e**). IR (film): 3094, 2925, 2869, 1648, 1484, 1445, 1101, 913, 760, 745 cm^−1^. ^1^H-NMR (500 MHz, CDCl_3_) δ: 1.48–1.56 (m, 1H), 1.75–1.81 (m, 1H), 1.92 (t, *J* = 1.0 Hz, 3H), 2.36 (s, 3H), 2.59–2.64 (m, 1H), 2.67–2.73 (m, 1H), 2.76–2.82 (m, 1H), 4.10 (d, *J* = 9.5 Hz, 1H), 4.26 (ddd, *J* = 16.5, 3.0, 1.0 Hz, 1H), 4.35 (dd, *J* = 16.5, 1.0 Hz, 1H), 7.08–7.10 (m, 1H), 7.12–7.15 (m, 2H), 7.16–7.18 (m, 1H), 7.19–7.20 (m, 1H), 7.24–7.27 (m, 2H), 7.43 (dd, *J* = 7.5, 1.5, 1H). ^13^C-NMR (75 MHz, CDCl_3_) δ: 16.6, 21.4, 26.1, 28.3, 40.7, 71.3, 83.5, 124.8, 125.0, 126.7, 126.9, 128.2, 128.3, 128.4, 128.8, 128.9, 129.7, 134.8, 137.9, 138.1, 141.0. LRMS *m/z* (rel. int.): 290 (M^+∙^, 1.4), 233 (9.8), 215 (2.5), 170 (100). HRMS [ESI(+)] calcd. for [C_21_H_22_O+Na]^+^ 313.1563, found 313.1555.

*Prins cyclization of* **1a** *and* **2f**. The reaction was performed following the general procedure, but using **1a** (0.081 g, 0.43 mmol), **2f** (0.050 mL, 0.41 mmol), CH_2_Cl_2_ (5 mL), I_2_ (0.0055, 0.022 mmol). Compound **3f** (*cis:trans* = 1.5:1, 0.089 g, 0.30 mmol, 70%) was obtained as colorless viscous oil. This mixture was subjected to another flash column chromatography (1% AcOEt in hexanes). Pure samples could be obtained for characterization.

*(±)-cis-2,4,5,6-Tetrahydro-1-methyl-4-o-tolyl-1H-benzo[f]isochromene* (***cis*-7f**). White solid, m.p. 155–157 °C. IR (film): 3060, 3017, 2954, 2919, 2850, 1486, 1458, 1122, 761, 734 cm^−1^. ^1^H-NMR (300 MHz, CDCl_3_) δ: 1.38 (d, *J* = 6.9 Hz, 3H), 1.79–2.04 (m, 2H), 2.50 (s, 3H), 2.66–2.72 (m, 2H), 2.79–2.81 (m, 1H), 3.83 (dd, *J* = 11.1, 2.4 Hz, 1H), 3.96 (dd, *J* = 11.1, 3.3 Hz, 1H), 5.40 (s, 1H), 7.10–7.15 (m, 2H), 7.17–7.21 (m, 3H), 7.25–7.27 (m, 1H), 7.31–7.35 (m, 2H). ^13^C-NMR (75 MHz, CDCl_3_) δ: 18.3, 19.3, 24.6, 27.9, 28.8, 70.2, 77.2, 122.1, 126.1, 126.5, 127.7, 128.0, 130.6, 1334, 133.8, 135.8, 137.4, 138.2. LRMS *m/z* (rel. int.): 290 (M^+∙^, 73), 248 (70), 247 (55), 234 (17), 233 (100). HRMS [ESI(+)] calcd. for [C_21_H_22_O+Na]^+^ 313.1563, found 313.1584.

*(±)-trans-2,4,5,6-Tetrahydro-1-methyl-4-o-tolyl-1H-benzo[f]isochromene* (***trans*-7f**). Viscous oil. IR (film): 3062, 3017, 2962, 2918, 2897, 1485, 1457, 1119, 1036, 765, 749, 739 cm^−1^. ^1^H-NMR (500 MHz, CDCl_3_) δ: 1.25 (d, *J* = 6.5 Hz, 3H), 1.85 (ddd, *J* = 15.5, 5.1, 5.0 Hz, 1H), 2.00–2.04 (m, 1H), 2.50 (s, 3H), 2.64–2.85 (m, 3H), 3.54 (dd, *J* = 11.5, 3.0 Hz, 1H), 3.92–3.94 (m, 1H), 5.45 (s, 1H), 7.09–7.17 (m, 4H), 7.19–7.23 (m, 2H), 7.29–7.32 (2H). ^13^C-NMR (75 MHz, CDCl_3_) δ: 18.2, 19.2, 26.2, 28.2, 28.3, 29.7, 77.2, 122.5, 125.2, 126.4, 126.5, 127.6, 128.1, 129.0, 130.9, 132.9, 133.7, 136.3, 136.5, 138.4. LRMS *m/z* (rel. int.): 290 (M^+∙^, 48), 248 (47), 247 (35), 233 (65), 215 (13), 199 (11), 128 (26), 119 (100). HRMS [ESI(+)] calcd. for [C_21_H_22_O+Na]^+^ 313.1563, found 313.1573.

*Prins cyclization of* **1a** *and* **2g**. The reaction was performed following the general procedure, but using **1a** (0.17 g, 0.89 mmol), **2g** (0.12 mL, 0.89 mmol), CH_2_Cl_2_ (5 mL), I_2_ (0.011 g, 0.045 mmol). A mixture of **7g** and **12g** (*cis:trans:***12g** = 1.4:1:6.2, 0.16 g, 0.53 mmol, 60%) was obtained as colorless viscous oil. This mixture was subjected to another flash column chromatography (1% AcOEt in hexanes). Partially pure samples could be obtained for characterization.

*(±)-(4R,4aR)-4,4a,5,6-Tetrahydro-1-methyl-4-(2,6-dimethylphenyl)-2H-benzo[f]isochromene* (**12g**). Solid, m.p: 148–150 °C. IR (film): 3066, 3015, 2919, 2857, 2797, 1443, 1374, 1106, 1095, 762, 735 cm^−1^. ^1^H-NMR (200 MHz, CDCl_3_) δ: 1.43–1.64 (m, 1H), 1.69–1.84 (m, 1H), 1.95 (s, 3H), 2.42 (s, 3H), 2.51 (s, 3H), 2.73–2.97 (m, 3H), 4.19 (d, *J* = 16.6 Hz, 1H), 4.35 (d, *J* = 16.4 Hz, 1H), 4.67 (d, *J* = 10.0 Hz, 1H), 7.04–7.22 (m, 6H), 7.43–7.47 (m, 1H). ^13^C-NMR (75 MHz, CDCl_3_) δ: 16.8, 27.4, 28.4, 38.9, 71.3, 79.2, 124.9, 126.7, 127.3, 127.5, 128.8, 129.1, 129.8, 134.7, 136.6, 137.4. LRMS *m/z* (rel. int.): 304 (M^+∙^, 0.34), 247 (2.5), 232 (1.0), 171 (9.7), 170 (100). HRMS [ESI(+)] calcd. for [C_22_H_24_O+Na] 327.1725, found 327.1732.

*(±)-2,4,5,6-Tetrahydro-1-methyl-4-(2,6-dimethylphenyl)-1H-benzo[f]isochromene* (**7g**). IR (film): 3095, 3062, 3021, 2917, 2855, 2731, 1486, 1377, 1126, 1101, 776, 729 cm^−1^. ^1^H-NMR (500 MHz, CDCl_3_) δ: *cis*-**7g**: 1.44 (d, *J* = 7.0 Hz, 1H), 1.74–1.82 (m, 2H), 2.19 (s, 3H), 2.42 (s, 3H), 2.51–2.93 (m, 3H), 4.08 (d, *J* = 11.2, 1.5 Hz, 1H), 4.05 (d, *J* = 11.5, 2.7 Hz, 1H), 5.59 (s, 1H), 6.98–7.24 (m, 7H), 7.30 (d, *J* = 7.0 Hz, 1H), *trans*-**7g**: 1.05 (d, *J* = 7.0 Hz, 1H), 1.50–1.71 (m, 2H), 2.48 (s, 3H), 2.49 (s, 3H), 3.16–3.22 (m, 1H), 3.52 (d, *J* = 11.5, 10.5Hz, 1H), 4.32 (d, *J* = 11.2, 6.0 Hz, 1H), 5.72 (s, 1H), 6.88 (d, J = 7.0 Hz, 1H). Other signals overlap with the major diastereomer. ^13^C-NMR (50 MHz, CDCl_3_) δ: *cis*-**7g**: 17.9, 21.0, 21.2, 23.7, 28.1, 28.6, 72.3, 77.6, 122.0, 123.3, 125.9, 126.39, 126.45, 127.6, 127.8, 128.2, 129.9, 132.3, 133.9, 134.0, 135.7, 137.5, 138.4. *trans*-**7g**: 16.1, 19.6, 21.0, 25.1, 27.8, 28.3, 72.3, 76.2, 125.3, 127.2, 127.3, 127.7, 128.0, 129.9, 134.0, 134.7, 135.7, 136.2, 137.0, 138.5. LRMS *m/z* (rel. int.): *cis*-**7g**: 304 (M^+∙^, 50), 262 (31), 261 (22), 248 (11), 247 (71), 229 (17), 152 (14), 141 (20), 133 (100), *trans*-**7g**: 304 (67), 262 (45), 261 (30), 248 (15), 247 (100). HRMS [ESI(+)] calcd. for [C_22_H_24_O+Na] 327.1725, found 327.1717.

*Prins cyclization of* **1a** *and* **2i**. The reaction was performed following the general procedure, but using **1a** (0.075 g, 0.40 mmol), **2i** (0.065 g, 0.40 mmol), CH_2_Cl_2_ (5 mL), I_2_ (0.0051 g, 0.020 mmol). A mixture of **7i** (*cis:trans* = 20:3.2, 0.933 g, 0.28 mmol, 70%) was obtained as colorless viscous oil.

*(±)-N-(4-(2,4,5,6-tetrahydro-1-methyl-1H-benzo[f]isochromen-4-yl)phenyl)acetamide* (**7i**). IR (film): 3308, 3197, 3123, 3059, 2964, 2929, 2871, 1684, 1671, 1601, 1540, 1411, 1372, 1318, 1267, 767 736 cm^−1^. ^1^H-NMR (200 MHz, CDCl_3_) δ: *cis*-**7i**: 1.43 (d, *J* = 6.8 Hz, 3H), 1.64–1.98 (m, 2H), 2.15 (s, 3H), 2.60–2.68 (m, 2H), 2.73–2.78 (m, 1H), 3.87 (d, *J* = 10.9, 2.0 Hz, 1H), 3.96 (d, *J* = 11.0, 3.0 Hz, 1H), 5.09 (s, 1H), 7.06–7.20 (m, 3H), 7.23–7.51 (m, 5H).

*trans*-**7i**: 1.21 (d, *J* = 6.8 Hz, 3H), 2.08 (s, 3H), 3.50 (dd, *J* = 11.4, 3.8 Hz, 1H), 5.16 (s, 1H). Other signals overlap with the major compound. ^13^C-NMR (75 MHz, CDCl_3_) δ: *cis*-**7i**: 18.8, 24.4, 24.6, 27.9, 28.8, 70.3, 80.2, 119.7, 122.1, 126.5 126.7, 127.7, 129.3, 132.0, 133.7, 135.7, 137.8, 168.3. *trans*-**7i**: 17.9, 24.6, 28.1, 28.2, 77.2, 78.6, 119.7, 122.6, 125.3, 126.3, 126.5, 127.6, 129.8, 132.9, 136.6, 137.1. LRMS *m/z* (rel. int.): 333 (M^+∙^, 28), 292 (13), 291 (69), 290 (45), 288 (20), 249 (18), 233 (13), 162 (55), 155 (12), 141 (13), 129 (15), 128 (23), 115 (16), 43 (100). HRMS [ESI(+)] calcd. for [C_22_H_23_O_2_+Na] 356.1626, found 356.1633.

*Prins cyclization of* **1a** *and* **2j**. The reaction was performed following the general procedure, but using **1a** (0.056 g, 0.30 mmol), **2j** (0.055 g, 0.30 mmol), CH_2_Cl_2_ (5 mL), I_2_ (0.0038 g, 0.015 mmol). A mixture of **7j** and **12j** (*cis:trans:***12j** = 12.5:0.01:1, 0.091 g, 0.26 mmol, 85%) was obtained as a colorless viscous oil. Aldehyde **2j** (12%) was also recovered. This mixture was subjected to another flash column chromatography (1% AcOEt in hexanes). Partially pure samples could be obtained for characterization.

*(±)-cis-4-(4-Bromophenyl)-2,4,5,6-tetrahydro-1-methyl-1H-benzo[f]isochromene* (***cis*-7j**). IR (film): 3065, 2962, 2927, 1712, 1487, 1462, 1453, 1276, 768, 735 cm^−1^. ^1^H-NMR (200 MHz, CDCl_3_) δ: 1.43 (d, *J* = 7.0 Hz, 3H), 1.67–2.05 (m, 2H), 2.57–2.80 (m, 3H), 3.87 (dd, *J* = 11.0, 2.0 Hz, 1H), 3.95 (dd, *J* = 11.0, 3.0 Hz, 1H), 5.08 (s, 1H), 7.07–7.12 (m, 1H), 7.15–7.21 (m, 1H), 7.24–7.35 (m, 4H), 7.46 (t, *J* = 2.0 Hz, 1H), 7.51 (t, *J* = 1.8 Hz, 1H). ^13^C-NMR (50 MHz, CDCl_3_) δ: 18.8, 24.3, 27.9, 28.8, 70.4, 80.1, 122, 126.5, 126.8, 127.7, 130.3, 131.7, 132.2, 132.3, 133.5, 135.6, 139.8. LRMS *m/z* (rel. int.): 356 (M^+∙^+2, 20), 354 (M^+∙^, 20), 314 (47), 312 (47), 233 (46), 215 (32), 185 (60), 183 (60), 129 (100). HRMS [ESI(+)] calcd. for [C_20_H_19_BrO+H] 355.0698, found 355.0549.

*(±)-(4R,4aR)-4-(4-bromophenyl)-4,4a,5,6-tetrahydro-1-methyl-2H-benzo[f]isochromene* (**12j**) and *(±)-trans-4-(4-bromophenyl)-2,4,5,6-tetrahydro-1-methyl-1H-benzo[f]isochromene* (***trans*-7j**). IR (film): 3067, 2956, 2926, 1719, 1590, 1484, 1454, 1271, 757, 733 cm^−1^. ^1^H-NMR (200 MHz, CDCl_3_) δ: **12j**: 1.37–2.05 (m, 2H), 1.92 (s, 3H), 2.60–2.79 (m, 3H), 4.10 (d, J = 9.6 Hz, 3H), 4.23 (dd, J = 16.5, 1.4 Hz, 1H), 4.35 (d, J = 15.4 Hz, 1H), 7.07–7.52 (m, 7H), 7.66–7.78 (m, 1H). *trans*-**7j**: 1.21 (d, J = 6.8 Hz, 3H), 3.50 (dd, J = 11.4, 4.0 Hz, 1H), 5.16 (s, 1H). Other signals overlap with the major diastereomer. ^13^C-NMR (50 MHz, CDCl_3_) δ: **12j**: 16.5, 26.0, 28.2, 40.8, 67.0, 78.4, 125.1, 125.3, 126.7, 126.9, 128.4, 128.9, 129.3, 130.8, 131.0, 131.5, 131.8, 132.4, 134.6, 137.7. *trans*-**7j**: 17.8, 25.9, 28.1, 28.2, 71.2, 82.3, 121.9, 122.3, 122.7, 126.8, 127.6, 129.4, 129.8, 133.1, 133.5, 135.1, 136.2, 138.2, 140.2. LRMS *m/z* (rel. int.): 356 (M^+∙^+2, 20), 354 (M^+∙^, 20), 314 (41), 312 (47), 233 (40), 215 (27), 185 (47), 183 (53), 157 (17), 155 (29), 129 (100). HRMS [ESI(+)] calcd. for [C_20_H_19_BrO+H] 355.0698, 357.0677, found 355.0319, 357.0301.

*Prins cyclization of* **1a** *and* **2k**. The reaction was performed following the general procedure, but using **1a** (0.39 g, 2.1 mmol), **2k** (0.24 mL, 2.1 mmol), CH_2_Cl_2_ (10 mL), I_2_ (0.026 g, 0.10 mmol). Compound *cis*-**7k** (0.55 g, 1.5 mmol, 75%) was obtained as a colorless solid.

*(±)-cis-4-(3-Bromophenyl)-2,4,5,6-tetrahydro-1-methyl-1H-benzo[f]isochromene* (***cis*-7k**). m.p. 154 °C. IR (film): 3060, 2959, 2917, 1487, 1460, 788, 732 cm^−1^. ^1^H-NMR (500 MHz, CDCl_3_) δ: 1.45 (d, *J* = 6.5 Hz, 3H), 1.75–1.81 (m, 1H), 1.94–2.00 (m, 1H), 2.61–2.72 (m, 2H), 2.76–2.77 (m, 1H), 3.88 (dd, *J* = 11.0, 2.0 Hz, 1H), 3.95 (dd, *J* = 11.0, 3.0 Hz, 1H), 5.08 (s, 1H), 7.10 (d, *J* = 7.0 Hz, 1H), 7.16 (td, *J* = 7.5, 1.0 Hz, 1H), 7.21–7.26 (m, 2H), 7.33 (d, *J* = 6.5 Hz, 1H), 7.34 (d, *J* = 7.5 Hz, 1H), 7.44–7.46 (m, 1H), 7.55 (t, *J* = 1.5 Hz, 1H). ^13^C-NMR (75 MHz, CDCl_3_) δ: 18.8, 24.3, 27.9, 28.9, 70.4, 80.2, 122.2, 122.6, 126.5, 126.8, 127.3, 127.7, 130.1, 131.4, 131.7, 132.0, 132.4, 133.5, 135.6, 143.0. LRMS *m/z* (rel. int.): 356 (M^+∙^+2, 25), 354 (M^+∙^, 25), 314 (45), 312 (46), 233 (33), 215 (25), 185 (36), 183 (34), 157 (22), 155 (22), 129 (100). HRMS [ESI(+)] calcd. for [C_20_H_19_BrO+Na] 377.0518, 379.0509, found 377.0516, 379.0504.

*Prins cyclization of* **1a** *and* **2l**. The reaction was performed following the general procedure, but using **1a** (0.094 g, 0.50 mmol), **2l** (0.092 g, 0.50 mmol), CH_2_Cl_2_ (5 mL), I_2_ (0.0063, 0.025 mmol). A mixture of **7l** and **12l** (*cis:***12l** = 11.1:1, 0.11 g, 0.31 mmol, 62%) was obtained as a colorless viscous oil.

*(±)-cis-4-(2-Bromophenyl)-2,4,5,6-tetrahydro-1-methyl-1H-benzo[f]isochromene* (***cis*-7l**) and *(±)-(4R,4aR)-4-(2-bromophenyl)-4,4a,5,6-tetrahydro-1-methyl-2H-benzo[f]isochromene* (**12l**). IR (film): 3062, 2962, 2928, 1470, 1438, 759, 732 cm^−1^. ^1^H-NMR (500 MHz, CDCl_3_) δ: cis**-7l**: 1.43 (d, *J* = 7.0 Hz, 3H), 1.75–1.82 (m, 1H), 2.00–2.08 (m, 1H), 2.60–2.73 (m, 2H), 2.77–2.81 (m, 1H), 3.88 (dd, *J* = 10.7, 2.5 Hz, 1H), 4.00 (dd, *J* = 10.7, 3.5 Hz, 1H), 5.69 (s, 1H), 7.01–7.18 (m, 3H), 7.28–7.46 (m, 3H), 7.45 (dd, *J* = 7.7, 1.5 Hz, 1H), 7.59 (dd, *J* = 7.7, 1.5 Hz, 1H). **12l**: 1.93 (d, *J* = 1.0 Hz, 3H), 4.33 (s, 2H), 4.78 (d, *J* = 9.5 Hz, 2H). Other signals overlap with the major diastereomer. ^13^C-NMR (75 MHz, CDCl_3_) δ: *cis***-7l**: 18.8, 24.3, 27.9, 28.8, 70.4, 78.7, 122.2, 125.5, 126.5, 126.7, 127.7, 127.8, 129.7, 129.9, 132.4, 132.7, 132.9, 133.6, 135.7, 139.7. **12l**: 16.5, 25.7, 28.4, 41.2, 71.6, 80.4, 125.1, 125.46, 125.53, 127.9, 128.3, 128.8, 129.0, 129.4, 132.6. LRMS *m/z* (rel. int.): 356 (M^+∙^+2, 21), 354 (M^+∙^, 24), 314 (38), 312 (38), 232 (55), 215 (34), 185 (34), 183 (38), 157 (19), 155 (20), 129 (83), 128 (100). HRMS [ESI(+)] calcd. for [C_20_H_19_BrO+Na] 377.0517, 379.0497, found 377.0516, 379.0504.

*Prins cyclization of* **1a** *and* **2n**. The reaction was performed following the general procedure, but using **1a** (0.094 g, 0.50 mmol), **2n** (0.076 g, 0.50 mmol), CH_2_Cl_2_ (5 mL), I_2_ (0.0063, 0.025 mmol). Compound **7n** (*cis:trans* = 16.7:1, 0.12 g, 0.38 mmol, 76%) was obtained as a white solid.

*(±)-cis-2,4,5,6-Tetrahydro-1-methyl-4-(3-nitrophenyl)-1H-benzo[f]isochromene* (***cis*-7n**). m.p. 104–105 °C. IR (film): 3061, 3020, 2963, 2927, 2249, 1727, 1486, 1451, 768, 735 cm^−1^. ^1^H-NMR (500 MHz, CDCl_3_) δ: 1.43 (d, *J* = 7.0 Hz, 3H), 1.74–1.80 (m, 1H), 2.08–2.16 (m, 1H), 2.59–2.73 (m, 2H), 2.78–2.82 (m, 1H), 3.86 (dd, *J* = 11.0, 2.0 Hz, 1H), 3.98 (dd, *J* = 11.0, 3.0 Hz, 1H), 5.24 (s, 1H), 7.11 (dd, *J* = 7.5, 1.0 Hz, 1H), 7.16 (td, *J* = 7.5, 1.5 Hz, 1H), 7.23–7.26 (m, 2H), 7.33 (d, *J* = 6.5 Hz, 1H), 7.44–7.48 (m, 1H), 7.58 (td, *J* = 7.0, 1.5 Hz 1H), 7.66 (dd, *J* = 8.0, 1.0 Hz, 1H), 7.84 (dd, *J* = 8.0, 1.0 Hz, 1H). ^13^C-NMR (75 MHz, CDCl_3_) δ: 19.0, 24.4, 27.8, 28.8, 70.5, 79.9, 122.3, 123.3, 123.7, 126.6, 127.1, 127.8, 129.5 131.1, 133.1, 133.3, 134.7, 135.5, 143.0, 148.4. LRMS *m/z* (rel. int.): 321 (M^+∙^, 11), 279 (33), 150 (28), 129 (100). HRMS [ESI(+)] calcd. for [C_20_H_19_NO_3_+Na] 344.1263, found 344.1253.

*Prins cyclization of* **1a** *and* **2o**. The reaction was performed following the general procedure, but using **1a** (0.094 g, 0.50 mmol), **2o** (0.076 g, 0.50 mmol), CH_2_Cl_2_ (5 mL), I_2_ (0.0063, 0.025 mmol). A mixture of **7o** and **12o** (*cis:***12o** = 10:1, 0.14 g, 0.42 mmol, 85%) was obtained as a colorless viscous oil.

*(±)-cis-2,4,5,6-Tetrahydro-1-methyl-4-(2-nitrophenyl)-1H-benzo[f]isochromene* (***cis*-7o**) and *(±)-(4R,4aR)-4,4a,5,6-tetrahydro-1-methyl-4-(2-nitrophenyl)-2H-benzo[f]isochromene* (**12o**). IR (film): 3065, 2962, 2928, 2253, 1527, 1488, 1450, 768, 735 cm^−1^. ^1^H-NMR (500 MHz, CDCl_3_) δ: *cis*-**7o**: 1.43 (d, *J* = 7.0 Hz, 3H), 1.74–1.80 (m, 1H), 2.08–2.16 (m, 1H), 2.59–2.73 (m, 2H), 2.78–2.82 (m, 1H), 3.86 (dd, *J* = 11.0, 2.0 Hz, 1H), 3.98 (dd, *J* = 11.0, 3.0 Hz, 1H), 5.79 (s, 1H), 7.11 (dd, *J* = 7.5, 1.0 Hz, 1H), 7.16 (td, *J* = 7.5, 1.5 Hz, 1H), 7.23–7.26 (m, 1H), 7.33 (d, *J* = 6.5 Hz, 1H), 7.44–7.48 (m, 1H), 7.58 (td, *J* = 7.0, 1.5 Hz, 1H), 7.66 (dd, *J* = 8.0, 1.0 Hz, 1H), 7.84 (dd, *J* = 8.0, 1.0 Hz, 1H). **12o**: 1.68–1.73 (m, 1H), 1.58–1.64 (m 1H), 1.91 (t, *J* = 1.0 Hz, 3H), 4.27 (dd, *J* = 16.5, 1.5 Hz, 1H), 4.32 (dd, *J* = 16.5, 1.0 Hz, 1H), 4.92 (d, *J* = 9.5 Hz, 1H), 7.41 (dd, *J* = 7.2, 1.5 Hz, 1H), 7.63 (dd, *J* = 7.2, 1.0 Hz, 1H), 7.72 (dd, *J* = 8.0, 1.5 Hz, 1H), 7.82 (dd, *J* = 8.2, 1.5 Hz, 1H). Other signals overlap with the major diastereomer. ^13^C-NMR (75 MHz, CDCl_3_) δ: *cis*-**7o**: 18.9, 24.4, 27.9, 28.6, 70.7, 74.3, 122.3, 123.9, 126.6, 127.0, 127.7, 129.0 130.4, 131.8, 132.8, 133.1, 133.3, 135.1, 135.7, 150.6. **12o**: 16.5, 25.7, 28.2, 40.8, 71.5, 76.2, 123.8, 125.1, 126.8, 127.1, 128.7, 128.8, 129.2, 129.3, 132.8, 134.6, 135.2, 138.0. LRMS *m/z* (rel. int.): 321 (M^+∙^, 1.7), 303 (22), 312 (46), 233 (33), 215 (25), 185 (36), 183 (34), 157 (22), 155 (22), 129 (100). HRMS [ESI(+)] calcd. for [C_20_H_19_NO_3_+Na] 344.1263, found 344.1263.

*Prins cyclization of* **1a** *and* **2q**. The reaction was performed following the general procedure, but using **1a** (0.084 g, 0.44 mmol), **2q** (0.040 mL, 0.44 mmol), CH_2_Cl_2_ (5 mL), I_2_ (0.0056, 0.022 mmol). Compound *cis*-**7q** (0.067 g, 0.28 mmol, 62%) was obtained as colorless viscous oil.

*(±)-cis-2,4,5,6-Tetrahydro-1-methyl-4-propyl-1H-benzo[f]isochromene* (***cis*-7q**). IR (film): 3065, 2965, 2936, 1726, 1457, 760 cm^−1^. ^1^H-NMR (200 MHz, CDCl_3_) δ: 0.93 (t, *J* = 7.2 Hz, 3H), 1.33 (d, *J* = 6.8 Hz, 3H), 1.25–1.85 (m, 4H), 1.94–2.30 (m, 2H), 2.50–2.62 (m, 1H), 2.73–2.81 (m, 2H), 3.75 (dd, *J* = 10.6, 2.5 Hz, 1H), 3.81 (dd, *J* = 10.6, 1.8 Hz, 1H), 4.22–4.24 (m, 1H), 7.11 (dd, *J* = 4.7, 1.4 Hz, 2H), 7.16–7.23 (m, 1H), 7.24–7.29 (m, 1H). ^13^C-NMR (75 MHz, CDCl_3_) δ: 14.3, 18.0, 18.6, 23.9, 28.2, 29.2, 35.2, 69.8, 76.6, 121.9, 126.3, 126.4, 127.5, 131.3, 133.8, 134.3, 135.3. LRMS *m/z* (rel. int.): 242 (M^+∙^, 31), 227 (20), 199 (29), 184 (36), 170 (26), 158 (32), 157 (24), 155 (100). HRMS [ESI(+)] calcd. for [C_17_H_22_O+Na] 265.1568, found 265.1556.

*Prins cyclization of* **1a** *and* **2r**. The reaction was performed following the general procedure, but using **1a** (0.0932 g, 0.496 mmol), **2r** (0.0600 mL, 0.496 mmol), CH_2_Cl_2_ (5 mL), I_2_ (0.0063, 0.024 mmol). Compound **7r** (*cis:trans* = 7.1:1, 0.10 g, 0.36 mmol, 72%) was obtained as colorless viscous oil.

*(±)-4-Cyclohexyl-2,4,5,6-tetrahydro-1-methyl-1H-benzo[f]isochromene* (**7r**). IR (film): 3091, 3063, 2930, 2853, 1711, 1451, 1451, 763 cm^−1^. ^1^H-NMR (300 MHz, CDCl_3_) δ: *cis*-**7r**: 1.09–1.28 (m, 4H), 1.34 (d, *J* = 6.9 Hz, 3H), 1.39–1.51 (m, 1H), 1.53–1.73 (m, 6H), 1.77–1.85 (m, 2H), 1.95–2.07 (m, 1H), 2.15–2.26 (m, 1H), 2.48–2.55 (m, 1H), 2.71–2.82 (m, 2H), 3.72 (dd, *J* = 10.6, 2.5 Hz, 1H), 3.81 (dd, *J* = 10.5, 1.2 Hz, 1H), 4.10 (s, 1H), 7.10–7.13 (m, 2H), 7.14–7.25 (m, 1H), 7.27 (d, *J* = 7.5 Hz, 1H). trans-**7r**: 3.02 (d, *J* = 9.6 Hz, 1H), 3.99 (dd, *J* = 16.2, 1.5 Hz, 1H), 4. 10 (s, 1H), 4.19 (dd, *J* = 16.2, 0.9 Hz, 1H), other signals overlap with major diastereomer. ^13^C-NMR (75 MHz, CDCl_3_) δ: *cis*-**7r**: 18.7, 23.6, 26.0, 26.4, 26.7, 27.2, 28.2, 29.4, 30.4, 39.7, 70.0, 81.2, 121.8, 126.2, 126.4, 127.4, 131.8, 133.0, 134.5, 135.3. *trans*-**7r**: 16.5, 26.1, 26.6, 26.9, 27.6, 28.4, 31.3, 35.6, 70.7, 84.3, 124.8, 126.5, 127.2, 128.3, 128.9, 129.8, 135.5, 137.6. LRMS *m/z* (rel. int.): 282 (M^+∙^, 20), 267 (9), 224 (6), 199 (30), 197 (6), 187 (12), 186 (89), 181 (16), 171 (32), 170 (68), 169 (20), 158 (44), 157 (24), 156 (16), 155 (100). HRMS [ESI(+)] calcd. for [C_20_H_26_O+H] 283.2062, found 283.2613.

*Prins cyclization of* **1a** *and* **2t**. The reaction was performed following the general procedure, but using **1a** (0.092 g, 0.49 mmol), **2t** (0.070 mL, 0.49 mmol), CH_2_Cl_2_ (5 mL), I_2_ (0.0062 g, 0.024 mmol). Compound **7t** (*cis:trans*= 2:1, 0.081 g, 0.34 mmol, 69%) was obtained as a colorless viscous oil. The relative configuration was assigned based on NOESY experiments of an enriched sample of *cis*-**7t** (*cis*:*trans =* 12:1) and enriched sample of *trans-***7t** (*cis:trans =* 1:10) that were obtained after successive purifications of the product by flash column chromatography (1% AcOEt in hexanes).

*(±)-cis-2,4,5,6-tetrahydro-1-methyl-4-((E)-prop-1-enyl)-1H-benzo[f]isochromene* (***cis*-7t**). IR (film): 2964, 2931, 2878, 2833, 1714, 1489, 1451, 1127, 768, 738 cm^−1^. ^1^H-NMR (300 MHz, CDCl_3_) δ: 1.30 (d, *J* = 6.9 Hz, 3H), 1.76 (dd, *J* = 6.4, 1.8 Hz, 3H), 1.94–2.03 (m, 1H), 2.05–2.20 (m, 1H), 2.63–2.67 (m, 1H), 3.73 (t, *J* = 7.2 Hz, 2H), 3.81 (s, 1H), 3.82 (s, 1H), 4.51 (d, *J* = 8.1 Hz, 1H), 5.46 (ddq, *J* = 15.1, 8.2, 1.8 Hz, 1H), 5.85 (dqd, *J* = 15.1, 6.4, 0.6 Hz, 1H), 7.11–7.13 (m, 2H), 7.15–7.27 (m, 2H). ^13^C-NMR (75 MHz, CDCl_3_) δ: 17.8, 18.4, 24.2, 28.1, 28.8, 69.6, 78.7, 122.0, 126.39, 126.42, 127.6, 129.1, 130.5, 131.0, 131.2, 132.9, 135.6. LRMS *m/z* (rel. int.): 240 (M^+∙^, 85), 225 (26), 198 (82), 197 (80), 183 (99), 181 (17), 179 (18), 177 (18), 165 (55), 155 (61), 153 (28), 152 (39), 141 (57), 129 (100). HRMS [ESI(+)] calcd. for [C_17_H_21_O+H]^+^ 241.1592, found 241.1589.

*(±)-trans-2,4,5,6-Tetrahydro-1-methyl-4-((E)-prop-1-enyl)-1H-benzo[f]isochromene* (***trans*-7t**). IR (film): 3067, 3022, 2964, 2931, 2878, 1708, 1451, 1380, 1127, 768, 738 cm^−1^. ^1^H-NMR (300 MHz, CDCl_3_) δ: 1.16 (d, *J* = 6.9 Hz, 3H), 1.72 (ddd, *J* = 9.0, 1.6, 0.6 Hz, 3H), 1.91–2.00 (m, 1H), 2.06–2.18 (m, 1H), 2.69–2.80 (m, 3H), 3.55 (dd, *J* = 11.2, 3.6 Hz, 1H), 4.03 (dd, *J* = 11.1, 3.9 Hz, 1H), 4.57 (d, *J* = 7.5 Hz, 1H), 5.51 (ddq, *J* = 15.4, 7.3, 1.5 Hz, 1H), 5.77 (dqd, *J* = 15.2, 6.4, 0.6 Hz, 1H), 7.12–7.14 (m, 2H), 7.15–7.19 (m, 2H). ^13^C-NMR (75 MHz, CDCl_3_) δ: 17.7, 17.9, 25.5, 28.19, 28.21, 67.2, 77.4, 122.4, 126.26, 126.29, 127.5, 128.2, 130.5, 131.3, 133.1, 133.8, 136.1. LRMS *m/z* (rel. int.): 240 (M^+∙^, 97), 225 (21), 199 (16), 198 (76), 197 (70), 183 (82), 181 (16), 179 (18), 166 (17), 165 (43), 155 (43), 153 (27), 152 (24), 141 (52), 129 (100). HRMS [ESI(+)] calcd. for [C_17_H_21_O+H]^+^ 241.1592, found 241.1581.

*Prins cyclization of* **1a** *and* **2u**. The reaction was performed following the general procedure, but using **1a** (0.104 g, 0.556 mmol), **2u** (0.070 mL, 0.556 mmol), CH_2_Cl_2_ (5 mL), I_2_ (0.0071, 0.028 mmol). Compound **7u** (*cis:trans* = 3:1, 0.111 g, 0.368 mmol, 66%) was obtained as colorless viscous oil. The relative configuration was assigned by NMR analysis, including NOESY experiments, of enriched samples of *cis*-**7u** and *trans-***7u** that were obtained after successive purifications of the product by flash column chromatography (1% AcOEt in hexanes).

*(±)-cis-2,4,5,6-Tetrahydro-1-methyl-4-styryl-1H-benzo[f]isochromene* (***cis*-7u**). IR (film): 3070, 3020, 2954, 2917, 2849, 1461, 1375, 1117, 756 cm^−1^. ^1^H-NMR (300 MHz, CDCl_3_) δ: 1.34 (d, *J* = 6.9 Hz, 3H), 2.02–2.26 (m, 2H), 2.68–2.77 (m, 3H), 3.87 (s, 1H), 3.88 (s, 1H), 4.74 (d, *J* = 8.1 Hz, 1H), 6.17 (*J* = 13.9, 8.1 Hz,1H), 6.73 (d, *J* = 15.9 Hz, 1H), 7.10–7.15 (m, 2H), 7.17–7.25 (m, 2H), 7.27–7.30 (m, 2H), 7.31–7.35 (m, 1H), 7.36–7.43 (m, 2H). ^13^C-NMR (75 MHz, CDCl_3_) δ: 18.4, 24.3, 28.1, 28.8, 69.6, 78.6, 122.1, 126.4, 126.57, 126.64, 127.6, 127.8, 128.5, 128.6, 131.7, 132.3, 133.8, 134.1, 135.6, 136.5. LRMS *m/z* (rel. int.): 302 (M^+∙^, 47), 301 (25), 287 (4), 260 (16), 259 (13), 241 (8), 210 (15), 198 (16), 197 (89), 181 (17), 165 (27), 156 (19), 155 (100). HRMS [ESI(+)] calcd. for [C_22_H_22_O+Na]^+^ 325.1568, found 325.1330. 

*(±)-trans-2,4,5,6-Tetrahydro-1-methyl-4-styryl-1H-benzo[f]isochromene* (***trans*-7u**). IR (film): 3058, 3025, 2959, 2926, 2871, 2834, 1490, 1450, 1121, 967, 765, 694 cm^−1^. ^1^H-NMR (500 MHz, CDCl_3_) δ: 1.21 (d, *J* = 7.0 Hz, 3H), 1.98–2.08 (m, 1H), 2.18–2.26 (m, 1H), 2.70–2.84 (m, 3H), 3.63 (dd, *J* = 11.0, 3.5 Hz, 1H), 4.10 (dd, *J* = 11.0, 4.0 Hz, 1H), 4.81 (d, *J* = 7.0 Hz, 1H), 6.25 (dd, *J* = 15.7, 7.0 Hz, 1H), 7.12–7.44 (m, 9H). ^13^C-NMR (50 MHz, CDCl_3_) δ: 17.8, 25.6, 28.1, 28.2, 28.2, 67.1, 77.1, 122.4, 125.2, 126.3, 126.4, 126.5, 127.6, 131.9, 132.3, 133.5, 133.6, 136.1. LRMS *m/z* (rel. int.): 302 (M^+∙^, 44), 301 (23), 260 (17), 259 (11), 210 (14), 207 (15), 197 (67), 179 (15), 165 (29), 155 (100). HRMS [ESI(+)] calcd. for [C_22_H_22_O+Na]^+^ 325.1568, found 325.1355.

*Prins cyclization of* **1b** *and* **2b**. The reaction was performed following the general procedure, but using **1b** (0.104 g, 0.600 mmol), **2b** (0.073 mL, 0.60 mmol), CH_2_Cl_2_ (5 mL), I_2_ (0.030, 0.076 mmol). Compound **13b** was obtained as a pale brown oil (3:1 *cis:trans,* 0.142 g, 0.486 mmol, 81%).

*(±)-1-(4-Methoxyphenyl)-4-methyl-1,3,4,9-tetrahydroindeno[2,1-c]pyran* (**13b**). IR (film): 3020, 2961, 2906, 1512, 1246, 832 cm^−1^. ^1^H-NMR (200 MHz, CDCl_3_) δ: *cis**-*****13b**: 1.48 (d, *J* = 7.0 Hz, 3H), 2.74–2.82 (m, 1H), 2.99 (br, 1H), 3.06–3.95 (br, 1H), 3.80 (s, 3H), 3.95–3.97 (m, 2H); *trans**-*****13b**: 1.34 (d, *J* = 7.0 Hz, 3H), 2.88 (br, 1H), 3.20 (br, 1H), 3.44–3.53 (m, 1H), 3.84–3.88 (m, 2H). Other signals overlap with the major diastereomer. ^13^C-NMR (75 MHz, CDCl_3_) δ: *cis-***13b**: 18.2, 28.7, 37.8, 55.3, 70.4, 78.6, 113.9, 118.5, 123.8, 124.4, 126.3, 129.1, 129.6, 133.2, 140.1, 143.1, 144.1, 159.5.

*trans*-**13b**: 16.4, 28.8, 38.3, 55.2, 67.7, 77.1, 113.8, 119.4, 123.8, 124.4, 126.2, 129.6, 132.8, 140.3, 140.4, 141.0, 143.2, 144.0, 159.5. LRMS *m/z* (rel. int.): 292 (M^+∙^, 3.9), 250 (7.1), 215 (6.0), 202 (14), 141 (19.7), 135 (100). HRMS [ESI(+)] calcd. for [C_20_H_20_O_2_+ Na]^+^ 315.1356, found 315.1355, [C_20_H_20_O_2_+ H]^+^ 293.1536, found 293.1537.

*Prins cyclization of* **1c** *and* **2b**. The reaction was performed following the general procedure, but using **1c** (0.121 g, 0.600 mmol), **2b** (0.073 mL, 0.60 mmol), CH_2_Cl_2_ (5 mL), I_2_ (0.030, 0.076 mmol). Compounds *cis***-14c** (0.110 g, 0.344 mmol, 57%) and *trans***-14c** (0.038 g, 0.118 mmol, 20%) were obtained as colorless oil.

*(±)-(4S,4aR)-4-(4-Methoxyphenyl)-1-methyl-2,4,4a,5,6,7-hexahydrobenzo[3,4]cyclohepta-[1,2-c]pyran* (***cis*-14c**). IR (film): 3061, 3035, 3012, 2927, 1513, 1249, 830, 760, 751 cm^−1^. ^1^H-NMR (200 MHz, CDCl_3_) δ: 1.45 (s, 3H), 1.56–1.75 (m, 1H), 1.82–1.90 (m, 2H), 2.00–2.09 (m, 1H), 2.53–2.58 (m, 1H), 2.69–2.90 (m, 2H), 3.87 (s, 3H), 3.98 (d, *J* = 16.6 Hz, 1H), 4.17 (d, *J* = 16.2 Hz, 1H), 4.67 (d, *J* = 4.0 Hz, 1H), 6.95 (d, *J* = 8.6 Hz, 2H), 7.12–7.15 (m, 1H), 7.22–7.30 (m, 3H), 7.39 (d, *J* = 8.8 Hz, 2H). ^13^C-NMR (50 MHz, CDCl_3_) δ: 15.36, 25.68, 33.59, 34.50, 40.00, 55.18, 66.13, 78.86, 113.49, 125.80, 126.79, 127.41, 128.70, 128.72, 128.82, 132.21, 133.58, 140.85, 141.19, 158.86. LRMS *m/z* (rel. int.): 320 (M^+∙^, 0.08), 263 (1.9), 203 (1.8), 202 9 (3.2), 186 (1.1), 185 (17.3), 184 (100.0). HRMS [ESI(+)] calcd. for [C_22_H_24_O_2_+H]^+^ 321.1849, found 321.1860.

*(±)-(4R,4aR)-4-(4-Methoxyphenyl)-1-methyl-2,4,4a,5,6,7-hexahydrobenzo[3,4]cyclohepta-[1,2-c]-pyran* (***trans*-14c**). IR (film): 3062, 3012, 2926, 1514, 1246, 832, 759 cm^−1^. ^1^H-NMR (200 MHz, CDCl_3_) δ: 1.23–1.30 (m, 1H), 1.44–1.69 (m, 2H), 1.57 (s, 3H), 1.86–1.93 (m, 1H), 2.08 (br, 1H), 2.55–2.79 (m, 2H), 3.80 (s, 3H), 4.40 (s, 2H), 4.827 (d, *J* = 2.6 Hz, 1H), 6.84–6.91 (m, 2H), 7.14–7.24 (m, 6H). ^13^C-NMR (50 MHz, CDCl_3_) δ: 15.1, 27.3, 30.5, 36.0, 43.3, 55.2, 70.7, 78.4, 113.4, 125.6, 126.60, 126.62, 126.66, 128.93, 133.2, 136.3, 141.7, 142.1, 158.2. LRMS *m/z* (rel. int.): 320 (M^+∙^, 0.05), 186 (0.9), 185 (10.3), 184 (100.0). HRMS [ESI(+)] calcd. for [C_22_H_24_O_2_+H]^+^ 321.1849, found 321.1864.

*Prins cyclization of* **1d** *and* **2b**. The reaction was performed following the general procedure, but using **1d** (0.104 g, 0.600 mmol), **2b** (0.073 mL, 0.60 mmol), CH_2_Cl_2_ (5 mL), I_2_ (0.0076 0.030 mmol). Compound **13d** (0.159 g, 0.544 mmol, 91%) was obtained as colorless viscous oil.

*(±)-4-(4-Methoxyphenyl)-1,4,5,6-tetrahydro-2H-benzo[f]isochromene* (**13d**). IR (film): 3061, 3003, 2836, 1713, 1606, 1511, 761, 735 cm^−1^. ^1^H-NMR (200 MHz,CDCl_3_) δ: 1.83–1.91 (m, 2H), 2.41–2.50 (m, 1H), 2.68–2.77 (m, 3H), 3.78 (s, 3H), 3.83–3.92 (m, 1H), 4.04–4.15 (m, 1H), 5.13 (s, 1H), 6.86 (d, *J* = 8.4 Hz, 2H), 7.08–7.19 (m, 2H), 7.23–7.31 (m, 4H). ^13^C-NMR (50 MHz, CDCl_3_) δ: 25.11, 25.15, 27.87, 55.21, 62.24, 78.96, 113.71, 121.74, 126.45, 126.66, 126.84, 127.30, 130.00, 132.01, 133.59, 135.10, 135.14, 159.46. LRMS- *m/z* (rel. int.): 292 (M^+∙^, 92), 264 (16), 263 (31), 233 (16), 135 (100). HRMS [ESI(+)] calcd. for [C_20_H_20_O_2_+H]^+^ 293.1536, found 293.1543.

*Prins cyclization of* **1e** *and* **2b**. The reaction was performed following the general procedure, but using **1e** (0.10 g, 0.49 mmol), **2b** (0.060 mL, 0.49 mmol), CH_2_Cl_2_ (5 mL), I_2_ (0.0063, 0.025 mmol). Compound **13e** (*cis:trans* = 2.3:1, 0.12 g, 0.37 mmol, 76%) was obtained as a colorless viscous oil. This mixture was subjected to another flash column chromatography (1% AcOEt in hexanes). Pure samples were obtained for characterization. 

*(±)-cis-1-Ethyl-2,4,5,6-tetrahydro-4-(4-methoxyphenyl)-1H-benzo[f]isochromene* (***cis*-13e**). IR (film): 3061, 3015, 2959, 2930, 2873, 1606, 1510, 1460, 1249, 1034, 766, 736 cm^−1^. ^1^H-NMR (200 MHz, CDCl_3_) δ: 0.99 (t, *J* = 7.4 Hz, 3H), 1.79–2.04 (m, 2H), 1.57–1.68 (m, 2H), 2.54–2.56 (m, 1H), 2.69–2.81 (m, 2H), 3.70 (dd, *J* = 11.7, 2.8 Hz, 1H), 3.88 (dd, *J* = 11.6, 3.6 Hz, 1H), 5.15 (s, 1H), 6.84–6.90 (m, 2H), 7.13–7.17 (m, 2H), 7.25–7.29 (4H). ^13^C-NMR (50 MHz, CDCl_3_) δ: 11.9, 24.3, 26.2, 28.2, 34.9, 55.2, 62.2, 78.1, 113.7, 122.3, 126.3, 126.5, 127.7, 130.4, 131.4, 131.7, 132.7, 136.3, 159.5. LRMS *m/z* (rel. int.): 320 (M^+∙^, 20), 264 (42), 263 (35), 233 (18), 139 (11), 135 (100). HRMS [ESI(+)] calcd. for [C_22_H_24_O_2_+Na]^+^ 343.1674, found 343.1652. 

*(±)-trans-1-Ethyl-2,4,5,6-tetrahydro-4-(4-methoxyphenyl)-1H-benzo[f]isochromene* (***trans*-13e**). IR (film): 2962, 2934, 2876, 1603, 1511, 1452, 1441, 1255, 1171, 1110, 831, 767 cm^−1^. ^1^H-NMR (200 MHz, CDCl_3_) δ: 1.08 (t, *J* = 7.6 Hz, 3H), 1.76–2.00 (m, 4H), 2.39–2.43 (m, 1H), 2.58–2.74 m, 2H), 3.80 (s, 3H), 3.80–3.88 (m, 1H), 4.10 (dd, *J* = 11.0, 1.6 Hz, 1H), 5.07 (s, 1H), 6.84–6.92 (m, 2H), 7.11–7.15 (m, 2H), 7.22–7.57 (m, 4H). ^13^C-NMR (50 MHz, CDCl_3_) δ: 12.5, 24.5, 24.7, 28.0, 36.0, 55.3, 66.3, 80.3, 113.9, 113.7, 121.9, 126.5, 126.6, 127.7, 129.8, 130.4, 131.3, 131.5, 133.1, 133.4, 133.9, 133.9, 135.8, 159.6. LRMS *m/z* (rel. int.): 320 (M^+∙^, 21), 264 (55), 263 (50), 233 (21), 135 (100). HRMS [ESI(+)] calcd. for [C_22_H_24_O_2_+Na]^+^ 343.1674, found 343.1553.

*Prins cyclization of* **1e** *and* **2k**. The reaction was performed following the general procedure, but using **1e** (0.10 g, 0.51 mmol), **2k** (0.060 mL, 0.51 mmol), CH_2_Cl_2_ (5 mL), I_2_ (0.0065 g, 0.026 mmol). Compound **15e** (0.15 g, 0.41 mmol, 80%) was obtained as a colorless solid. 

*(±)-4-(3-Bromophenyl)-1-ethyl-2,4,5,6-tetrahydro-1H-benzo[f]isochromene* (**15e**). m.p. 125.2 °C. IR (film): 3102, 3053, 2956, 2926, 1488, 1461, 884, 793, 766, 727, 696 cm^−1^. ^1^H-NMR (200 MHz, CDCl_3_) δ: 1.10 (d, *J* = 7.4 Hz, 3H), 1.70–2.07 (m, 4H), 2.40–2.44 (m, 1H), 2.61–2.71 (m, 2H), 3.84 (dd, *J* = 11.1, 2.4 Hz, 1H), 4.12 (dd, *J* = 11.1, 1.6 Hz, 1H), 5.07 (s, 1H), 7.08–7.22 (m, 3H), 7.24–7.27 (m, 2H), 7.33 (dt, *J* = 7.8, 1.4 Hz, 1H), 7.45 (ddd, *J* = 7.6, 2.0, 1.4 Hz, 1H), 7.53 (t, *J* = 1.8 Hz, 1H). ^13^C-NMR (50 MHz, CDCl_3_) δ: 12.5, 24.3, 24.7, 27.9, 36.0, 66.4, 80.2, 122.0, 122.6, 126.5, 126.8, 127.3, 127.7, 130.1, 131.4, 131.7, 131.8, 132.2, 133.5, 135.6, 143.2. LRMS *m/z* (rel. int.): 370 (M^+∙^, 18), 368 (M^+∙^, 18), 314 (46), 312 (49), 233 (37), 215 (24), 185 (48), 183 (44), 157 (25), 155 (28), 129 (100). HRMS [ESI(+)] calcd. for [C_21_H_21_BrO+Na] 391.0673, 393.0653, found 391.0664, 393.0646.

*Prins cyclization of* **1g** *and* **2b**. The reaction was performed following the general procedure, but using **1g** (0.10 g, 0.49 mmol), **2b** (0.060 mL, 0.49 mmol), CH_2_Cl_2_ (5 mL), I_2_ (0.0063, 0.025 mmol). Compound **13g** (*cis:trans* = 12.5:1, 0.83 g, 0.35 mmol, 52%) was obtained as a colorless viscous oil.

*(±)-2,4,5,6-Tetrahydro-4-(4-methoxyphenyl)-1,2-dimethyl-1H-benzo[f]isochromene* (**13g**). IR (film): 3062, 3031, 2971, 2934, 2887, 2834, 1609, 1511, 1488, 1452, 1245, 1033, 832, 767, 732 cm^−1^. ^1^H-NMR (500 MHz, CDCl_3_) δ: *cis*-**13g**: 1.28 (d, *J =* 6.5 Hz, 3H), 1.30 (d, *J =* 6.5 Hz, 3H), 1.75–1.81 (m, 1H), 1.89–1.96 (m, 1H), 2.59–2.70 (m, 3H), 3.79 (s, 3H), 4.01 (qd, *J* = 6.5, 2.5 Hz, 1H), 5.13 (s, 1H), 6.85–6.88 (m, 2H), 7.08 (dd, *J* = 7.2, 1.0 Hz, 2H), 7.13 (td, *J* = 7.2, 1.0 Hz, 2H), 7.22 (dd, *J* = 8.0, 1.0 Hz, 1H), 7.24–7.33 (m, 3H). 

*trans*-**13g**: 1.11 (d, *J* = 4.5 Hz, 3H), 1.12 (d, *J* = 3.5 Hz, 3H), 3.76 (s, 3H), 3.96 (q, *J* = 3.0 Hz, 1H), 5.20 (s, 1H), other signals overlap with the major diastereomer. ^13^C-NMR (50 MHz, CDCl_3_) δ: *cis*-**13g**: 13.6, 18.5, 24.3, 28.1, 32.8, 55.3, 73.0, 81.4, 113.9, 121.8, 126.4, 126.5, 127.7, 129.8, 133.06, 133.12, 133.3, 133.9, 135.7, 159.5. LRMS *m/z* (rel. int.): 320 (M^+∙^, 15), 264 (44), 263 (40), 233 (19), 135 (100). HRMS [ESI(+)] calcd . for [C_22_H_24_O_2_+Na]^+^ 343.1674, found 343.1667.

*Prins cyclization of* **1h** *and* **2b**. The reaction was performed following the general procedure, but using **1h** (0.10 g, 0.70 mmol), **2b** (0.085 mL, 0.70 mmol), CH_2_Cl_2_ (5 mL), I_2_ (0.089, 0.35 mmol). Compound **5h** (0.092 g, 0.23 mmol, 33%) was obtained as a white solid.

*(±)-(2R,4R,6R)-Tetrahydro-4-iodo-2-(4-methoxyphenyl)-6-phenyl-2H-pyran* (**5h**). m.p. 115–117 °C. IR (film): 3065, 3033, 3004, 2921, 2249, 1514, 1249, 909, 733 cm^−1^. ^1^H-NMR (500 MHz, CDCl_3_) δ: 2.22–2.36 (m, 2H), 2.57–2.66 (m, 2H), 3.79 (s, 3H), 4.49–4.59 (m, 3H), 6.86.12 (t, *J* = 2.1 Hz, 1H), 6.89 (t, *J* = 3.0 Hz, 1H), 7.24–7.32 (m, 1H), 7.33–7.41 (m, 6H). ^13^C-NMR (75 MHz, CDCl_3_) δ: 21.7, 29.7, 47.0, 47.1, 55.3, 80.6, 80.9, 113.8, 125.7, 127.1, 127.7, 128.4, 133.4, 141.2, 159.1. LRMS *m/z* (rel. int.): 394 (M^+∙^, 0.1), 267 (5), 161 (15), 160 (19), 159 (19), 144 (15), 137 (43), 136 (44), 135 (81), 131 (56), 130 (51), 129 (72), 127 (86), 115 (60), 107 (16), 106 (22), 105 (35), 92 (18), 91 (50), 78 (19), 77 (100). HRMS [ESI(+)] calcd. for [C_18_H_19_IO_2_+H]^+^ 395.0508, found 395.0500.

*Prins cyclization of* **1i** *and* **2b**. The reaction was performed following the general procedure, but using **1i** (0.0762 g, 0.495 mmol), **2b** (0.0600 mL, 0.495 mmol), CH_2_Cl_2_ (5 mL), I_2_ (0.00626, 0.247 mmol). Compound **5i** (0.0822 g, 0.205 mmol, 41%) was obtained as colorless viscous oil. 

*(±)-(2S,4R,6R)-2-Cyclohexyltetrahydro-4-iodo-6-(4-methoxyphenyl)-2H-pyran* (**5i**). IR (film): 2924, 2851, 1612, 1513, 1248, 1066, 1035, 826, 551 cm^−1^. ^1^H-NMR (300 MHz, CDCl_3_) δ: 1.00–1.15 (m, 3H), 1.15–1.25 (m, 3H), 1.42–1.53 (m, 1H), 1.62–1.80 (m, 5H), 1.84–1.91 (m, 1H), 1.94–2.20 (m, 1H), 2.34–2.57 (m, 1H), 3.22 (ddd, *J* = 11.1, 6.0, 1.8 Hz, 1H), 3.79 (s, 3H), 4.28 (dd, *J* = 11.1, 1.8 Hz, 1H), 4.40 (tt, *J* = 12.3, 4.2 Hz, 1H), 6.84–6.88 (m, 2H), 7.23–7.28 (m, 2H). ^13^C-NMR (75 MHz, CDCl_3_) δ: 23.8, 26.1, 26.2, 26.5, 28.6, 28.9, 42.0, 42.8, 47.4, 55.3, 80.0, 83.3, 113.7, 126.9, 133.9, 159.0. LRMS *m/z* (rel. int.): 400 (M^+∙^, 0.2), 273 (10), 161 (8), 138 (9), 137 (100). HRMS [ESI(+)] calcd. for [C_18_H_25_IO_2_+H]^+^ 401.0977, found 401.1062.

*Prins cyclization of* **1j** *and* **2b**. The reaction was performed following the general procedure, but using **1j** (0.0426 g, 0.495 mmol), **2b** (0.0600 mL, 0.495 mmol), CH_2_Cl_2_ (5 mL), I_2_ (0.00626, 0.247 mmol). Compound **5j** (0.0471 g, 0142 mmol, 29%) was obtained as colorless viscous oil.

*(±)-(2R,4R,6R)-Tetrahydro-4-iodo-2-(4-methoxyphenyl)-6-methyl-2H-pyran* (**5j**). IR (film): 3067, 3036, 2970, 2954, 2835, 1613, 1514, 1250, 1178, 1055, 1036, 827, 774, 548 cm^−1^. ^1^H-NMR (300 MHz, CDCl_3_) δ: 1.25 (d, *J* = 6.0 Hz, 3H), 2.00 (td, *J* = 11.5, 11.0 Hz, 1H), 2.18 (td, *J* = 11.5, 11.0 Hz, 1H), 2.38 (dqt, *J* = 12.5, 2.0 Hz, 1H), 2.49 (td, *J* = 12.5, 2.0 Hz, 1H), 3.59–3.66 (m, 1H), 4.31 (dd, *J* = 11.0, 2.0 Hz, 1H), 4.40 (tt, *J* = 12.5, 4.5 Hz, 1H), 6.85–6.86 (m, 1H), 6.87–6.88 (m, 1H), 7.24–7.25 (m, 1H), 7.26–7.27 (m, 1H). ^13^C-NMR (50 MHz, CDCl_3_) δ: 21.5, 22.3, 46.6, 46.8, 55.3, 75.2, 80.4, 113.8, 125.3, 127.2, 133.5. LRMS *m/z* (rel. int.): 332 (M^+∙^, 0.4), 206 (6), 205 (46), 161 (8), 146 (3), 137 (100). HRMS [ESI(+)] calcd. for [C_13_H_17_IO_2_+Na]^+^ 355.0171, found 355.0164.

*Prins cyclization of* **1k** *and* **2b**. The reaction was performed following the general procedure, but using **1k** (0.0496 g, 0.495 mmol), **2h** (0.0600 mL, 0.495 mmol), CH_2_Cl_2_ (5 mL), I_2_ (0.00626, 0.247 mmol). Compound **5k** (0.0642 g, 0185 mmol, 37%) was obtained as colorless viscous oil.

*(±)-(2R,3S,4S)-3-Ethyltetrahydro-4-iodo-2-(4-methoxyphenyl)-2H-pyran* (**5k**). IR (film): 2958, 2933, 2872, 2848, 1516, 1444, 1257, 1088, 1029, 826, 814, 545 cm^−1^. ^1^H-NMR (300 MHz, CDCl_3_) δ: 0.66 (t, *J* = 7.8 Hz, 3H), 1.36–1.30 (m, 1H), 1.48–1.62 (m, 2H), 2.02–2.12 (m, 1H), 2.45–2.53 (m, 1H), 2.57–2.71 (m, 1H), 3.51 (td, *J* = 11.8, 2.1 Hz, 1H), 3.80 (s, 3H), 3.81–3.85 (m, 1H), 4.12 (d, *J* = 9.9 Hz, 1H), 4.35 (td, *J* = 11.7, 4.5), 6.85–6.90 (m, 2H), 7.23–7.28 (m, 2H). ^13^C-NMR (75 MHz, CDCl_3_) δ: 8.3, 25.0, 33.2, 41.5, 51.5, 55.3, 69.7, 83.2, 113.8, 128.3, 132.7, 159.5. LRMS *m/z* (rel. int.): 346 (M^+^, 0.05), 220 (3), 219 (20), 137 (52), 135 (15), 83 (38), 77 (11), 67 (85), 55 (100). HRMS [ESI(+)] calcd. for [C_14_H_19_IO_2_+Na]^+^ 369.0327, found 369. 0333.

*Prins cyclization of* **1l** *and* **2b** *with 0.5 equiv of Iodine*. The reaction was performed following the general procedure, but using **1l** (0.0496 g, 0.495 mmol), **2b** (0.0600 mL, 0.495 mmol), CH_2_Cl_2_ (5 mL), I_2_ (0.0626, 0.247 mmol). Compound **5l** [30] (0.0689 g, 0.199 mmol, 40%) was obtained as colorless viscous oil.

*Prins cyclization of **1l** and* **2b** *with 1 equiv of Iodine*. The reaction was performed following the general procedure, but using **1l** (0.0496 g, 0.495 mmol), **2b** (0.0600 mL, 0.496 mmol), CH_2_Cl_2_ (5 mL), I_2_ (0.1252, 0.495 mmol). Compound **5l** [30] (0.139 g, 0.402 mmol, 81%) was obtained as colorless viscous oil.

*Prins cyclization of* **1l** *and* **2b** *with 1 equiv of Iodine* *and 2 equiv of* **2b**. The reaction was performed following the general procedure, but using **1l** (0.0992 g, 0.990 mmol), **2b** (0.0600 mL, 0.495 mmol), CH_2_Cl_2_ (5 mL), I_2_ (0.125, 0.495 mmol). Compound **5l**[30] (0.144 g, 0.417 mmol, 84%) was obtained as colorless viscous oil.

### 3.3. Computational Details

Gaussian09 revision A.01 was used for all calculations [47]. All structures were optimized at the B3LYP/6–31+G(d,p) theoretical levels [48]. Stationary points were characterized as minima by vibrational analysis. All reported energies include zero-point energy (ZPE) as well as thermal corrections (T = 298.15 K) from frequency calculations. Relaxed potential energy surface scan was carried out at the B3LYP/6–31G(d) level. Natural-bonding orbitals (NBO) analysis [49] was carried out by NBO 3.1 as implemented in the Gaussian09 suite of programs.

### 3.4. X-ray Crystallography

Well-shaped single crystals of **7k** and **15e** were chosen for the X-ray experiments. The single crystal X-ray diffraction measurements were performed at 150K on a Gemini A-Ultra diffractometer equipped with an Atlas CCD detector using mirror monochromatized CuKα radiation (λ = 1.5418 Å). The programs CrysAlis CCD and CrysAlis RED [50] were used for data collection, cell refinement and data reduction. The structures were solved by direct methods using the software Sir92 [51] and refined by full-matrix least squares on F² using the software SHELXL-2013 [52]. All non-hydrogen atoms were clearly identified and refined with least square of complete matrix in F² with anisotropic parameters considered. H atoms on C atoms were positioned stereochemically and were refined with fixed individual displacement parameters [Uiso(H) = 1.5Ueq(C) for methyl groups or 1.2Ueq(C) for aromatic, methine and methylene groups], using the SHELXL riding model with C-H bond lengths of 0.95, 0.96, 1.00 and 0.99 Å for aromatic, methyl, methine and methylene groups, respectively. WINGX software [53] was used to analyze and prepare the data for publication. Molecular graphics were prepared using ORTEP-3 for Windows [54] and Mercury [55]. Crystal data, data collection procedures, structure determination methods and refinement results are summarized in Table S1. Crystallographic data for the structural analysis of the compounds discussed here have been deposited at the Cambridge Crystallographic Data Centre, 12 Union Road, Cambridge CB2 1EZ, UK, and are available on request quoting the deposition numbers CCDC 948196 and 948057, for **7k** and **15e**, respectively.

## 4. Conclusions

The Prins cyclization of homoallylic alcohols and aldehydes can be performed using catalytic amounts of iodine as Lewis acid. Anhydrous conditions and inert atmosphere are not required in this metal-free protocol. The desired *O*-heterocycles were obtained in 52%–91% yield for 30 examples, including different homoallylic alcohols and several aliphatic and aromatic aldehydes. The main limitation is the use of some acyclic homoallylic alcohols, where the use of stoichiometric iodine is required to obtain good yield of the product. The mechanism and the ratio of the products could be explained by DFT calculations.

## Supplementary Materials

Additional information regarding X-ray analysis, DFT calculations, and NMR analysis are available at http://www.mdpi.com/1420-3049/18/9/11100/s1.
