# Supplementary material for: Iodine-Catalyzed Prins Cyclization of Homoallylic Alcohols and Aldehydes
Source: Molecules. 2013 Sep 10;18(9):11100–30. doi: 10.3390/molecules180911100 (PMC6269844; doi:10.3390/molecules180911100)

Supplementary Materials

Contents

Part 1: X-ray Crystallographic Data Collection S2

Part 2: Cartesian Coordinates for Optimized Geometries and Themochemical data S3

1. Themochemical Data S3
2. Energy Surface Scan S4
3. Cartesian Coordinates for Optimized geometries S4

Part 3: Selected-NMR Spectra S24

Part 1: X-ray Crystallographic Data Collection

Table S1. Crystallographic data for **7k** and **15e**.

**Compound**	**7k**	**15e**
Empirical formula	$C_{20}H_{19}BrO$	$C_{21}H_{21}BrO$
Formula weight	355.26	369.29
Crystal system	Orthorhombic	Monoclinic
Space group	$P2_12_12_1$	$P2_1/c$
Unit Cell Dimensions, (Å, °)	*a* = 7.2662(1)<br>*b* = 12.8517(2)<br>*c* = 16.8476(3)	*a* = 14.0033(3)<br>*b* = 7.7072(2)<br>*c* = 15.9479(3)<br>β = 105.606(2)
V, Å^3	1573.28(4)	1657.74(6)
Z	4	4
ρ calc., Mg m^{-3}	1.500	1.480
μ, mm^{-1}	3.538	3.379
F(000)	728	760
θ-max, °	65.79	66.481
Index ranges	−8 ≤ h ≤ 8,<br>−14 ≤ k ≤ 15,<br>−19 ≤ l ≤ 19	−16 ≤ h ≤ 16,<br>−8 ≤ k ≤ 8,<br>−18 ≤ l ≤ 18
Reflections collected	25884	61561
Independent reflections	2620 [R(int) = 0.0429]	2893 [R(int) = 0.0670]
Completeness to θ-max, %	97.1	96.5
Refinement method	Full-matrix least-squares on F^2	Full-matrix least-squares on F^2
Data/restraints/ parameters	2620 / 0 / 200	2893 / 0 / 209
Goodness-of-fit on F^2	1.114	1.080
Final R indices [I>2sigma(I)]	R1 = 0.0216, wR2 = 0.0510	R1 = 0.0297, wR2 = 0.0635
R indices (all data)	R1 = 0.0240, wR2 = 0.0525	R1 = 0.0406, wR2 = 0.0689
Absolute structure parameter	−0.041(8)	
Largest diff. peak and hole, e.Å^{-3}	0.224 and -0.355	0.436 and −0.283

Part 2: Cartesian Coordinates for Optimized Geometries and Themochemical data

1. Themochemical Data

Table S2. Free energy (G + ZPE + thermal corrections at 298.15 K) in Hartrees for all compounds studied determined at the DFT B3LYP/6–31+G(d,p) level, changes in free energy (ΔG^0) for different processes, activation energy ($\Delta G^‡$) and relative energies ($\Delta G^{0,rel}$).[a]

REAGENTS	G^0 (Hartrees)	G^0 (Hartrees)	$\Delta G^{0,E/Z}$ (kJ mol^{-1})	$\Delta G^{0,rel}$ (kJ mol^{-1})
(*E*)–3h, A	–849.49142			0
(*Z*)–3h, A	–849.47487		43	43
(*E*)–3h, B_2	–849.49210			
(*Z*)–3h, B_2	–849.47645		41	
(*E*)–3h, B	–849.48584			15
(*Z*)–3h, B	–849.47245			50
TS			$\Delta G^‡$ (kJ mol^{-1})	
(*E*)–3h, A → *cis*–4h, A	–849.48408		19	
(*Z*)–3h, A → *trans*–4h, A	–849.46937		14	
INTERMEDIATES			$\Delta G^{0,R–I}$ (kJ mol^{-1})	$\Delta G^{0,rel}$ (kJ mol^{-1})
cis–4h, A	–849.49182		–1	0
trans–4h, A	–849.48516		–27	17
cis–4h, B	–849.47772		21	37
trans–4h, B	–849.47804		–15	36
PRODUCTS	7h	12h	$\Delta G^{0,7h–12h}$ (kJ mol^{-1})	
cis, A	–849.14799	–849.138025	–26	
trans, A	–849.14685	–849.132352	–38	
cis, B	–849.14691	–849.140602	–17	
trans, B	–849.14513	–849.137892	–19	
	7s	12s	$\Delta G^{0,7s–12s}$ (kJ mol^{-1})	*K*, Boltzmann
	–775.311037	–775.310585	–1.2	1.6

[a] All compounds have no imaginary frequencies, except those corresponding to transition states (TS), which have one negative (imaginary) frequency corresponding to the reaction coordinate.

2. Energy Surface Scan

Figure S1. Change in energy resulting from the rotation around the dihedral angle formed between the 1,2-dihydronaphtalene ring and the benzylidene(propyl)oxonium moiety (Me–CH–(C=C), φ_1) of compound (*E*)-3h, A, determined by a relaxed potential energy surface scan at the B3LYP/6-31G(d) level.

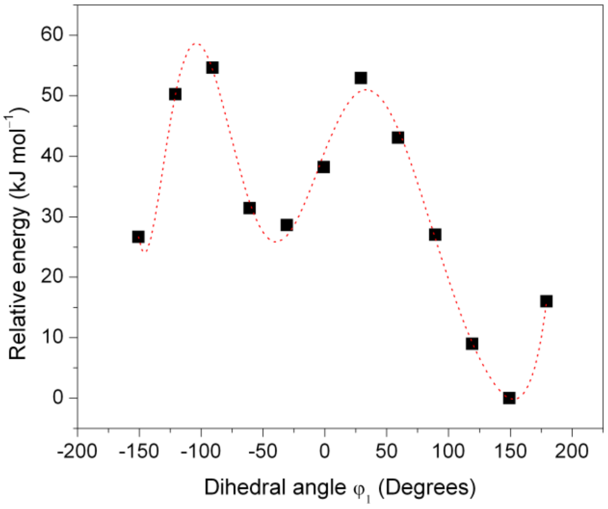

3. Cartesian Coordinates for Optimized geometries

Reagents

(*E*)–3h, A
symmetry c1

C	-4.283592000	-1.508947000	-1.143037000
C	-3.568098000	-2.001736000	-0.048264000
C	-2.556013000	-1.250802000	0.552221000
C	-2.242845000	0.036178000	0.040823000
C	-2.987242000	0.524962000	-1.051061000
C	-3.994433000	-0.238057000	-1.641832000
H	-5.067248000	-2.109336000	-1.594399000
H	-3.807682000	-2.982805000	0.353133000
H	-2.798696000	1.515540000	-1.451139000
H	-4.552556000	0.162267000	-2.482171000
C	1.289091000	0.562286000	-0.887981000
C	-0.960575000	2.283842000	0.338441000
H	-1.940813000	2.703454000	0.083442000
C	-0.132985000	2.446623000	-0.959966000
H	0.071639000	3.494712000	-1.178166000
H	-0.606374000	1.970045000	-1.821832000
O	1.191226000	1.848323000	-0.793883000
C	2.512108000	-0.108818000	-0.609865000
C	2.579464000	-1.498251000	-0.880967000
C	3.646619000	0.571119000	-0.098551000
C	3.758913000	-2.192922000	-0.643569000
H	1.711839000	-2.013376000	-1.283657000
C	4.817030000	-0.133900000	0.132205000

H	3.591088000	1.635431000	0.102892000
C	4.873350000	-1.512106000	-0.137836000
H	3.817589000	-3.255525000	-0.853377000
H	5.691757000	0.377384000	0.519989000
H	5.795352000	-2.055417000	0.045629000
C	-0.388980000	-1.285918000	1.845627000
C	-1.849296000	-1.747506000	1.794096000
C	-1.147206000	0.809369000	0.674158000
H	-1.911476000	-2.837691000	1.859918000
H	0.222022000	-1.880272000	1.144999000
H	0.036241000	-1.469634000	2.837383000
H	-2.376103000	-1.351560000	2.674773000
C	-0.269032000	0.172569000	1.497360000
H	0.534997000	0.733602000	1.964007000
C	-0.376927000	3.145681000	1.472065000
H	-0.400747000	4.202389000	1.188239000
H	-0.966654000	3.028647000	2.384437000
H	0.660760000	2.891276000	1.704540000
H	0.449160000	0.012281000	-1.311983000

(*Z*)–3h, A
symmetry c1

C	0.934339000	3.572147000	-0.947600000
C	0.918136000	3.220170000	0.404813000
C	1.110214000	1.898501000	0.811015000
C	1.320754000	0.889078000	-0.165542000
C	1.350964000	1.265837000	-1.523797000
C	1.157008000	2.590016000	-1.914535000
H	0.788305000	4.606956000	-1.241481000
H	0.766758000	3.988302000	1.158489000
H	1.541586000	0.529828000	-2.297818000
H	1.190596000	2.853628000	-2.967012000
C	-0.944806000	-2.257988000	0.174932000
C	2.023247000	-1.556282000	-0.698240000
H	2.627631000	-1.051815000	-1.461283000
C	0.841210000	-2.159736000	-1.503709000
H	1.179746000	-2.958436000	-2.164105000
H	0.305132000	-1.406839000	-2.074201000
O	-0.051934000	-2.831286000	-0.568644000
C	-1.840113000	-1.167161000	-0.052558000
C	-2.691919000	-0.869940000	1.047853000
C	-2.006766000	-0.437773000	-1.259862000
C	-3.649604000	0.130460000	0.955770000
H	-2.597552000	-1.442965000	1.965828000
C	-2.981160000	0.545147000	-1.346176000
H	-1.416272000	-0.661110000	-2.137226000
C	-3.794523000	0.839765000	-0.241770000
H	-4.291799000	0.347823000	1.802551000
H	-3.116028000	1.085660000	-2.276974000
H	-4.551094000	1.614112000	-0.322806000
C	0.640435000	0.135194000	2.547892000
C	1.189582000	1.539554000	2.277716000

C	1.517760000	-0.508699000	0.281268000
H	0.661520000	2.282158000	2.883047000
H	-0.460907000	0.152637000	2.478369000
H	0.876523000	-0.181967000	3.568385000
H	2.244092000	1.576100000	2.588509000
C	1.182833000	-0.856612000	1.556112000
H	1.358441000	-1.871133000	1.901939000
C	2.897701000	-2.665055000	-0.087269000
H	3.308851000	-3.296482000	-0.880896000
H	3.735390000	-2.229553000	0.462851000
H	2.343836000	-3.315256000	0.594643000
H	-1.131027000	-2.851099000	1.070461000

(*E*)–3h, B_2
symmetry c1

C	-4.237654000	-1.607939000	-1.140190000
C	-3.292698000	-2.156218000	-0.269218000
C	-2.322576000	-1.358622000	0.341547000
C	-2.290940000	0.035484000	0.076468000
C	-3.254873000	0.571679000	-0.799681000
C	-4.218036000	-0.237762000	-1.402748000
H	-4.987926000	-2.243981000	-1.599306000
H	-3.313894000	-3.221430000	-0.054066000
H	-3.287009000	1.637150000	-1.001632000
H	-4.955440000	0.204696000	-2.065027000
C	1.269729000	0.417519000	-0.857044000
C	-0.994802000	2.283527000	0.241727000
H	-1.955909000	2.746545000	-0.014449000
C	-0.253013000	2.229780000	-1.108752000
H	-0.080267000	3.224298000	-1.519654000
H	-0.765274000	1.600087000	-1.840576000
O	1.102132000	1.692060000	-0.921695000
C	2.546165000	-0.143428000	-0.580574000
C	2.679415000	-1.553158000	-0.644786000
C	3.672196000	0.664344000	-0.277375000
C	3.914346000	-2.142089000	-0.410617000
H	1.814922000	-2.167291000	-0.880776000
C	4.898767000	0.063450000	-0.044435000
H	3.565187000	1.742750000	-0.234363000
C	5.019862000	-1.335049000	-0.110089000
H	4.023764000	-3.219994000	-0.461549000
H	5.766933000	0.671446000	0.186715000
H	5.985376000	-1.796721000	0.073387000
C	-1.284197000	-1.966312000	1.260510000
C	-0.873513000	-0.998547000	2.374488000
C	-0.597696000	0.372881000	1.823228000
C	-1.266498000	0.874735000	0.755114000
H	-1.679055000	-0.919224000	3.121791000
H	0.000619000	-1.378423000	2.913224000
H	-0.389822000	-2.228330000	0.672092000
H	-1.654508000	-2.905927000	1.680957000
H	0.106528000	0.997832000	2.363671000

C	-0.292889000	3.233719000	1.225020000
H	-0.226771000	4.237211000	0.793571000
H	-0.858697000	3.307198000	2.156984000
H	0.723573000	2.912918000	1.470129000
H	0.424052000	-0.225258000	-1.103132000

(*Z*)–3h, B_2
symmetry c1

C	1.385325000	3.382441000	-0.922980000
C	1.170288000	2.985104000	0.399962000
C	1.280230000	1.645890000	0.782284000
C	1.619485000	0.664644000	-0.185032000
C	1.842141000	1.083130000	-1.511221000
C	1.725138000	2.425554000	-1.880222000
H	1.306290000	4.429907000	-1.197394000
H	0.921962000	3.729992000	1.151788000
H	2.143591000	0.371035000	-2.272932000
H	1.917852000	2.720553000	-2.907165000
C	-1.588236000	-2.123833000	0.024117000
C	1.768410000	-1.853497000	-0.811756000
H	2.412336000	-1.519899000	-1.636743000
C	0.399193000	-2.018097000	-1.496642000
H	0.448916000	-2.770768000	-2.285630000
H	-0.005981000	-1.091553000	-1.891701000
O	-0.557079000	-2.620536000	-0.546375000
C	-2.271291000	-0.873095000	-0.049684000
C	-3.526167000	-0.883047000	0.627705000
C	-1.859331000	0.316486000	-0.712955000
C	-4.350463000	0.231473000	0.612326000
H	-3.845591000	-1.779994000	1.150348000
C	-2.690306000	1.425783000	-0.711961000
H	-0.890218000	0.400011000	-1.183331000
C	-3.933661000	1.385626000	-0.061638000
H	-5.307688000	0.208432000	1.121736000
H	-2.367251000	2.333510000	-1.209837000
H	-4.573482000	2.262902000	-0.073332000
C	1.016692000	1.214350000	2.208438000
C	1.915917000	0.042655000	2.613758000
C	1.894829000	-1.033167000	1.560825000
C	1.750709000	-0.754983000	0.246686000
H	2.952697000	0.388527000	2.749583000
H	1.602703000	-0.363684000	3.580762000
H	-0.035112000	0.898724000	2.298143000
H	1.146412000	2.059427000	2.891183000
H	2.058489000	-2.055492000	1.887286000
C	2.319923000	-3.213526000	-0.354117000
H	2.370029000	-3.904582000	-1.201412000
H	3.331063000	-3.098251000	0.042910000
H	1.701704000	-3.679917000	0.417518000
H	-2.045047000	-2.873932000	0.671856000

(*E*)–3h, B

symmetry c1

C	2.741286000	-2.588619000	-1.363417000
C	2.404522000	-2.666718000	-0.009231000
C	1.928764000	-1.552880000	0.684210000
C	1.781931000	-0.312716000	0.005846000
C	2.132119000	-0.253172000	-1.357353000
C	2.604401000	-1.375168000	-2.038170000
H	3.112768000	-3.466476000	-1.882875000
H	2.519246000	-3.608937000	0.519967000
H	2.046075000	0.674241000	-1.909009000
H	2.869189000	-1.297411000	-3.087984000
C	-1.453158000	1.526500000	-0.191104000
C	1.375412000	2.287358000	0.269538000
H	0.968940000	2.903742000	1.079502000
C	0.518550000	2.602883000	-0.990425000
H	1.064794000	2.488021000	-1.924289000
H	0.108844000	3.615963000	-0.944964000
O	-0.609922000	1.677098000	-1.154233000
C	-2.463798000	0.531760000	-0.250102000
C	-3.439678000	0.509141000	0.778731000
C	-2.521272000	-0.412722000	-1.307565000
C	-4.457641000	-0.433360000	0.743959000
H	-3.390396000	1.233262000	1.587300000
C	-3.542185000	-1.349676000	-1.327804000
H	-1.767829000	-0.392383000	-2.087183000
C	-4.507888000	-1.359242000	-0.307470000
H	-5.210783000	-0.452846000	1.524259000
H	-3.597094000	-2.075984000	-2.131695000
H	-5.305378000	-2.095702000	-0.333693000
C	1.628464000	-1.631565000	2.164490000
C	0.452462000	-0.727349000	2.548370000
C	0.622149000	0.638462000	1.943976000
C	1.259149000	0.848828000	0.761737000
H	-0.493902000	-1.167629000	2.192326000
H	0.363520000	-0.651901000	3.636636000
H	2.519747000	-1.309640000	2.723255000
H	1.432534000	-2.666693000	2.458967000
H	0.241229000	1.491993000	2.502923000
C	2.817346000	2.782215000	0.030449000
H	2.818464000	3.835180000	-0.271533000
H	3.393472000	2.701416000	0.955482000
H	3.331780000	2.203784000	-0.739855000
H	-1.431470000	2.237464000	0.634323000

(*Z*)–3h, B

symmetry c1

C	-4.952870000	-0.164819000	-0.957945000
C	-4.474611000	1.000269000	-0.353209000
C	-3.182073000	1.067765000	0.170023000
C	-2.329845000	-0.065672000	0.090665000
C	-2.832163000	-1.230605000	-0.518547000
C	-4.125286000	-1.284445000	-1.039406000

H	-5.960991000	-0.196139000	-1.359274000
H	-5.118088000	1.873253000	-0.283182000
H	-2.220770000	-2.120385000	-0.600227000
H	-4.481141000	-2.198130000	-1.505152000
C	1.730217000	-0.286787000	-1.481544000
C	-0.090351000	-1.159186000	1.000502000
H	0.788256000	-0.741310000	1.503456000
C	0.435072000	-1.978600000	-0.202137000
H	-0.317709000	-2.636581000	-0.628776000
H	1.310894000	-2.576749000	0.056710000
O	0.769050000	-1.121145000	-1.350194000
C	2.932157000	-0.051465000	-0.749036000
C	3.746118000	0.981752000	-1.293584000
C	3.392808000	-0.754930000	0.397959000
C	4.964949000	1.304397000	-0.714290000
H	3.407999000	1.520301000	-2.174069000
C	4.616330000	-0.430734000	0.960700000
H	2.808329000	-1.546873000	0.843614000
C	5.400744000	0.597486000	0.411911000
H	5.576219000	2.094880000	-1.135960000
H	4.968633000	-0.973081000	1.831632000
H	6.355502000	0.843559000	0.866645000
C	-2.693616000	2.315788000	0.871445000
C	-1.200376000	2.546635000	0.620763000
C	-0.432801000	1.276255000	0.872815000
C	-0.954936000	0.047787000	0.644221000
H	-1.040538000	2.876350000	-0.418499000
H	-0.823226000	3.351211000	1.260014000
H	-2.857346000	2.201441000	1.953664000
H	-3.279213000	3.184188000	0.555592000
H	0.574481000	1.362956000	1.277223000
C	-0.738739000	-2.146990000	1.996087000
H	-0.042175000	-2.948767000	2.265895000
H	-1.005586000	-1.614703000	2.912374000
H	-1.647491000	-2.602150000	1.597155000
H	1.587192000	0.312489000	-2.381645000

TS

(*E*)–3h, A →*cis*–4h, A

symmetry c1

C	-2.033770000	-3.266068000	-0.666258000
C	-1.748057000	-2.860648000	0.639761000
C	-1.486235000	-1.522708000	0.936128000
C	-1.519054000	-0.558978000	-0.112617000
C	-1.828648000	-0.987324000	-1.425533000
C	-2.073429000	-2.326008000	-1.703013000
H	-2.233838000	-4.312643000	-0.874631000
H	-1.735130000	-3.594786000	1.440181000
H	-1.870709000	-0.273887000	-2.241600000
H	-2.302369000	-2.638159000	-2.716645000
C	1.194630000	1.782956000	0.316088000
C	-1.619120000	1.955513000	-0.734366000

H	-2.284303000	1.574260000	-1.512881000
C	-0.313187000	2.398684000	-1.472243000
H	-0.484099000	3.329468000	-2.014712000
H	0.036306000	1.637985000	-2.171192000
O	0.682554000	2.712960000	-0.497591000
C	1.963249000	0.593207000	-0.054004000
C	2.972417000	0.227834000	0.867997000
C	1.819211000	-0.175963000	-1.230054000
C	3.818124000	-0.849540000	0.618269000
H	3.111377000	0.815412000	1.771315000
C	2.673595000	-1.245640000	-1.478663000
H	1.052143000	0.047352000	-1.957520000
C	3.671705000	-1.587775000	-0.558659000
H	4.594481000	-1.103547000	1.332489000
H	2.559591000	-1.818759000	-2.393058000
H	4.332349000	-2.424706000	-0.761922000
C	-0.249327000	0.090757000	2.415309000
C	-1.254195000	-1.066215000	2.357289000
C	-1.268532000	0.838941000	0.217967000
H	-0.905563000	-1.901174000	2.971675000
H	0.757641000	-0.304562000	2.226411000
H	-0.230584000	0.531226000	3.416203000
H	-2.215522000	-0.746553000	2.784616000
C	-0.588429000	1.156725000	1.394372000
H	-0.660546000	2.177970000	1.754373000
C	-2.311441000	3.167774000	-0.078290000
H	-2.652039000	3.857597000	-0.855915000
H	-3.186386000	2.852734000	0.496277000
H	-1.642606000	3.721943000	0.584586000
H	1.582331000	2.275995000	1.205796000

(*Z*)–3h, A →*trans*–4h, A
symmetry c1

C	-4.467599000	-1.562995000	-0.716525000
C	-3.535860000	-2.065653000	0.195960000
C	-2.428155000	-1.310088000	0.579122000
C	-2.259331000	-0.001193000	0.039497000
C	-3.222026000	0.495367000	-0.872156000
C	-4.307580000	-0.281077000	-1.256251000
H	-5.320674000	-2.169658000	-1.004582000
H	-3.674912000	-3.057841000	0.615485000
H	-3.116909000	1.487085000	-1.298197000
H	-5.030155000	0.108005000	-1.965858000
C	1.144294000	0.537699000	-0.749143000
C	2.671692000	-1.381132000	-1.035874000
H	-2.027393000	2.649920000	-0.027911000
C	-0.129280000	2.493269000	-0.999759000
H	0.075626000	3.554646000	-1.144792000
H	-0.590253000	2.079320000	-1.903907000
O	1.141100000	1.875234000	-0.785145000
C	2.434568000	-0.099546000	-0.502069000
C	0.020721000	0.131152000	0.975005000

C	3.448190000	0.550904000	0.230531000
C	3.904484000	-2.002028000	-0.842346000
H	1.903099000	-1.876766000	-1.622528000
C	4.675746000	-0.074330000	0.418221000
H	3.272382000	1.543542000	0.632054000
C	4.904435000	-1.351277000	-0.113949000
H	4.088205000	-2.984462000	-1.264792000
H	5.459073000	0.429434000	0.975243000
H	5.865015000	-1.834196000	0.036126000
C	-0.015597000	-1.363505000	1.240393000
C	-1.435313000	-1.818981000	1.596782000
C	-1.115220000	0.784621000	0.479098000
H	0.719324000	0.716262000	1.563527000
H	-1.713050000	-1.438970000	2.590393000
H	0.686184000	-1.614791000	2.039682000
H	0.320565000	-1.914431000	0.352448000
C	-1.040600000	2.271572000	0.249659000
H	-1.480299000	-2.910039000	1.658115000
C	-0.542482000	3.091764000	1.455547000
H	-0.635099000	4.159195000	1.234764000
H	-1.142871000	2.880334000	2.344399000
H	0.505234000	2.891764000	1.691968000
H	0.477596000	0.032523000	-1.450934000

INTERMEDIATES

cis–4h, A

symmetry c1

C	-4.792629000	-1.449483000	-0.276856000
C	-3.671000000	-2.073346000	0.274457000
C	-2.459527000	-1.395919000	0.389585000
C	-2.382033000	-0.025619000	-0.047024000
C	-3.554187000	0.590289000	-0.591005000
C	-4.733023000	-0.115337000	-0.718200000
H	-5.722539000	-2.003459000	-0.366598000
H	-3.740732000	-3.101837000	0.615175000
H	-3.518471000	1.613578000	-0.943269000
H	-5.608298000	0.354697000	-1.153248000
C	1.216661000	0.529938000	-0.645258000
C	-1.079269000	2.179146000	0.064765000
H	-2.008832000	2.605186000	-0.316572000
C	0.065222000	2.563183000	-0.924648000
H	0.234804000	3.641367000	-0.879805000
H	-0.217748000	2.292910000	-1.952481000
O	1.274368000	1.938843000	-0.559365000
C	2.575684000	-0.055703000	-0.362875000
C	3.135041000	-0.984618000	-1.249687000
C	3.295808000	0.325202000	0.779520000
C	4.395845000	-1.532284000	-0.997783000
H	2.593096000	-1.274096000	-2.146542000
C	4.556518000	-0.216470000	1.027358000
H	2.884938000	1.065145000	1.460577000
C	5.106884000	-1.149999000	0.141266000

H	4.822671000	-2.247309000	-1.694070000
H	5.113323000	0.092475000	1.906582000
H	6.088063000	-1.571139000	0.337158000
C	0.024559000	-1.549887000	0.348319000
C	-1.256452000	-2.047105000	1.013387000
C	-1.167911000	0.689950000	0.137948000
H	-1.339591000	-3.135128000	0.937943000
H	0.039977000	-1.886109000	-0.696392000
H	0.905073000	-1.979536000	0.830511000
H	-1.243089000	-1.814679000	2.089381000
C	0.114532000	-0.019271000	0.391332000
H	0.494989000	0.316291000	1.366608000
C	-0.820532000	2.801139000	1.465636000
H	-0.892446000	3.888911000	1.379974000
H	-1.564284000	2.469221000	2.195254000
H	0.176677000	2.553076000	1.833671000
H	0.886334000	0.226854000	-1.650358000

trans–4h, A
symmetry c1

C	-4.191931000	-1.684774000	-0.620147000
C	-3.520184000	-1.721713000	0.603542000
C	-2.397307000	-0.928395000	0.830189000
C	-1.947913000	-0.044004000	-0.213936000
C	-2.667205000	-0.018763000	-1.450916000
C	-3.761679000	-0.833824000	-1.654034000
H	-5.055716000	-2.324599000	-0.775597000
H	-3.872191000	-2.382000000	1.390280000
H	-2.338210000	0.623175000	-2.258507000
H	-4.286133000	-0.820012000	-2.603281000
C	1.538801000	0.948742000	0.939129000
C	-0.551912000	2.018863000	-0.816362000
H	-0.989027000	1.901993000	-1.810656000
C	0.975806000	2.244384000	-0.978243000
H	1.153351000	3.240073000	-1.390157000
H	1.398090000	1.502713000	-1.667485000
O	1.627772000	2.208563000	0.274428000
C	2.320535000	-0.153354000	0.243149000
C	3.672007000	-0.310717000	0.588890000
C	1.787080000	-0.974141000	-0.761436000
C	4.471679000	-1.250499000	-0.062770000
H	4.103532000	0.315440000	1.365283000
C	2.584413000	-1.914775000	-1.418520000
H	0.737046000	-0.904137000	-1.035944000
C	3.929722000	-2.053982000	-1.069658000
H	5.515410000	-1.355713000	0.216256000
H	2.153768000	-2.541292000	-2.193517000
H	4.549112000	-2.789140000	-1.573820000
C	-0.205510000	-0.671356000	1.996567000
C	-1.698892000	-0.940779000	2.162856000
C	-0.844118000	0.820622000	0.030009000
H	-1.873421000	-1.896640000	2.665117000

H	0.261298000	-1.501386000	1.457443000
H	0.284340000	-0.610296000	2.973019000
H	-2.155061000	-0.170185000	2.804501000
C	0.018507000	0.638856000	1.233966000
H	-0.265847000	1.480419000	1.891705000
C	-1.217138000	3.273838000	-0.164967000
H	-1.121239000	4.110038000	-0.862741000
H	-2.280381000	3.113457000	0.032239000
H	-0.713177000	3.542974000	0.765638000
H	2.003717000	1.131256000	1.910958000

cis–4h, B
symmetry c1

C	-4.752716000	-1.560628000	0.061510000
C	-3.520052000	-2.188765000	0.248667000
C	-2.325729000	-1.481152000	0.121869000
C	-2.383942000	-0.079517000	-0.195006000
C	-3.662333000	0.543064000	-0.353323000
C	-4.826051000	-0.189361000	-0.244543000
H	-5.667283000	-2.137554000	0.162897000
H	-3.489079000	-3.241992000	0.511530000
H	-3.722839000	1.593275000	-0.612178000
H	-5.789253000	0.285670000	-0.395674000
C	1.377603000	0.773470000	-0.760260000
C	-1.155796000	2.149299000	-0.147456000
H	-1.991997000	2.600162000	-0.695155000
C	0.168990000	2.770531000	-0.618573000
H	0.263196000	3.788203000	-0.233268000
H	0.217936000	2.814610000	-1.717076000
O	1.259294000	2.028564000	-0.104187000
C	2.667034000	0.097811000	-0.344151000
C	3.285834000	-0.796411000	-1.228315000
C	3.244636000	0.333885000	0.909952000
C	4.456883000	-1.462418000	-0.858181000
H	2.864983000	-0.963403000	-2.217746000
C	4.420822000	-0.326017000	1.275254000
H	2.789076000	1.048744000	1.586869000
C	5.025477000	-1.229341000	0.396797000
H	4.931444000	-2.147240000	-1.554215000
H	4.868642000	-0.128176000	2.244412000
H	5.941147000	-1.737591000	0.682586000
C	-1.005259000	-2.166099000	0.369061000
C	0.154602000	-1.191084000	0.581919000
C	0.096213000	-0.103960000	-0.532269000
C	-1.176589000	0.663122000	-0.322380000
H	0.110826000	-0.731378000	1.575637000
H	1.110938000	-1.711394000	0.509069000
H	-0.782104000	-2.791598000	-0.510302000
H	-1.103653000	-2.856228000	1.213201000
H	-0.057229000	-0.673862000	-1.464814000
C	-1.377781000	2.464319000	1.371666000
H	-1.422535000	3.550899000	1.485051000

H	-2.307812000	2.033225000	1.746240000
H	-0.542089000	2.091326000	1.967589000
H	1.432241000	0.961295000	-1.844577000

trans–4h, B
symmetry c1

C	-4.927067000	-1.087482000	-0.487774000
C	-3.896664000	-1.859630000	0.050893000
C	-2.609408000	-1.344023000	0.191064000
C	-2.353760000	0.012745000	-0.218796000
C	-3.431594000	0.773054000	-0.779259000
C	-4.693096000	0.233754000	-0.911597000
H	-5.921388000	-1.514098000	-0.582895000
H	-4.098432000	-2.878346000	0.367774000
H	-3.259593000	1.786520000	-1.118681000
H	-5.498216000	0.820331000	-1.340442000
C	1.388329000	0.417651000	0.837321000
C	-0.775188000	2.010879000	-0.425903000
H	-1.252107000	2.256105000	-1.382677000
C	0.739139000	2.245207000	-0.526031000
H	0.949593000	3.310492000	-0.642395000
H	1.178987000	1.713342000	-1.381676000
O	1.331724000	1.835687000	0.694791000
C	2.669868000	-0.145445000	0.209001000
C	3.818396000	0.661450000	0.209631000
C	2.768731000	-1.449099000	-0.299006000
C	5.025841000	0.183274000	-0.301981000
H	3.760014000	1.668906000	0.606994000
C	3.979253000	-1.929823000	-0.808706000
H	1.915451000	-2.121866000	-0.297282000
C	5.111005000	-1.113929000	-0.814421000
H	5.901291000	0.825436000	-0.297210000
H	4.033294000	-2.941834000	-1.198229000
H	6.050617000	-1.486016000	-1.210551000
C	-1.503911000	-2.209452000	0.730584000
C	-0.369335000	-1.388276000	1.335237000
C	0.083399000	-0.290821000	0.353279000
C	-1.056498000	0.578846000	-0.080658000
H	-0.693388000	-0.915160000	2.270614000
H	0.479689000	-2.030846000	1.581813000
H	-1.111276000	-2.810999000	-0.105824000
H	-1.909216000	-2.924235000	1.453193000
H	0.325229000	-0.790222000	-0.609661000
C	-1.373281000	2.950844000	0.672085000
H	-1.205046000	3.985163000	0.359130000
H	-2.444251000	2.797233000	0.812148000
H	-0.863221000	2.794907000	1.624382000
H	1.443231000	0.271345000	1.923377000

PRODUCTS

cis-7h, A

symmetry c1

C	-4.740495000	-1.468807000	0.264218000
C	-3.526189000	-2.147210000	0.123616000
C	-2.325333000	-1.454020000	-0.038705000
C	-2.324889000	-0.037469000	-0.066565000
C	-3.554306000	0.629482000	0.080635000
C	-4.749674000	-0.074008000	0.243286000
H	-5.665610000	-2.024536000	0.386932000
H	-3.509912000	-3.234630000	0.140588000
H	-3.590567000	1.713240000	0.064632000
H	-5.683680000	0.470110000	0.350911000
C	1.389513000	0.716690000	-0.920912000
C	-0.982008000	2.188711000	-0.000011000
H	-1.877593000	2.647974000	-0.439861000
C	0.214174000	2.772523000	-0.756055000
H	0.400242000	3.811124000	-0.466271000
H	0.029803000	2.743768000	-1.842468000
O	1.408984000	2.063310000	-0.446987000
C	2.569551000	0.008246000	-0.270254000
C	3.651604000	-0.426637000	-1.042158000
C	2.598474000	-0.199606000	1.115821000
C	4.744217000	-1.065317000	-0.445868000
H	3.643668000	-0.261365000	-2.116885000
C	3.688062000	-0.831626000	1.715348000
H	1.764344000	0.139789000	1.722604000
C	4.764387000	-1.269186000	0.934973000
H	5.577149000	-1.397064000	-1.059310000
H	3.699087000	-0.984047000	2.791014000
H	5.611850000	-1.762753000	1.402076000
C	-0.032884000	-1.439594000	-1.064242000
C	-1.004415000	-2.180908000	-0.143352000
C	-1.040047000	0.688001000	-0.248078000
H	-1.160431000	-3.207950000	-0.490748000
H	-0.377342000	-1.518077000	-2.108357000
H	0.952956000	-1.912135000	-1.024633000
H	-0.555275000	-2.250626000	0.859525000
C	0.056595000	0.023488000	-0.682299000
C	-0.946143000	2.546978000	1.498964000
H	-1.031826000	3.630867000	1.639595000
H	-1.765102000	2.068530000	2.044504000
H	-0.002505000	2.220258000	1.945923000
H	1.567702000	0.722648000	-2.014060000

trans-7h, A

symmetry c1

C	4.714000000	-1.326016000	0.617308000
C	3.806529000	-1.819519000	-0.325092000
C	2.582110000	-1.187270000	-0.548343000
C	2.243046000	-0.025327000	0.188028000
C	3.167177000	0.459734000	1.129758000

C	4.389228000	-0.181174000	1.345440000
H	5.661071000	-1.832307000	0.779990000
H	4.053725000	-2.710802000	-0.897407000
H	2.935230000	1.345004000	1.712523000
H	5.081848000	0.214482000	2.082818000
C	-1.413024000	0.547526000	-0.958129000
C	0.702480000	2.060850000	0.409277000
H	1.082570000	2.168888000	1.434481000
C	-0.800105000	2.352333000	0.474133000
H	-0.988544000	3.424868000	0.578236000
H	-1.255846000	1.841098000	1.334792000
O	-1.452317000	1.962798000	-0.732326000
C	-2.562118000	-0.150087000	-0.223630000
C	-3.840868000	-0.137334000	-0.800360000
C	-2.398474000	-0.755527000	1.029829000
C	-4.930659000	-0.708534000	-0.141858000
H	-3.981399000	0.332531000	-1.770795000
C	-3.487953000	-1.327168000	1.694656000
H	-1.413320000	-0.788642000	1.486577000
C	-4.756470000	-1.305334000	1.111052000
H	-5.913177000	-0.690283000	-0.605120000
H	-3.342954000	-1.792461000	2.665683000
H	-5.602275000	-1.752942000	1.625127000
C	0.163342000	-1.481036000	-1.143705000
C	1.611934000	-1.685904000	-1.594975000
C	0.929914000	0.628231000	-0.052390000
H	1.803087000	-2.740159000	-1.822148000
H	-0.075653000	-2.178552000	-0.326570000
H	-0.526398000	-1.724140000	-1.960377000
H	1.776135000	-1.124320000	-2.527837000
C	-0.053428000	-0.055030000	-0.681495000
C	1.443201000	3.073508000	-0.488122000
H	1.359052000	4.086540000	-0.077686000
H	2.506274000	2.829735000	-0.574246000
H	1.011139000	3.074523000	-1.493590000
H	-1.611189000	0.453019000	-2.034145000

cis-7h, B
symmetry c1

C	-4.762351000	-1.281285000	0.619633000
C	-3.729557000	-1.980874000	-0.011824000
C	-2.516184000	-1.360460000	-0.315194000
C	-2.313620000	-0.001161000	0.027413000
C	-3.369321000	0.694238000	0.643491000
C	-4.579675000	0.063994000	0.940654000
H	-5.698348000	-1.781116000	0.851612000
H	-3.870609000	-3.025308000	-0.280825000
H	-3.251990000	1.740360000	0.902569000
H	-5.374239000	0.625436000	1.423863000
C	1.401300000	0.486545000	-0.994507000
C	-0.893839000	2.163642000	-0.274800000
H	-1.819662000	2.589091000	-0.686489000

C	0.252284000	2.562237000	-1.209955000
H	0.468087000	3.632975000	-1.145008000
H	-0.000628000	2.319713000	-2.254482000
O	1.457934000	1.903787000	-0.828919000
C	2.567092000	-0.102561000	-0.211997000
C	3.667855000	-0.644508000	-0.883623000
C	2.567832000	-0.090641000	1.189878000
C	4.751294000	-1.171255000	-0.172946000
H	3.681542000	-0.649521000	-1.970904000
C	3.648031000	-0.610997000	1.903066000
H	1.718544000	0.329160000	1.721124000
C	4.743219000	-1.155335000	1.222889000
H	5.598676000	-1.588320000	-0.709717000
H	3.636245000	-0.594346000	2.989310000
H	5.582989000	-1.562532000	1.778745000
C	-1.431898000	-2.084637000	-1.080587000
C	-0.035377000	-1.623022000	-0.659992000
C	0.056379000	-0.112821000	-0.619771000
C	-1.015443000	0.645313000	-0.293329000
H	0.214558000	-2.026004000	0.333205000
H	0.717745000	-2.030338000	-1.344623000
H	-1.566217000	-1.875712000	-2.153665000
H	-1.534734000	-3.168232000	-0.957491000
C	-0.664724000	2.749551000	1.136072000
H	0.333088000	2.483120000	1.495618000
H	-0.734431000	3.843482000	1.111719000
H	-1.393217000	2.380361000	1.862626000
H	1.569353000	0.251664000	-2.063264000

trans-7h, B
symmetry c1

C	4.745150000	-1.417921000	0.337440000
C	3.603933000	-2.079827000	-0.124621000
C	2.380983000	-1.416785000	-0.244609000
C	2.285525000	-0.044252000	0.094287000
C	3.438335000	0.601304000	0.577329000
C	4.655380000	-0.072791000	0.696270000
H	5.688781000	-1.949306000	0.420532000
H	3.661545000	-3.132846000	-0.391111000
H	3.395359000	1.645474000	0.864958000
H	5.529058000	0.455823000	1.066744000
C	-1.388785000	0.723605000	-0.886258000
C	0.800048000	2.050045000	0.541438000
H	1.289577000	2.092885000	1.524262000
C	-0.692925000	2.311480000	0.760099000
H	-0.877086000	3.357136000	1.023818000
H	-1.086467000	1.679316000	1.569199000
O	-1.420514000	2.088730000	-0.446961000
C	-2.544328000	-0.057776000	-0.250979000
C	-3.814381000	0.015935000	-0.841284000
C	-2.398191000	-0.792801000	0.933708000
C	-4.913100000	-0.622409000	-0.263541000

H	-3.941727000	0.585699000	-1.758583000
C	-3.496391000	-1.431581000	1.517753000
H	-1.419836000	-0.874523000	1.398401000
C	-4.756626000	-1.348579000	0.921097000
H	-5.888566000	-0.555257000	-0.737073000
H	-3.364923000	-1.997152000	2.436096000
H	-5.609000000	-1.848330000	1.372408000
C	1.128270000	-2.159926000	-0.652579000
C	0.153410000	-1.260760000	-1.413439000
C	-0.036222000	0.067204000	-0.712123000
C	0.980292000	0.653183000	-0.038954000
H	0.536243000	-1.066708000	-2.428761000
H	-0.807398000	-1.770947000	-1.538707000
H	0.630683000	-2.529520000	0.257697000
H	1.385031000	-3.043437000	-1.247023000
C	1.403413000	3.145316000	-0.367440000
H	1.380466000	4.117574000	0.138910000
H	2.438373000	2.932263000	-0.645983000
H	0.818495000	3.227339000	-1.288174000
H	-1.597234000	0.788690000	-1.963478000

cis-12h, A
symmetry c1

C	4.708180000	-1.721093000	0.162062000
C	3.516731000	-2.242237000	-0.341304000
C	2.354081000	-1.467756000	-0.420857000
C	2.375037000	-0.107885000	-0.013351000
C	3.572325000	0.374762000	0.560037000
C	4.722184000	-0.409115000	0.638205000
H	5.599425000	-2.339654000	0.214763000
H	3.475679000	-3.280229000	-0.663410000
H	3.605298000	1.366113000	0.987605000
H	5.622186000	0.003562000	1.085364000
C	-1.311696000	0.744126000	0.393218000
C	1.100148000	2.080281000	-0.114641000
C	-0.219815000	2.822513000	-0.100478000
H	-0.264677000	3.520929000	-0.946733000
H	-0.243329000	3.442364000	0.816308000
O	-1.406285000	2.051379000	-0.178686000
C	-2.677540000	0.101596000	0.273810000
C	-3.221605000	-0.613090000	1.348043000
C	-3.410367000	0.191278000	-0.918448000
C	-4.466063000	-1.239762000	1.233559000
H	-2.670630000	-0.677853000	2.283317000
C	-4.655560000	-0.429668000	-1.034229000
H	-3.008637000	0.766240000	-1.746649000
C	-5.186476000	-1.150691000	0.040235000
H	-4.873851000	-1.788814000	2.077673000
H	-5.214453000	-0.347052000	-1.962322000
H	-6.155993000	-1.632216000	-0.050231000
C	-0.102770000	-1.507616000	-0.039239000
C	1.052601000	-2.104899000	-0.848061000

C	1.146595000	0.724353000	-0.140526000
H	1.112419000	-3.189929000	-0.709552000
H	0.071171000	-1.679198000	1.031936000
H	-1.047900000	-1.993619000	-0.298389000
H	0.863505000	-1.935904000	-1.918953000
C	-0.189313000	-0.011814000	-0.348803000
H	-0.450436000	0.064690000	-1.415893000
C	2.260304000	3.050744000	-0.088843000
H	2.588126000	3.292801000	0.931126000
H	3.125975000	2.674902000	-0.638750000
H	1.960339000	3.999068000	-0.550553000
H	-1.045825000	0.818615000	1.460496000

trans-12h, A
symmetry c1

C	-4.414364000	-1.133933000	-0.868750000
C	-3.804459000	-1.416694000	0.353024000
C	-2.608047000	-0.800568000	0.736760000
C	-1.999972000	0.162039000	-0.113987000
C	-2.599596000	0.379044000	-1.374560000
C	-3.789284000	-0.245740000	-1.745097000
H	-5.342889000	-1.624868000	-1.145368000
H	-4.252919000	-2.147544000	1.022074000
H	-2.116893000	1.019630000	-2.098092000
H	-4.216051000	-0.047431000	-2.724240000
C	1.540404000	0.417236000	1.355176000
C	-0.232808000	1.985340000	-0.208864000
C	1.136900000	2.490164000	0.201416000
H	1.071011000	3.542690000	0.503827000
H	1.784633000	2.459931000	-0.694985000
O	1.776817000	1.834364000	1.284157000
C	2.306814000	-0.335133000	0.267984000
C	3.695802000	-0.463736000	0.436793000
C	1.728294000	-0.879798000	-0.887126000
C	4.485039000	-1.109896000	-0.514899000
H	4.160725000	-0.044733000	1.325888000
C	2.515020000	-1.533087000	-1.842119000
H	0.660234000	-0.799659000	-1.052072000
C	3.894302000	-1.649394000	-1.661790000
H	5.556866000	-1.196698000	-0.359929000
H	2.044164000	-1.951352000	-2.727474000
H	4.502912000	-2.157828000	-2.404188000
C	-0.405127000	-1.221133000	1.814050000
C	-1.923532000	-1.227999000	2.016221000
C	-0.743599000	0.840002000	0.309635000
H	-2.280447000	-2.221945000	2.307183000
H	-0.136769000	-1.921539000	1.014921000
H	0.109561000	-1.554362000	2.723428000
H	-2.179469000	-0.548565000	2.843193000
C	0.025419000	0.209726000	1.483117000
H	-0.236606000	0.801561000	2.373592000
C	-0.857442000	2.891154000	-1.246342000

H	-0.609138000	2.591007000	-2.273639000
H	-1.945998000	2.927714000	-1.161963000
H	-0.480446000	3.913046000	-1.122694000
H	1.994393000	0.140459000	2.313427000

cis-12h, B
symmetry c1

C	4.637695000	-1.804954000	0.130106000
C	3.443655000	-2.211785000	-0.464511000
C	2.300729000	-1.400814000	-0.445931000
C	2.370970000	-0.127121000	0.167204000
C	3.571355000	0.254982000	0.797045000
C	4.695728000	-0.568470000	0.778900000
H	5.509063000	-2.452980000	0.101851000
H	3.390520000	-3.184126000	-0.950068000
H	3.612192000	1.196953000	1.332829000
H	5.608114000	-0.250423000	1.275269000
C	-1.354120000	0.829437000	0.477742000
C	1.125178000	2.063264000	-0.039160000
C	-0.204330000	2.784944000	-0.143148000
H	-0.171359000	3.497720000	-0.974519000
H	-0.394182000	3.370509000	0.776843000
O	-1.313350000	1.941782000	-0.410423000
C	-2.689849000	0.125138000	0.341407000
C	-3.109013000	-0.757567000	1.347193000
C	-3.515381000	0.322213000	-0.772246000
C	-4.322441000	-1.440166000	1.238530000
H	-2.486564000	-0.907780000	2.226640000
C	-4.732415000	-0.357505000	-0.880166000
H	-3.203056000	1.016591000	-1.544154000
C	-5.139290000	-1.242632000	0.121162000
H	-4.632829000	-2.118147000	2.028698000
H	-5.364270000	-0.191810000	-1.748472000
H	-6.085864000	-1.768657000	0.036186000
C	1.007297000	-1.939153000	-1.041735000
C	-0.178707000	-0.957257000	-1.035159000
C	-0.132271000	-0.082454000	0.232808000
C	1.155833000	0.726965000	0.160273000
H	-0.144700000	-0.303395000	-1.913848000
H	-1.115762000	-1.520643000	-1.088698000
H	0.728488000	-2.831837000	-0.463402000
H	1.194289000	-2.295842000	-2.062296000
H	-0.066189000	-0.755454000	1.101418000
C	2.321065000	2.966746000	-0.225796000
H	2.582794000	3.496623000	0.700559000
H	3.206857000	2.420427000	-0.555150000
H	2.102005000	3.736956000	-0.975260000
H	-1.272879000	1.208200000	1.511844000

trans-12h, B
symmetry c1

C	4.629398000	-0.841572000	-1.114439000

C	4.072633000	0.434230000	-1.197821000
C	2.805299000	0.717742000	-0.670672000
C	2.087135000	-0.308628000	-0.012515000
C	2.645158000	-1.601460000	0.030345000
C	3.901897000	-1.869845000	-0.509077000
H	5.613338000	-1.035506000	-1.531828000
H	4.627857000	1.230645000	-1.689171000
H	2.072167000	-2.411372000	0.468318000
H	4.306571000	-2.876947000	-0.464462000
C	-1.445202000	1.271197000	0.215503000
C	0.268281000	-0.434926000	1.738905000
C	-1.075609000	0.055221000	2.235183000
H	-1.019992000	0.257722000	3.310149000
H	-1.841874000	-0.727711000	2.092639000
O	-1.504395000	1.277554000	1.651587000
C	-2.487851000	0.318126000	-0.369184000
C	-3.837256000	0.700633000	-0.298634000
C	-2.178026000	-0.922988000	-0.941665000
C	-4.848560000	-0.127660000	-0.785938000
H	-4.092780000	1.658453000	0.147875000
C	-3.188813000	-1.757399000	-1.431519000
H	-1.145492000	-1.250642000	-1.007286000
C	-4.525850000	-1.363501000	-1.356021000
H	-5.885350000	0.191347000	-0.725487000
H	-2.927180000	-2.715359000	-1.872337000
H	-5.309493000	-2.010324000	-1.740126000
C	2.222330000	2.109807000	-0.869950000
C	0.852595000	2.343942000	-0.208809000
C	0.001871000	1.057200000	-0.253701000
C	0.752382000	0.009731000	0.557869000
H	0.970375000	2.647725000	0.837418000
H	0.336602000	3.162638000	-0.725871000
H	2.124524000	2.270405000	-1.953214000
H	2.941497000	2.862556000	-0.523692000
H	-0.033231000	0.728050000	-1.302276000
C	0.958322000	-1.385982000	2.687401000
H	0.560457000	-2.406836000	2.602096000
H	2.035624000	-1.435491000	2.520005000
H	0.791500000	-1.071861000	3.724845000
H	-1.750762000	2.288869000	-0.051855000

7s
symmetry c1

C	-4.638861000	-0.602377000	0.249785000
C	-3.619592000	-1.556802000	0.194321000
C	-2.285369000	-1.181133000	0.026817000
C	-1.941776000	0.188538000	-0.094722000
C	-2.981637000	1.135538000	-0.031429000
C	-4.312543000	0.749445000	0.137208000
C	-0.515793000	0.578481000	-0.252809000
C	0.424267000	-0.358087000	-0.543328000
H	-5.672000000	-0.912239000	0.377481000

H	-3.863155000	-2.613364000	0.280947000
H	-2.760629000	2.193752000	-0.113678000
H	-5.090353000	1.506621000	0.178953000
C	-1.179166000	-2.209140000	0.000186000
H	-0.785107000	-2.343874000	1.020289000
H	-1.570860000	-3.181269000	-0.318730000
C	-0.046092000	-1.754029000	-0.918683000
H	-0.417306000	-1.719076000	-1.956180000
H	0.763451000	-2.480826000	-0.913478000
C	1.899165000	-0.025408000	-0.708299000
H	2.193547000	-0.369568000	-1.720603000
C	-0.140373000	2.051666000	-0.217562000
H	-0.929836000	2.611315000	-0.736862000
C	1.122418000	2.229442000	-1.062046000
H	1.519135000	3.246665000	-0.980667000
H	0.894934000	2.030551000	-2.122029000
O	2.162165000	1.378063000	-0.610814000
C	-0.001287000	2.638480000	1.199420000
H	0.169242000	3.720676000	1.153899000
H	-0.906269000	2.463325000	1.789635000
H	0.839077000	2.182522000	1.728512000
C	2.926448000	-0.683372000	0.295485000
C	2.924784000	-2.224280000	0.239652000
H	3.785844000	-2.602148000	0.802216000
H	2.030574000	-2.660500000	0.693503000
H	3.016977000	-2.599671000	-0.786455000
C	2.638875000	-0.238295000	1.741313000
H	1.631130000	-0.528402000	2.057856000
H	3.354379000	-0.706812000	2.426922000
H	2.732126000	0.845449000	1.844091000
C	4.339091000	-0.207855000	-0.116033000
H	5.087698000	-0.641828000	0.556516000
H	4.580634000	-0.530004000	-1.136587000
H	4.424084000	0.879333000	-0.074700000

12s
symmetry c1

C	-4.510766000	-0.952518000	0.553385000
C	-3.534499000	-1.699876000	-0.103471000
C	-2.244715000	-1.194151000	-0.315750000
C	-1.930380000	0.113494000	0.120196000
C	-2.916288000	0.840849000	0.817352000
C	-4.191940000	0.322925000	1.029090000
H	-5.503513000	-1.365705000	0.707381000
H	-3.770579000	-2.701768000	-0.456261000
H	-2.665333000	1.814103000	1.224213000
H	-4.929659000	0.906651000	1.572343000
C	-1.199461000	-2.080428000	-0.968271000
H	-0.992399000	-2.918139000	-0.286016000
H	-1.611826000	-2.533800000	-1.878538000
C	0.115889000	-1.360814000	-1.286590000
H	0.006903000	-0.761664000	-2.200186000

H	0.893920000	-2.101858000	-1.487185000
C	1.956340000	0.155907000	-0.323565000
H	2.173126000	0.174709000	-1.408318000
C	1.157519000	2.385091000	-0.467739000
H	1.289378000	3.350980000	0.035816000
H	1.446873000	2.533117000	-1.526890000
O	2.050249000	1.495289000	0.165785000
C	-1.268386000	3.063389000	-0.650612000
H	-0.896109000	3.686011000	-1.475503000
H	-2.256849000	2.694181000	-0.929353000
H	-1.384991000	3.732922000	0.212868000
C	3.110467000	-0.630696000	0.373822000
C	3.115590000	-2.113272000	-0.047044000
H	3.980146000	-2.616578000	0.400125000
H	2.220203000	-2.644847000	0.290847000
H	3.196086000	-2.227667000	-1.134420000
C	2.990654000	-0.540674000	1.909858000
H	2.073510000	-1.012348000	2.278958000
H	3.835530000	-1.057656000	2.379111000
H	2.995311000	0.499727000	2.243562000
C	4.450934000	0.000819000	-0.062072000
H	5.289886000	-0.530591000	0.401571000
H	4.579146000	-0.058704000	-1.150037000
H	4.504704000	1.052434000	0.229387000
H	0.480061000	-0.973596000	0.806796000
C	0.524742000	-0.408181000	-0.134500000
C	-0.285840000	1.953048000	-0.373796000
C	-0.565780000	0.661384000	-0.099718000

Part 3: Selected-NMR Spectra

^{1}H-NMR of compound *cis*-**7c** (300 MHz, $CDCl_3$)

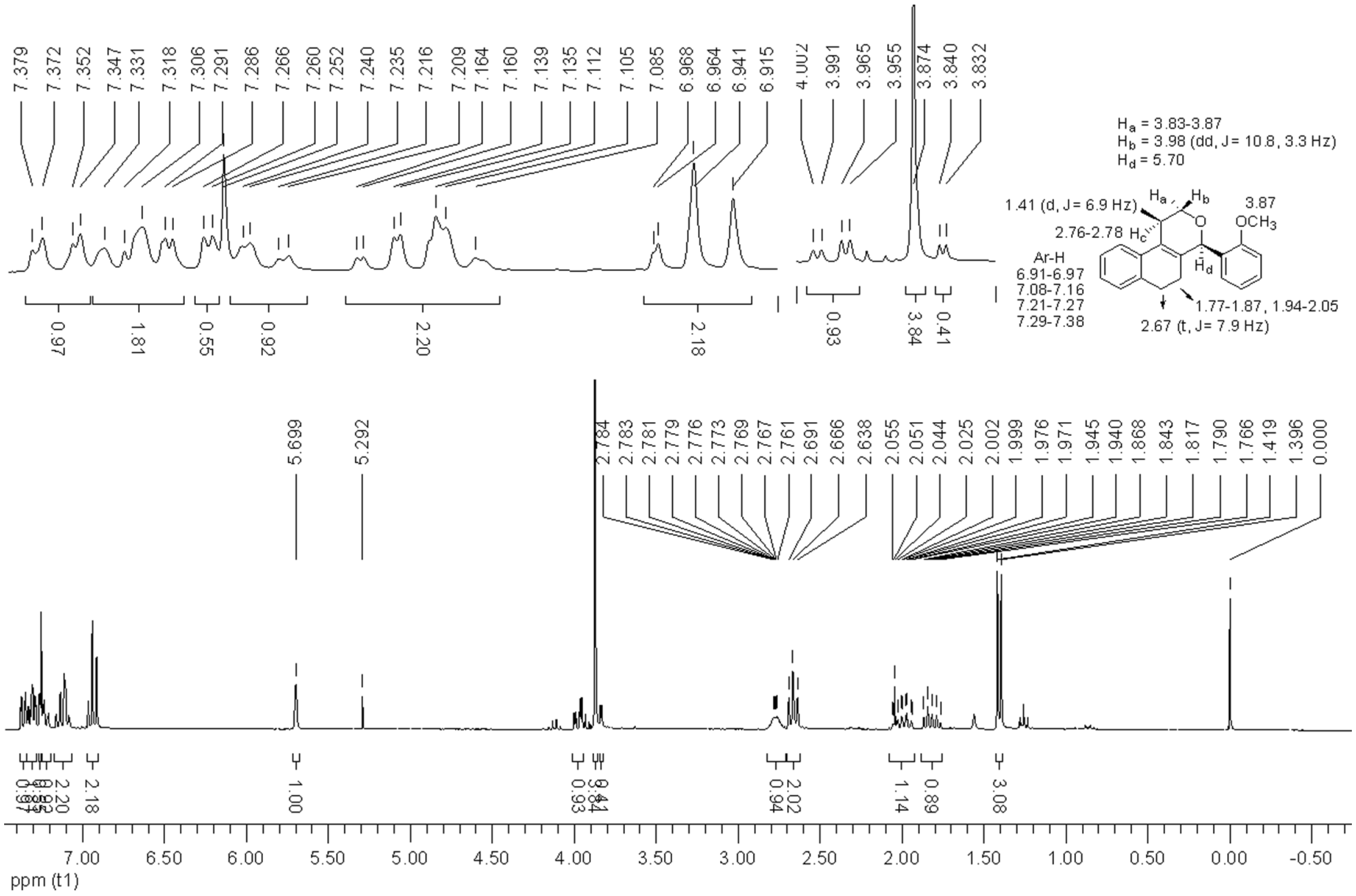

^{13}C-NMR of compound *cis*-**7c** (75 MHz, $CDCl_3$)

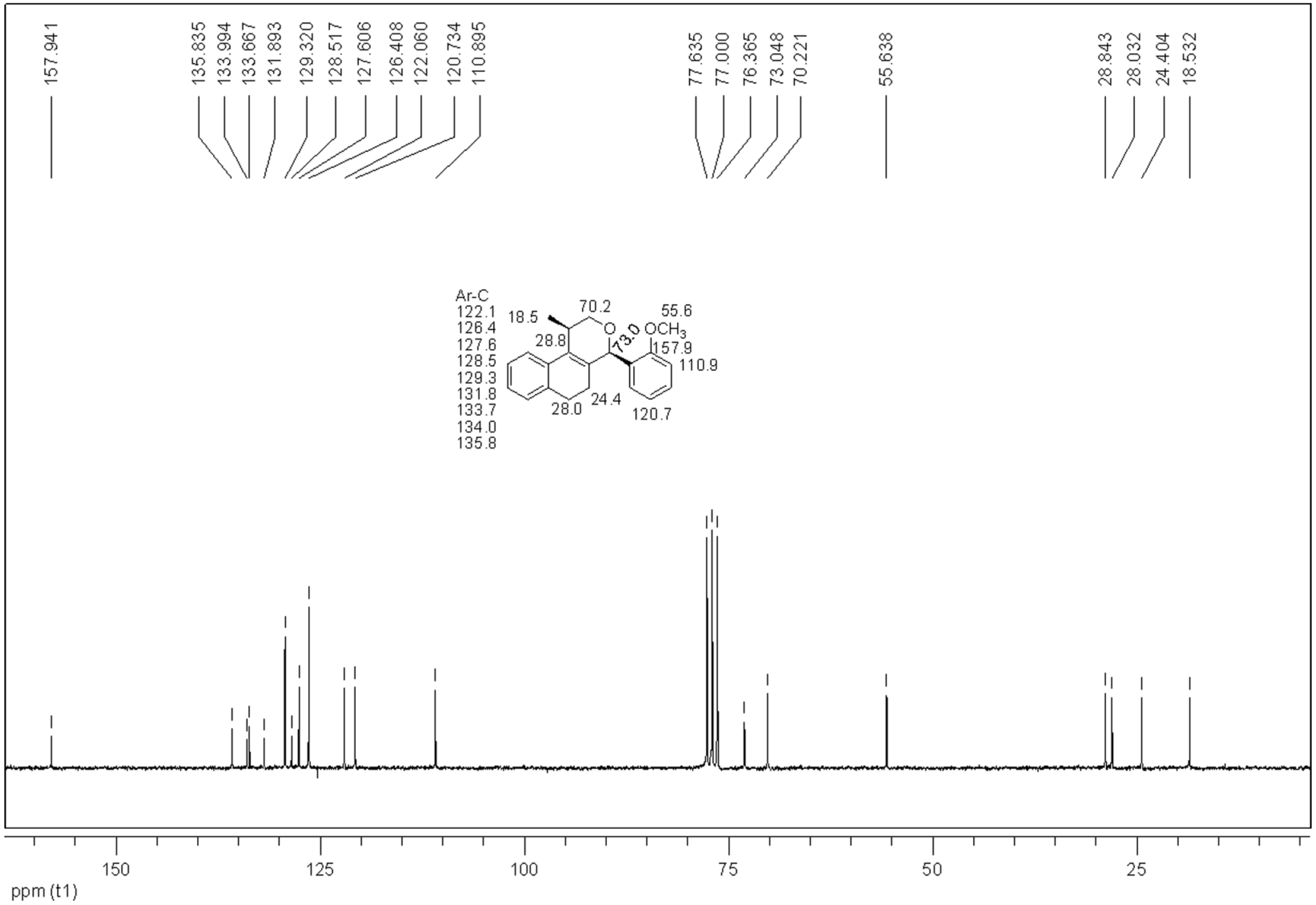

2D NOESY-NMR of compound *cis*-**7c** (300 MHz, $CDCl_3$)

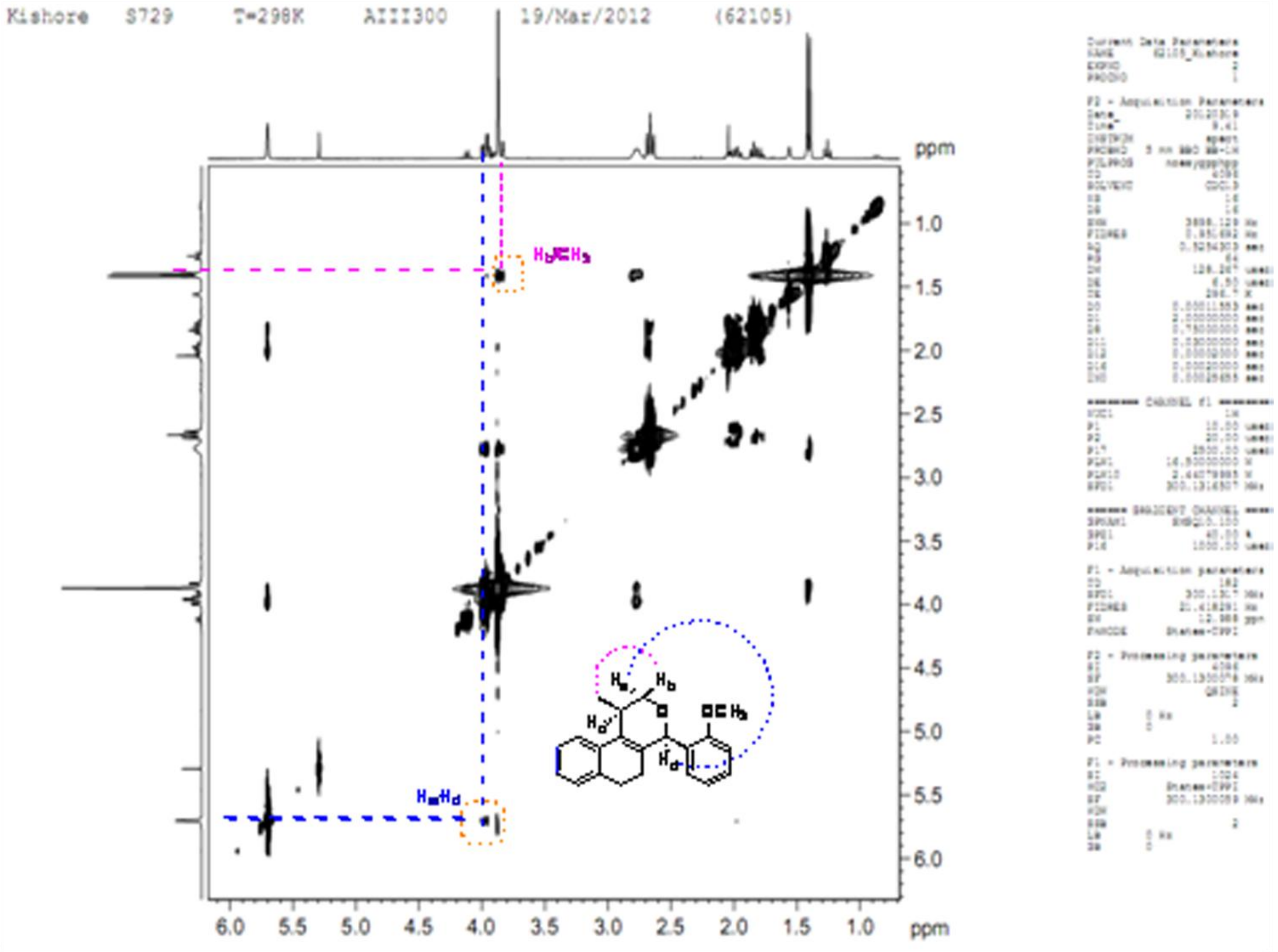

^{1}H-NMR of compound *trans*-**7c** (300 MHz, $CDCl_3$)

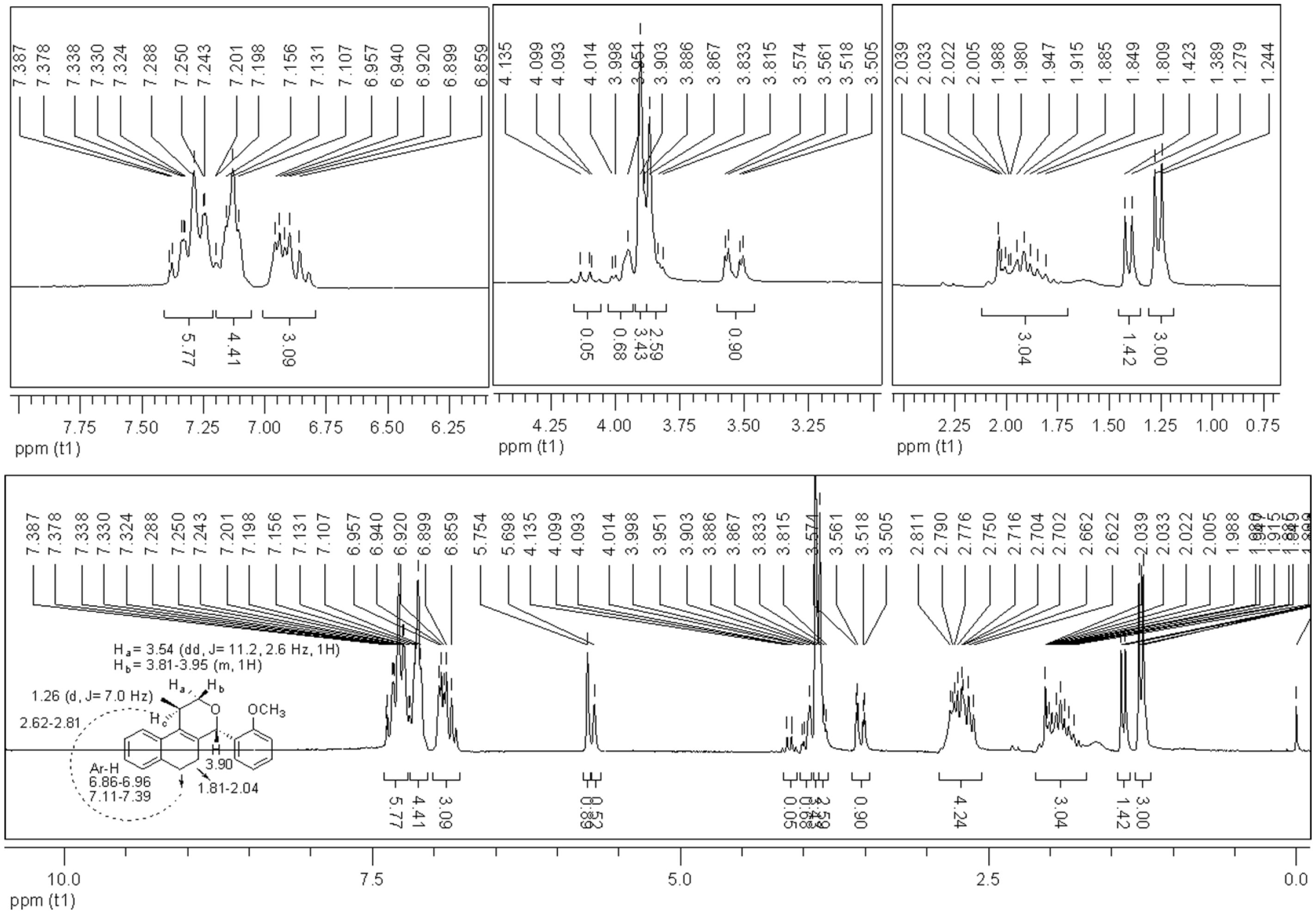

^{13}C-NMR of compound *trans*-**7c** (75 MHz, $CDCl_3$)

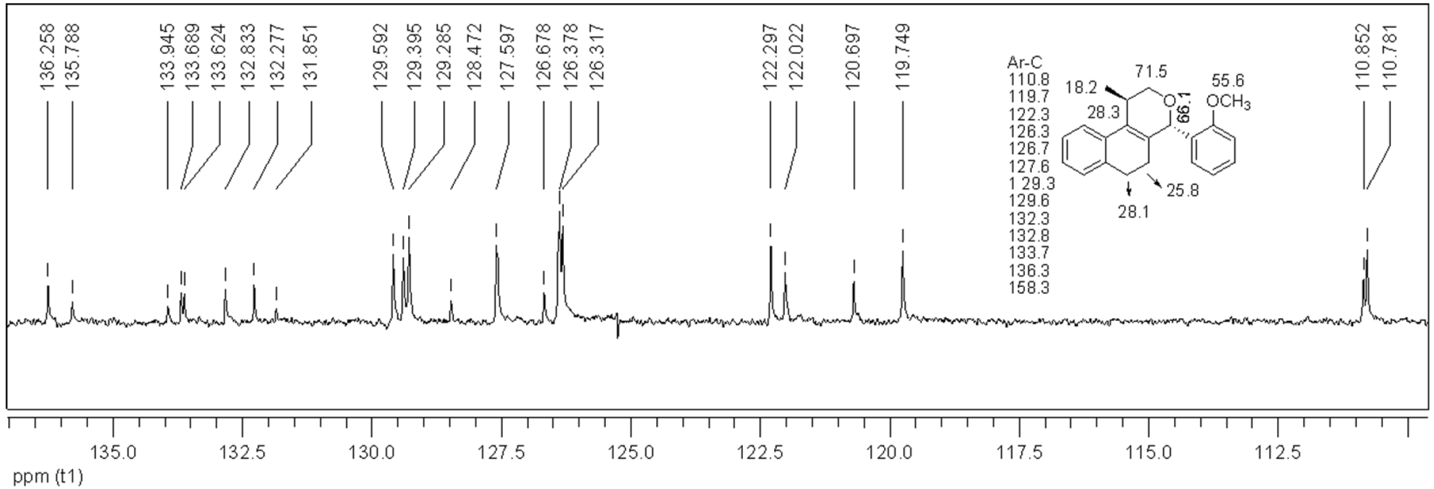

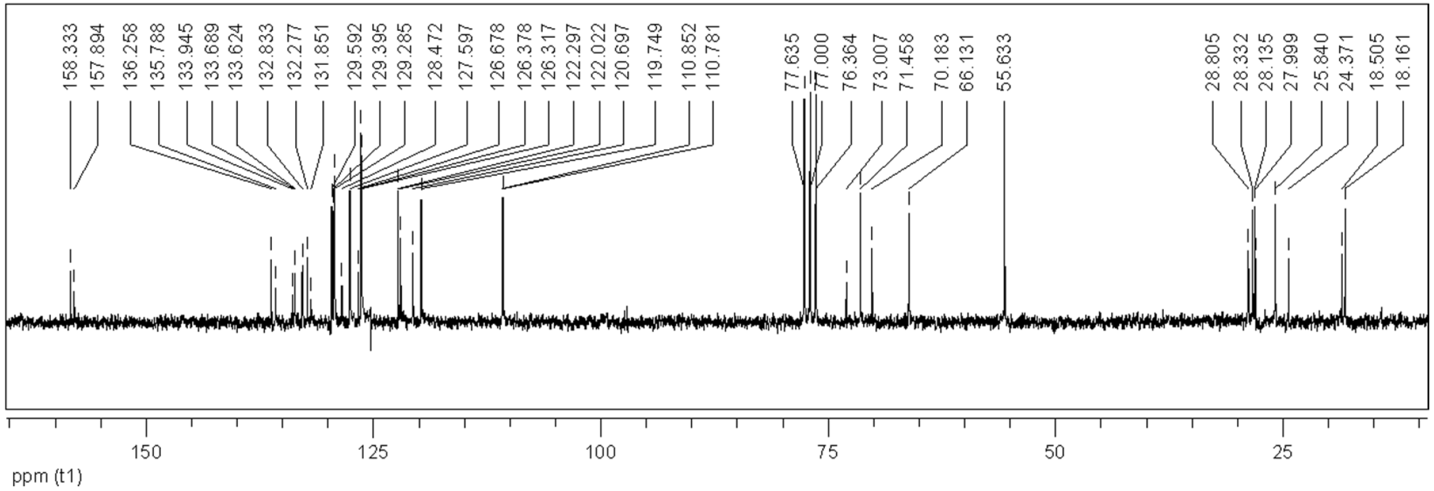

^{1}H-NMR of compound *trans*-**7d** (200 MHz, $CDCl_3$)

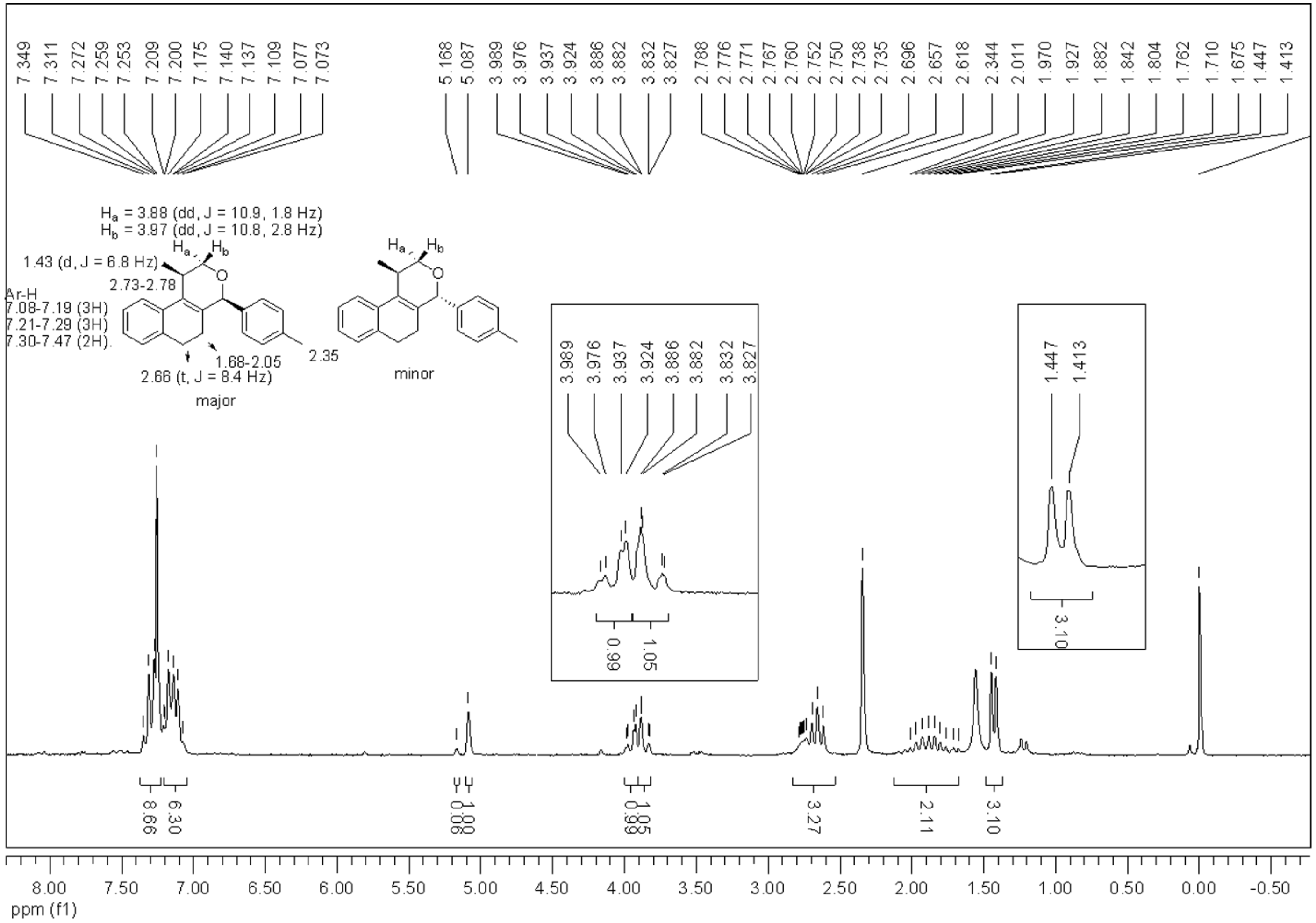

^{13}C-NMR of compound *cis*-**7d** (50 MHz, $CDCl_3$)

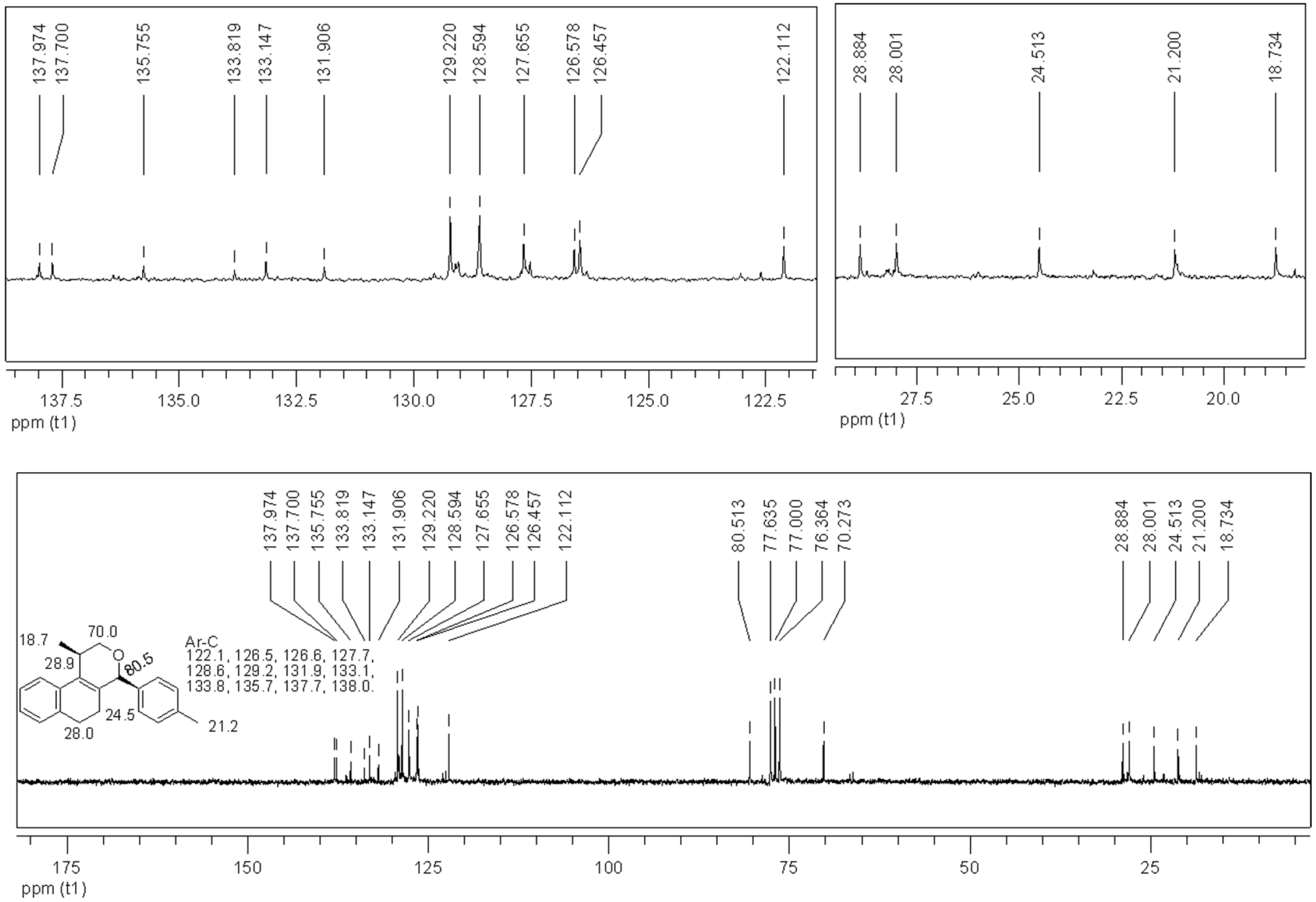

^{1}H-NMR of compounds **12d** and *trans*-**7d** (200 MHz, $CDCl_3$)

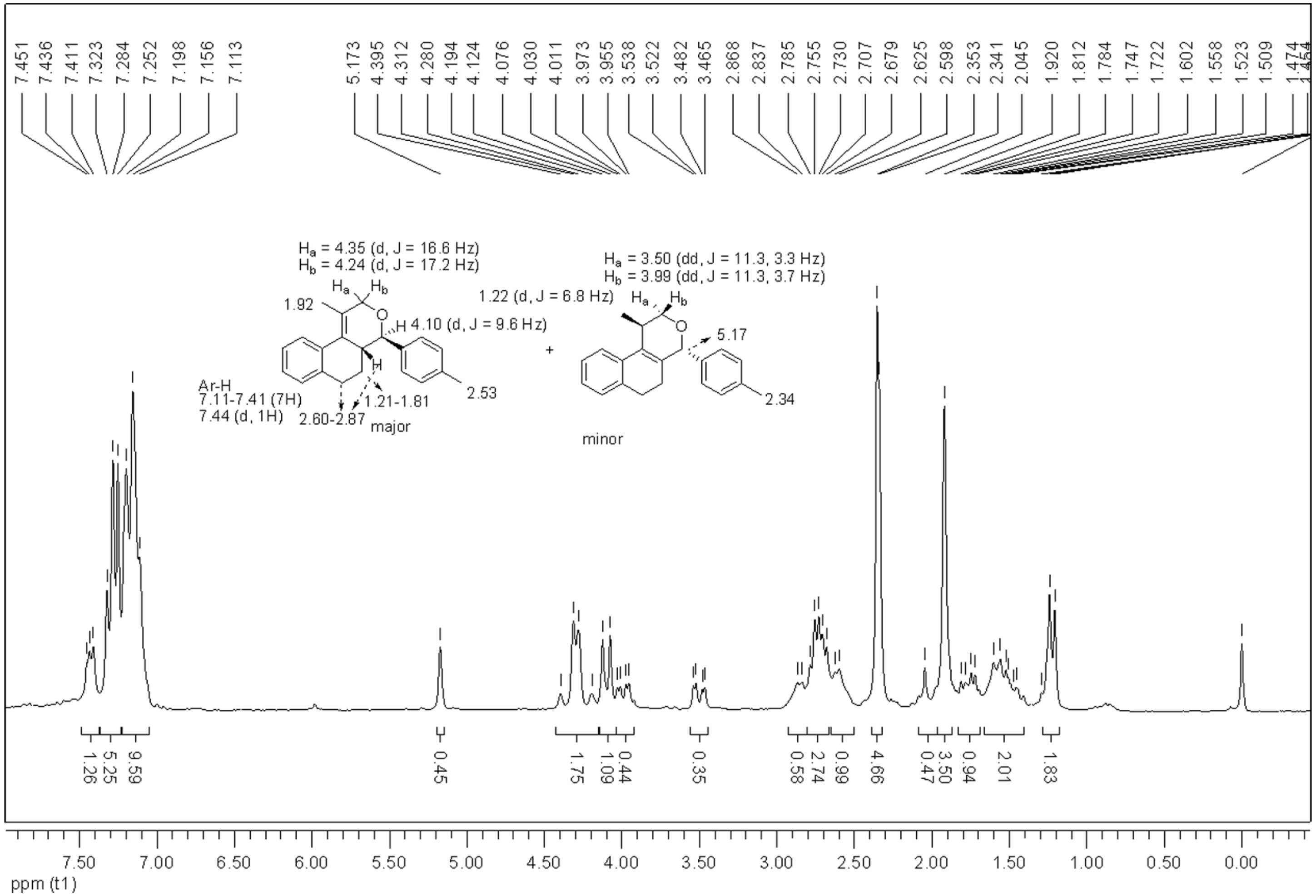

^{1}H-NMR of compounds **12d** and *trans*-**7d** (200 MHz, $CDCl_3$, selected expansions)

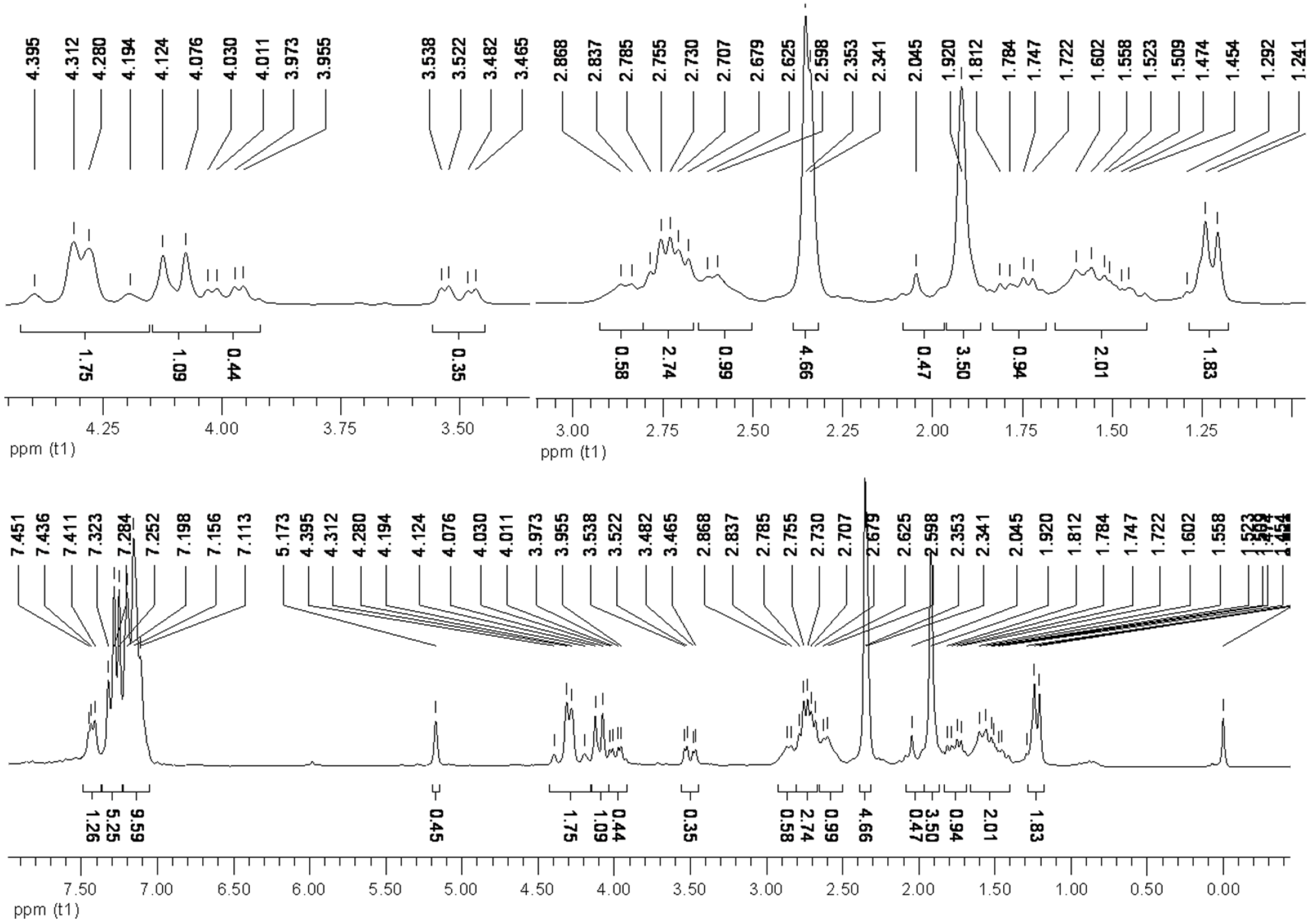

^{13}C-NMR of compounds **12d** and *trans*-**7d** (75 MHz, $CDCl_3$)

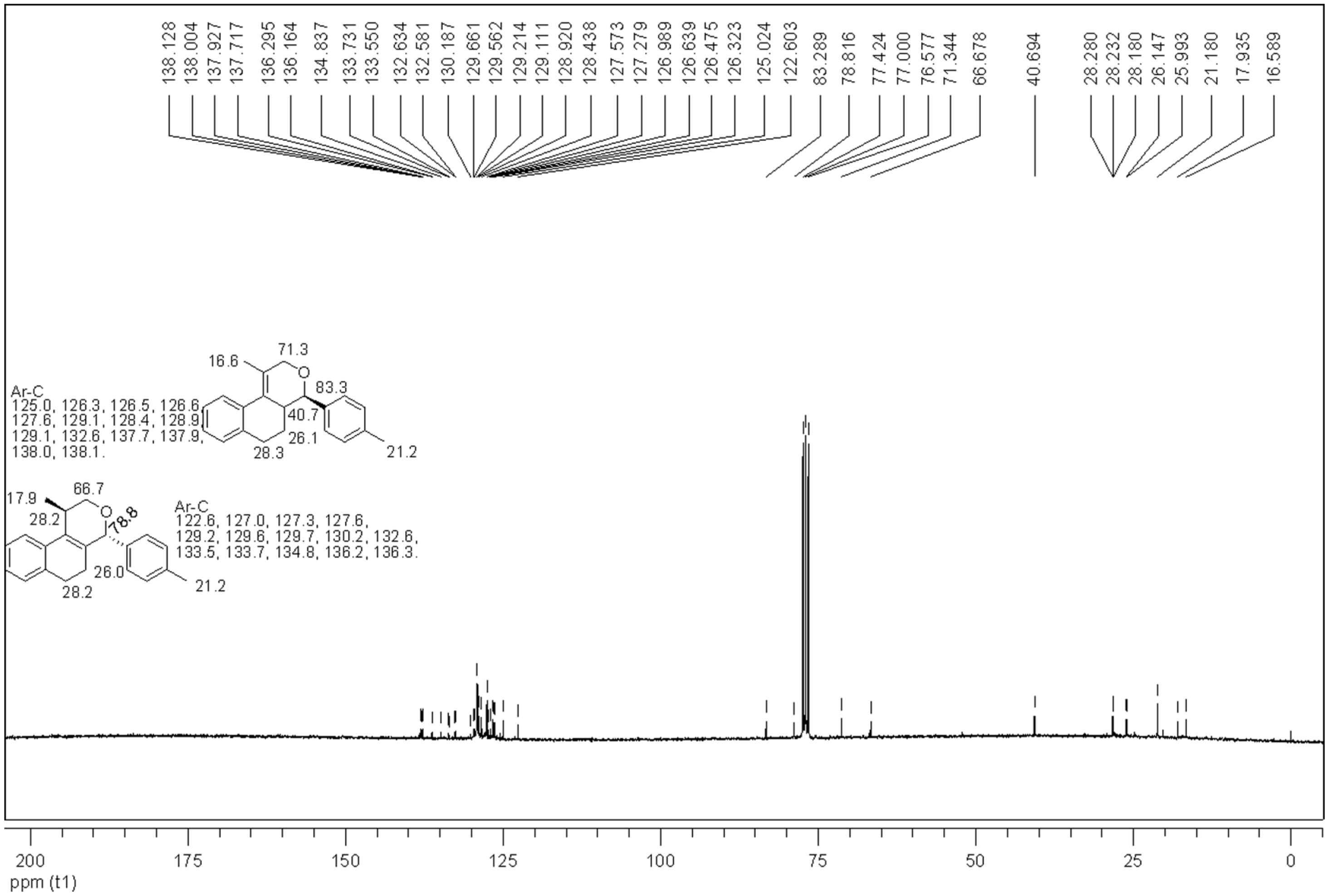

^{13}C-NMR of compounds **12d** and *trans*-**7d** (75 MHz, $CDCl_3$, selected expansions)

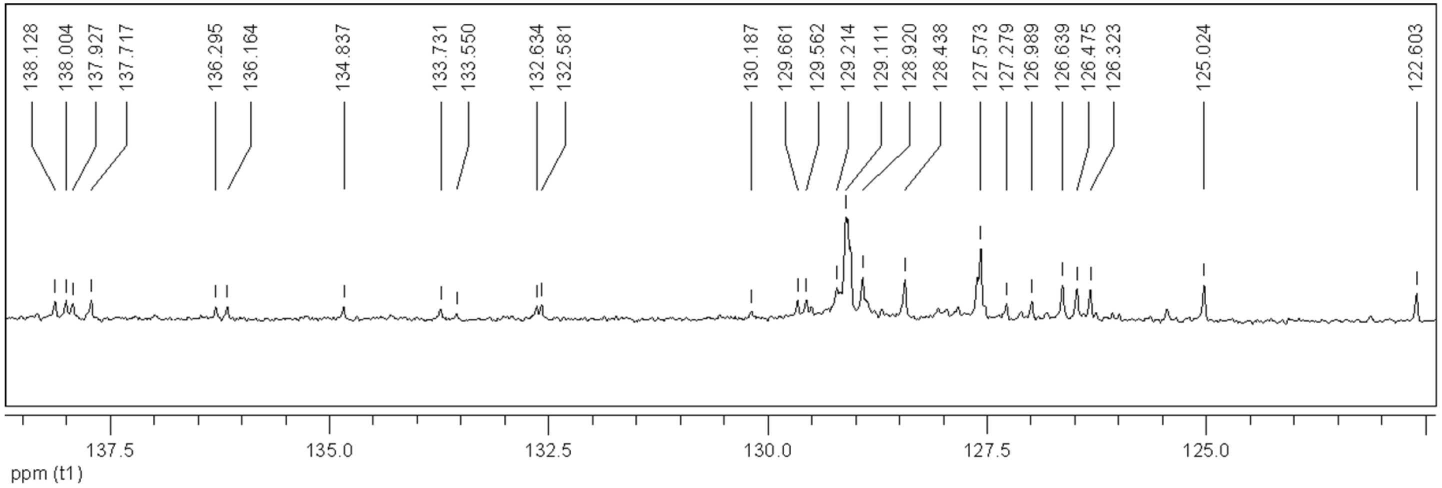

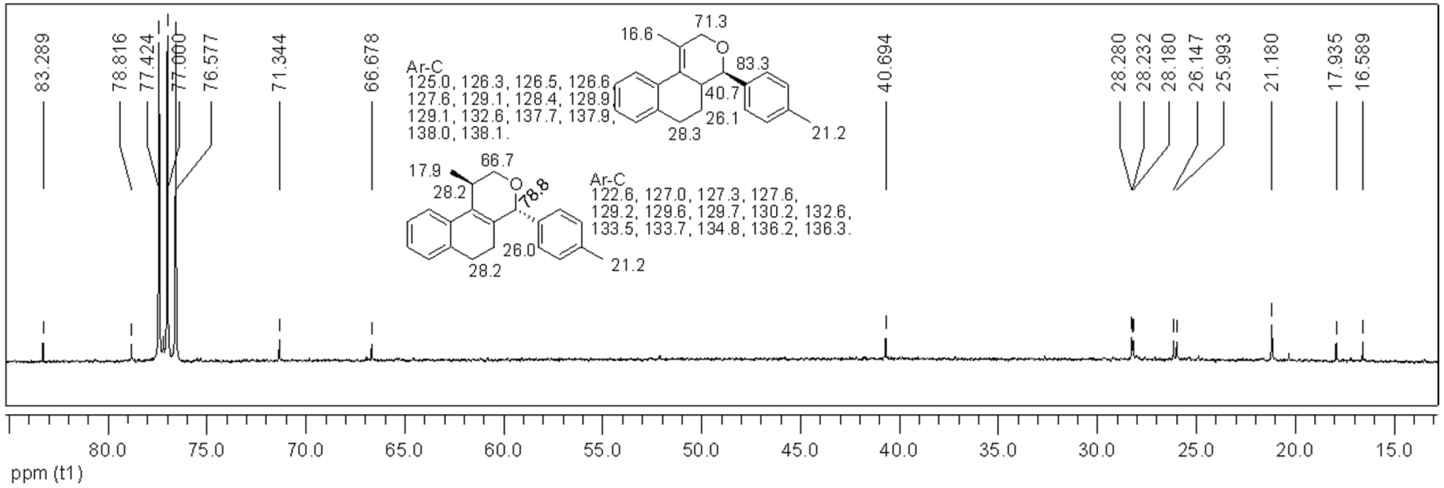

^{1}H-NMR of compounds *cis*-**7e** and *trans*-**7e** (200 MHz, $CDCl_3$)

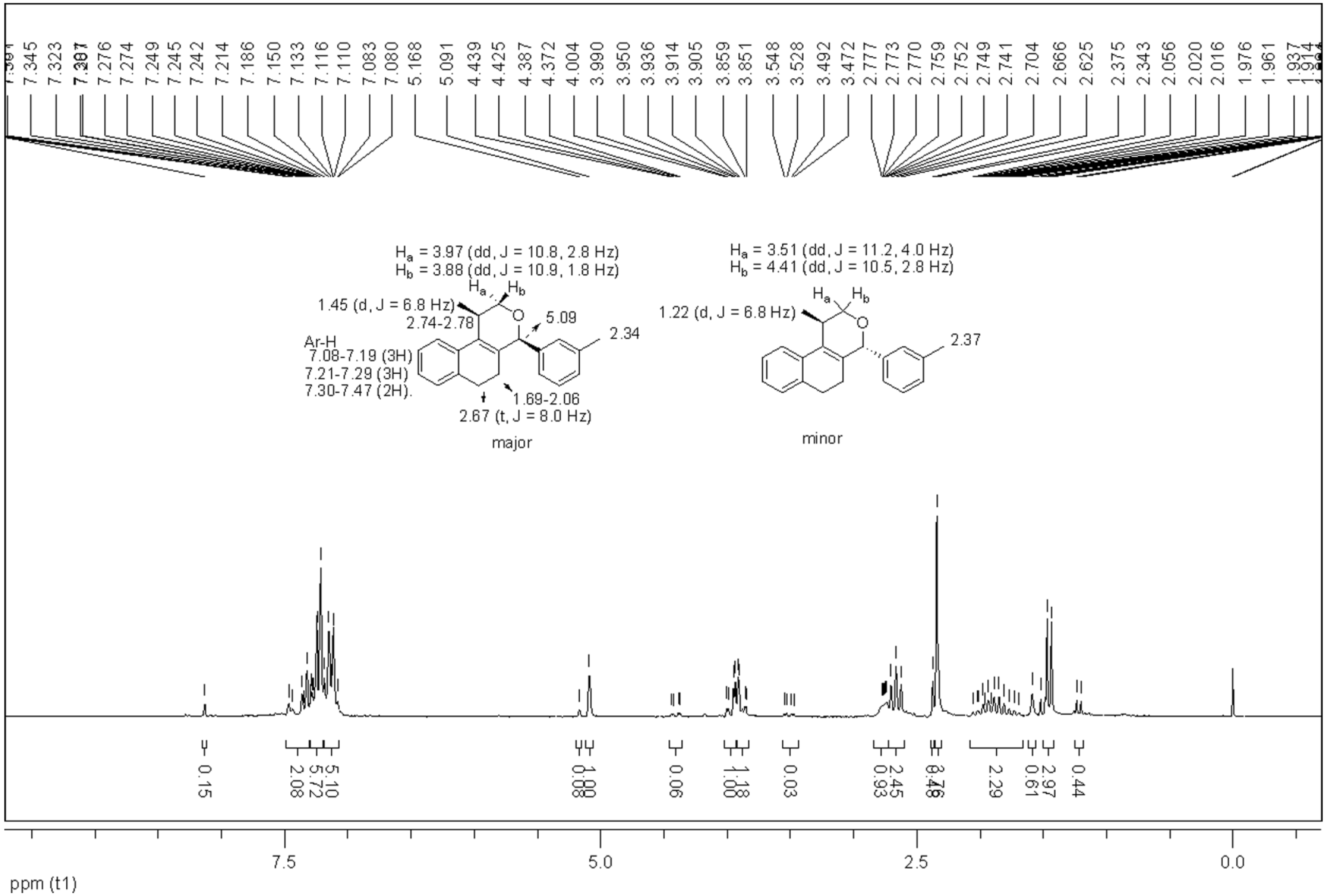

^{1}H-NMR of compounds *cis*-**7e** and *trans*-**7e** (200 MHz, $CDCl_3$)

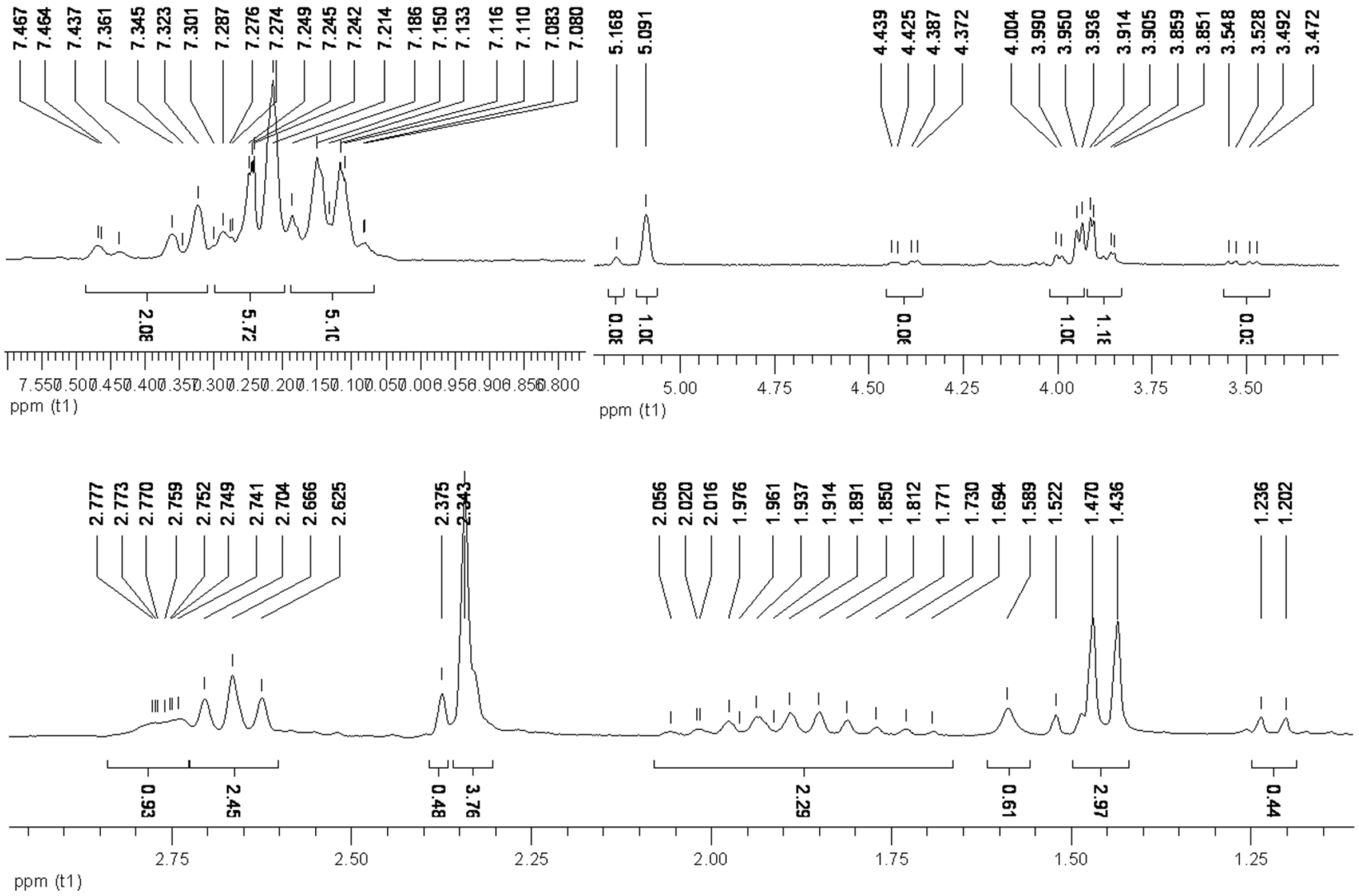

^{13}C-NMR of compound *cis*-**7e** (50 MHz, $CDCl_3$)

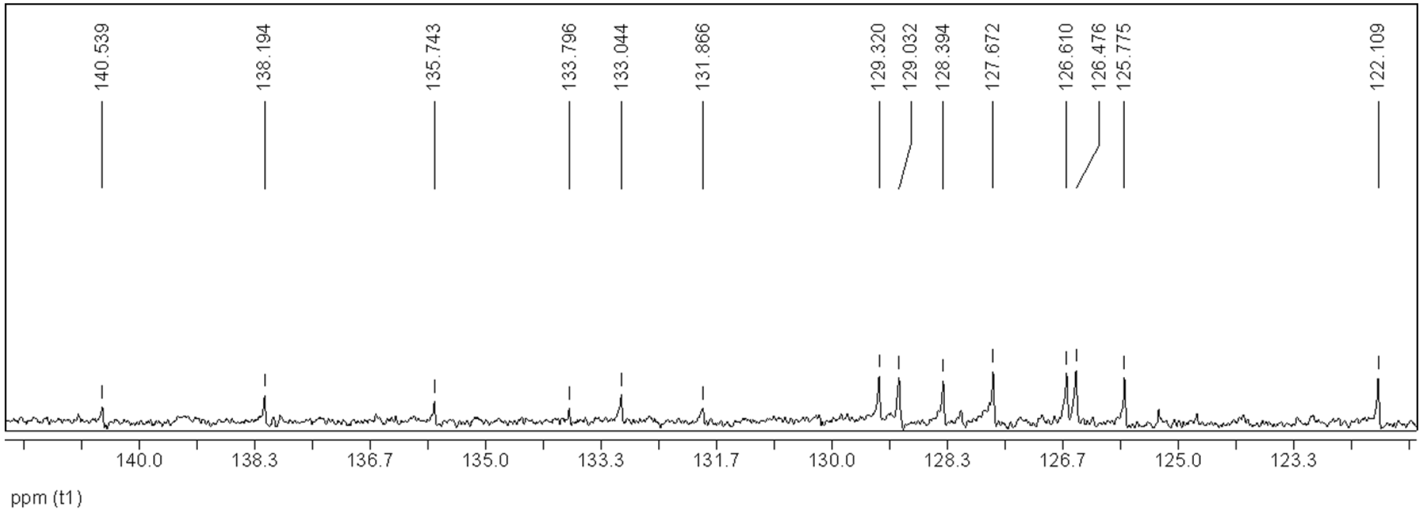

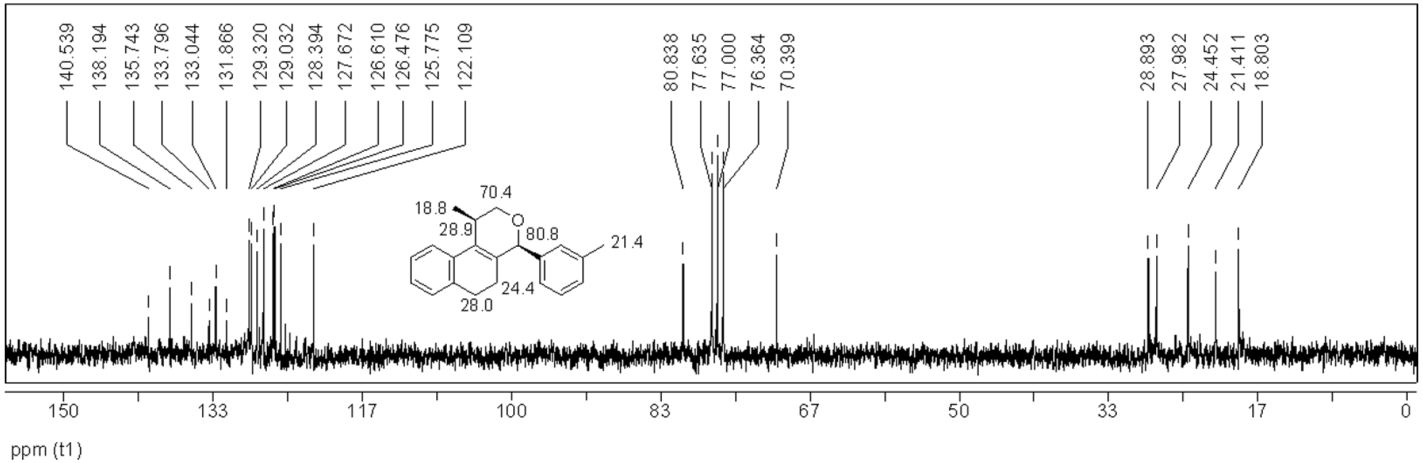

^{1}H-NMR of compound **12e** (500 MHz, $CDCl_3$)

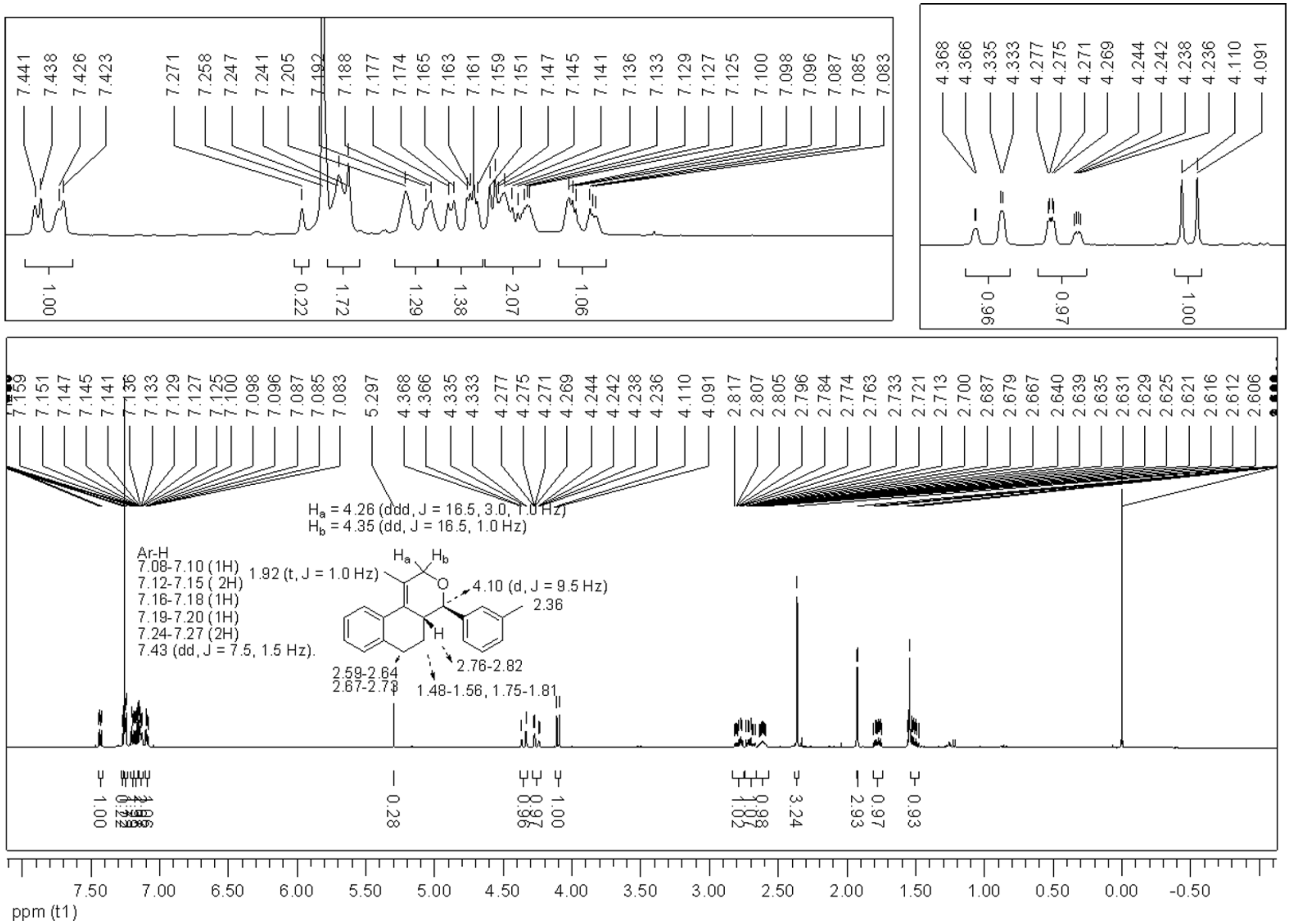

^{1}H-NMR of compound **12e** (300 MHz, $CDCl_3$)

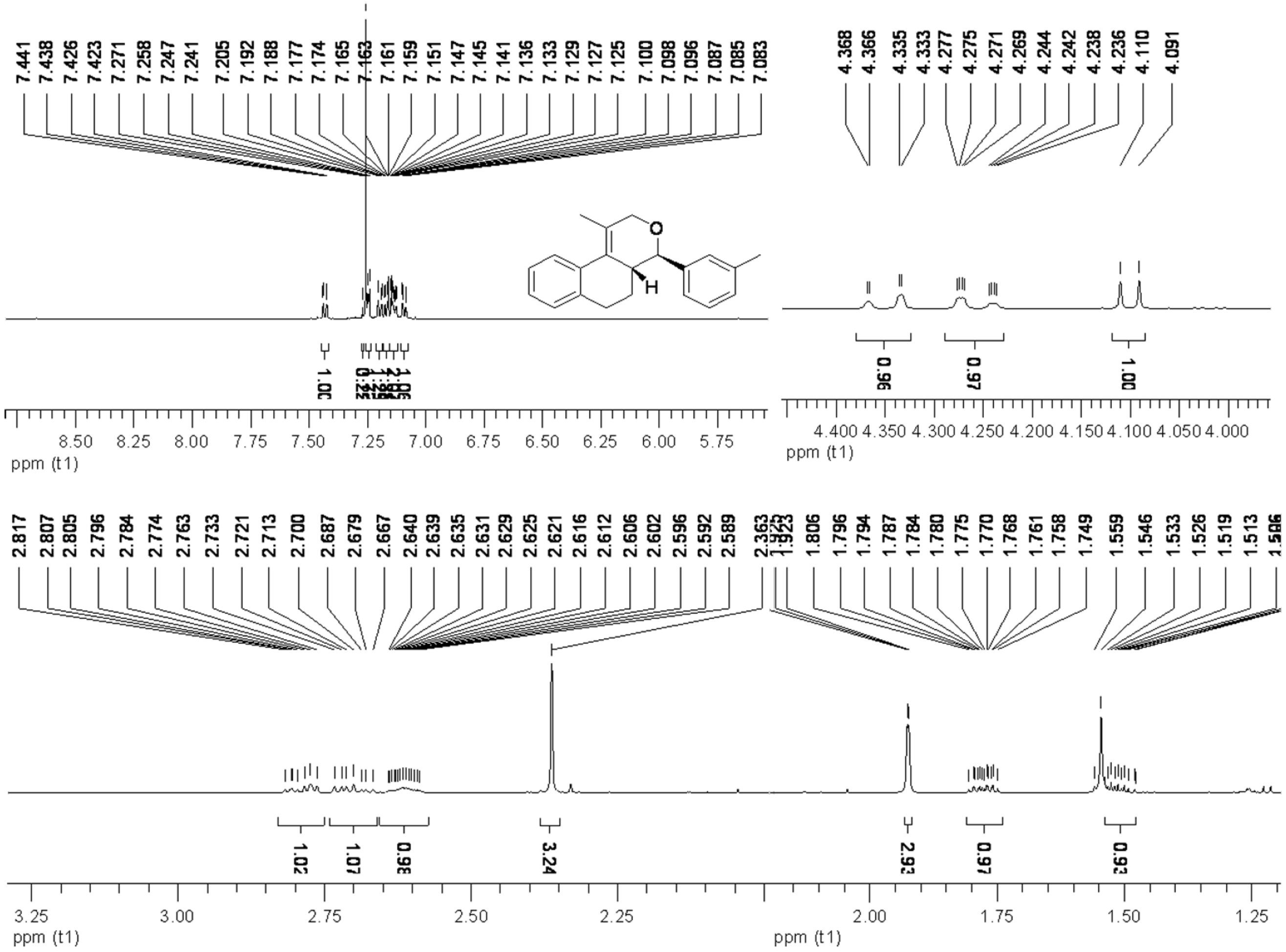

^{13}C-NMR of compound **12e** (75 MHz, $CDCl_3$)

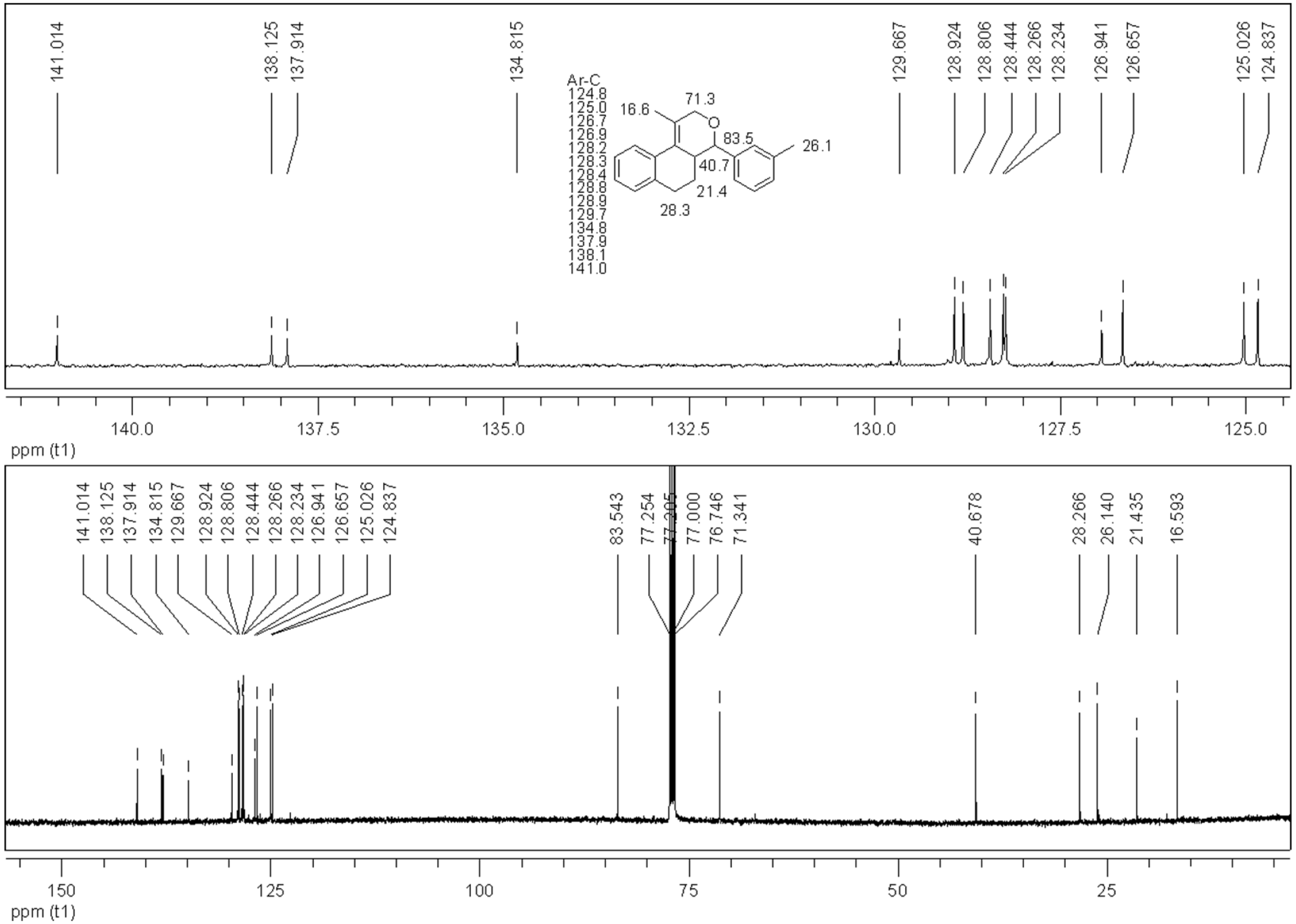

^{1}H-NMR of compound *cis*-**7f** (300 MHz, $CDCl_3$)

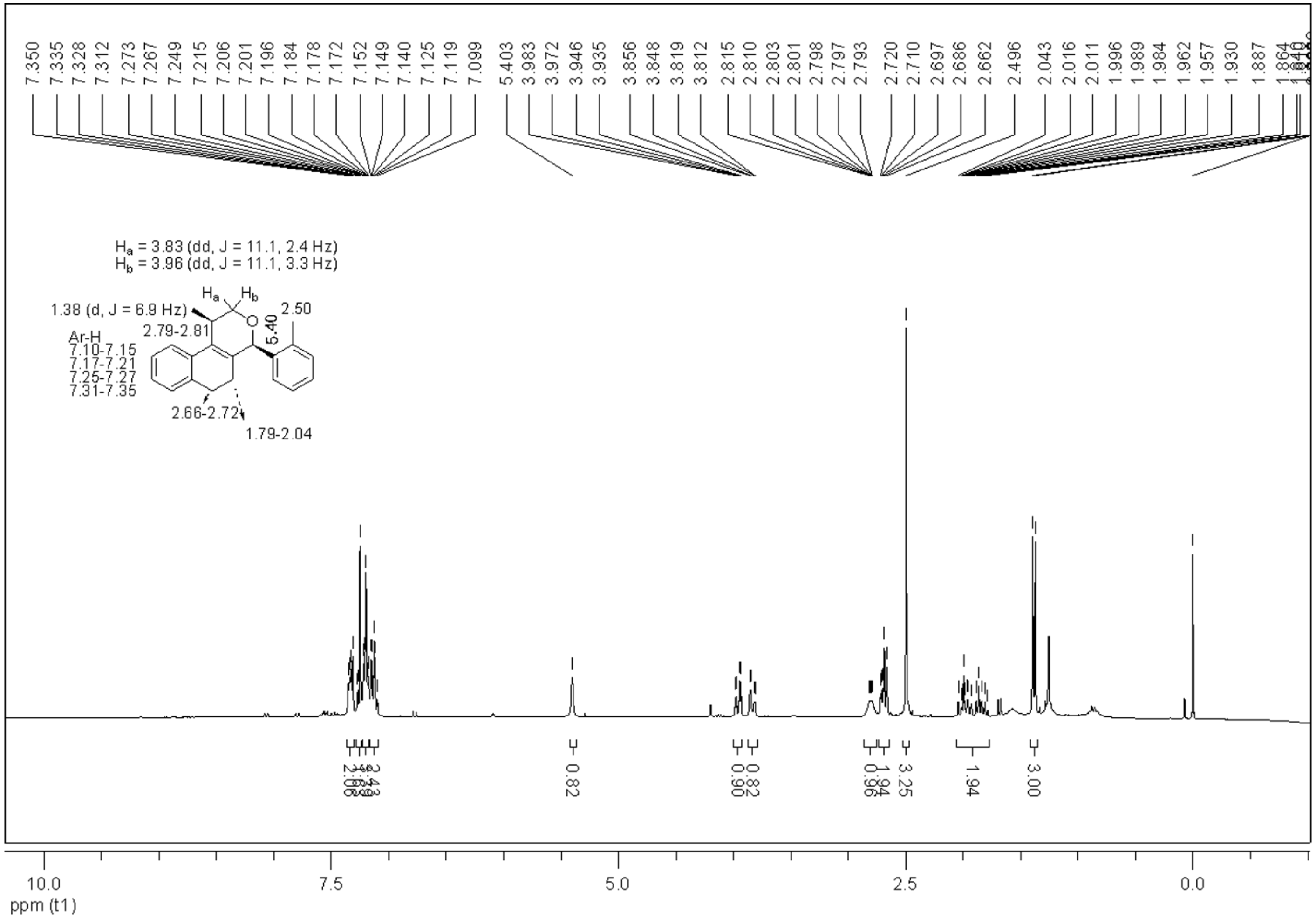

^{1}H-NMR of compound *cis*-**7f** (300 MHz, $CDCl_3$)

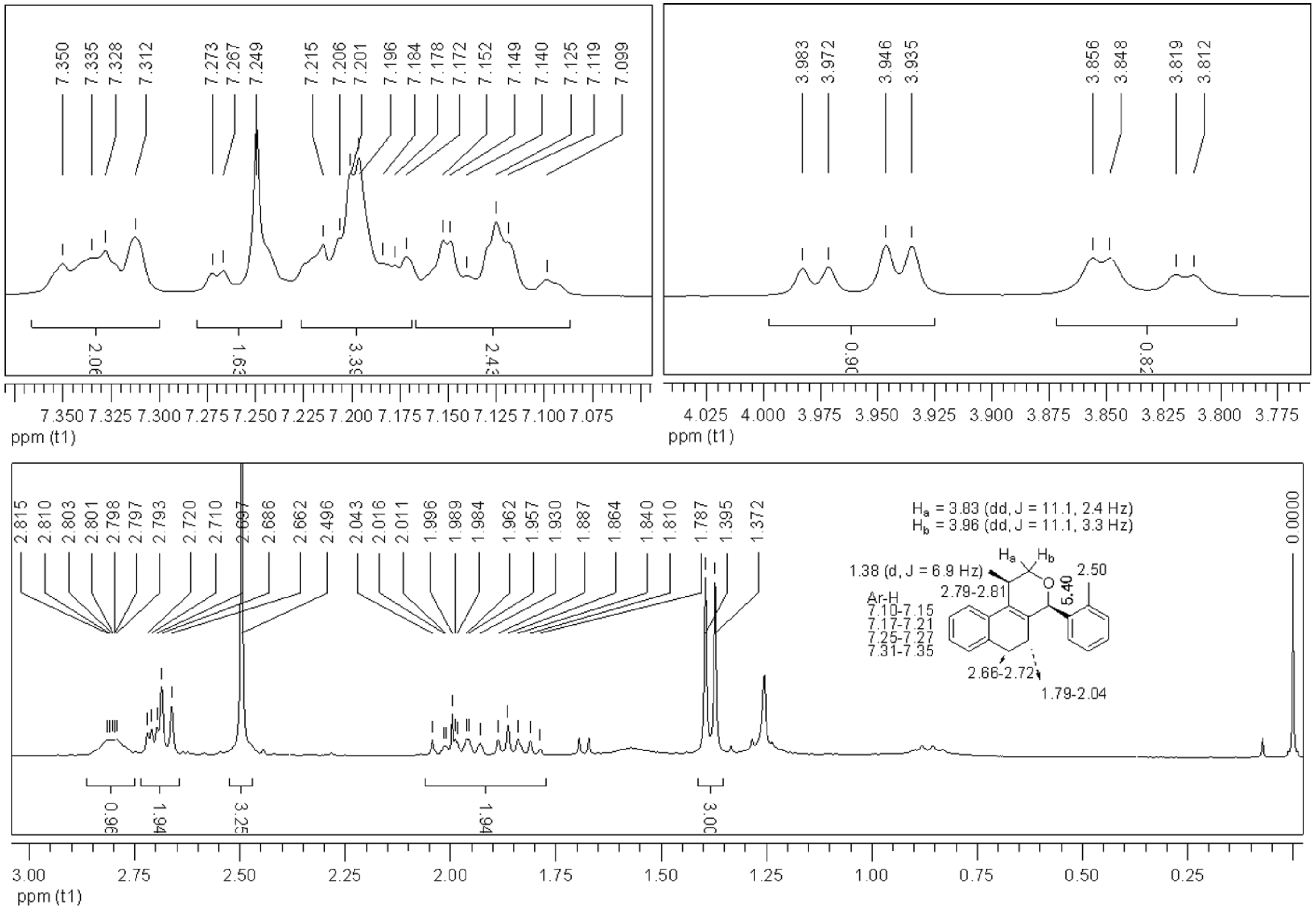

^{13}C-NMR of compound *cis*-**7f** (75 MHz, $CDCl_3$)

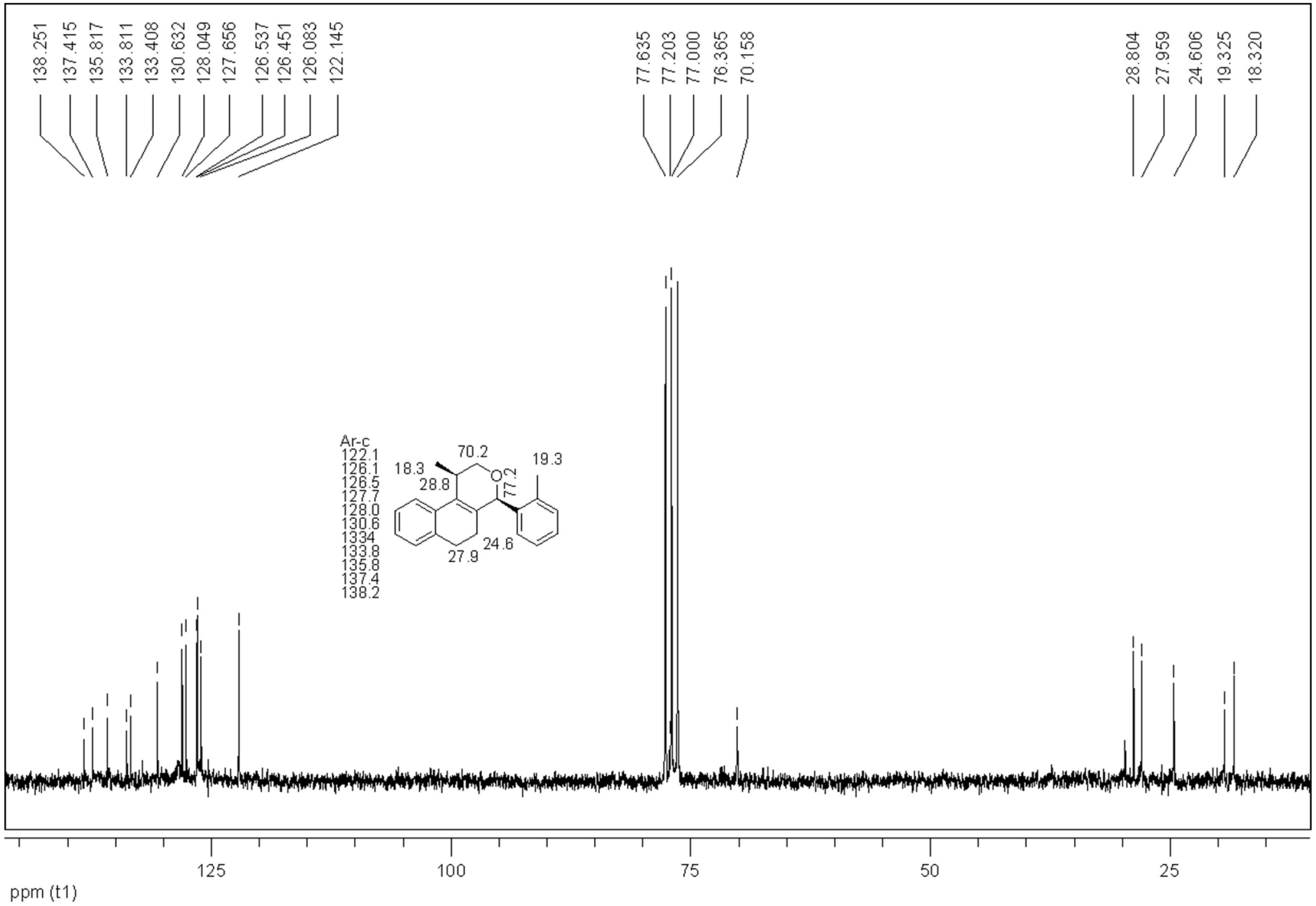

2D NOESY NMR of compound *cis*-**7f** (300 MHz, $CDCl_3$)

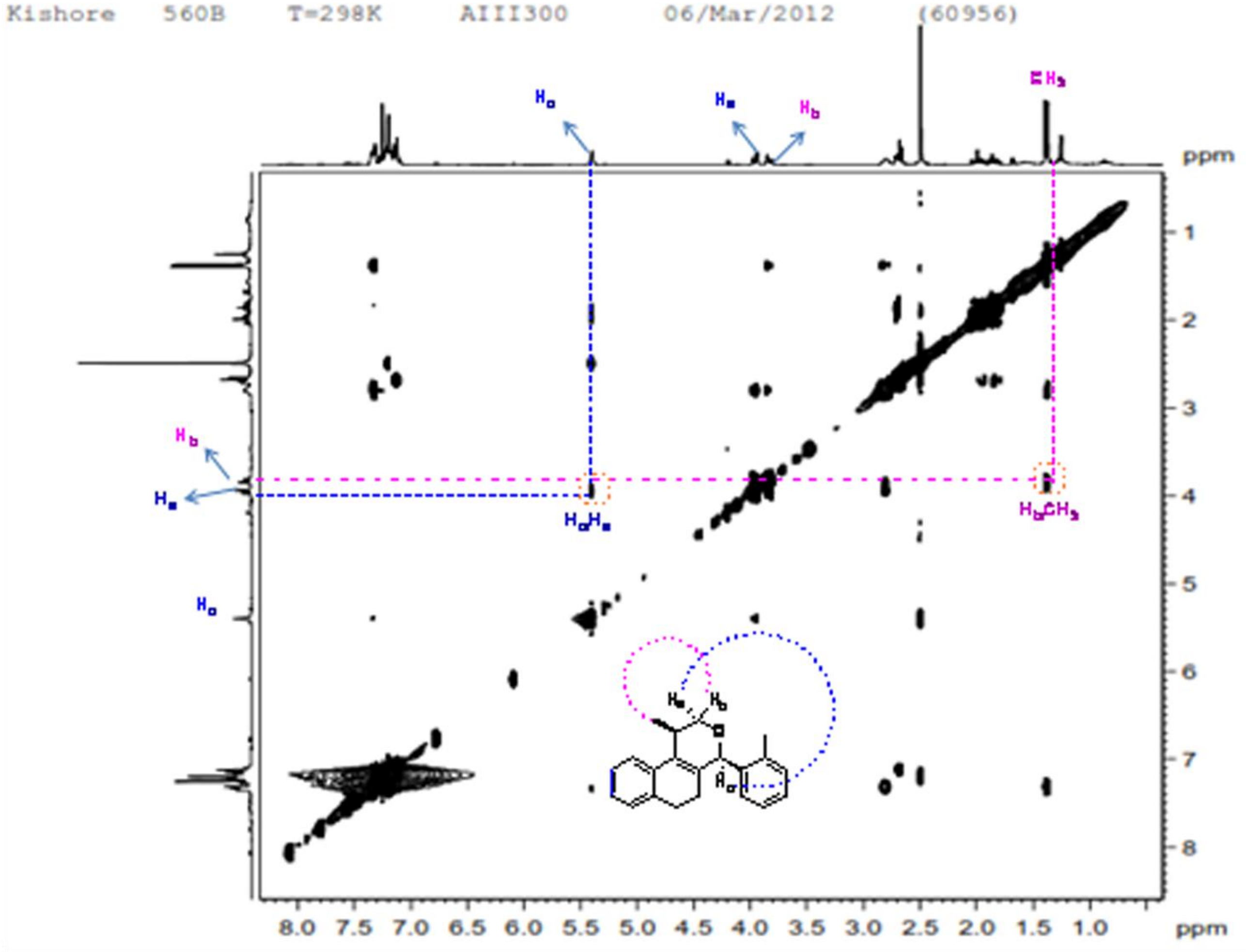

^{1}H-NMR of compound *trans*-**7f** (500 MHz, $CDCl_3$)

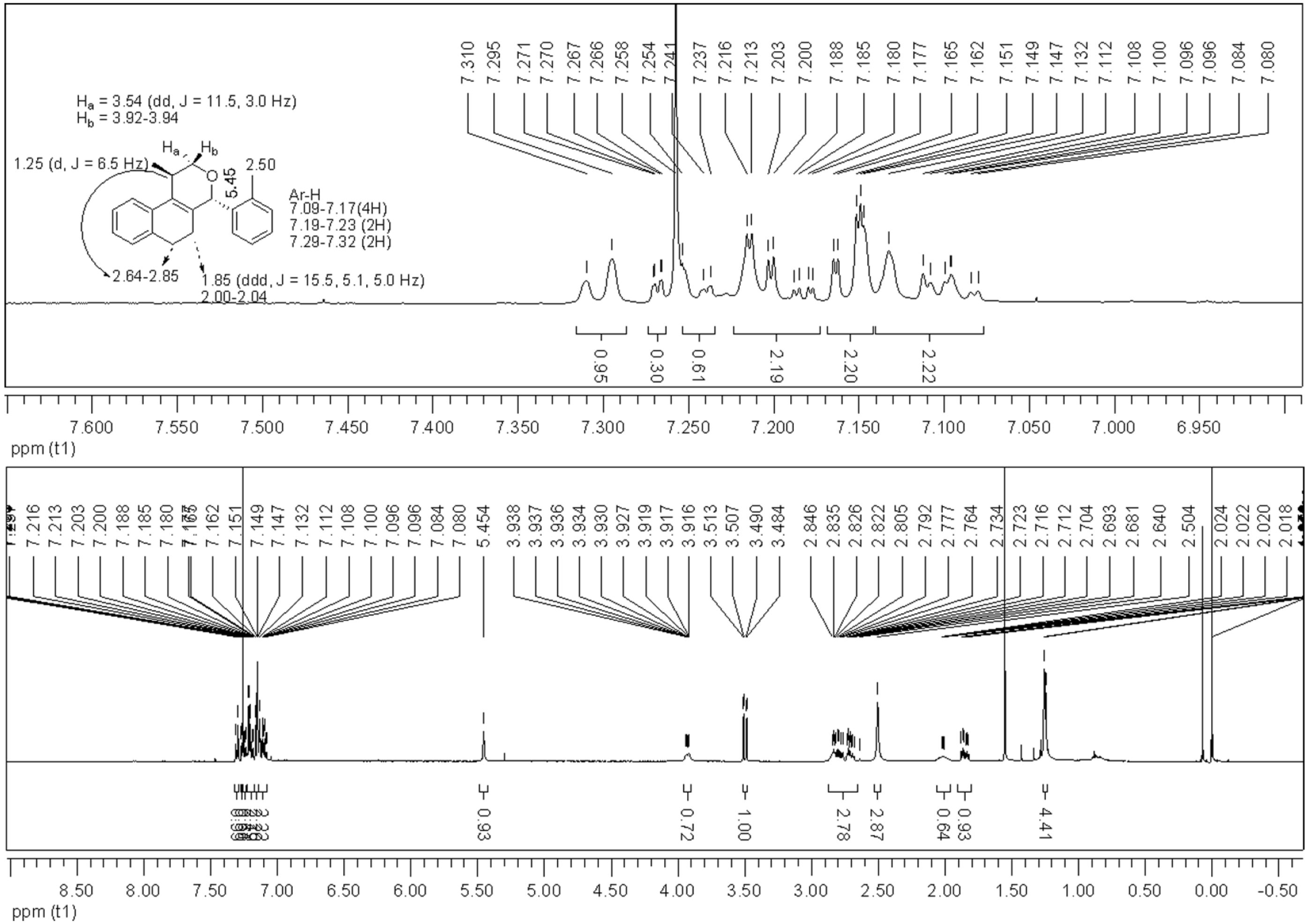

^{1}H-NMR of compound *trans*-**7f** (500 MHz, $CDCl_3$)

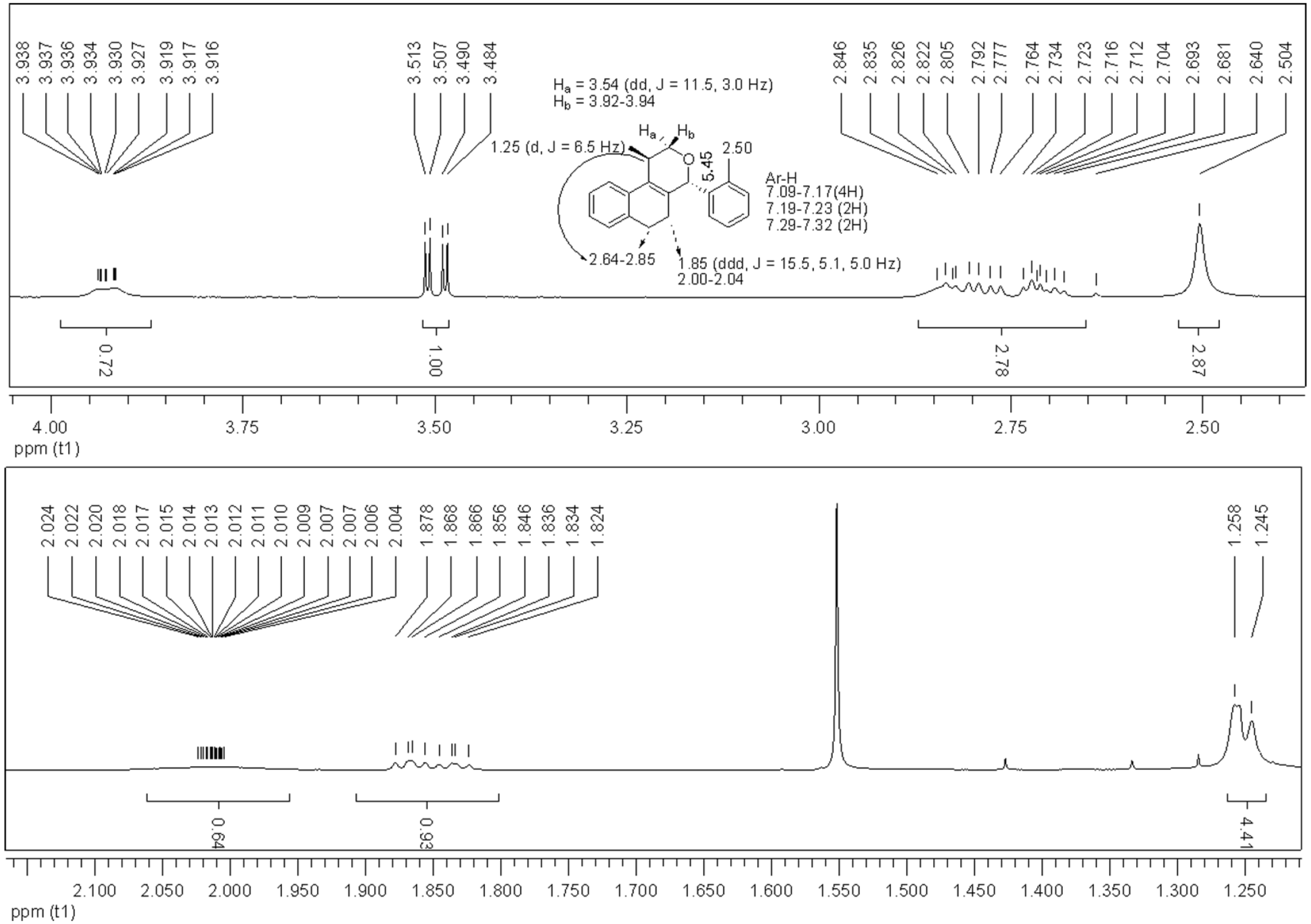

^{13}C-NMR of compound *trans*-**7f** (75 MHz, $CDCl_3$)

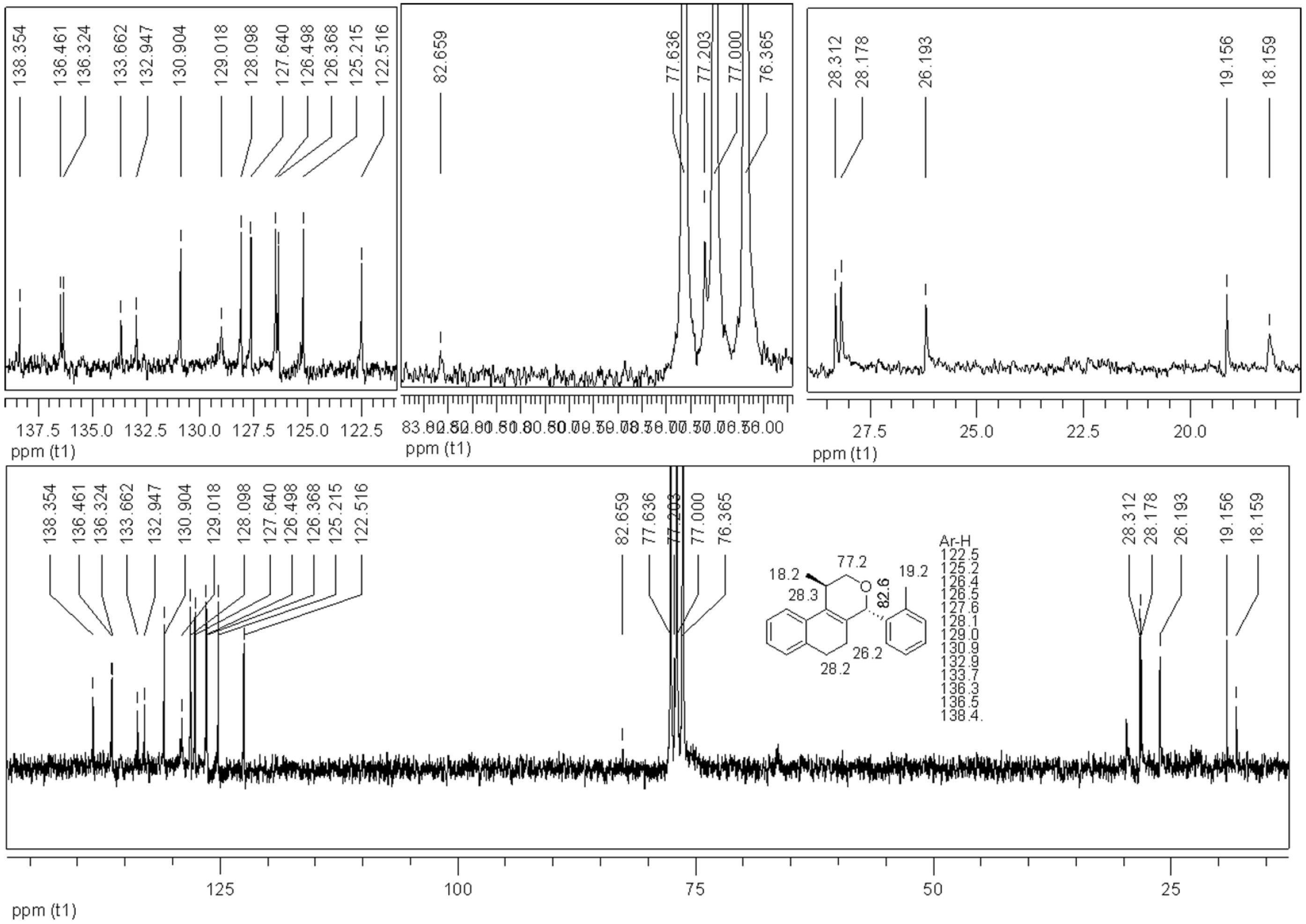

2D NOESY NMR of compound *trans*-**7f** (500 MHz, $CDCl_3$)

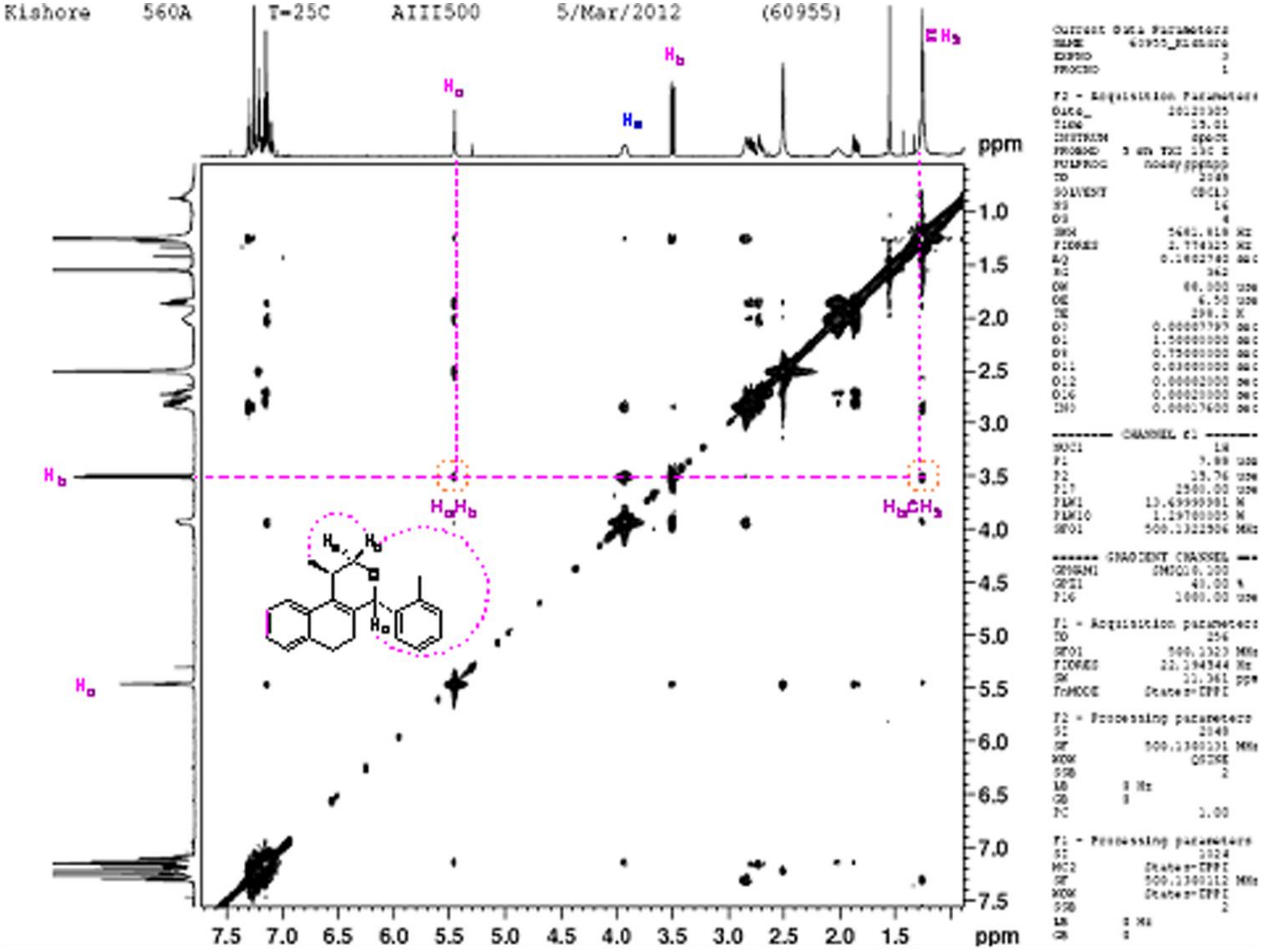

^{1}H-NMR of compound **12g** (200 MHz, $CDCl_3$)

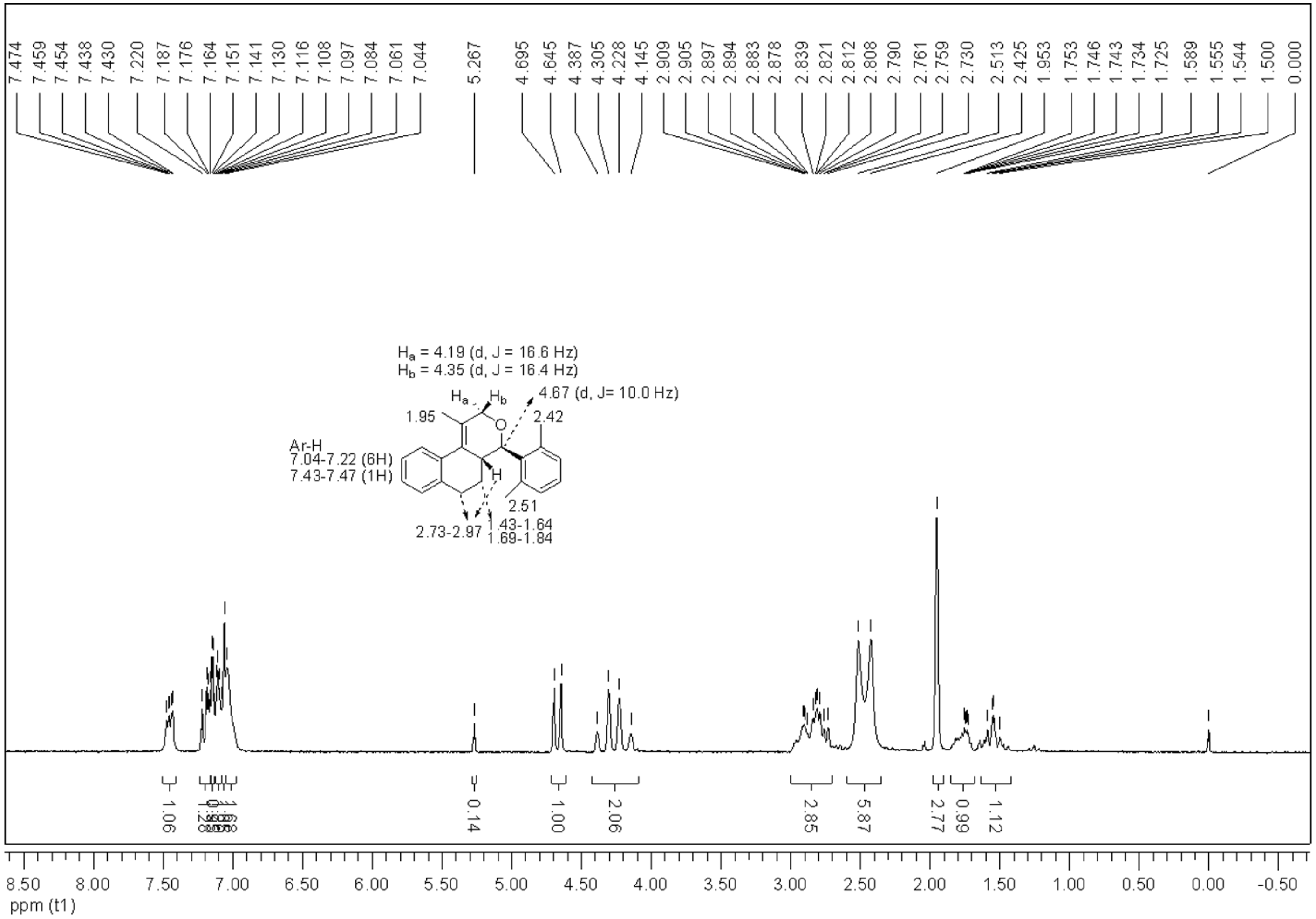

^{1}H-NMR of compound **12g** (200 MHz, $CDCl_3$)

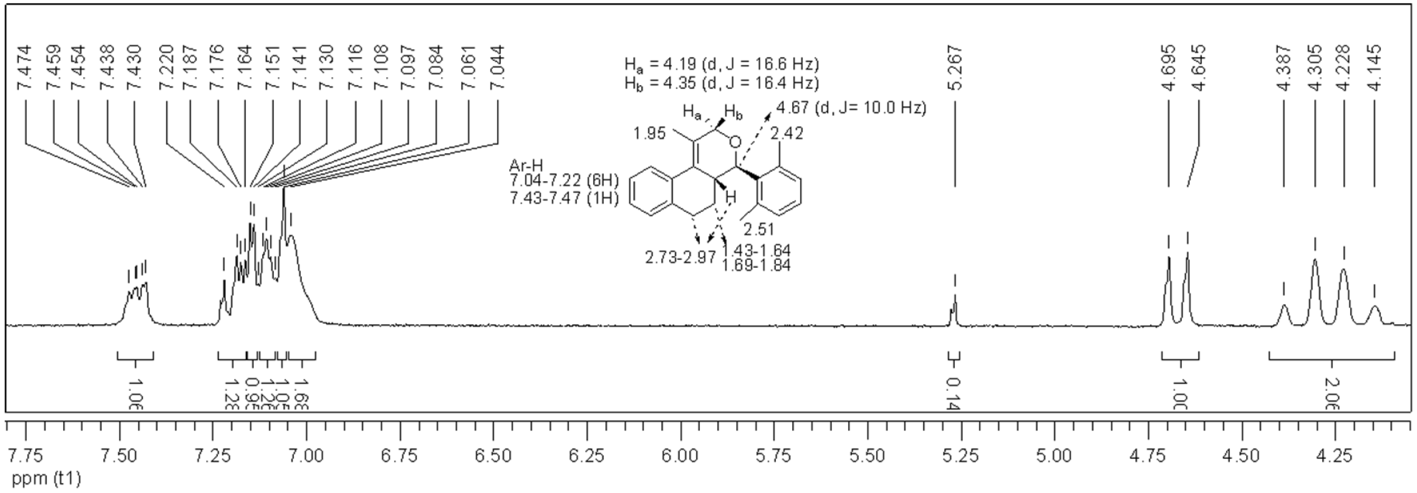

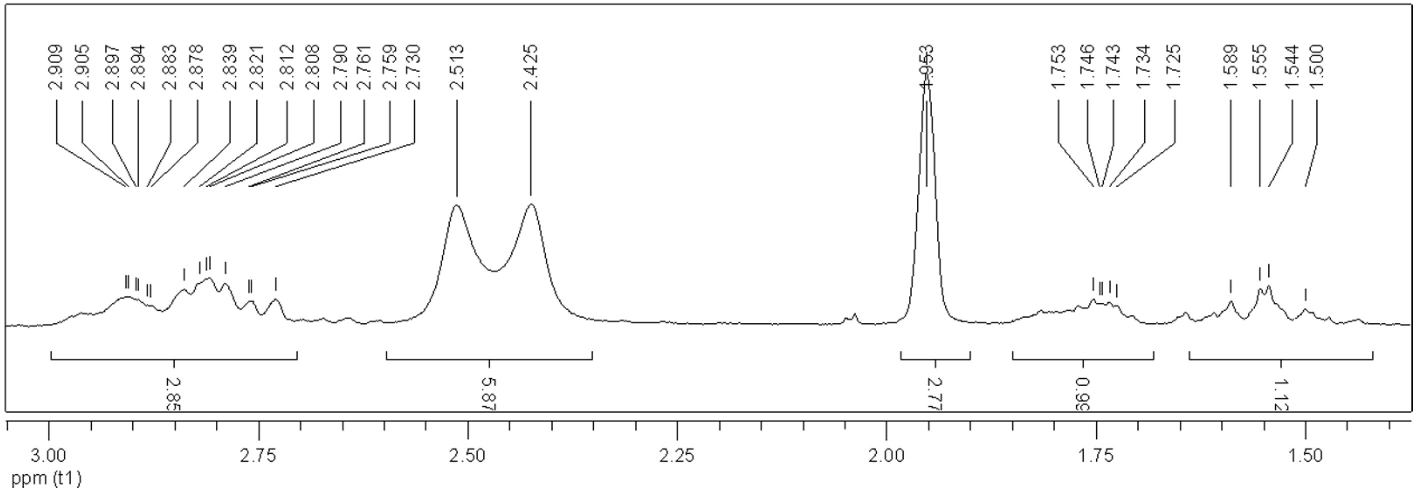

^{13}C-NMR of compound **12g** (75 MHz, $CDCl_3$)

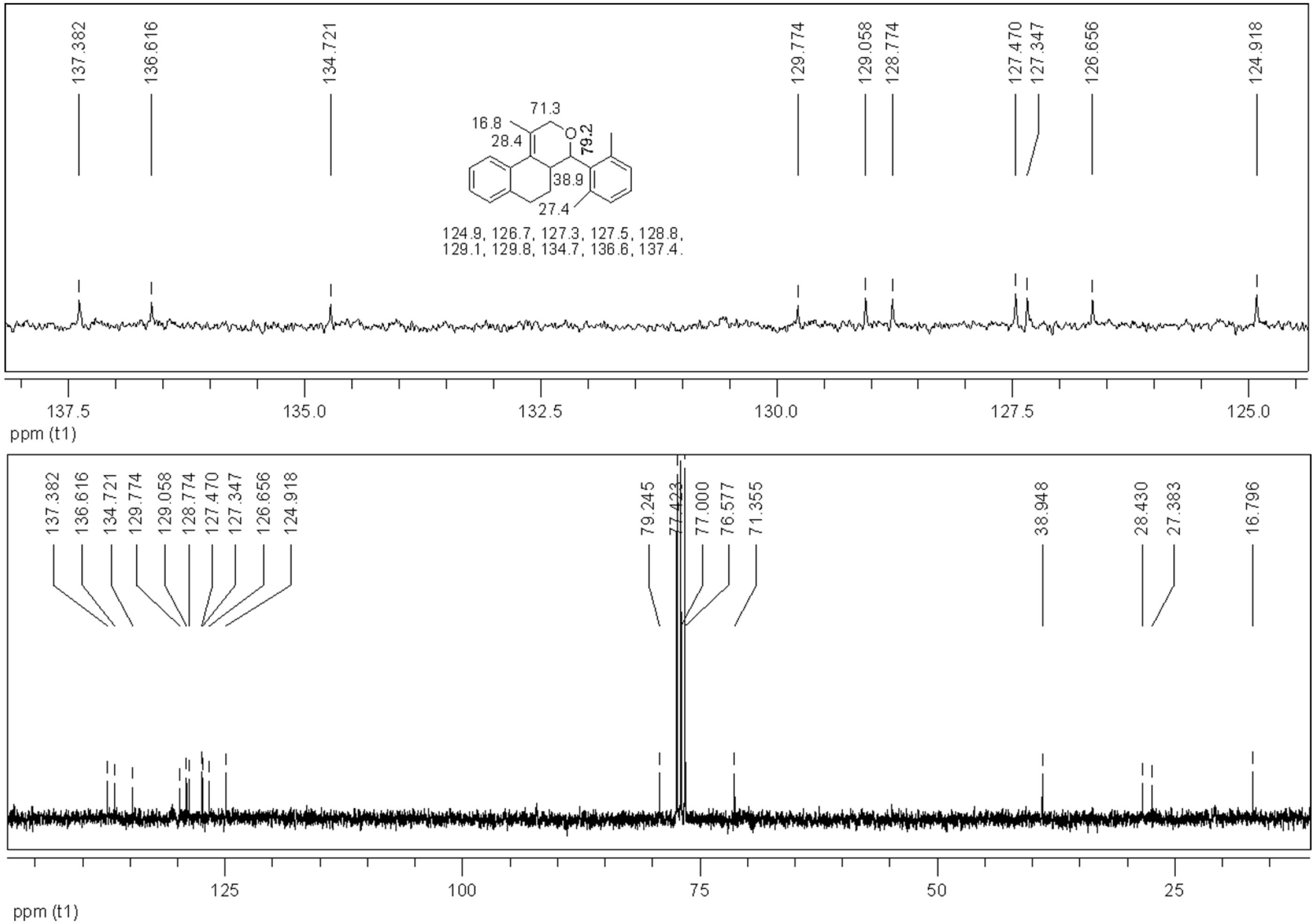

^{1}H-NMR of compounds *cis*-**7g** and *trans*-**7g** (500 MHz, $CDCl_3$)

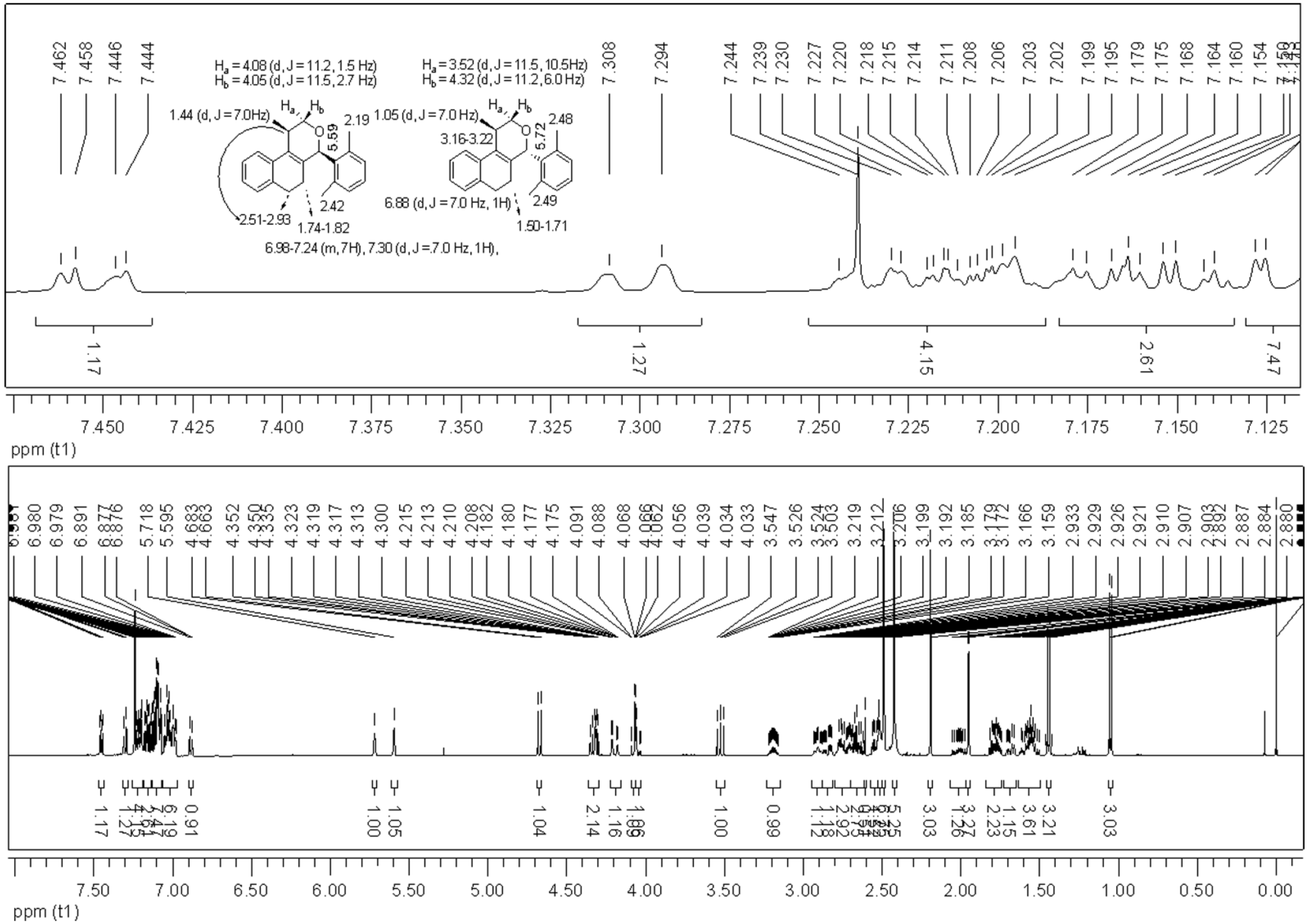

^{1}H-NMR of compounds *cis*-**7g** and *trans*-**7g** (300 MHz, $CDCl_3$)

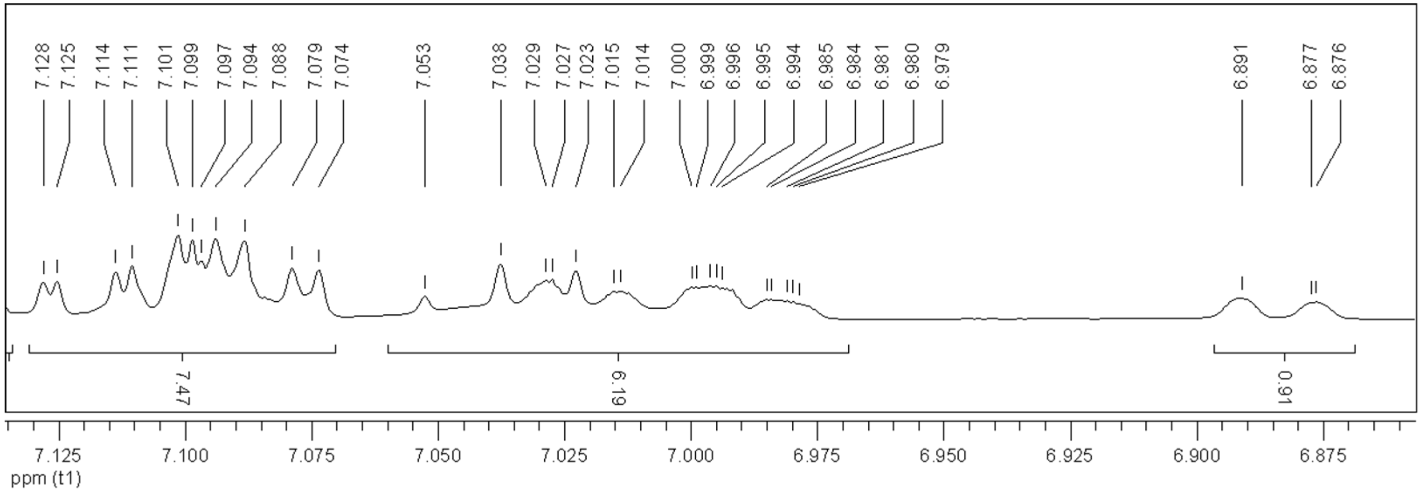

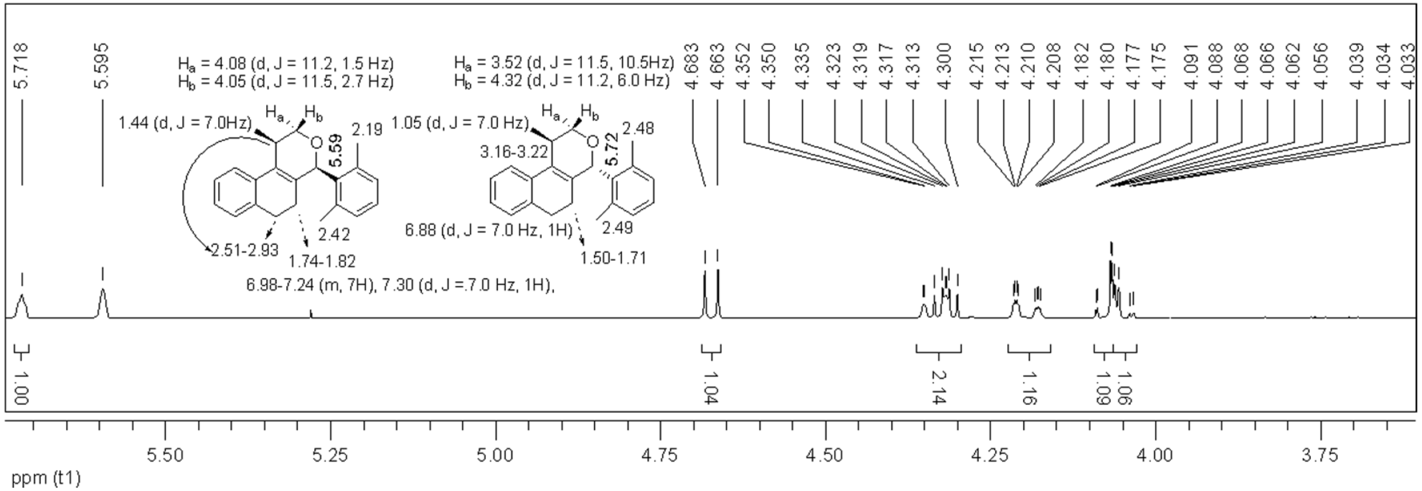

^{1}H-NMR of compounds *cis*-**7g** and *trans*-**7g** (500 MHz, $CDCl_3$)

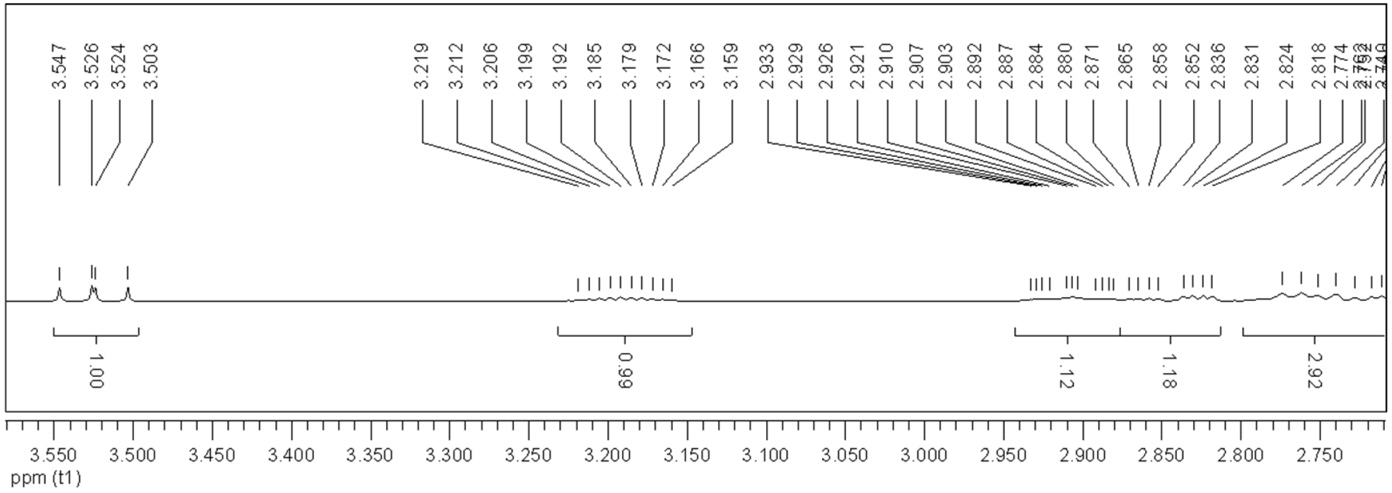

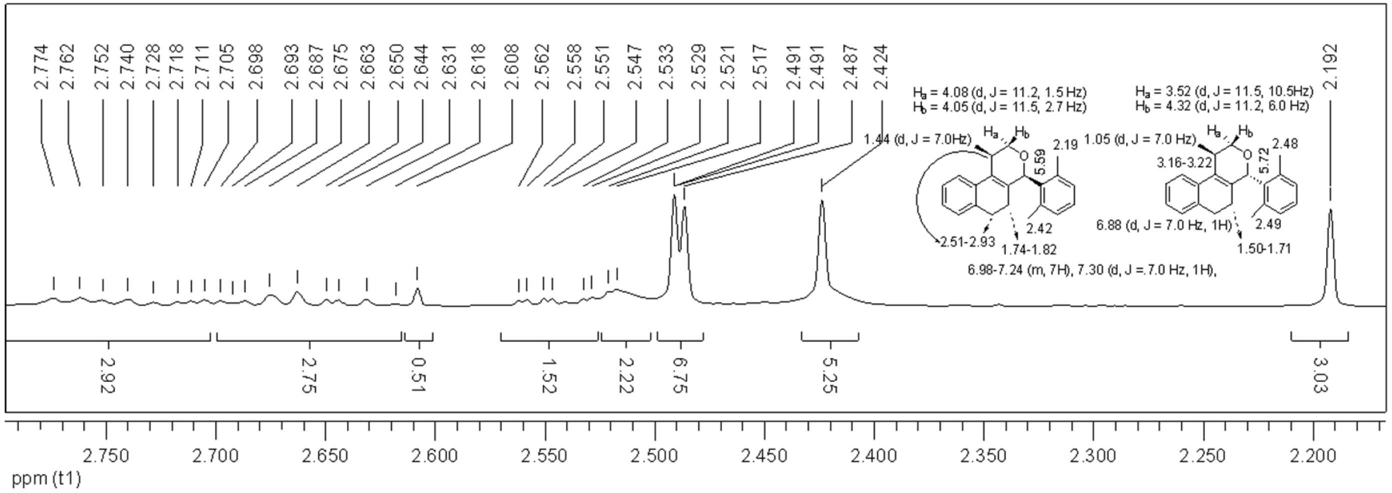

^{1}H-NMR of compounds *cis*-**7g** and *trans*-**7g** (500 MHz, $CDCl_3$)

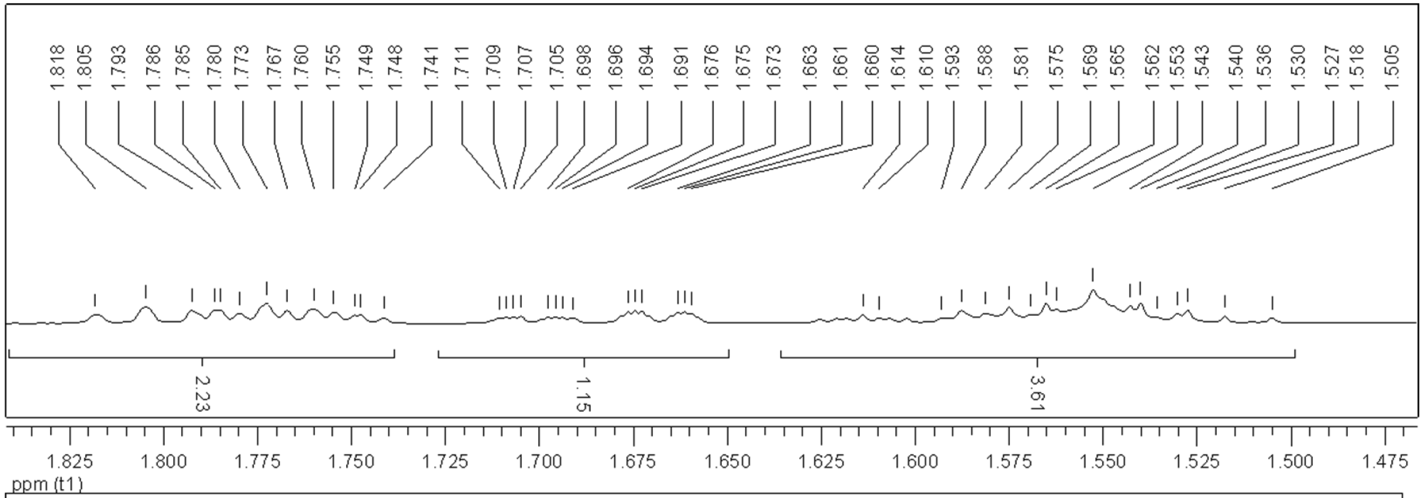

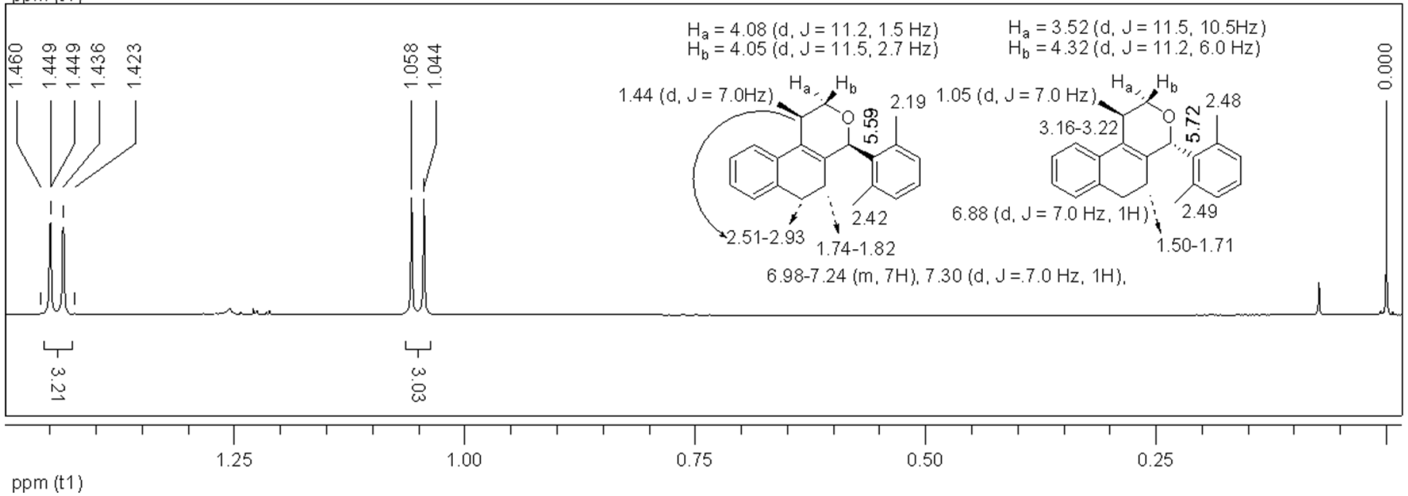

^{13}C-NMR of compounds *cis*-**7g** and *trans*-**7g** (50 MHz, $CDCl_3$)

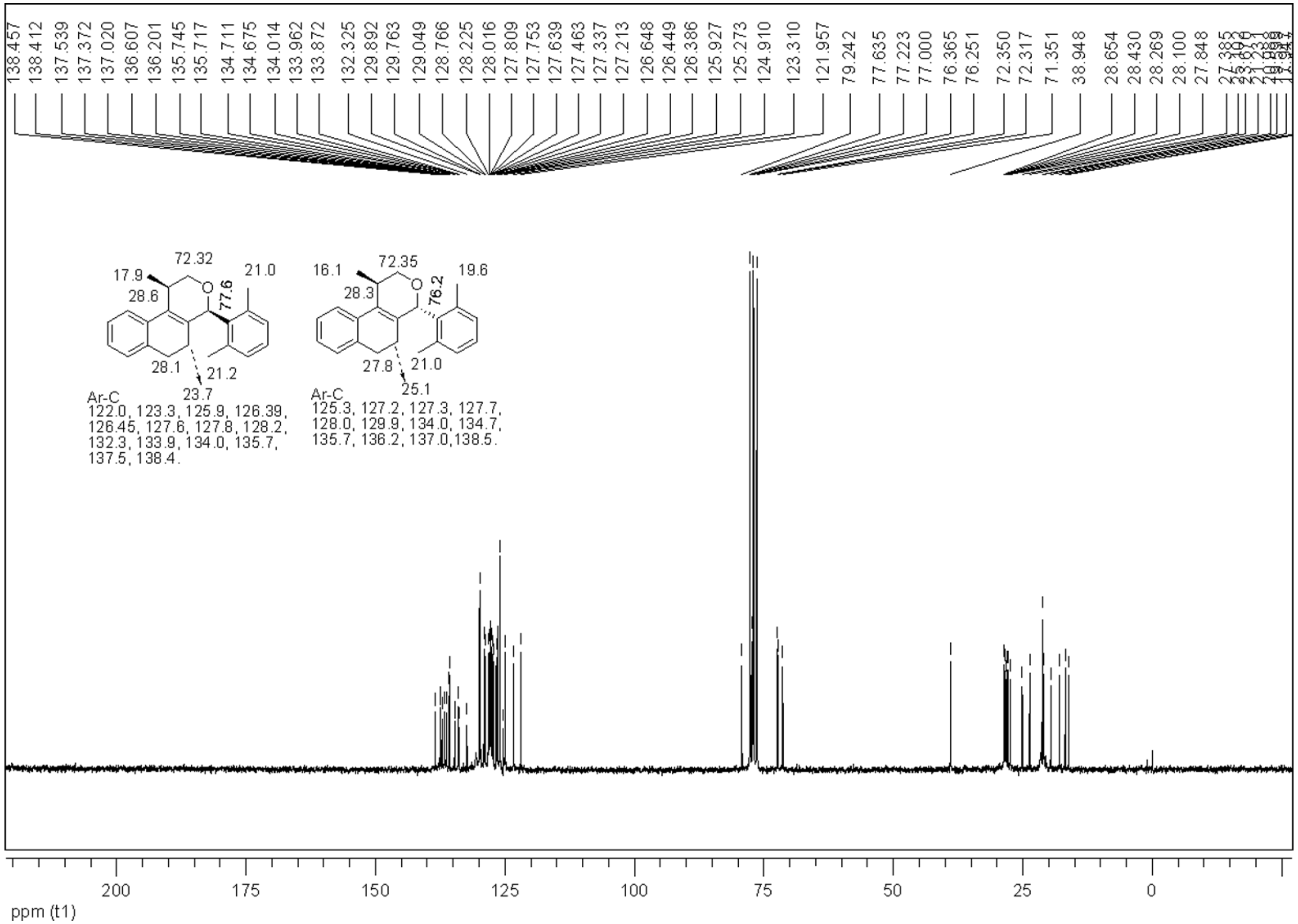

^{13}C-NMR of compounds *cis*-**7g** and *trans*-**7g** (50 MHz, $CDCl_3$)

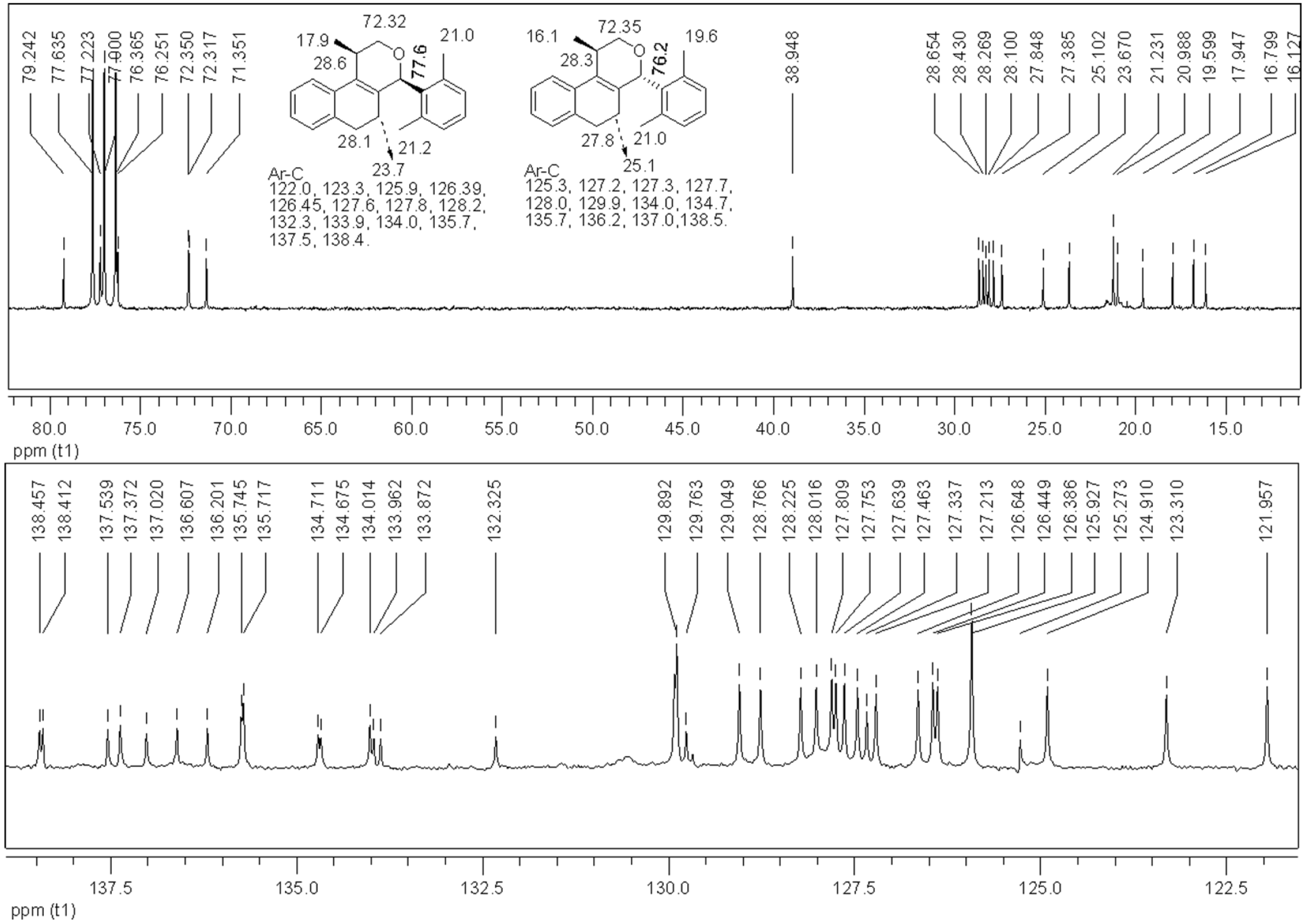

2D NOESY NMR of compound *cis*-**7g** (500 MHz, $CDCl_3$)

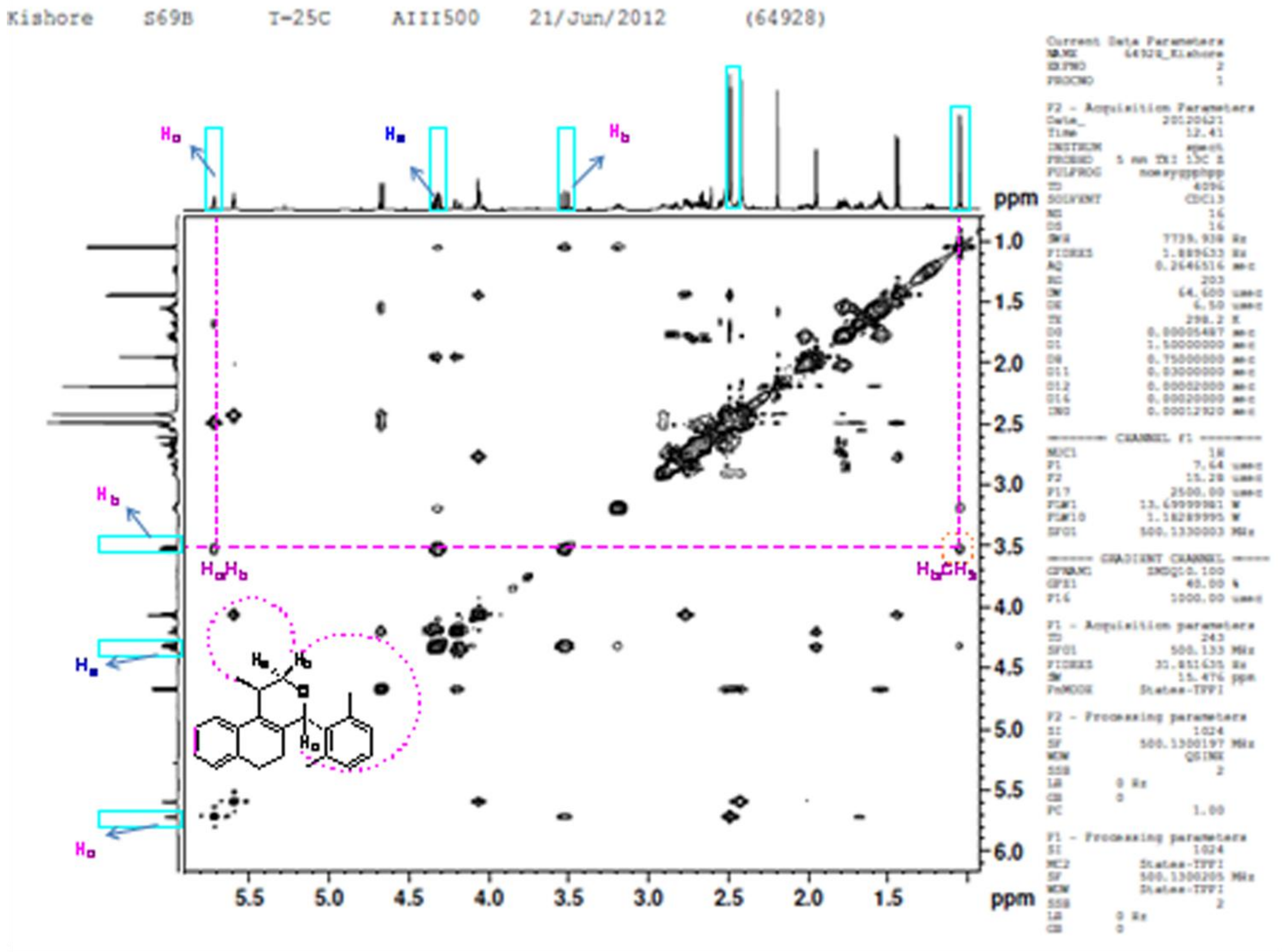

^{1}H-NMR of compounds *cis*-**7i** and *trans*-**7i** (200 MHz, $CDCl_3$)

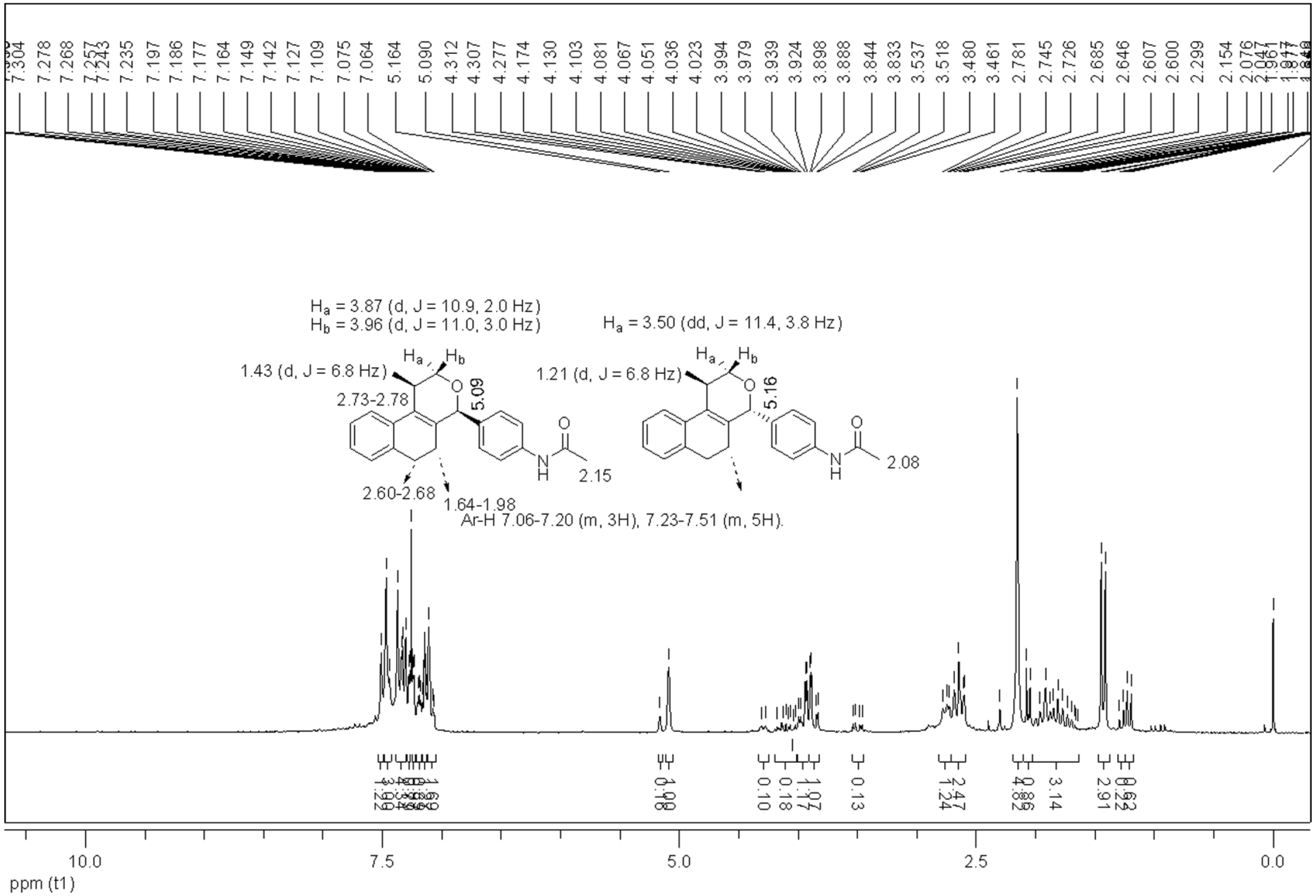

^{1}H-NMR of compounds *cis*-**7i** and *trans*-**7i** (200 MHz, $CDCl_3$)

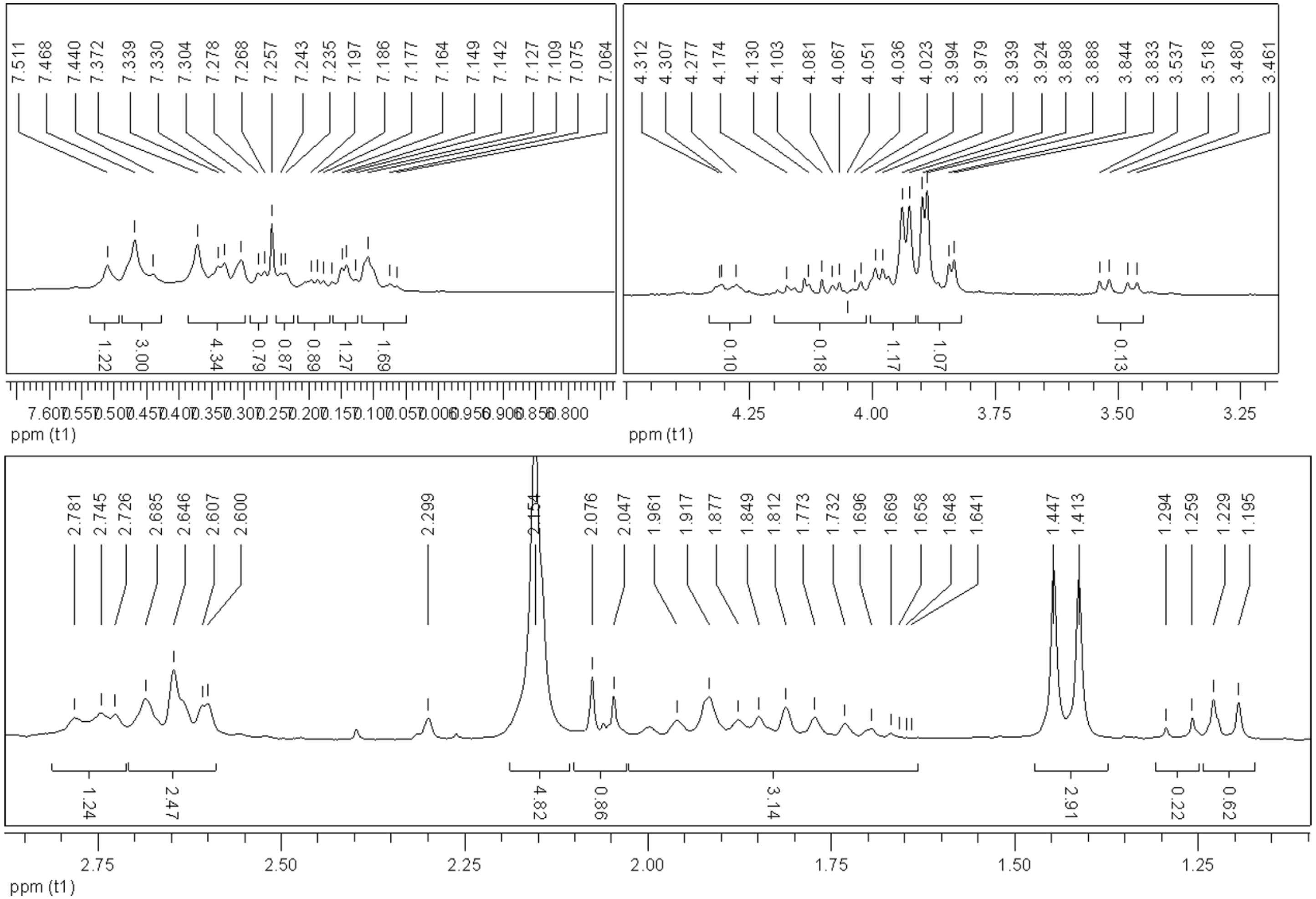

^{13}C-NMR of compounds *cis*-**7i** and *trans*-**7i** (75 MHz, $CDCl_3$)

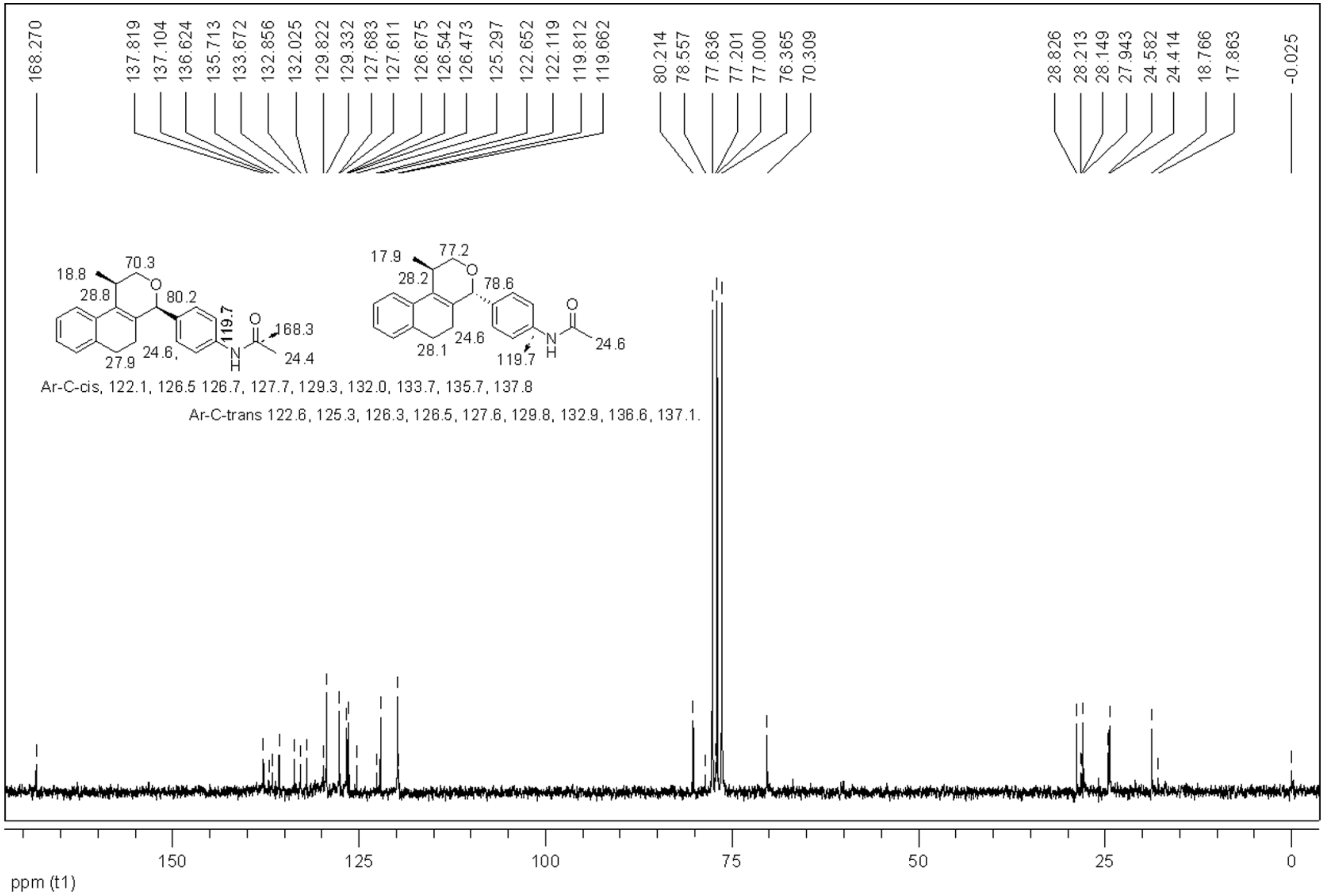

^{13}C-NMR of compounds *cis*-**7i** and *trans*-**7i** (75 MHz, $CDCl_3$)

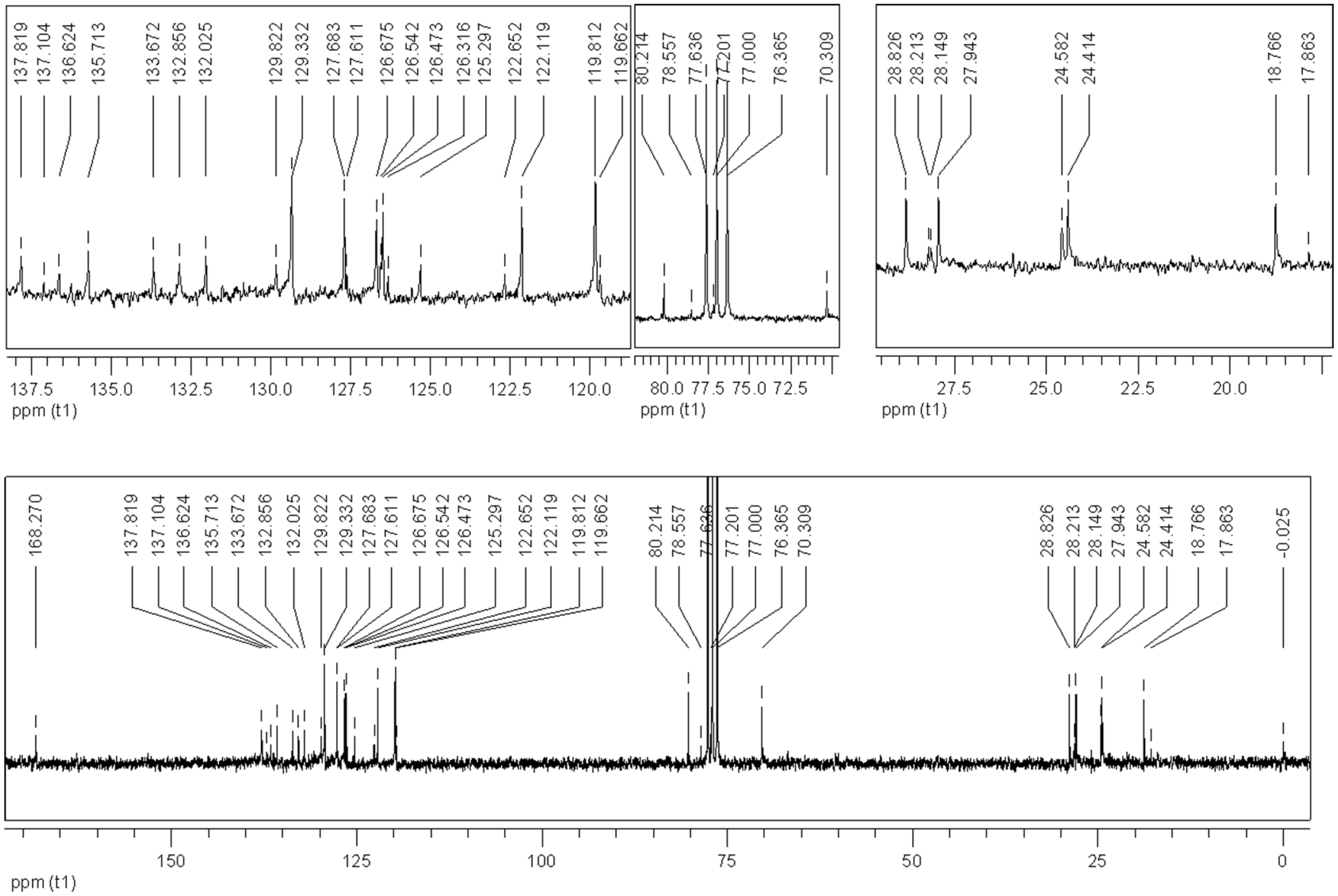

^{1}H-NMR of compound *cis*-**7j** (200 MHz, $CDCl_3$)

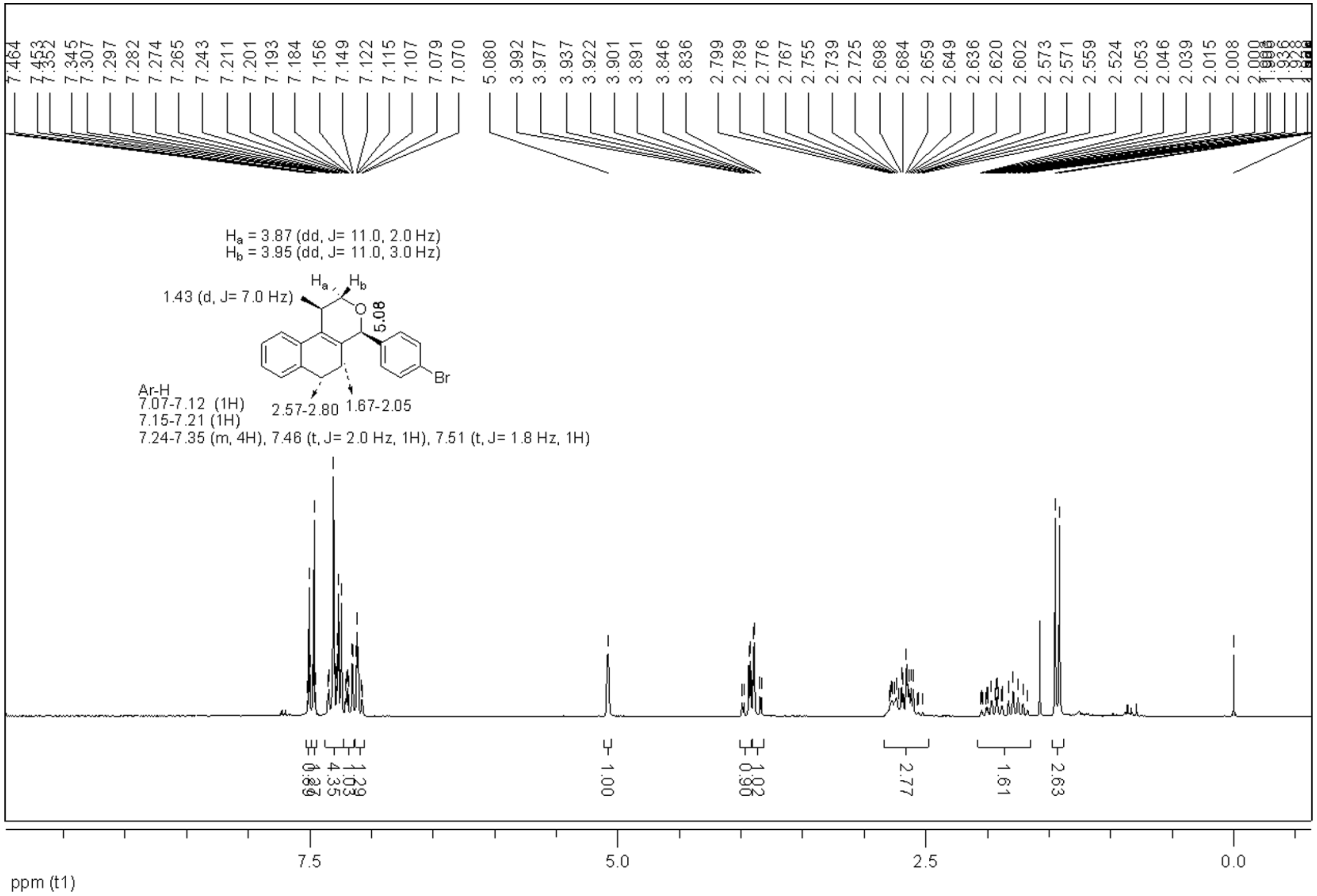

^{13}C-NMR of compound *cis*-**7j** (50 MHz, $CDCl_3$)

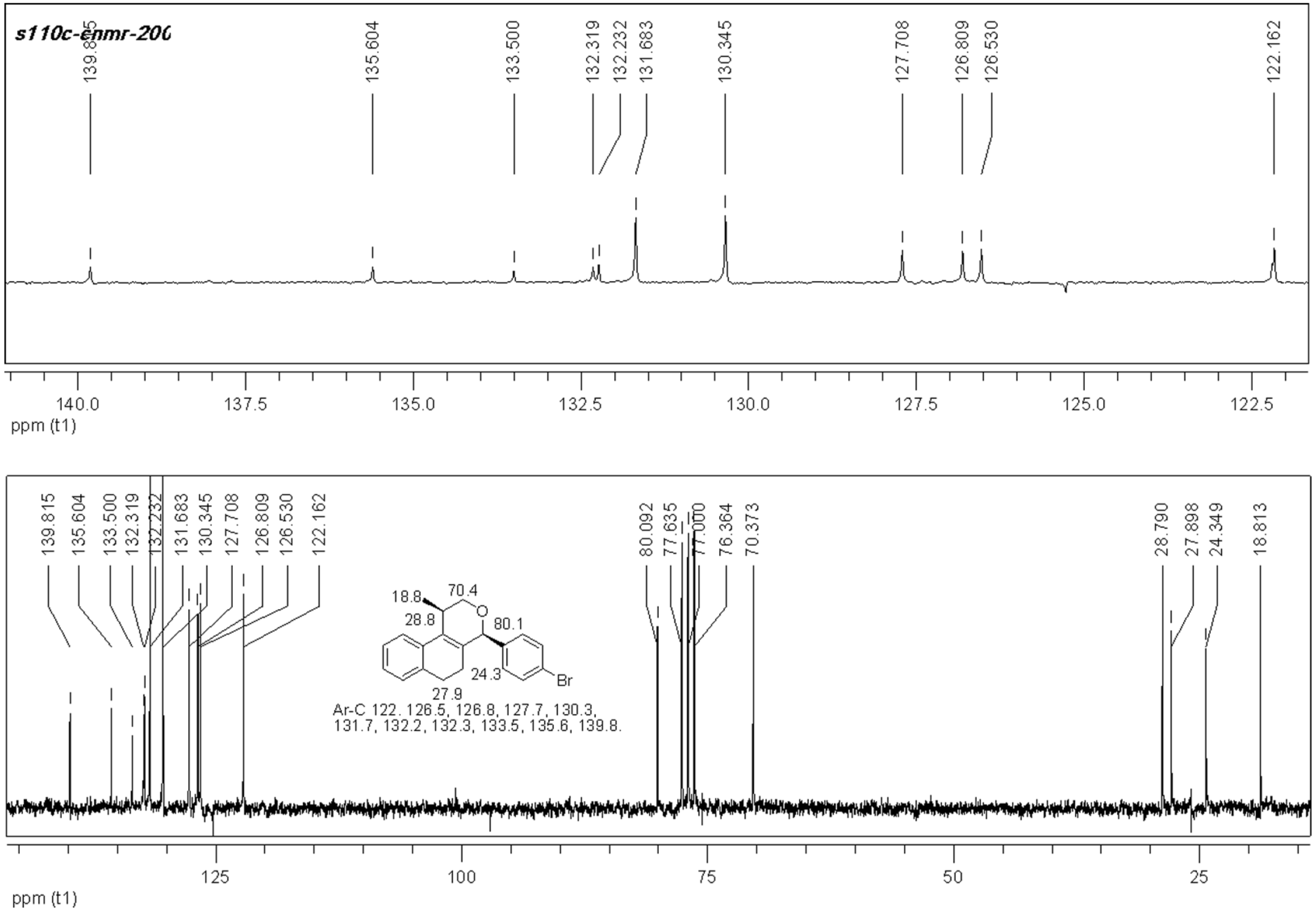

^{1}H-NMR of compounds **12j** and *trans*-**7j** (200 MHz, $CDCl_3$)

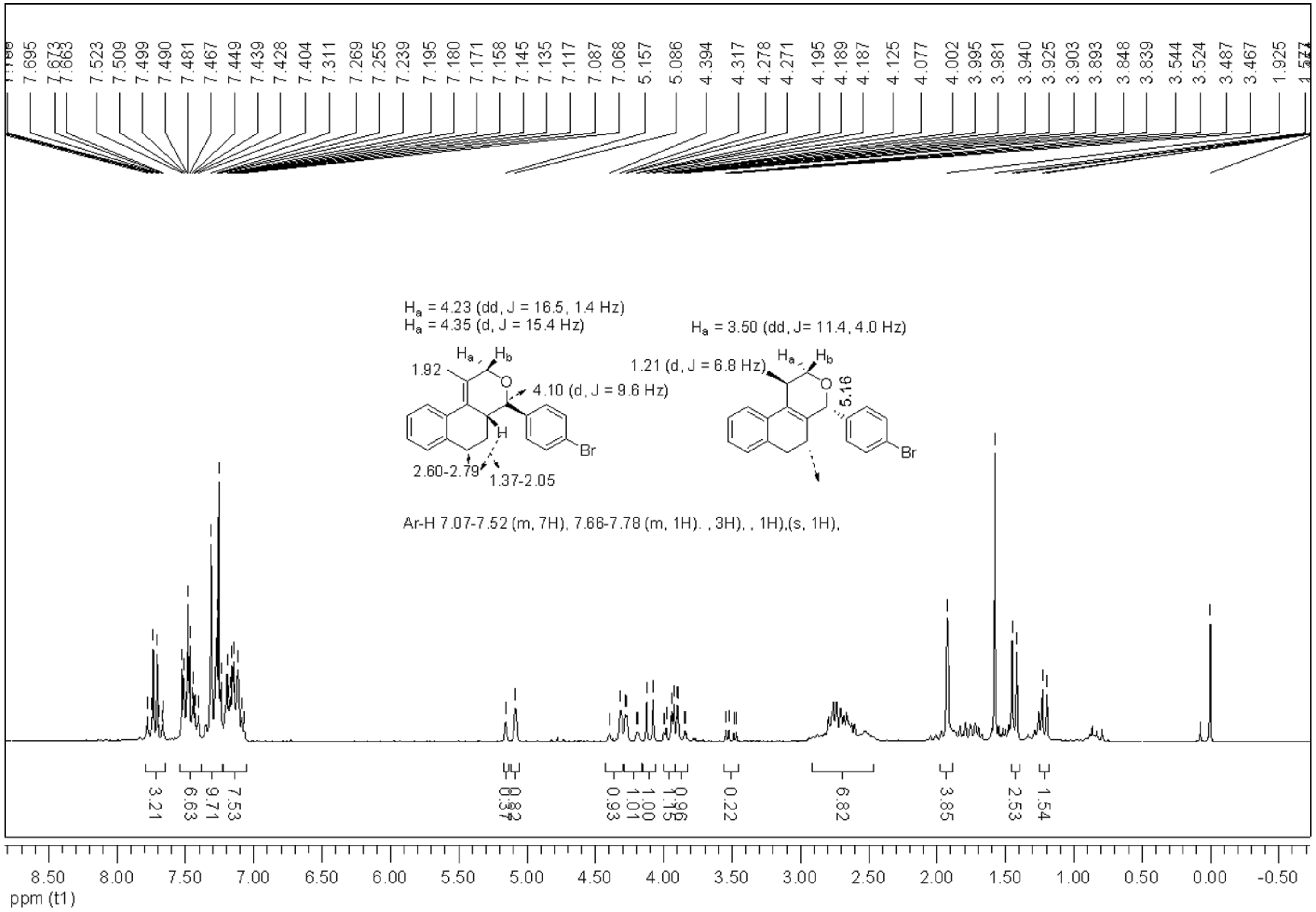

^{1}H-NMR of compounds **12j** and *trans*-**7j** (200 MHz, $CDCl_3$)

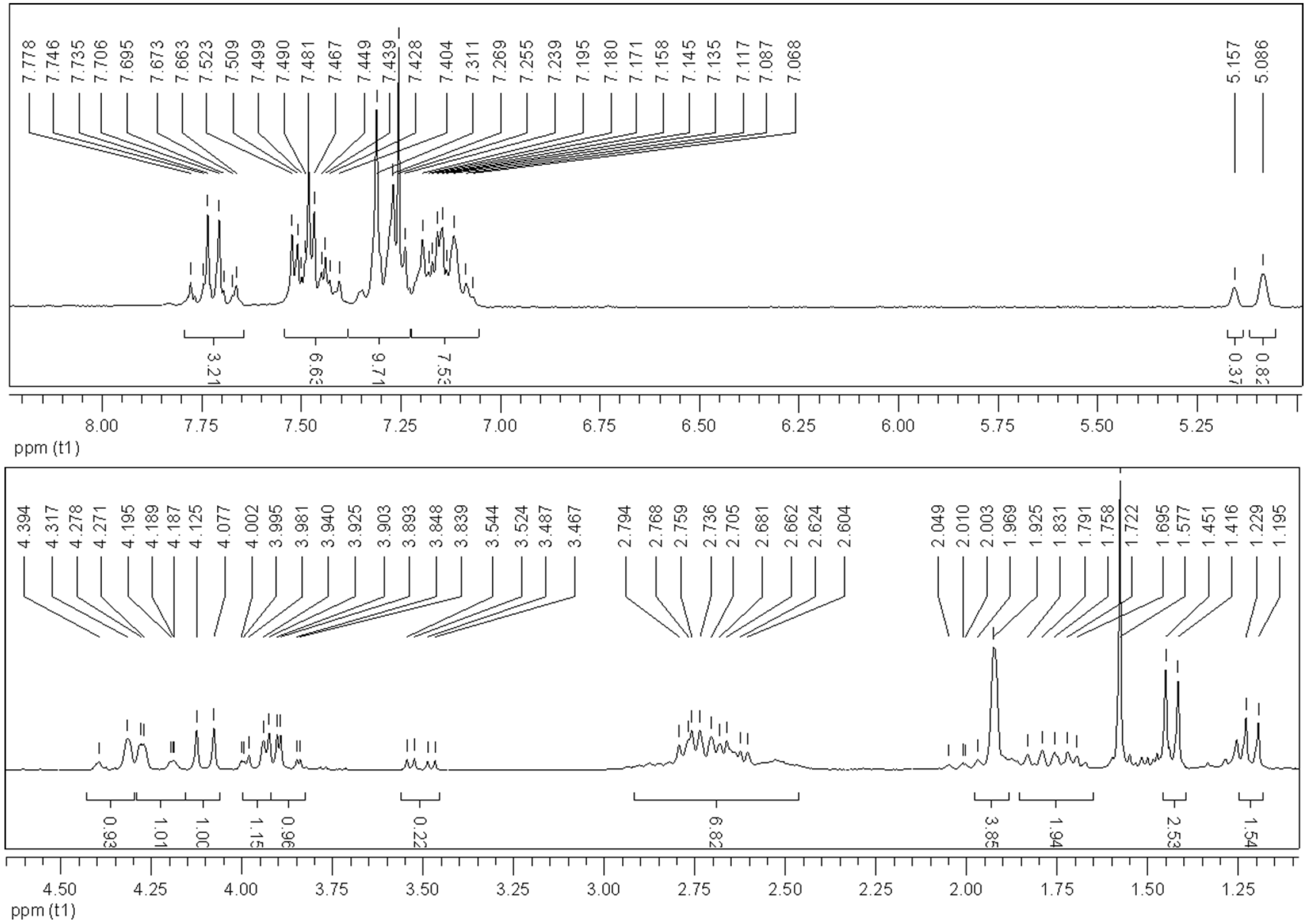

^{13}C-NMR of compounds **12j** and *trans*-**7j** (50 MHz, $CDCl_3$)

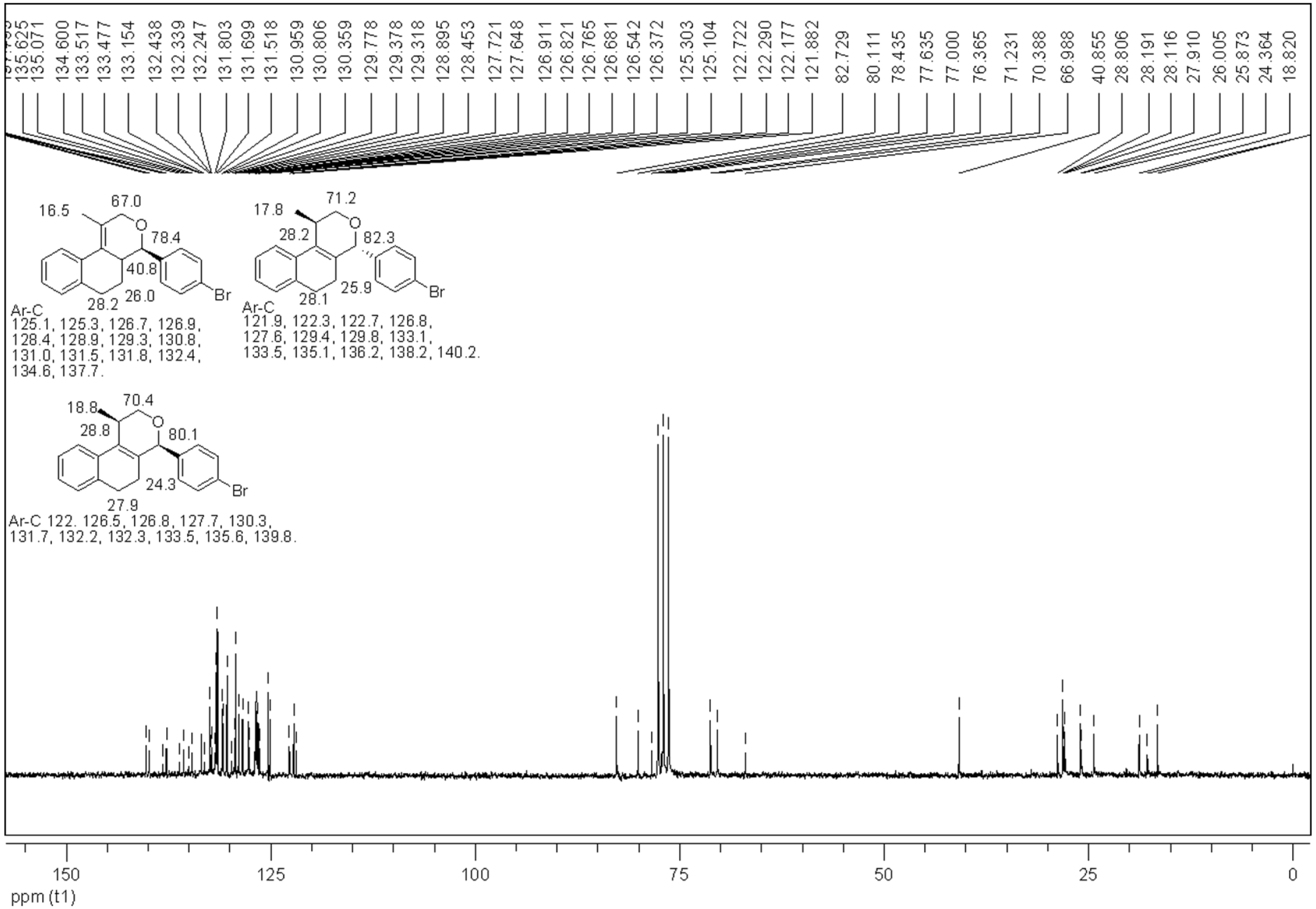

^{13}C-NMR of compounds **12j** and *trans*-**7j** (50 MHz, $CDCl_3$)

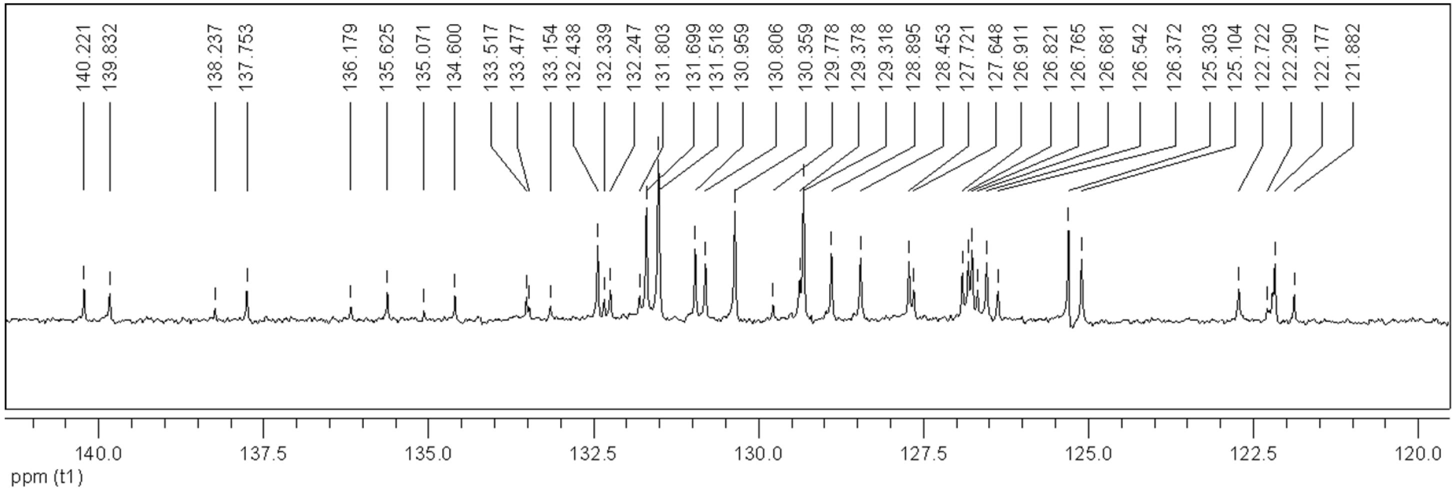

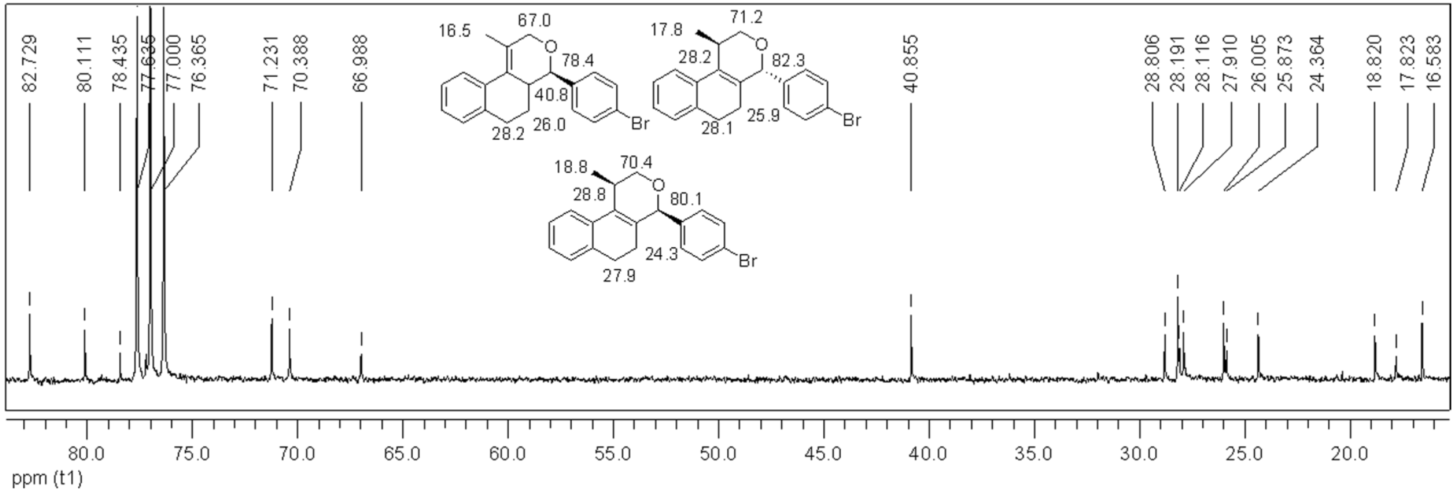

^{1}H-NMR of compound *cis*-**7k** (500 MHz, $CDCl_3$)

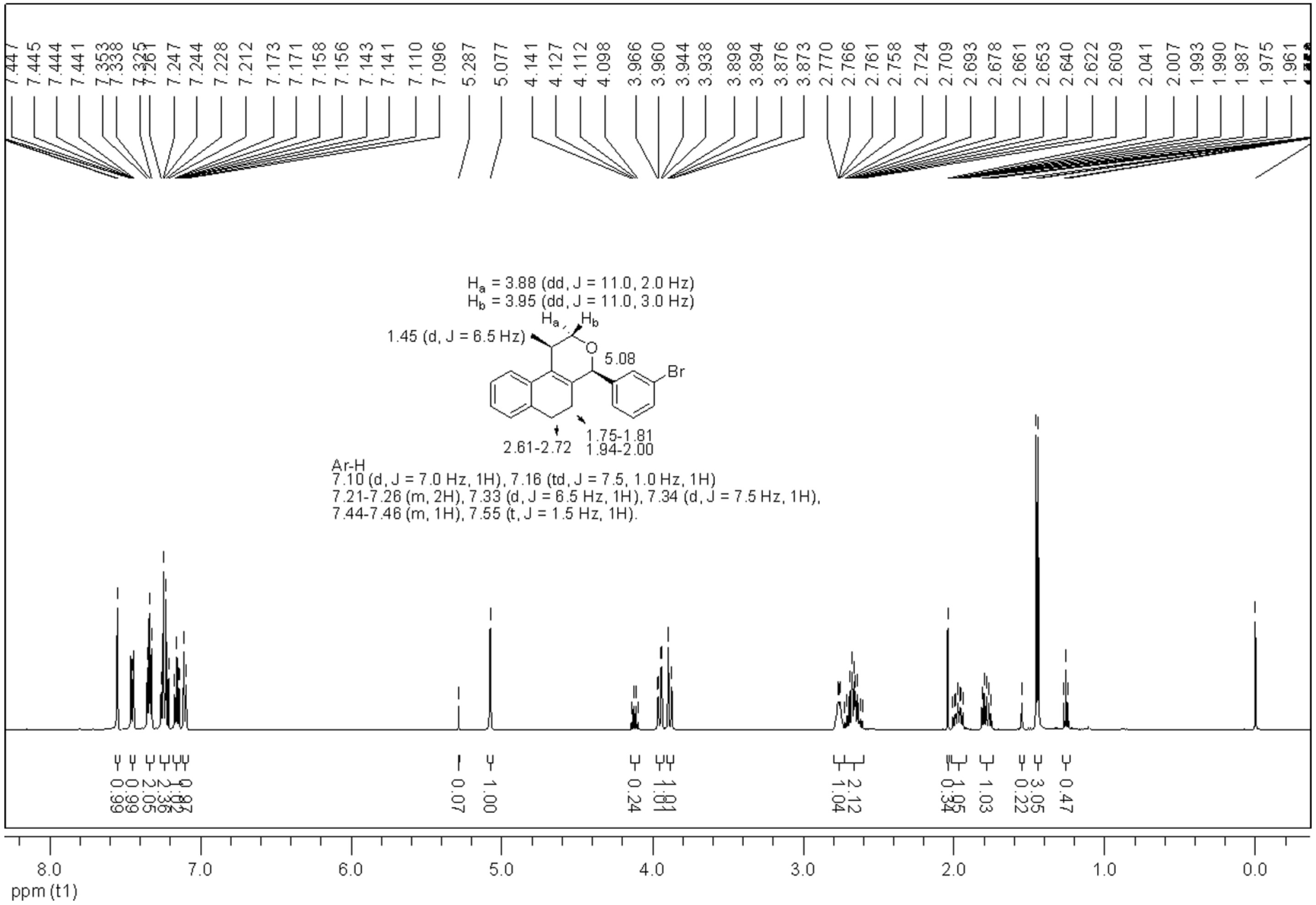

^{1}H-NMR of compound *cis*-**7k** (300 MHz, $CDCl_3$)

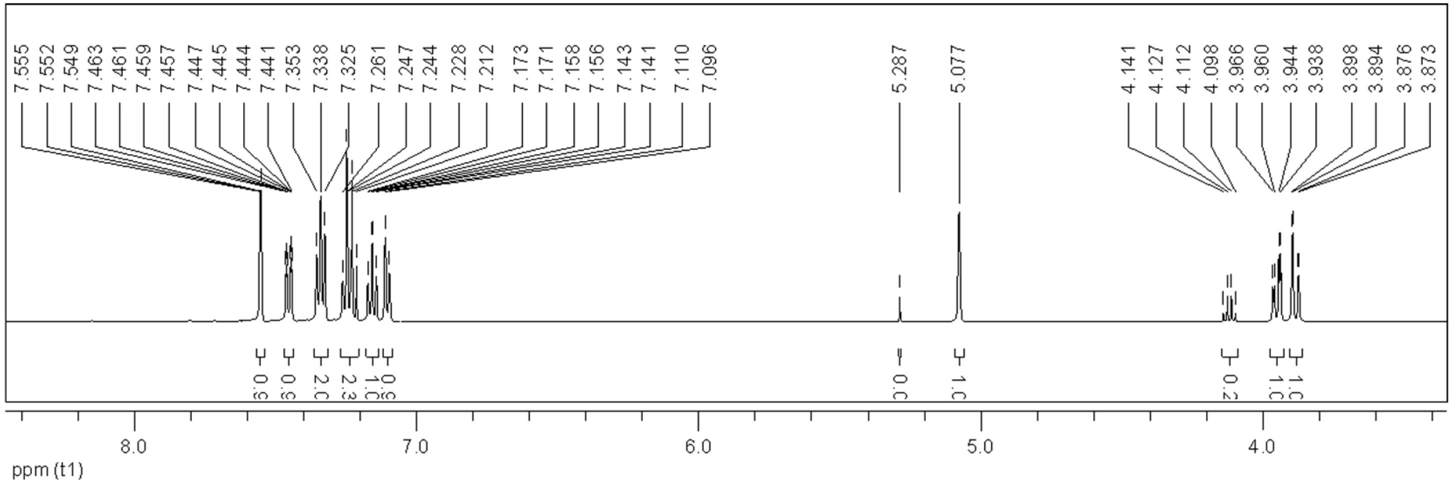

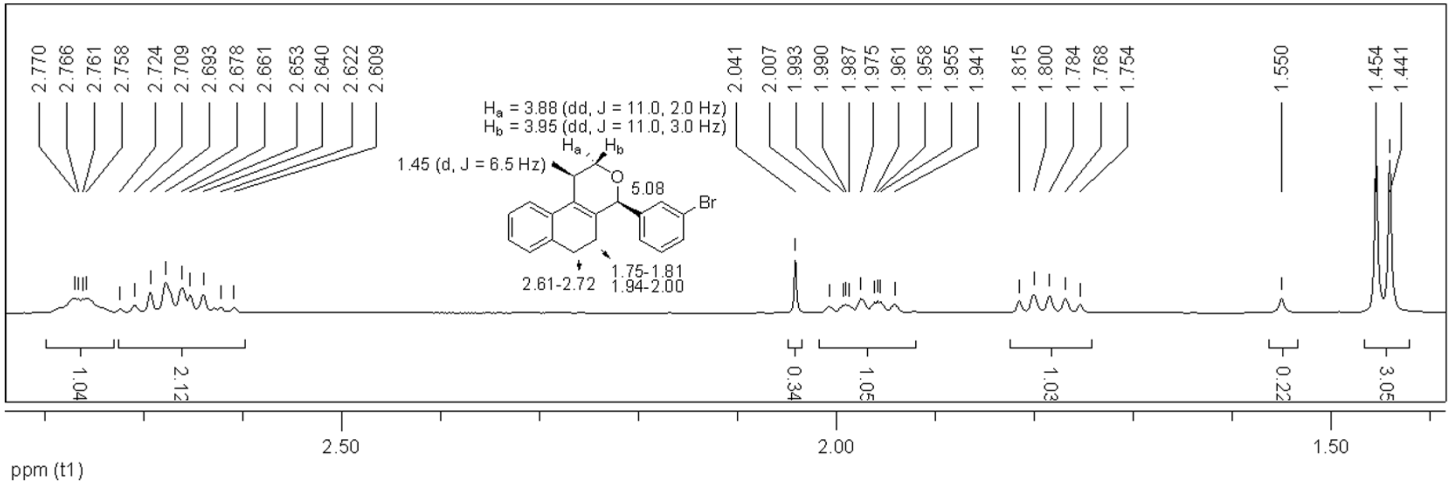

^{13}C-NMR of compound *cis*-**7k** (75 MHz, $CDCl_3$)

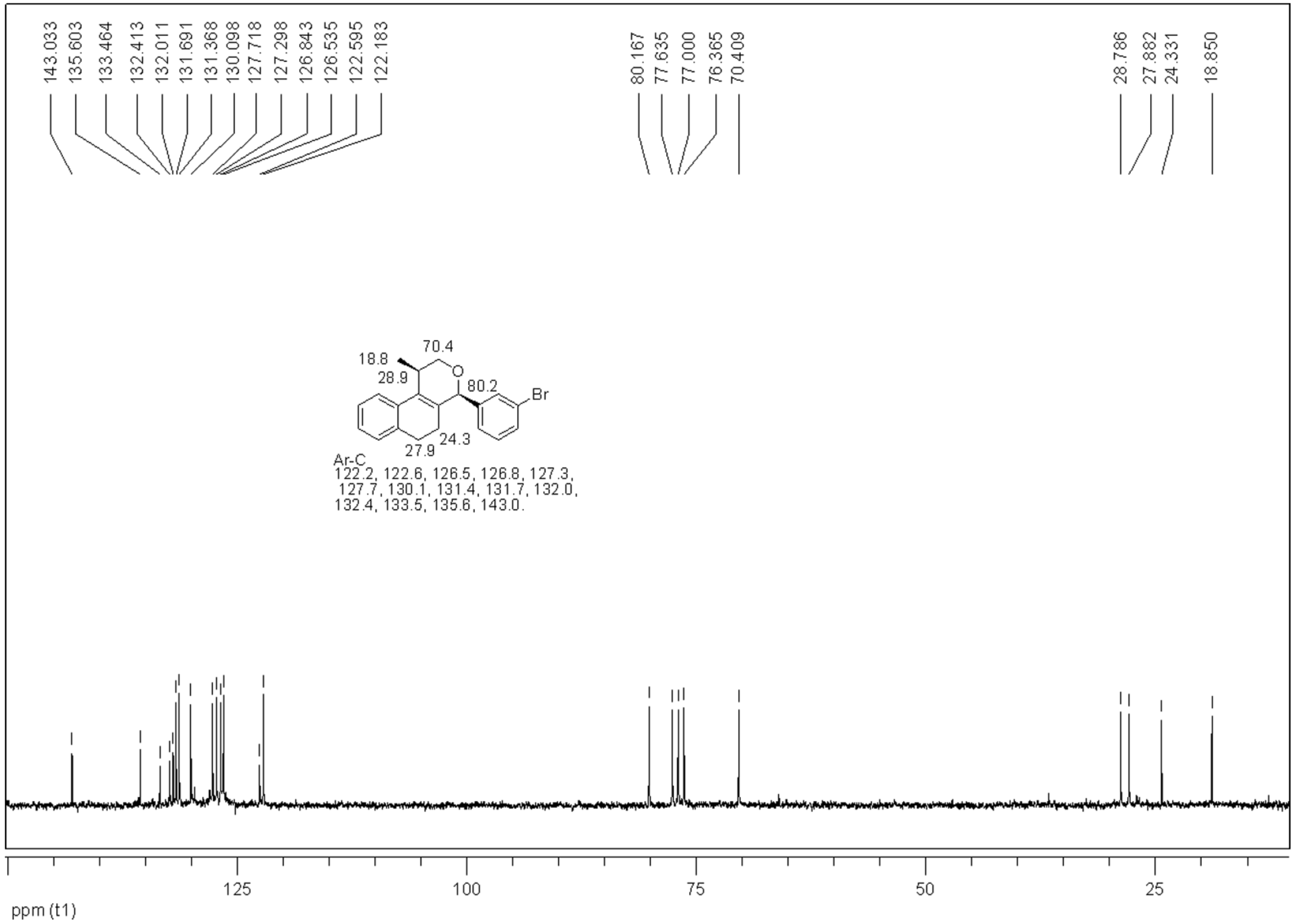

^{13}C-NMR of compound *cis*-**7k** (75 MHz, $CDCl_3$)

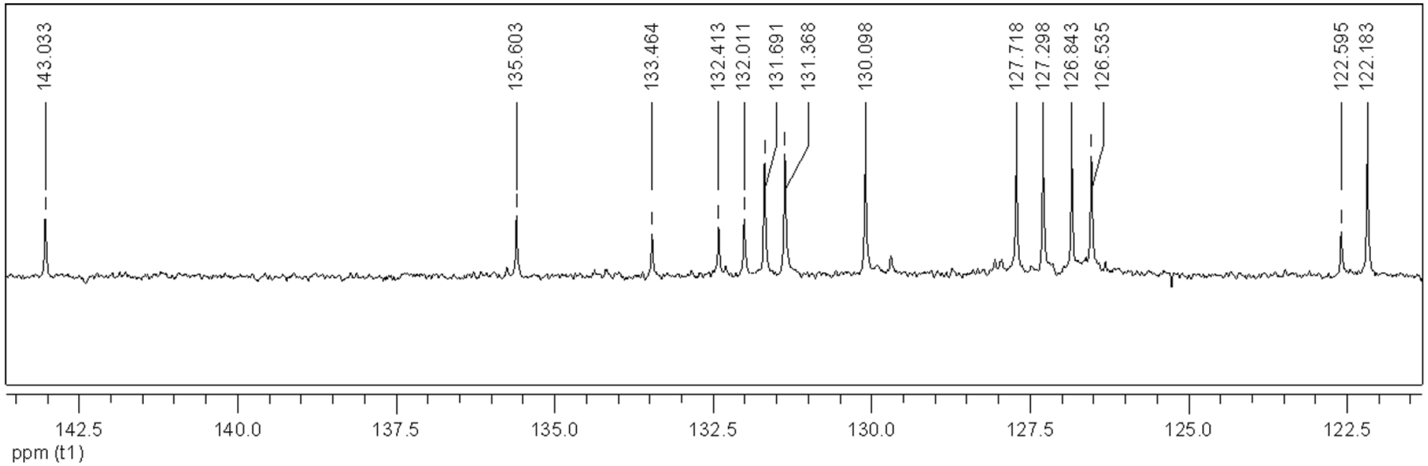

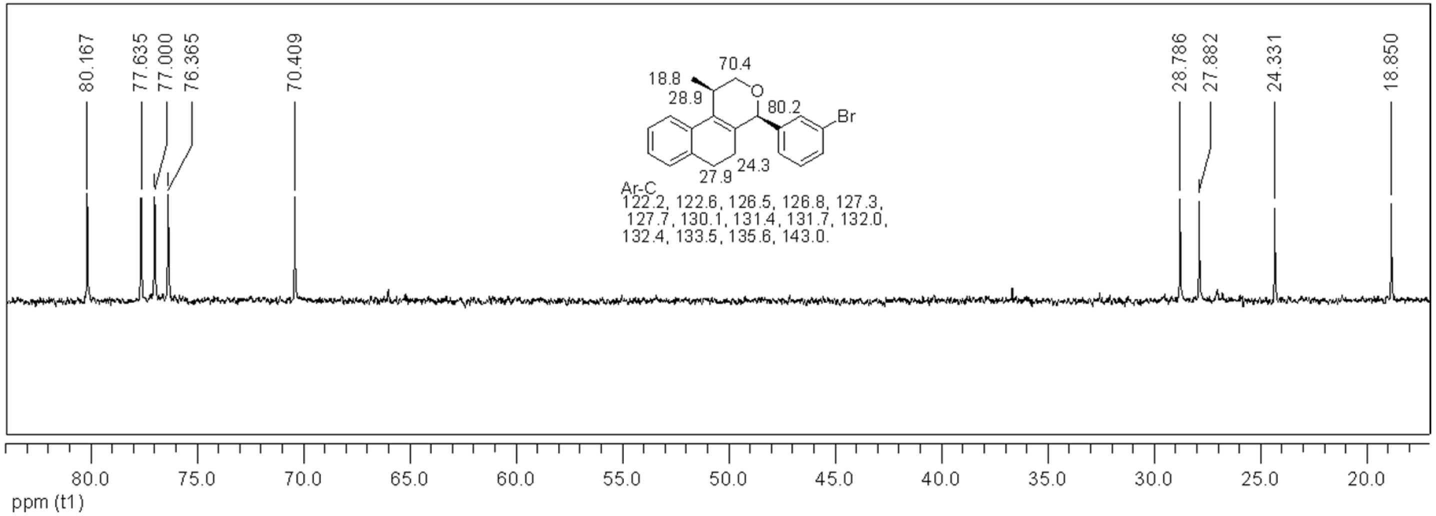

2D NOESY NMR of compound *cis*-**7k** (500 MHz, $CDCl_3$)

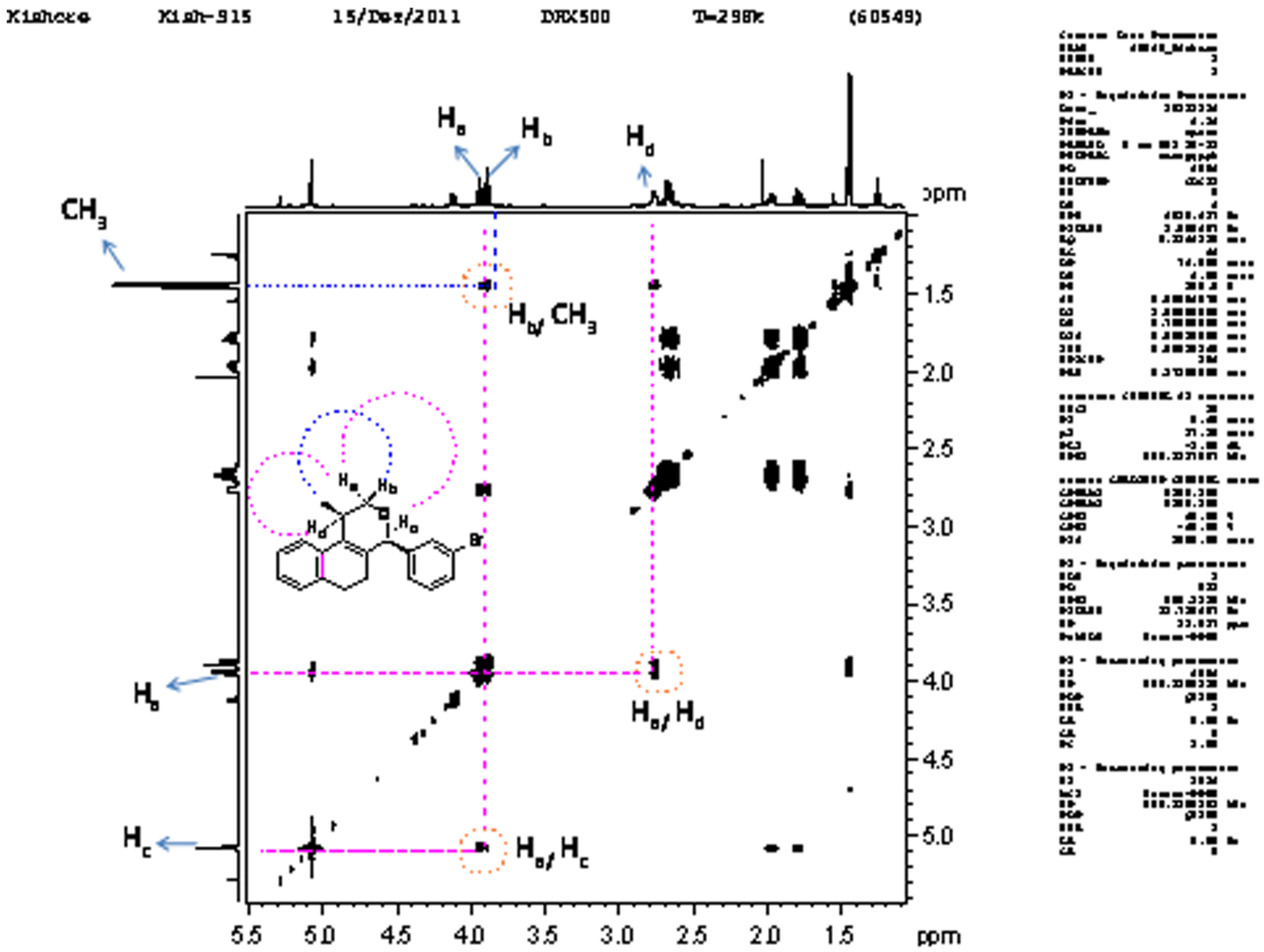

^{1}H-NMR of compounds *cis*-**7l** and **12l** (500 MHz, $CDCl_3$)

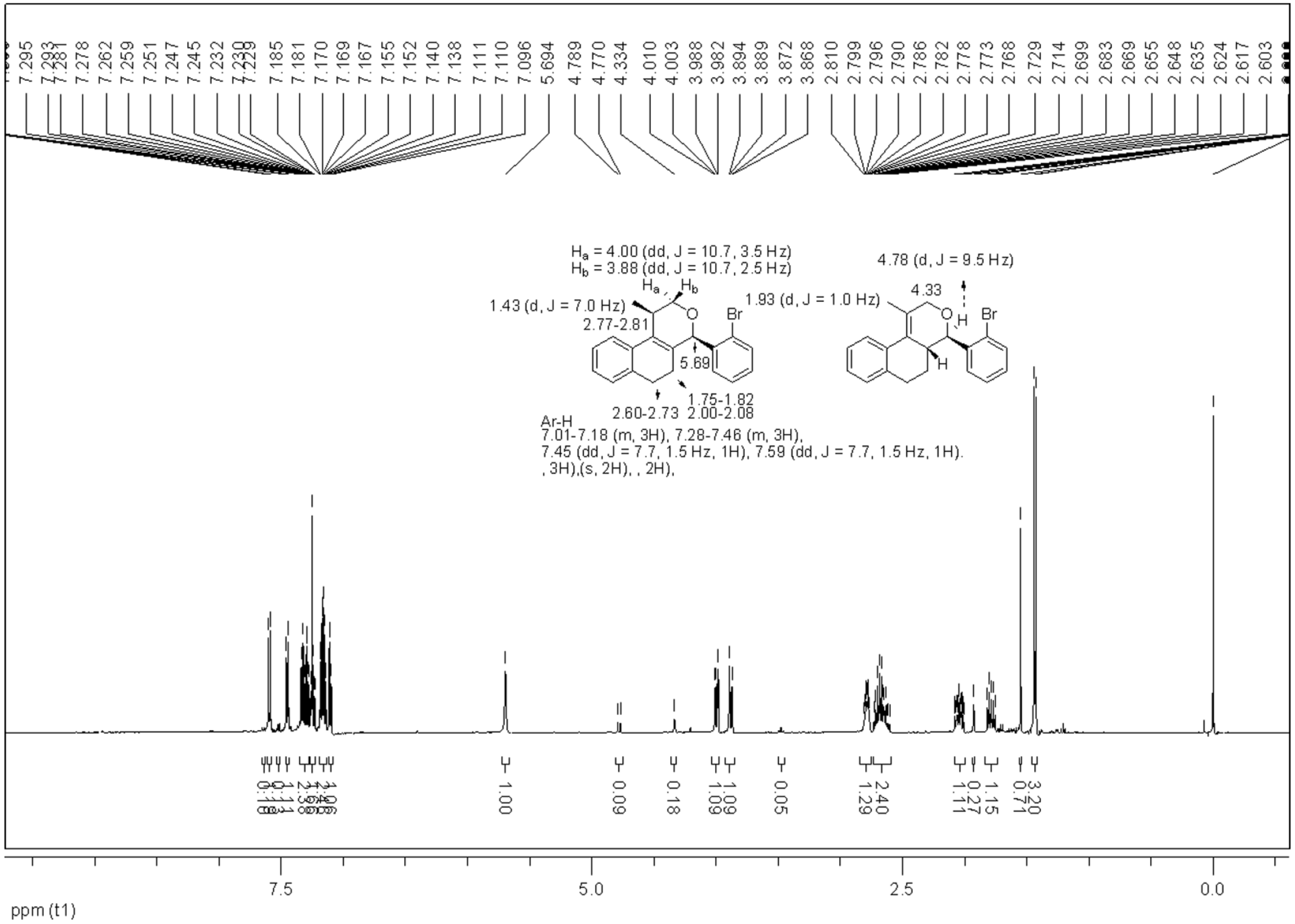

^{1}H-NMR of compounds *cis*-**7l** and **12l** (500 MHz, $CDCl_3$)

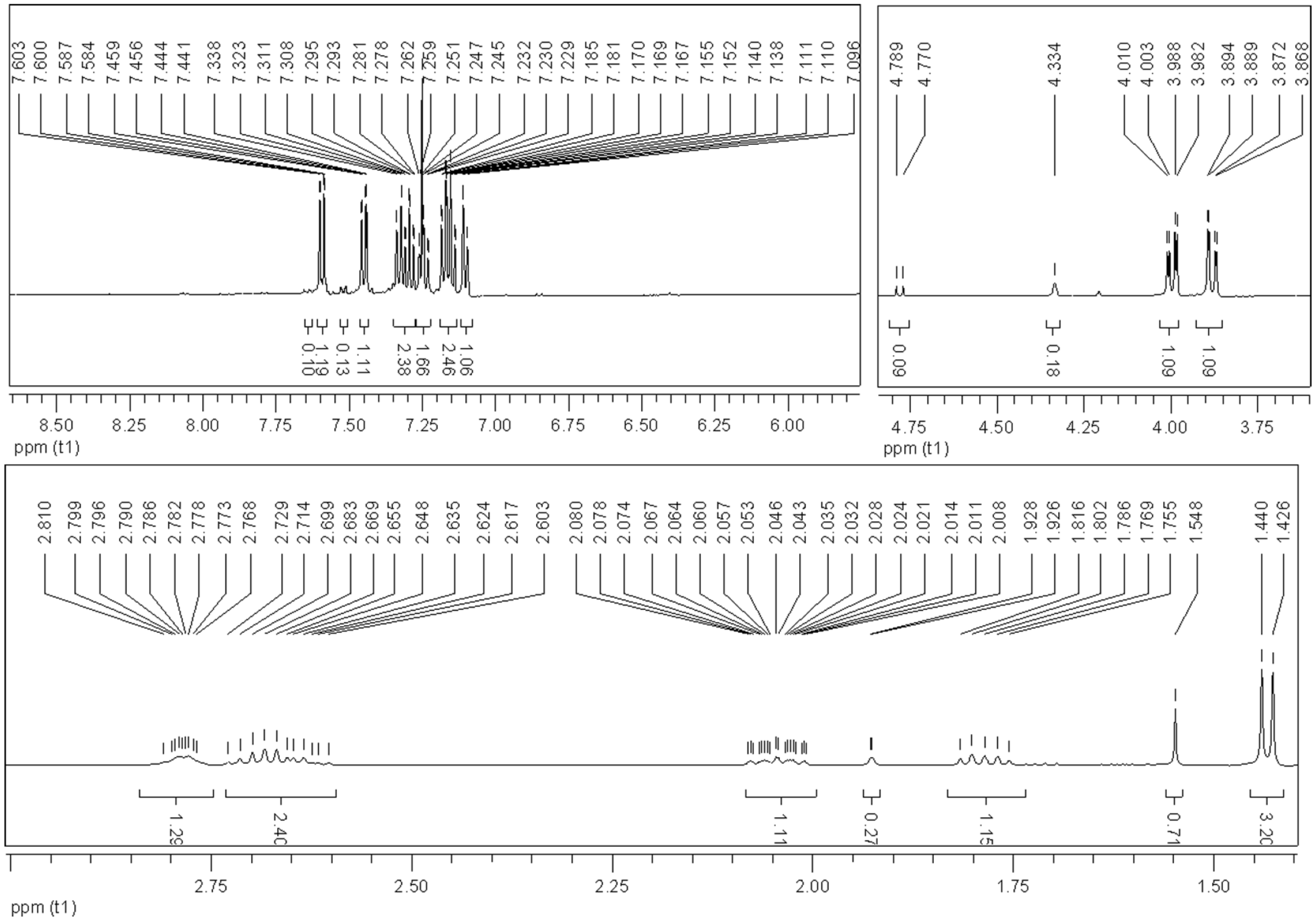

^{13}C-NMR of compounds *cis*-**7l** and **12l** (75 MHz, $CDCl_3$)

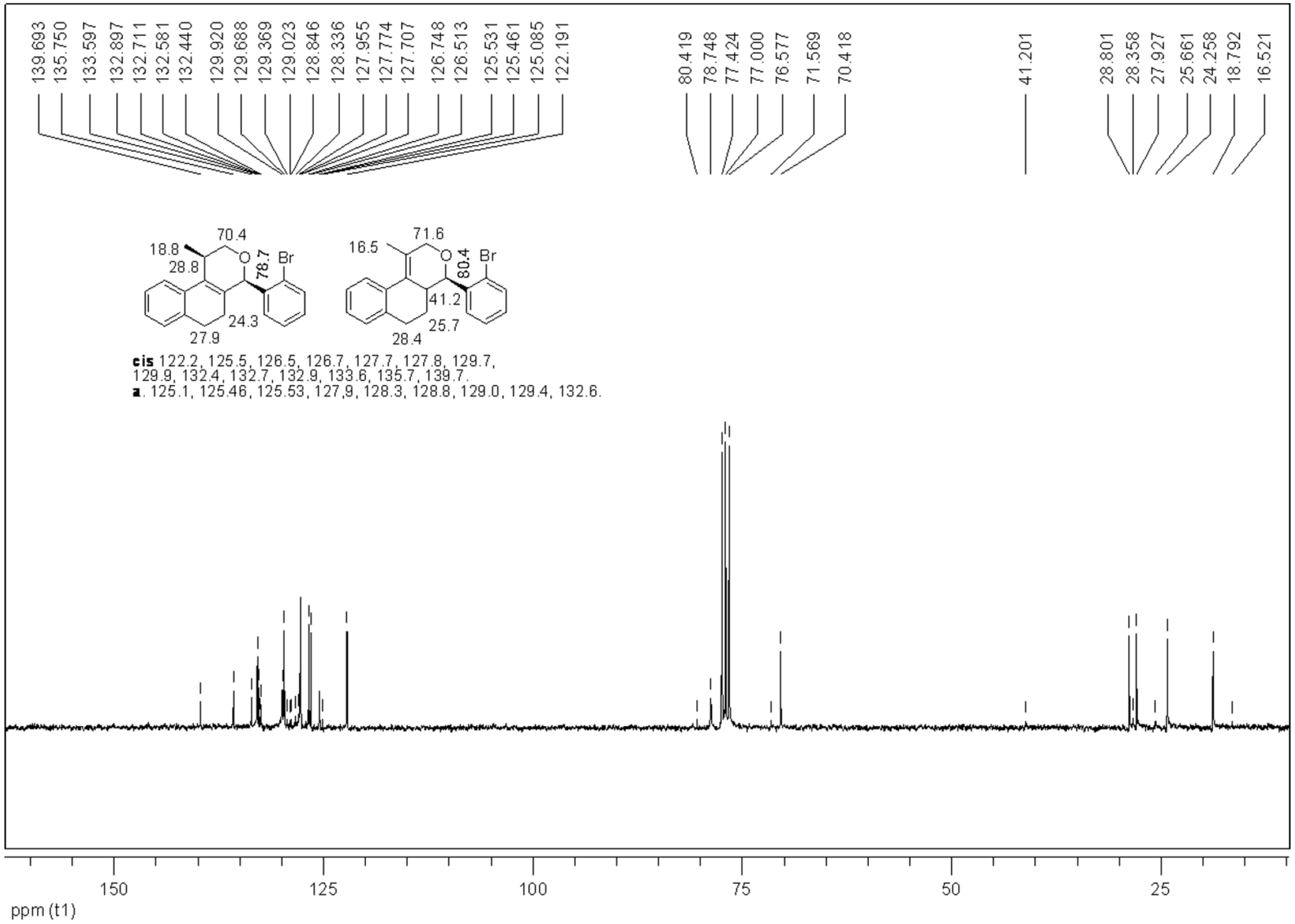

^{13}C-NMR of compounds *cis*-**7l** and **12l** (75 MHz, $CDCl_3$,)

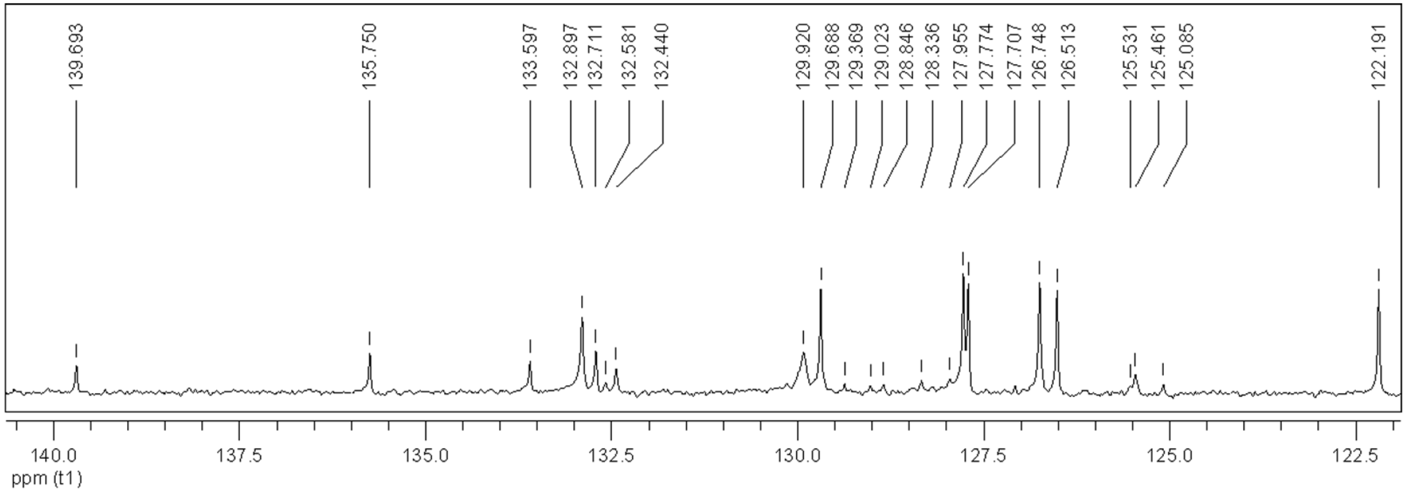

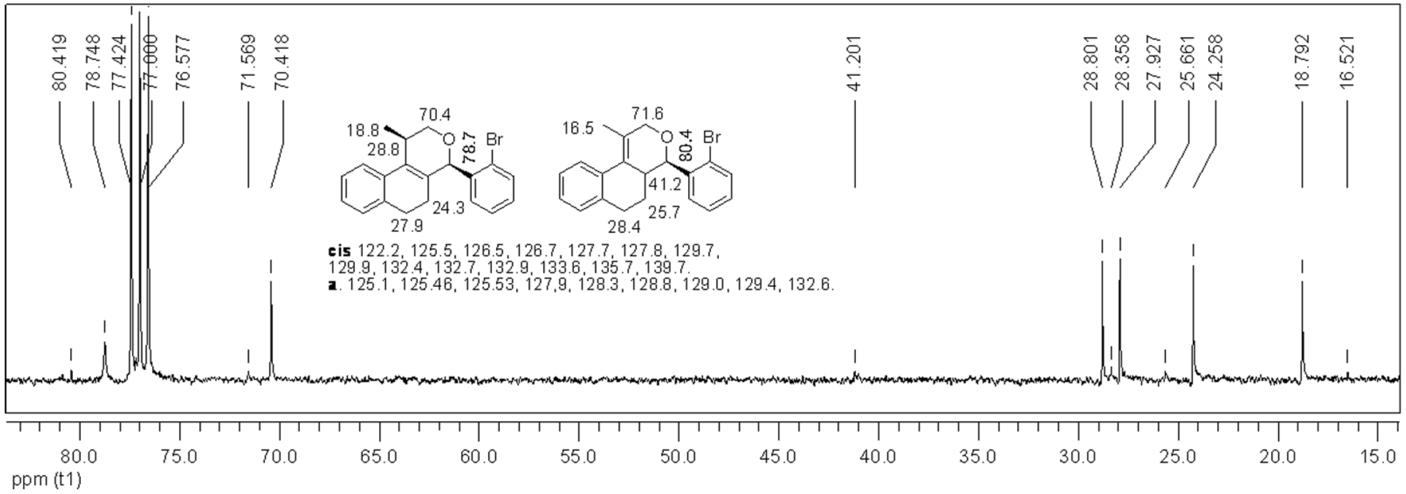

2D NOESY NMR of compound *cis*-**7l** (500 MHz, $CDCl_3$)

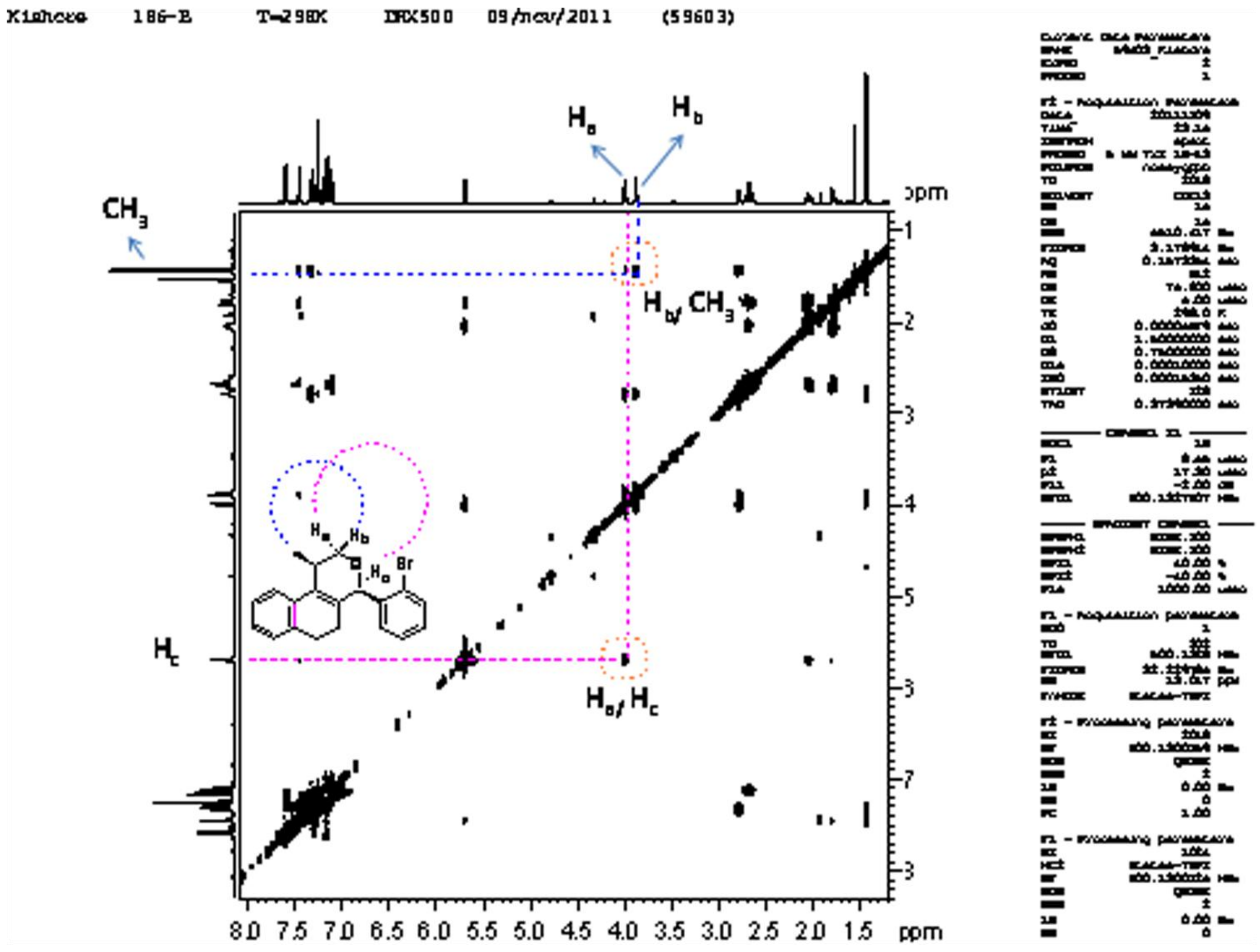

^{1}H-NMR of compound *cis*-**7n** (500 MHz, $CDCl_3$)

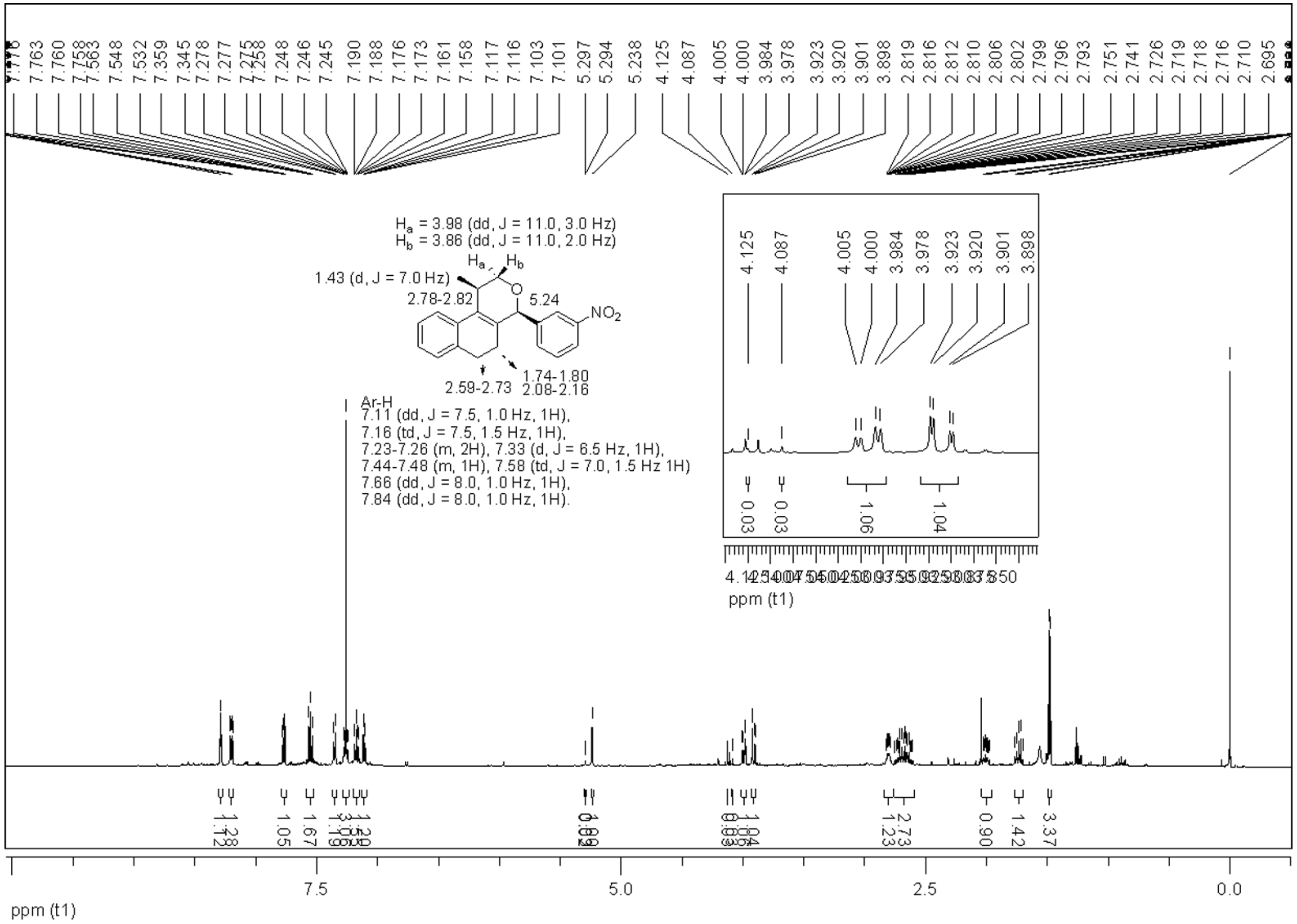

^{1}H-NMR of compound *cis*-**7n** (500 MHz, $CDCl_3$)

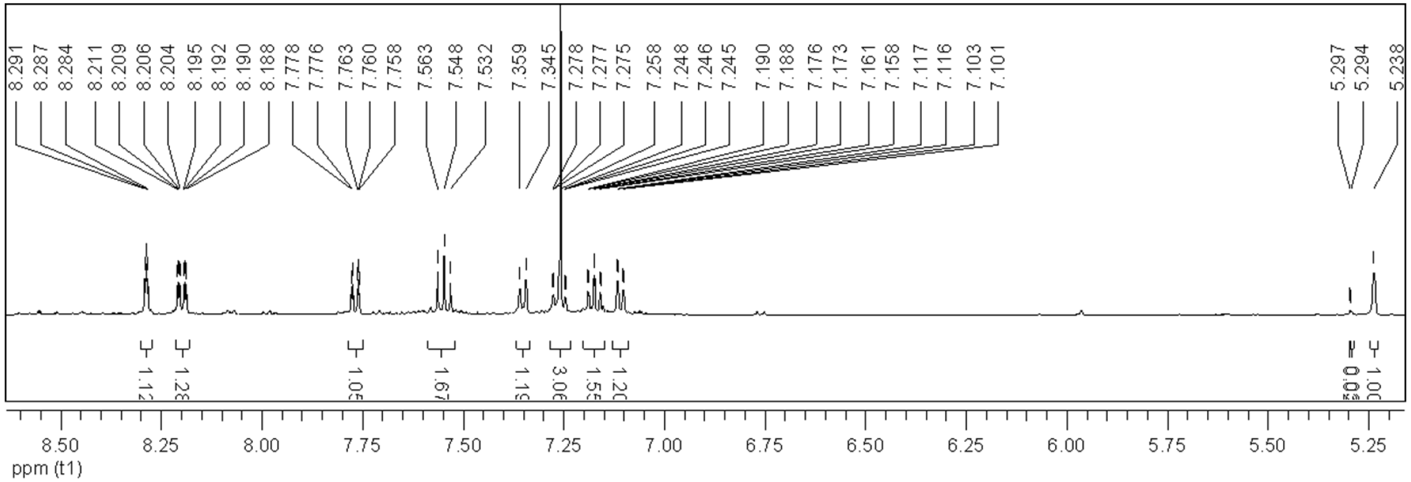

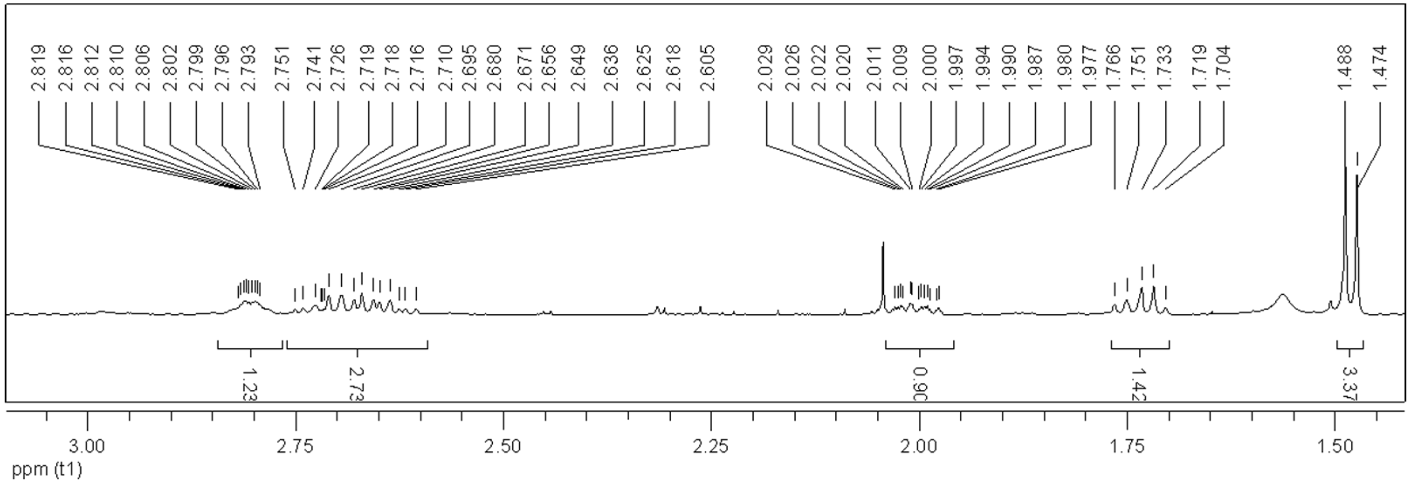

^{13}C-NMR of compound *cis*-**7n** (50 MHz, $CDCl_3$)

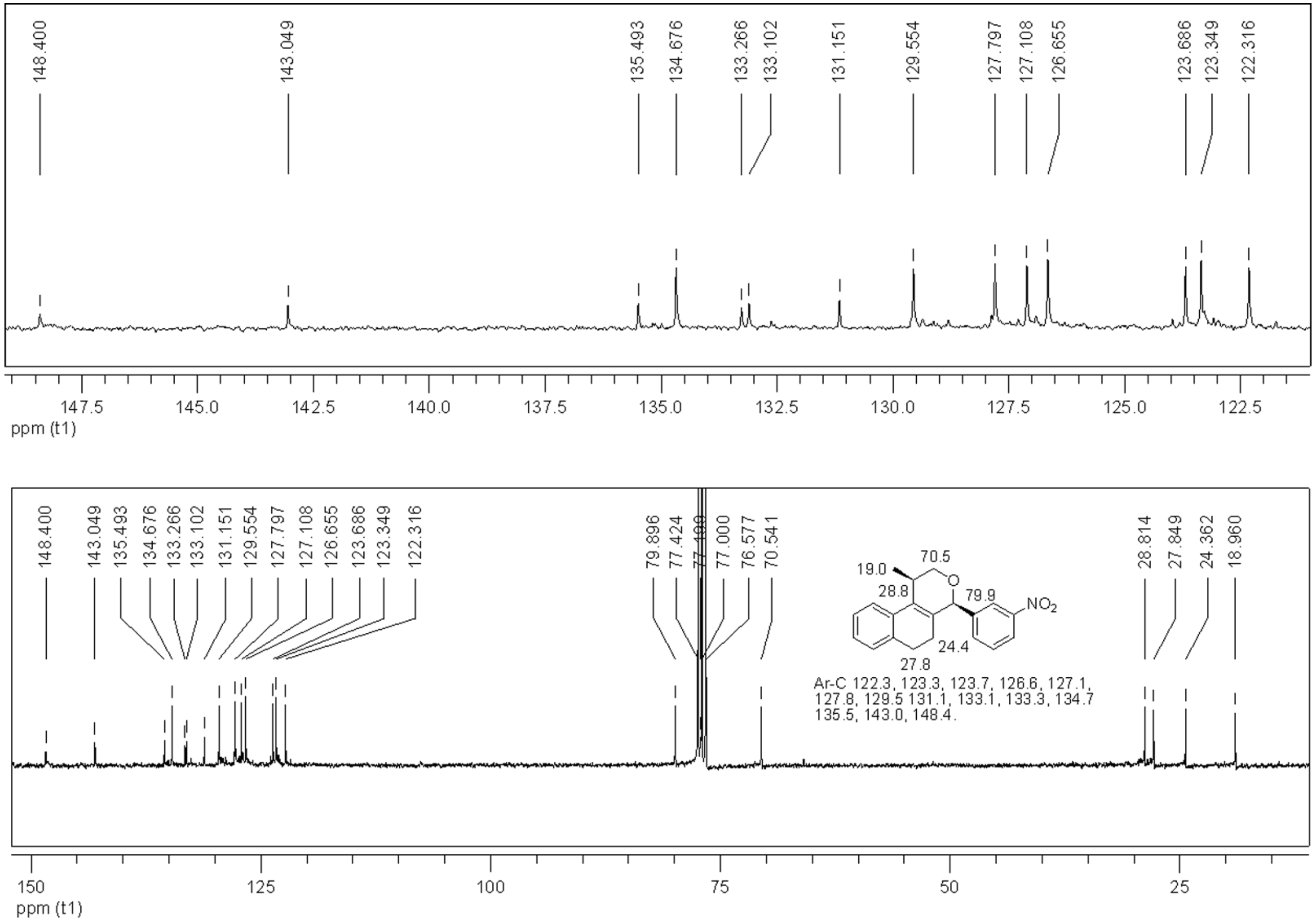

2D NOESY NMR of compound *cis*-**7n** (500 MHz, $CDCl_3$)

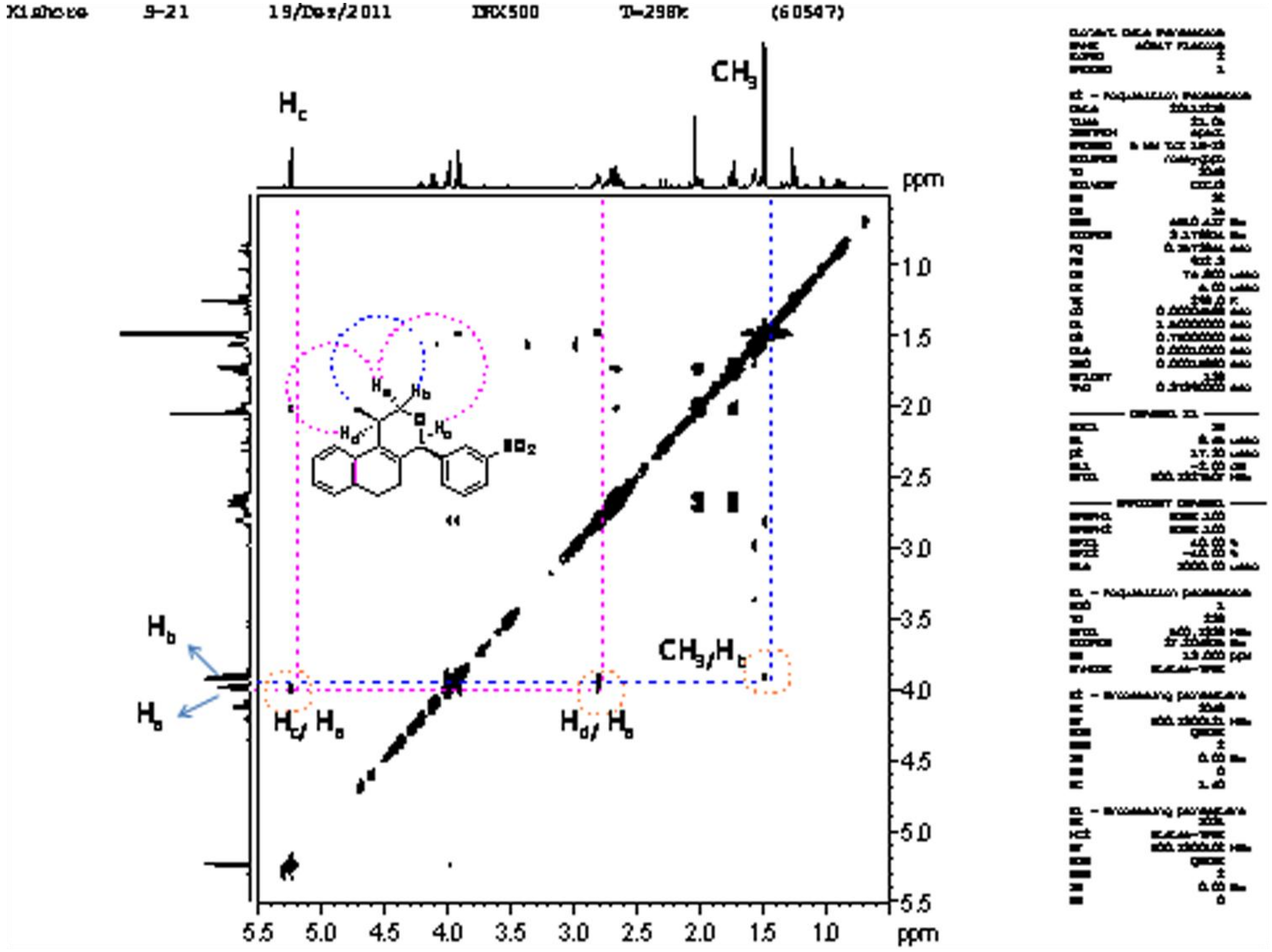

^{1}H-NMR of compounds *cis*-**7o** and **12o** (500 MHz, $CDCl_3$)

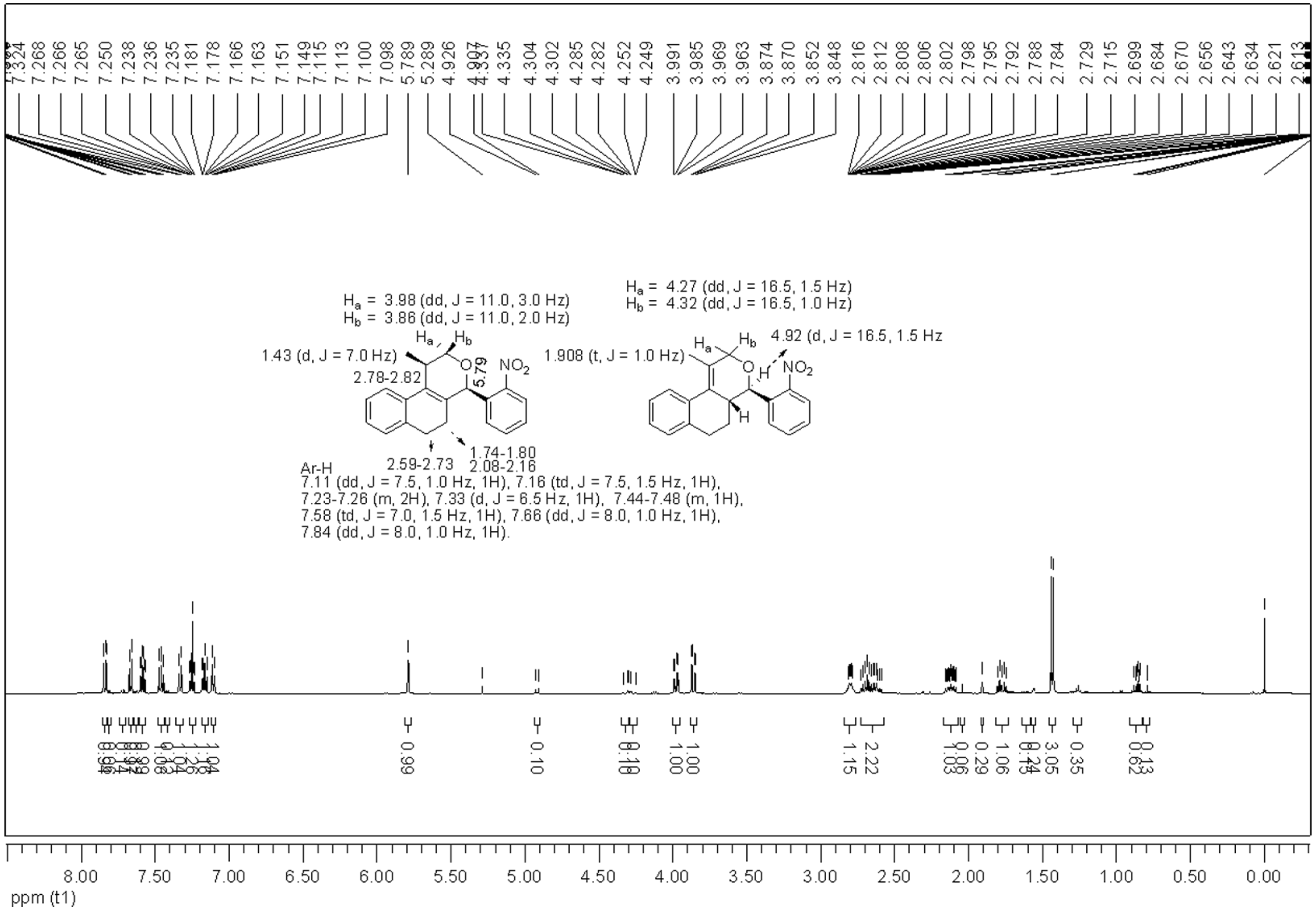

^{1}H-NMR of compounds *cis*-**7o** and **12o** (500 MHz, $CDCl_3$)

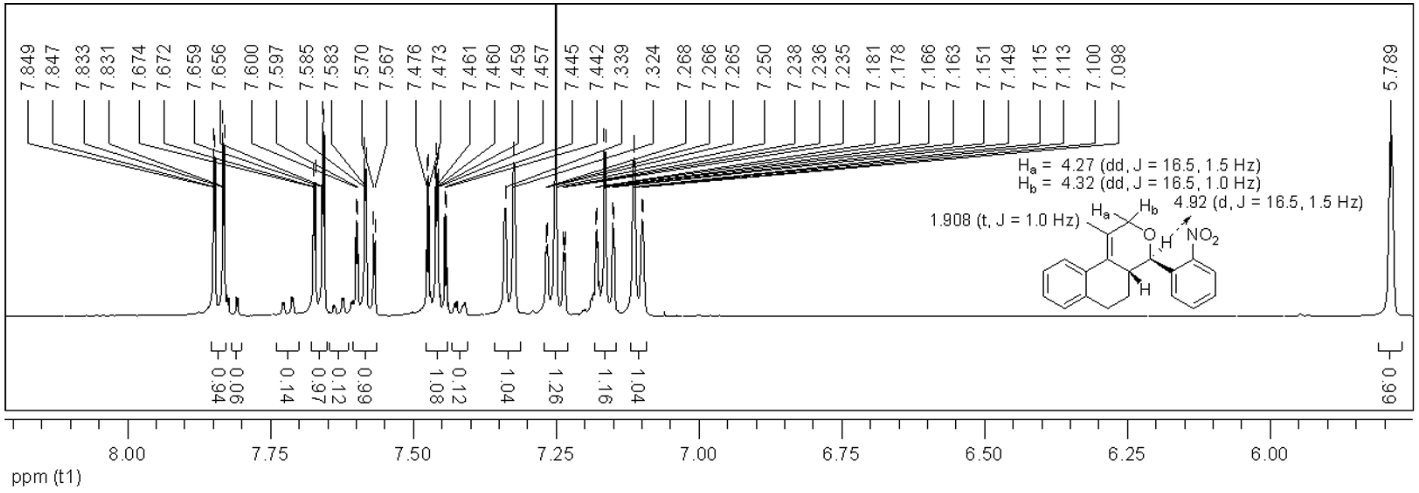

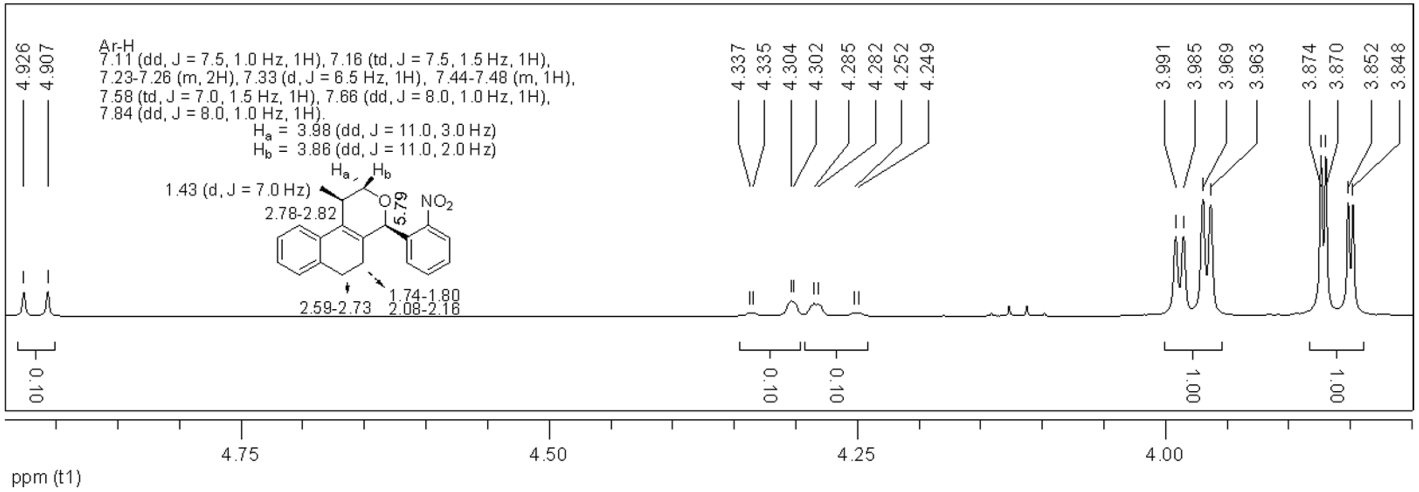

^{1}H-NMR of compounds *cis*-**7o** and **12o** (500 MHz, $CDCl_3$)

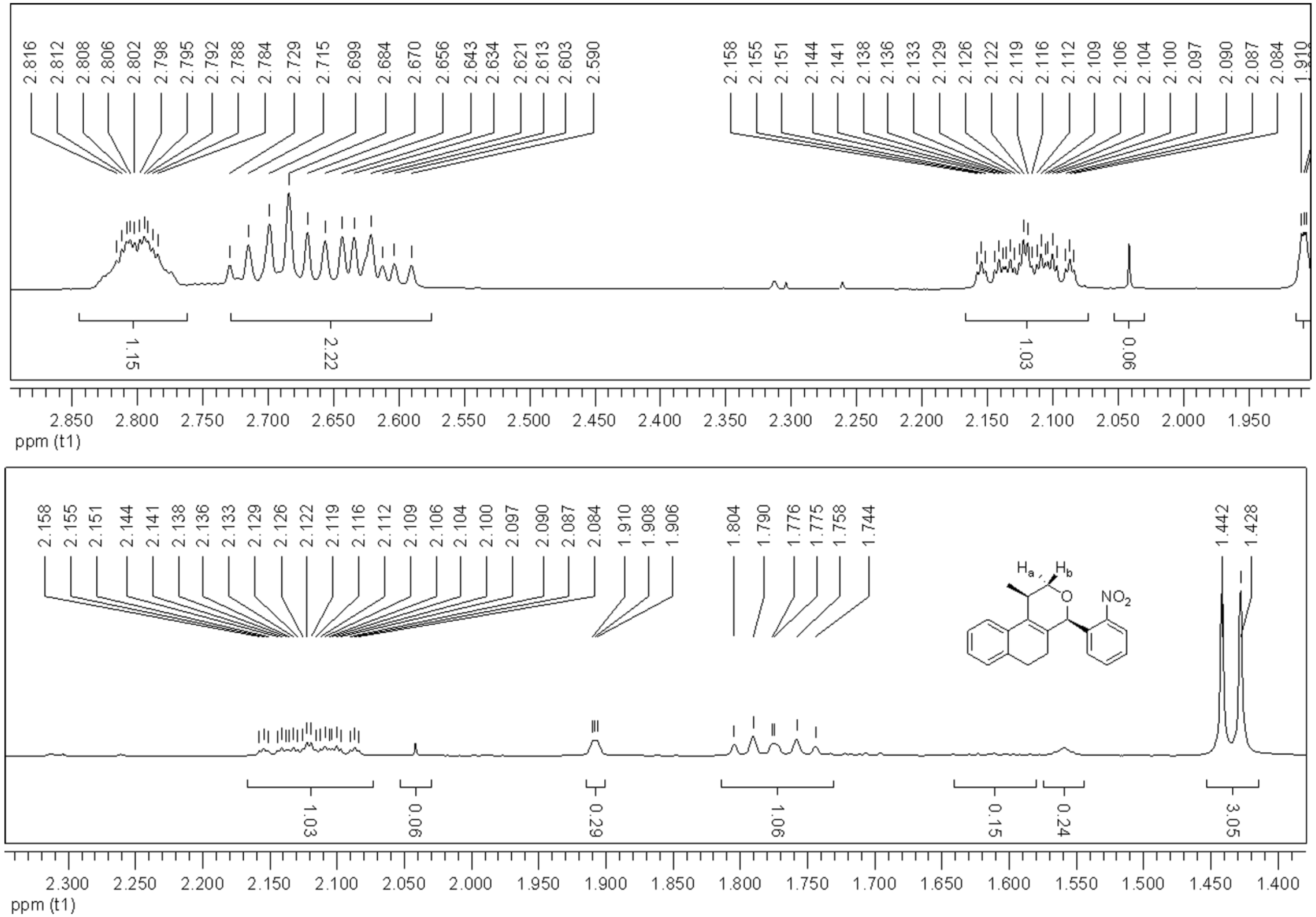

^{13}C-NMR of compounds *cis*-**7o** and **12o** (75 MHz, $CDCl_3$)

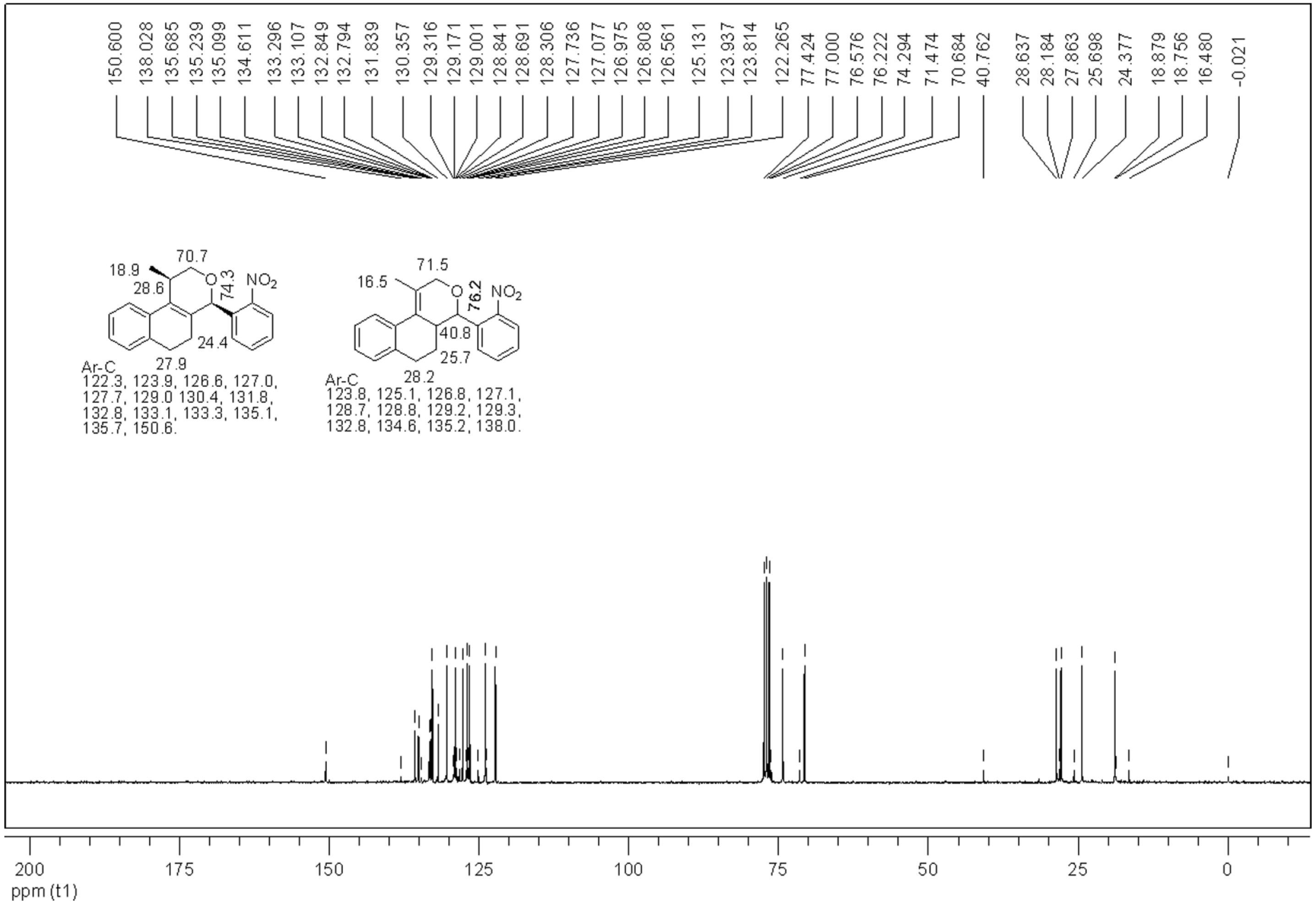

^{13}C-NMR of compounds *cis*-**7o** and **12o** (75 MHz, $CDCl_3$)

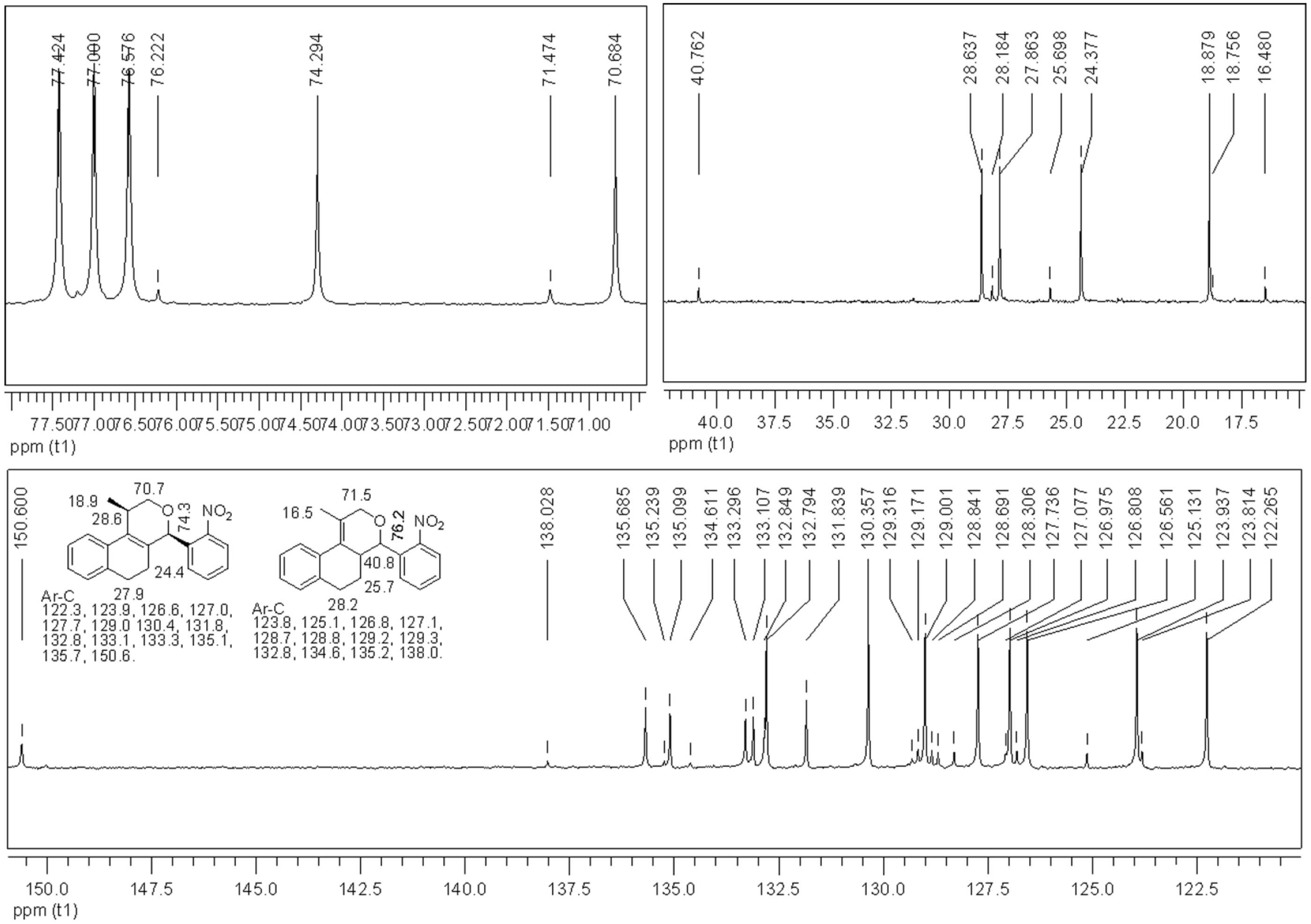

2D NOESY NMR of compounds *cis*-**7o** (500 MHz, $CDCl_3$)

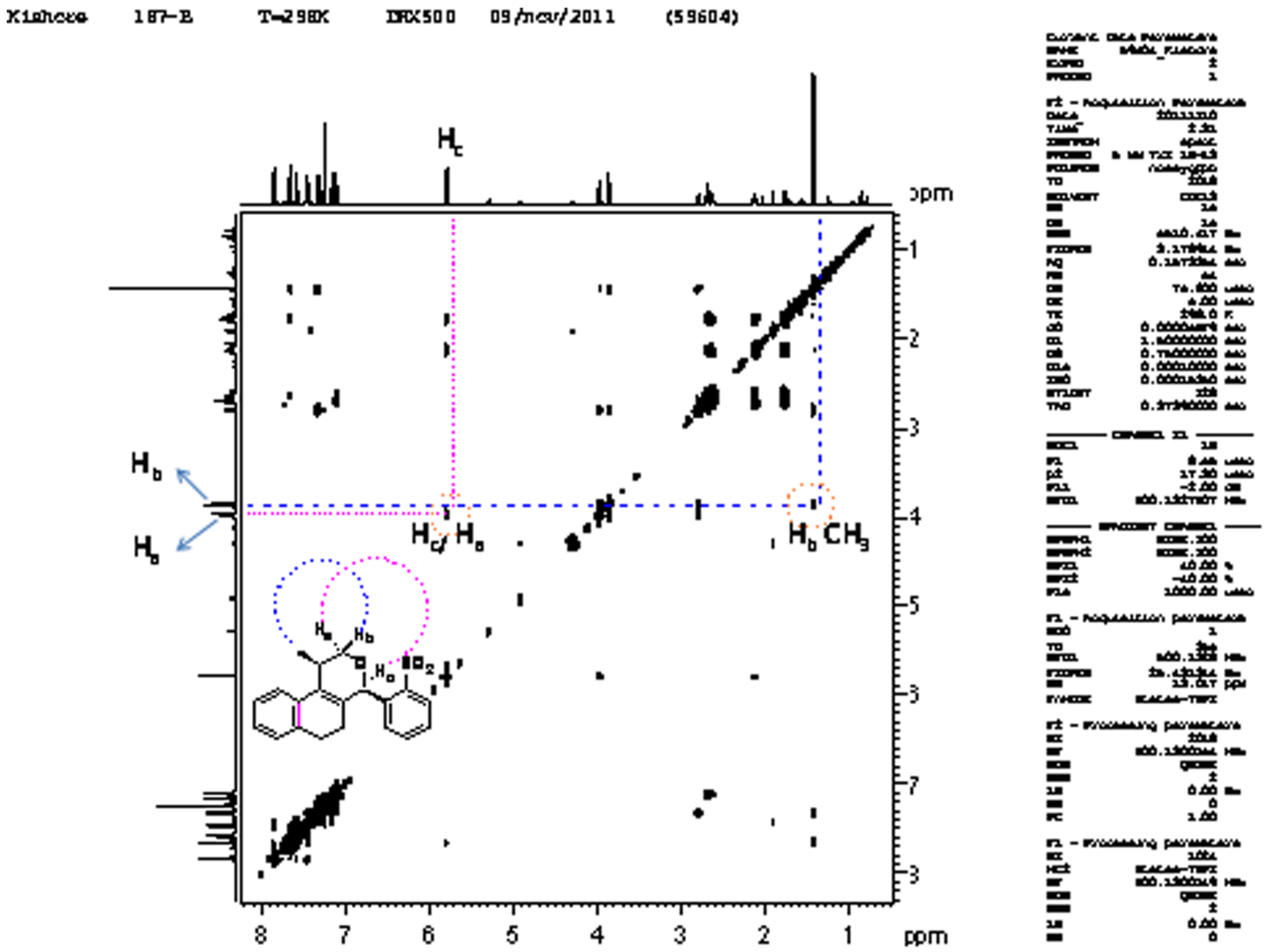

^{1}H-NMR of compound *cis*-**7q** (200 MHz, $CDCl_3$)

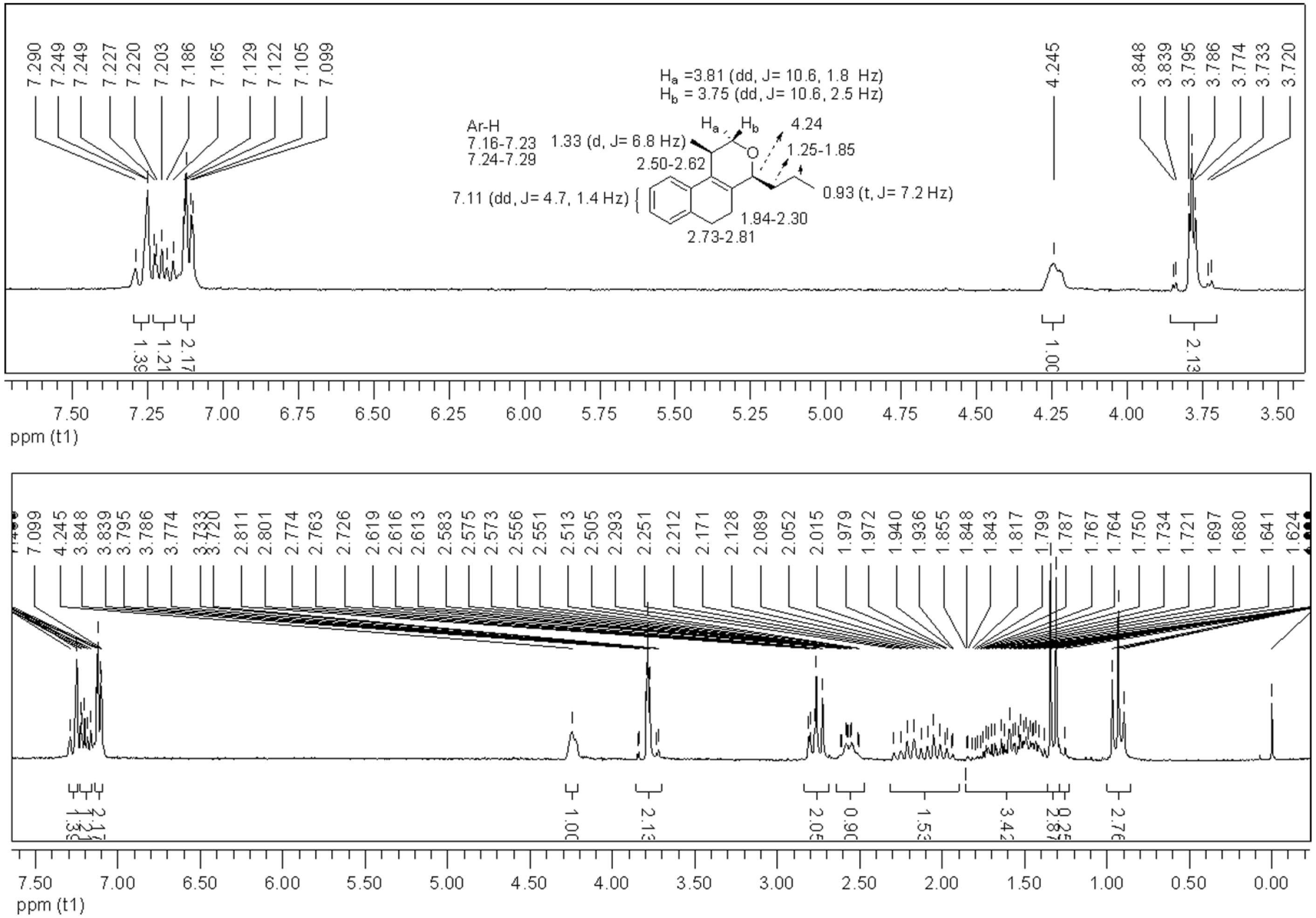

^{1}H-NMR of compound *cis*-**7q** (200 MHz, $CDCl_3$)

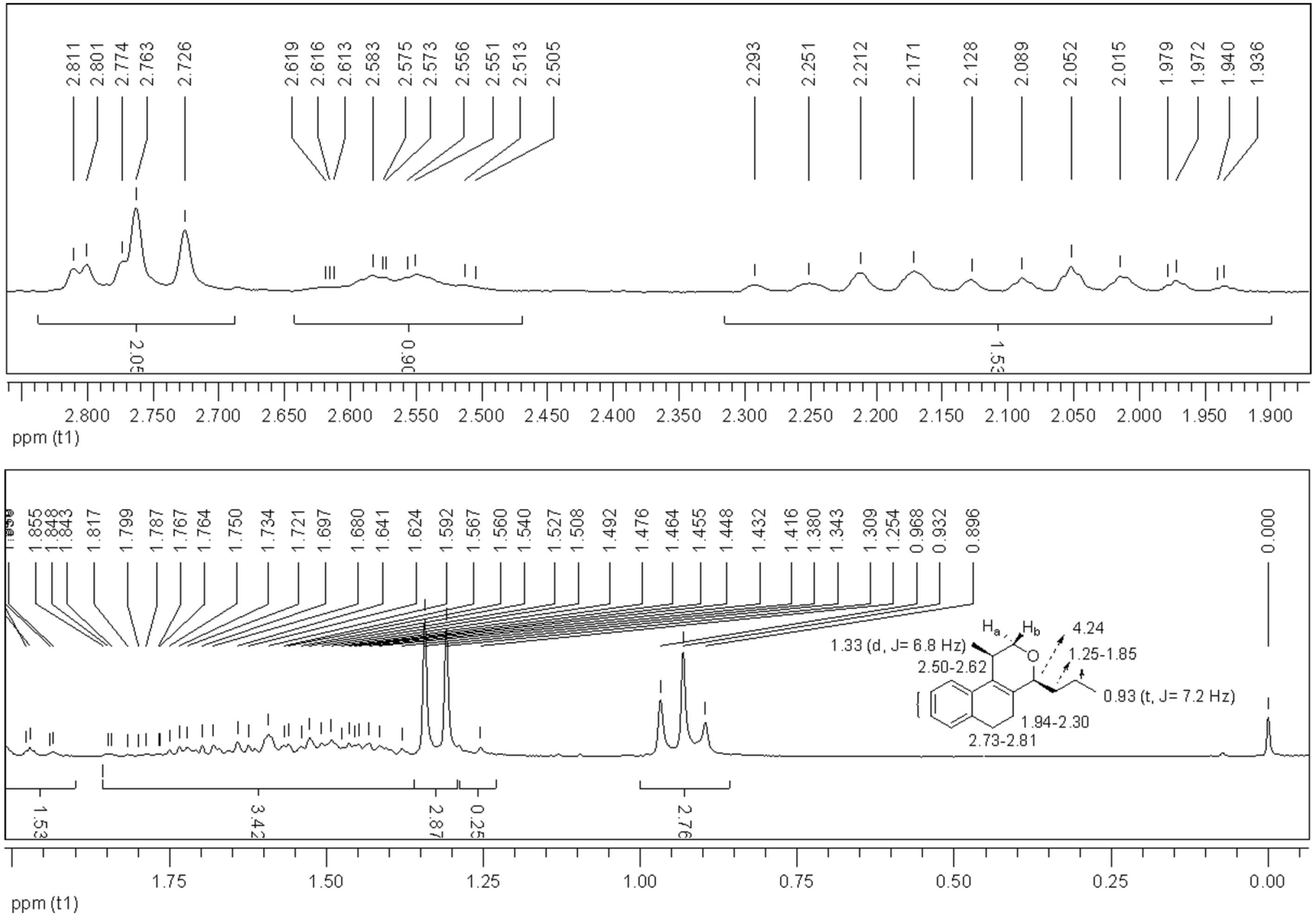

^{13}C-NMR of compound *cis*-**7q** (50 MHz, $CDCl_3$)

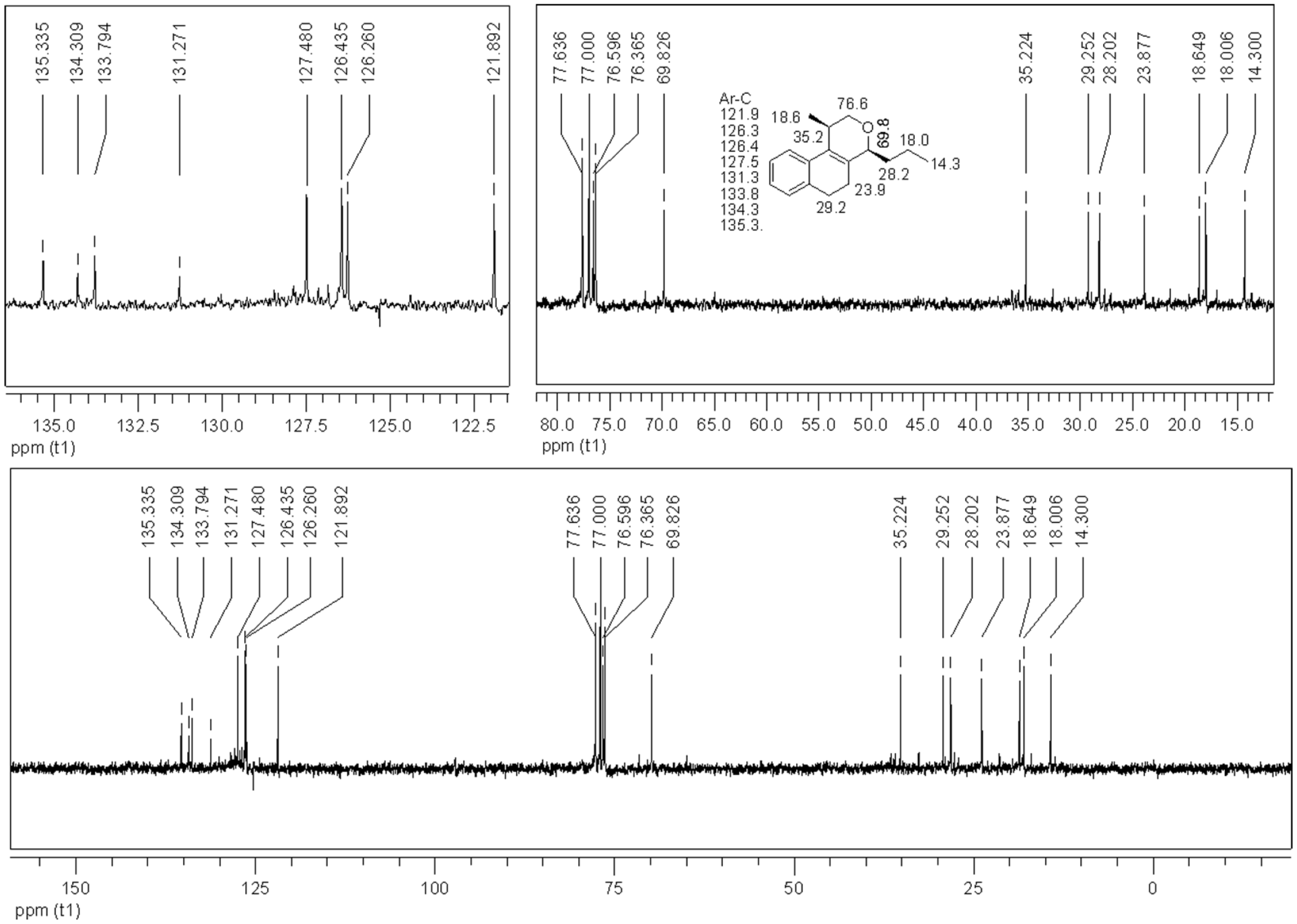

^{1}H-NMR of compound *cis*-**7r** and *trans*-**7r** (300 MHz, $CDCl_3$)

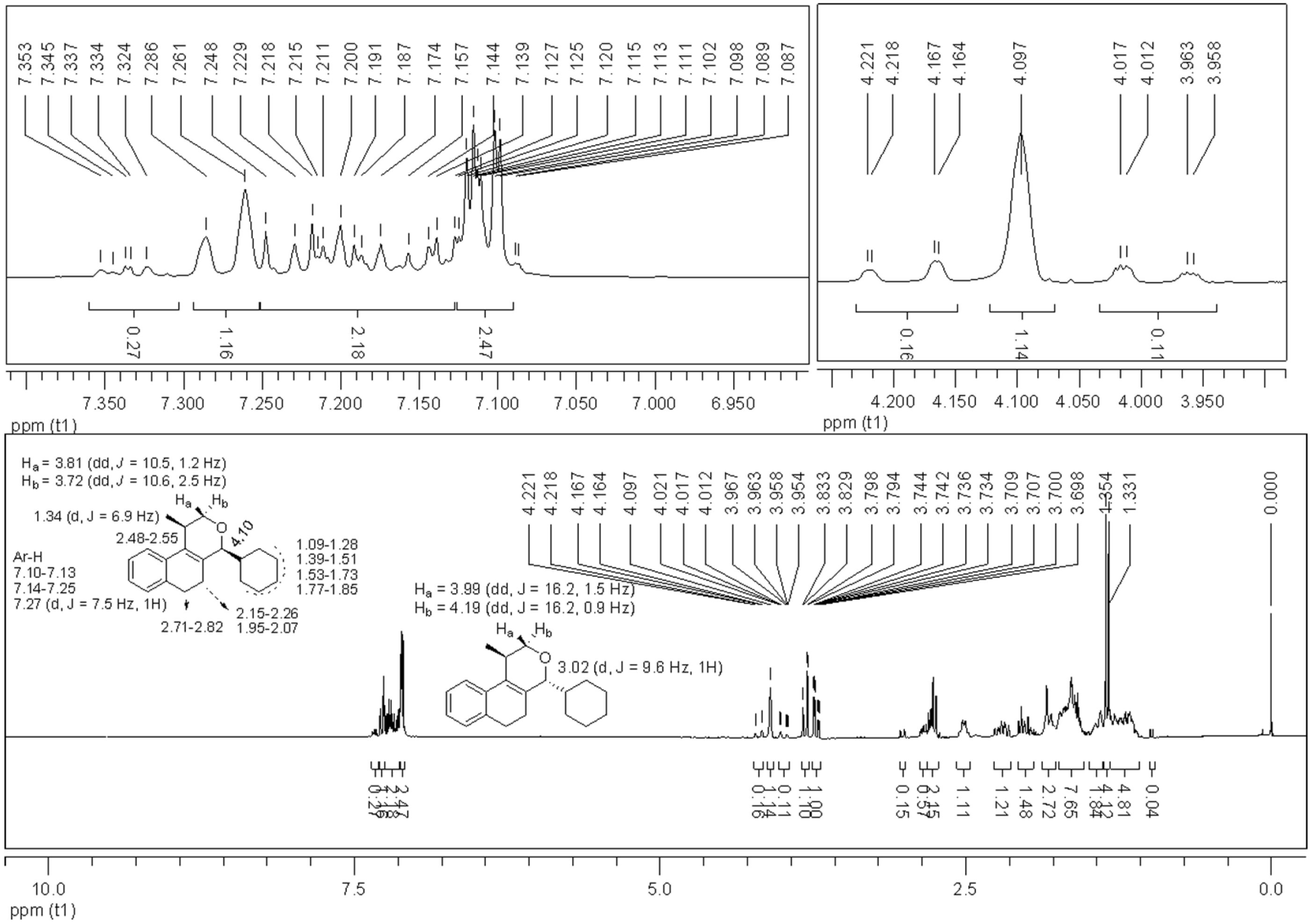

^{1}H-NMR of compound *cis*-**7r** and *trans*-**7r** (300 MHz, $CDCl_3$)

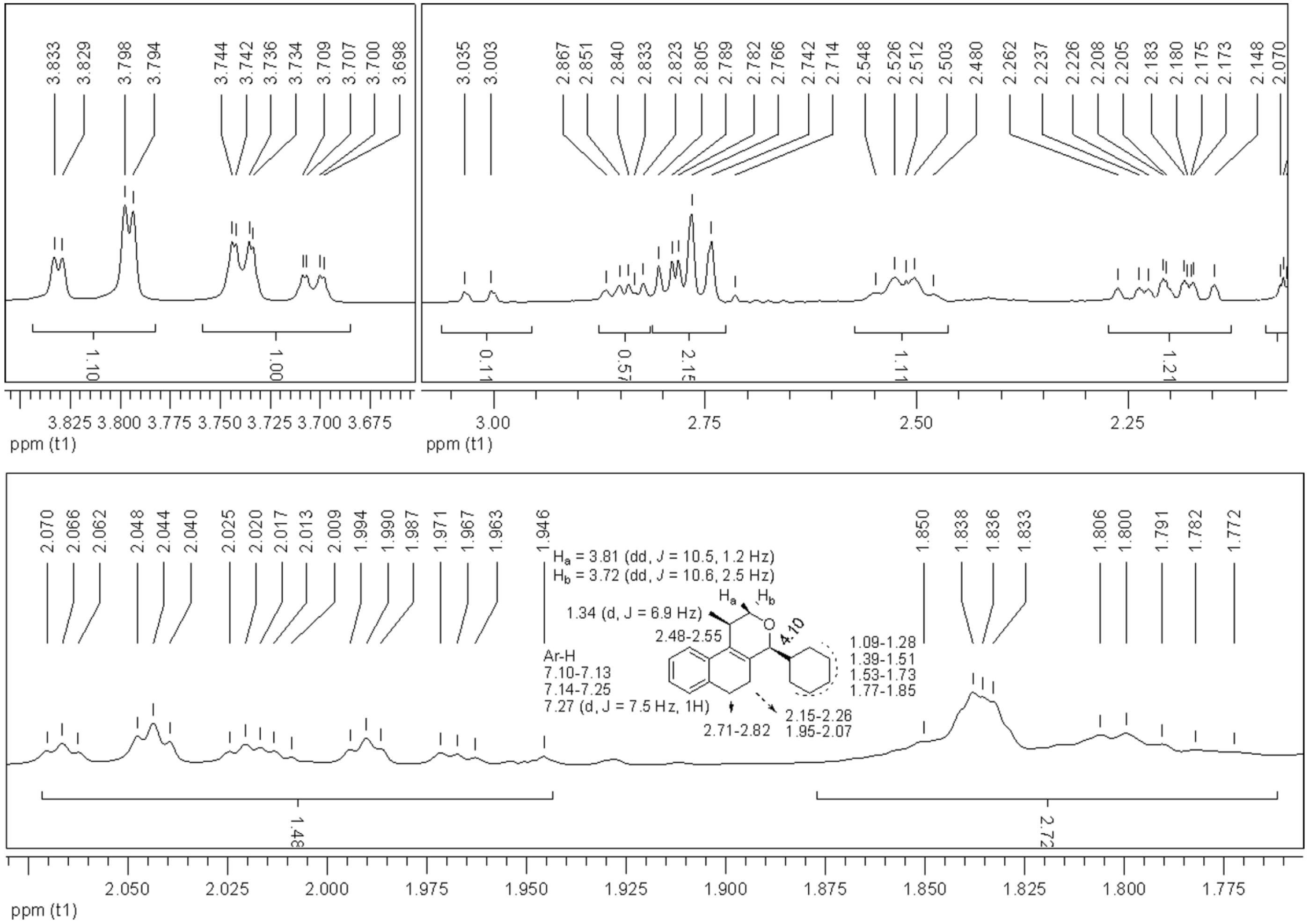

^{13}C-NMR of compound *cis*-**7r** and *trans*-**7r** (75 MHz, $CDCl_3$)

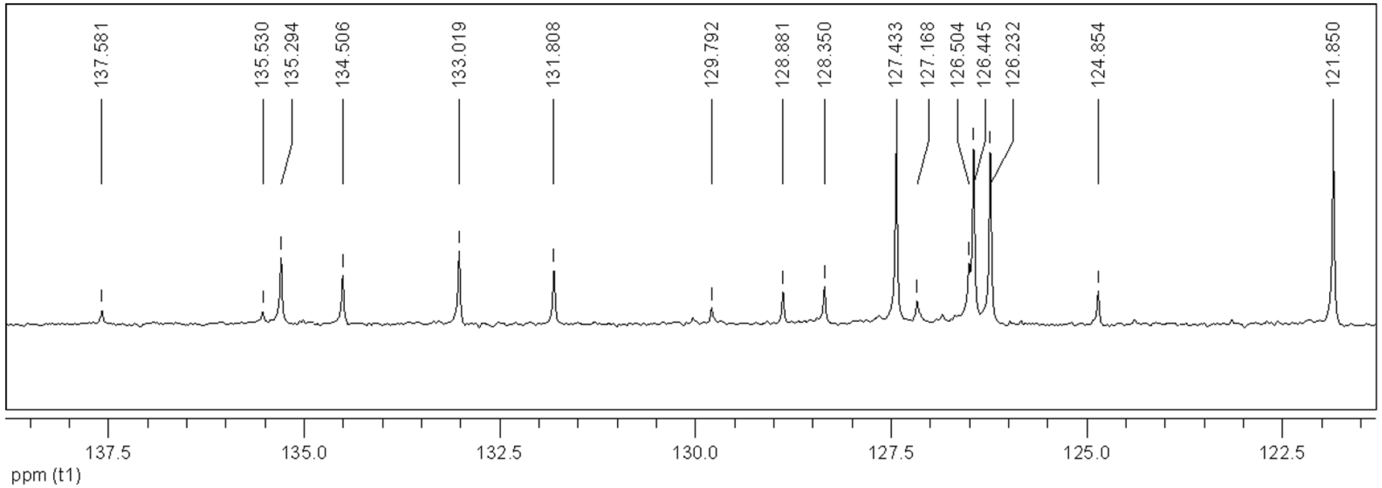

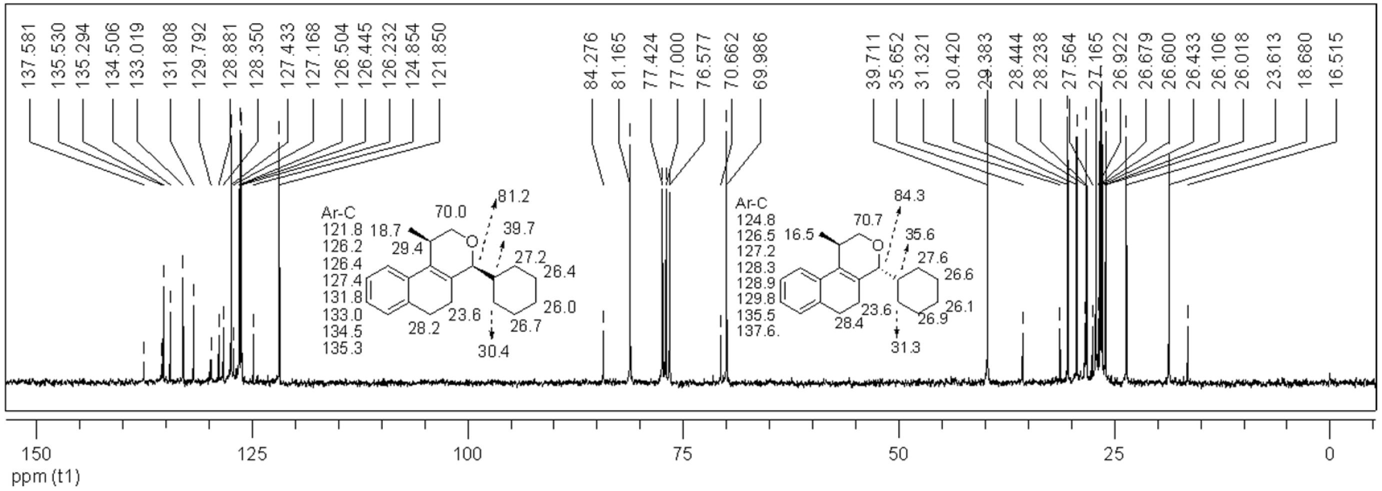

^{13}C-NMR of compound *cis*-**7r** and *trans*-**7r** (75 MHz, $CDCl_3$)

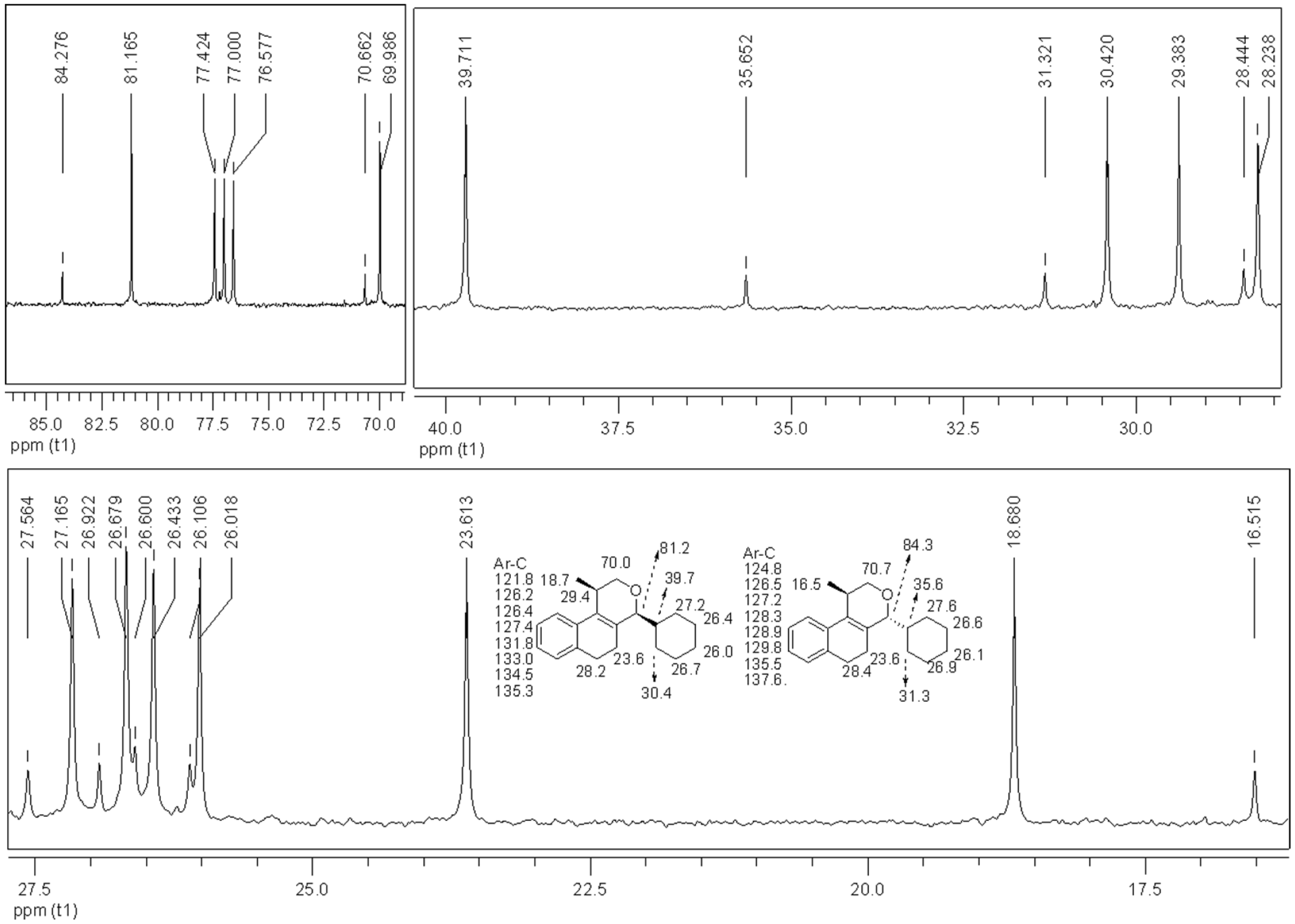

2D NOESY NMR of compound *cis*-**7r** and *trans*-**7r** (300 MHz, $CDCl_3$)

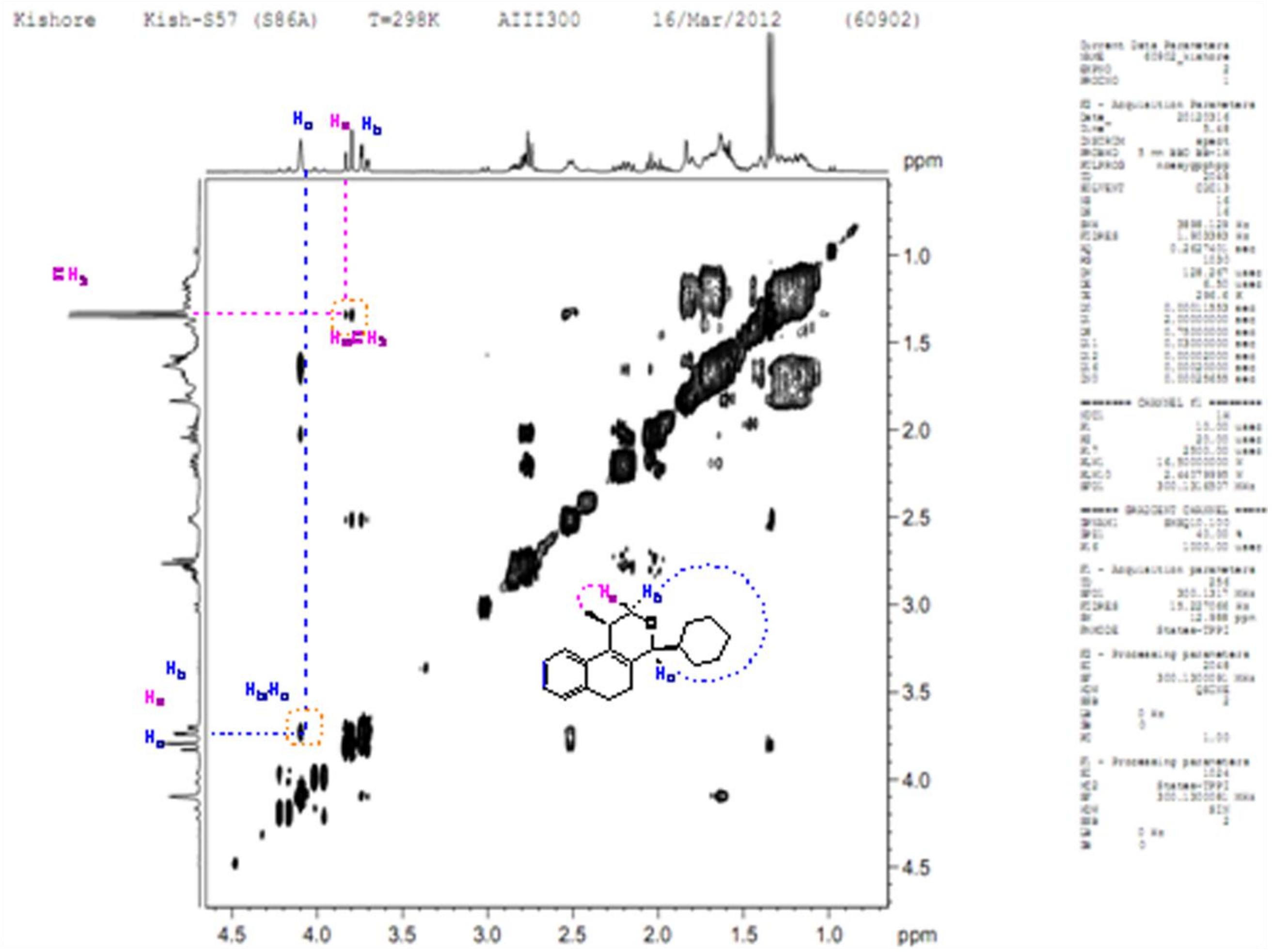

^{1}H-NMR of compound *cis*-**7t** (300 MHz, $CDCl_3$)

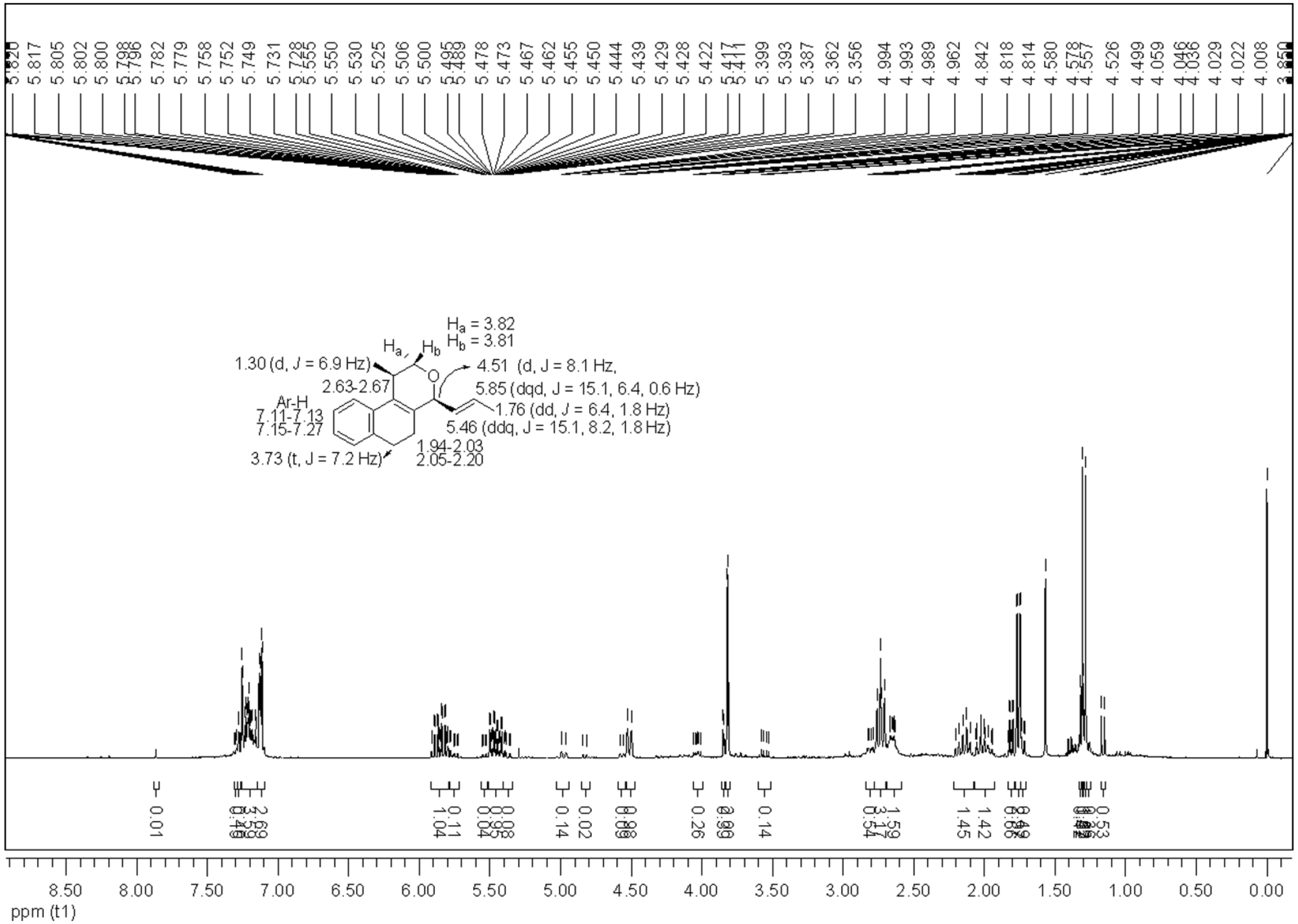

^{1}H-NMR of compound *cis*-**7t** (300 MHz, $CDCl_3$)

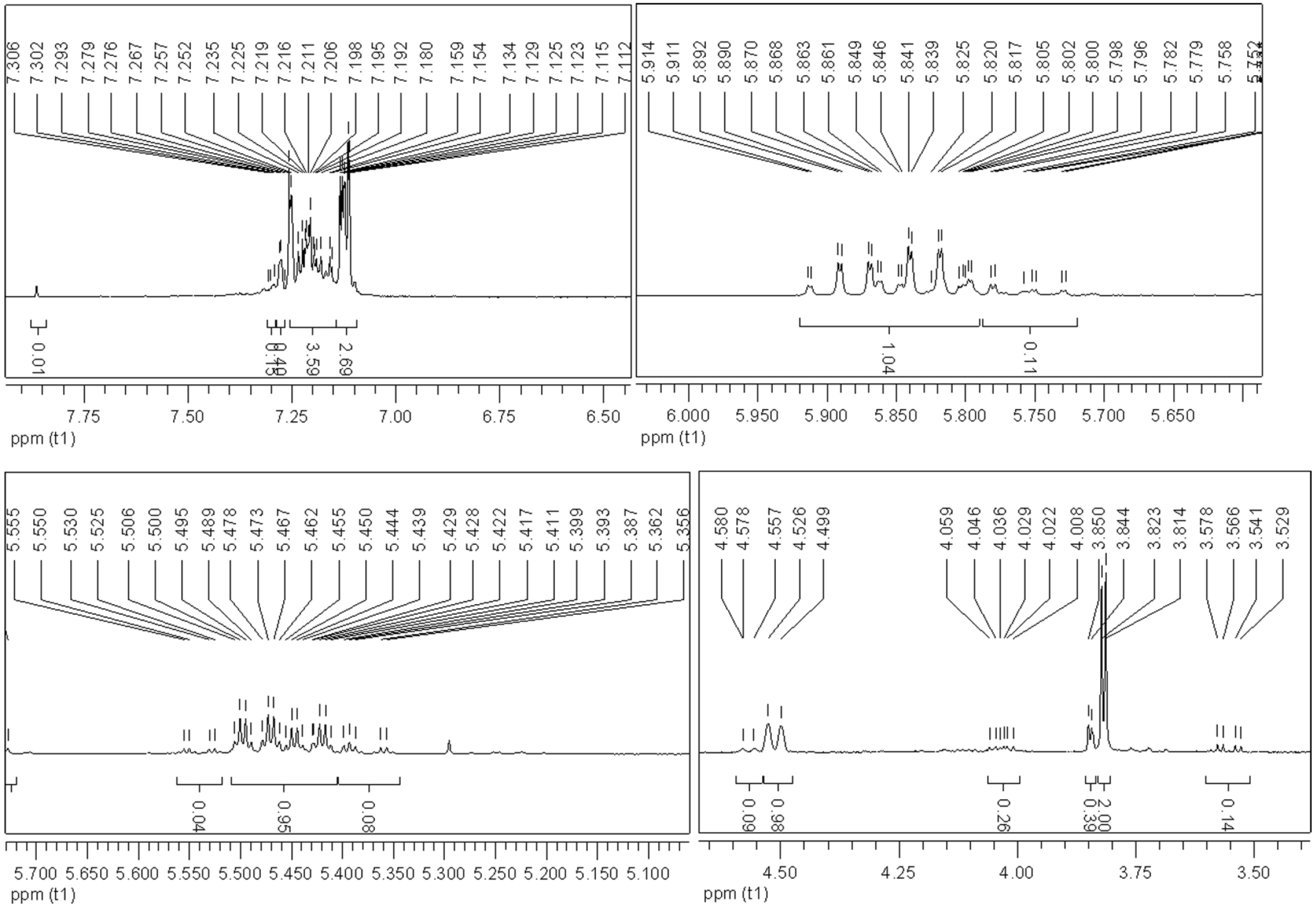

^{1}H-NMR of compound *cis*-**7t** (300 MHz, $CDCl_3$)

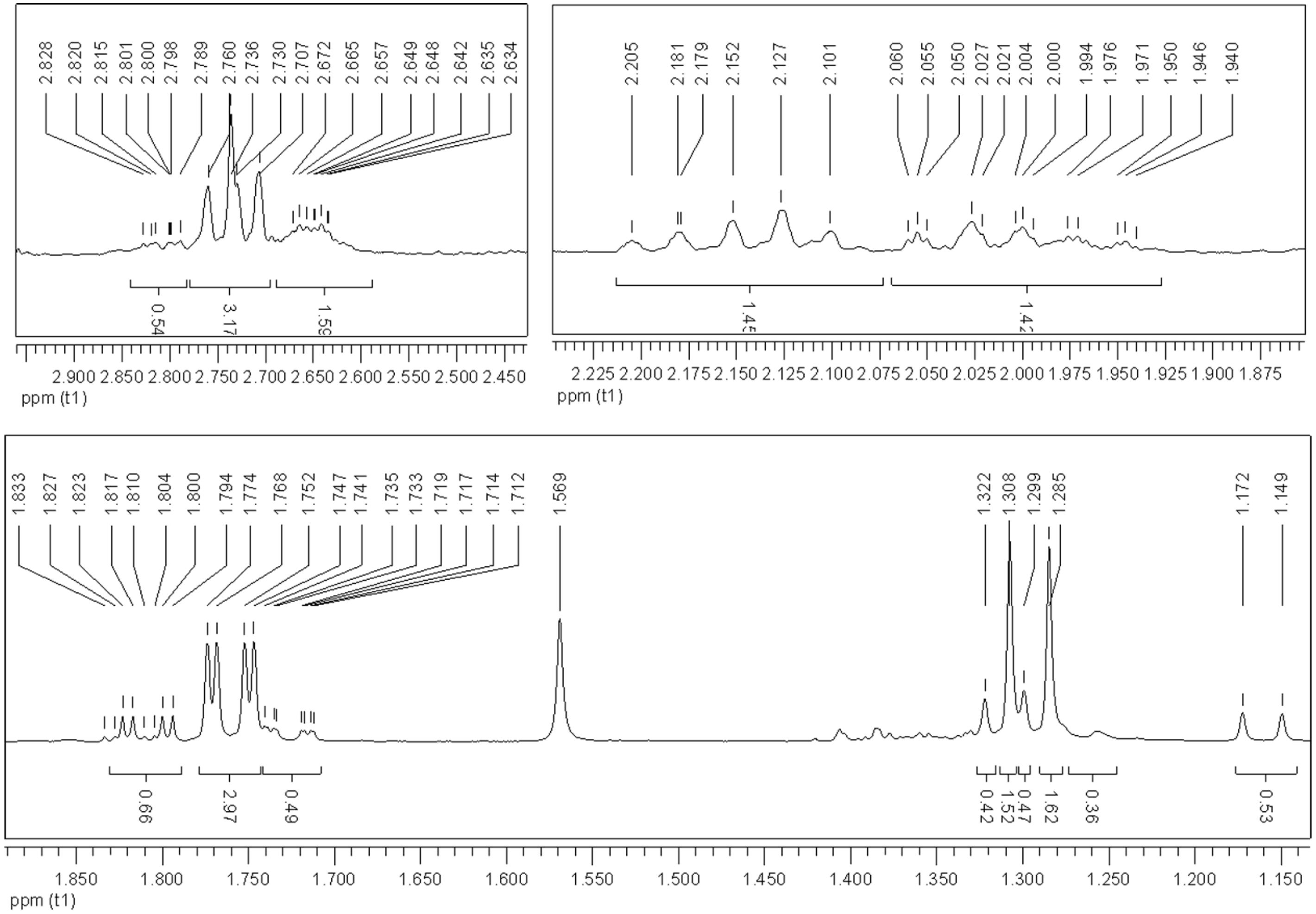

^{13}C-NMR of compound *cis*-**7t** (75 MHz, $CDCl_3$)

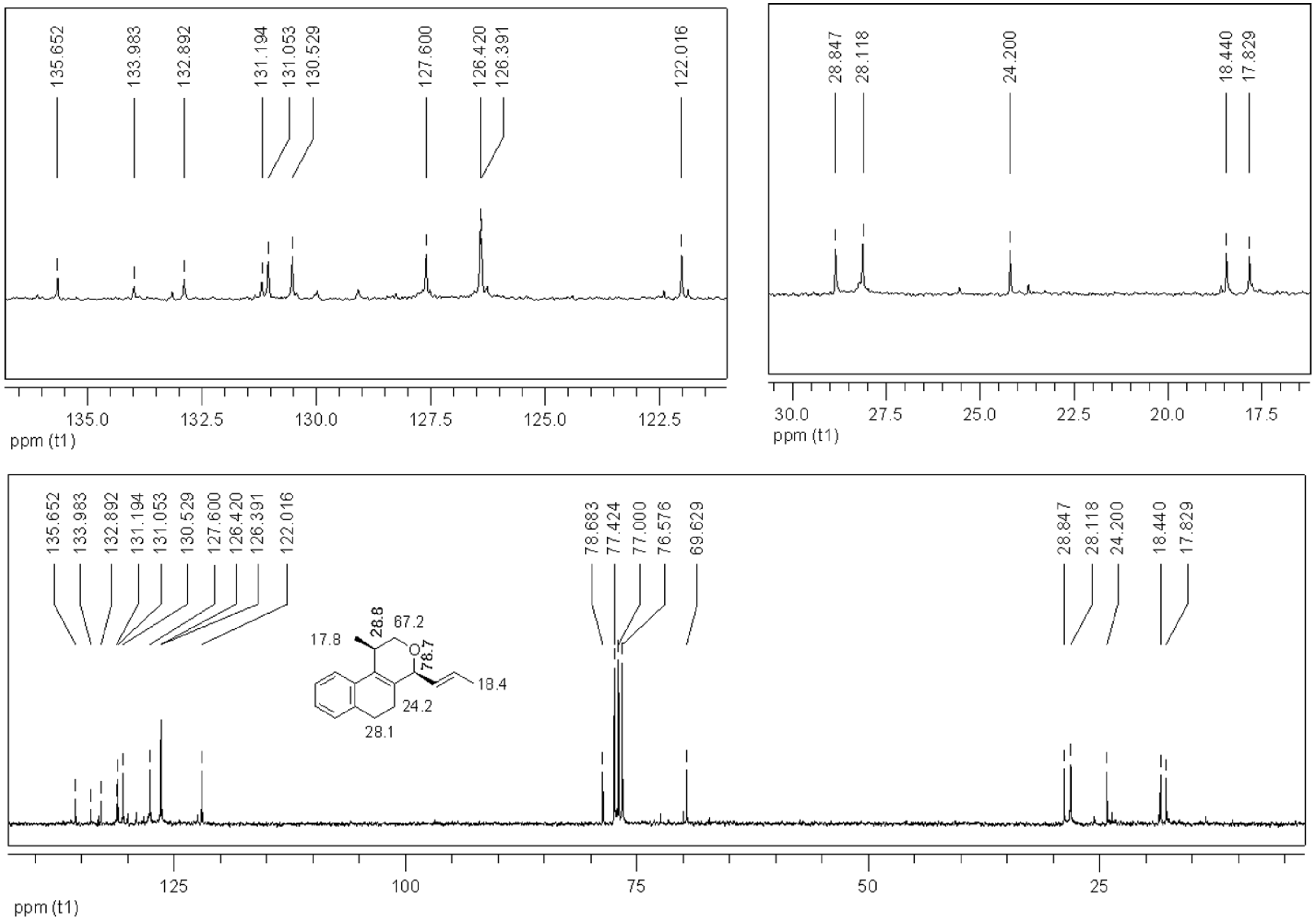

^{1}H-NMR of compound *trans*-**7t** (300 MHz, $CDCl_3$)

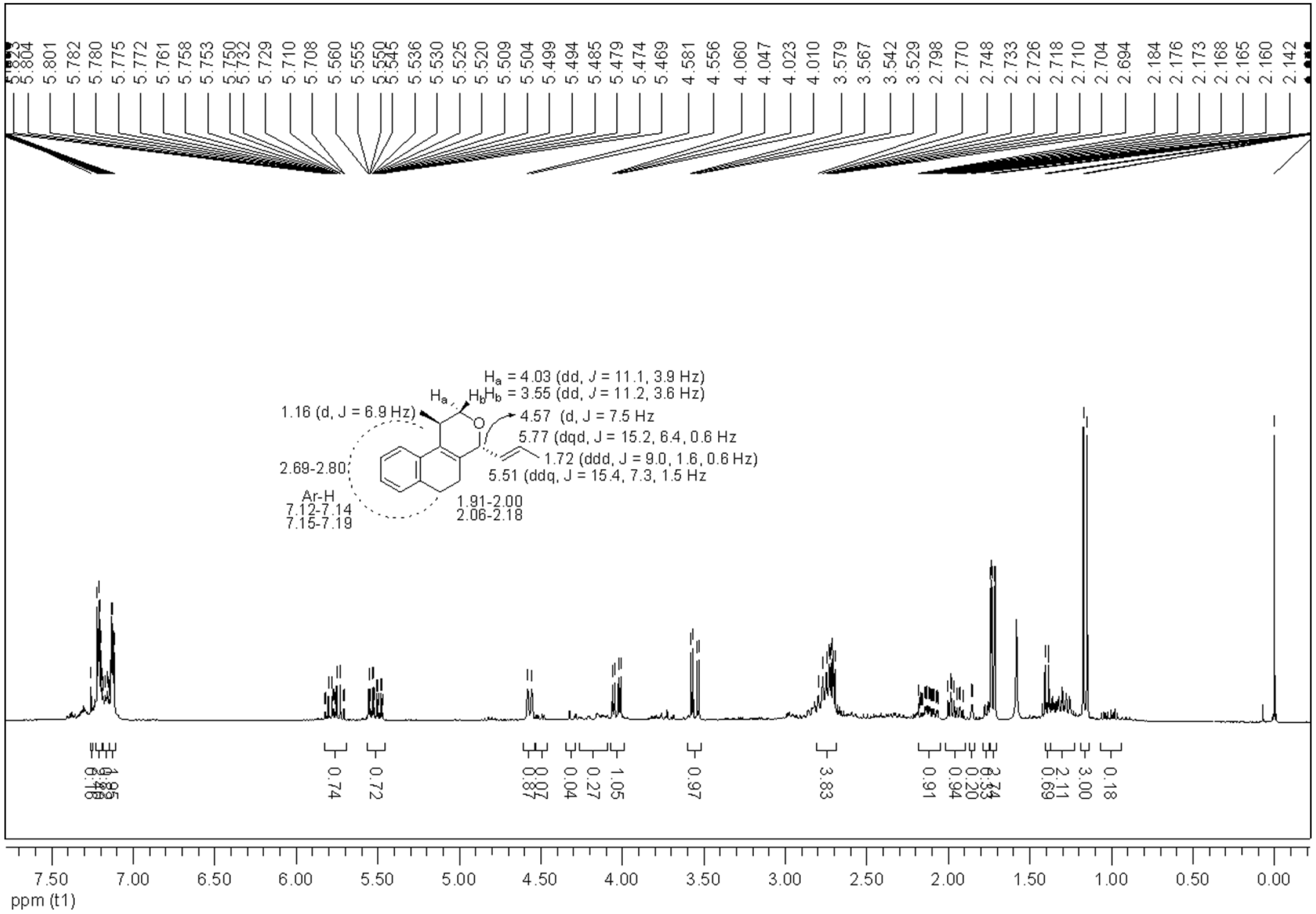

^{1}H-NMR of compound *trans*-**7t** (300 MHz, $CDCl_3$)

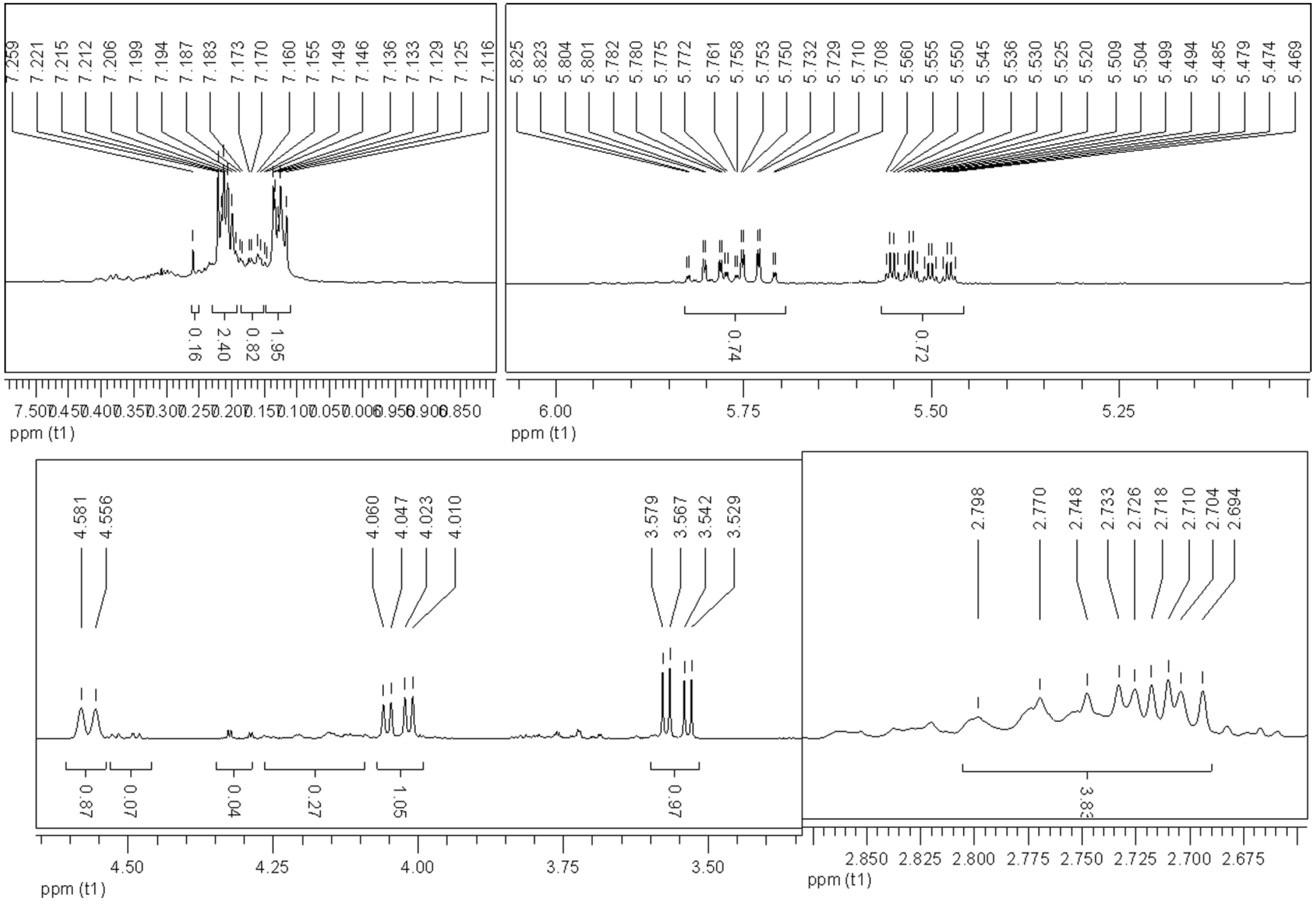

^{1}H-NMR of compound *trans*-**7t** (300 MHz, $CDCl_3$)

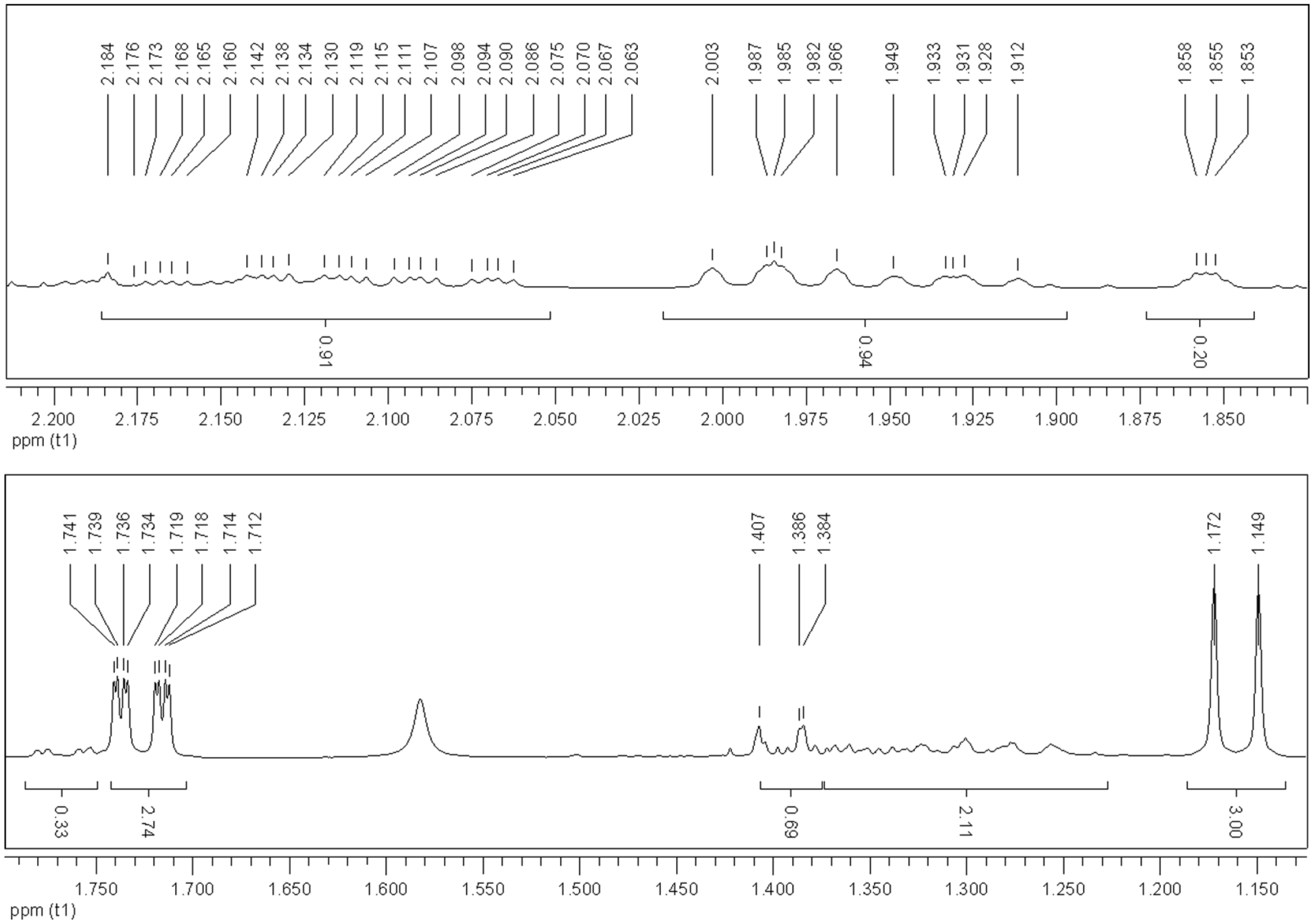

^{13}C-NMR of compound *trans*-**7t** (75 MHz, $CDCl_3$)

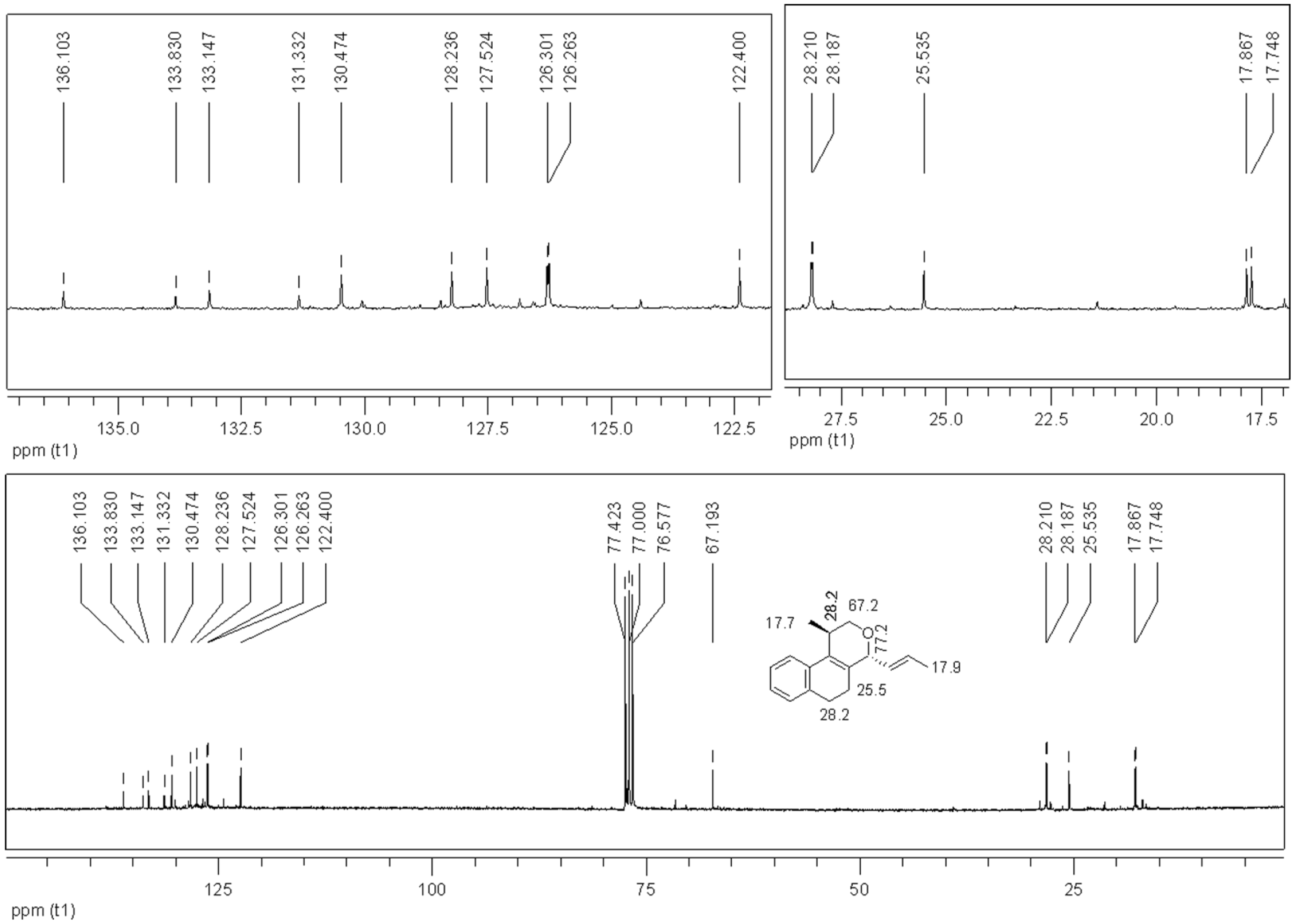

2D NOESY NMR of compound *trans*-**7t** (300 MHz, $CDCl_3$)

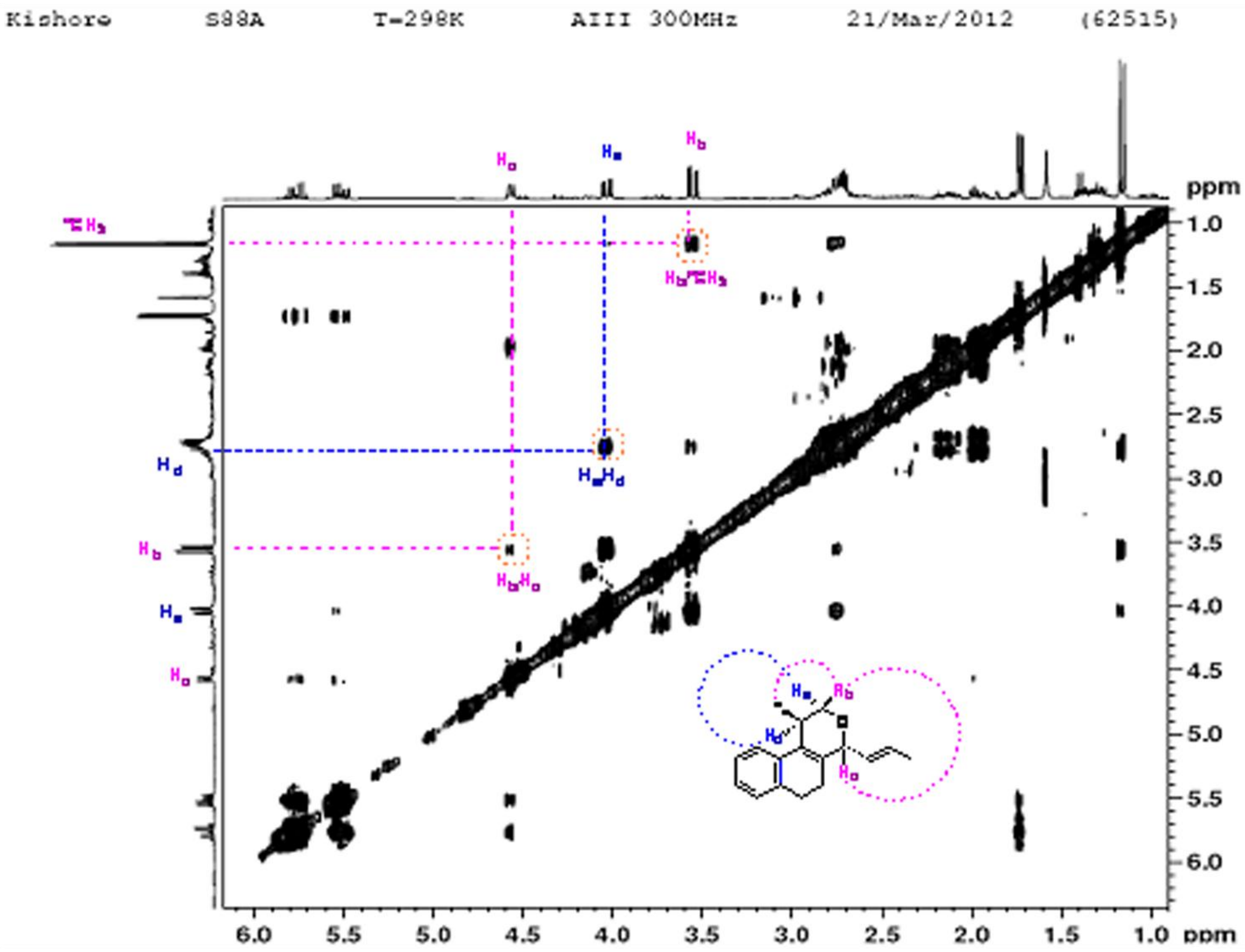

^{1}H-NMR of compound *cis*-**7u** (300 MHz, $CDCl_3$)

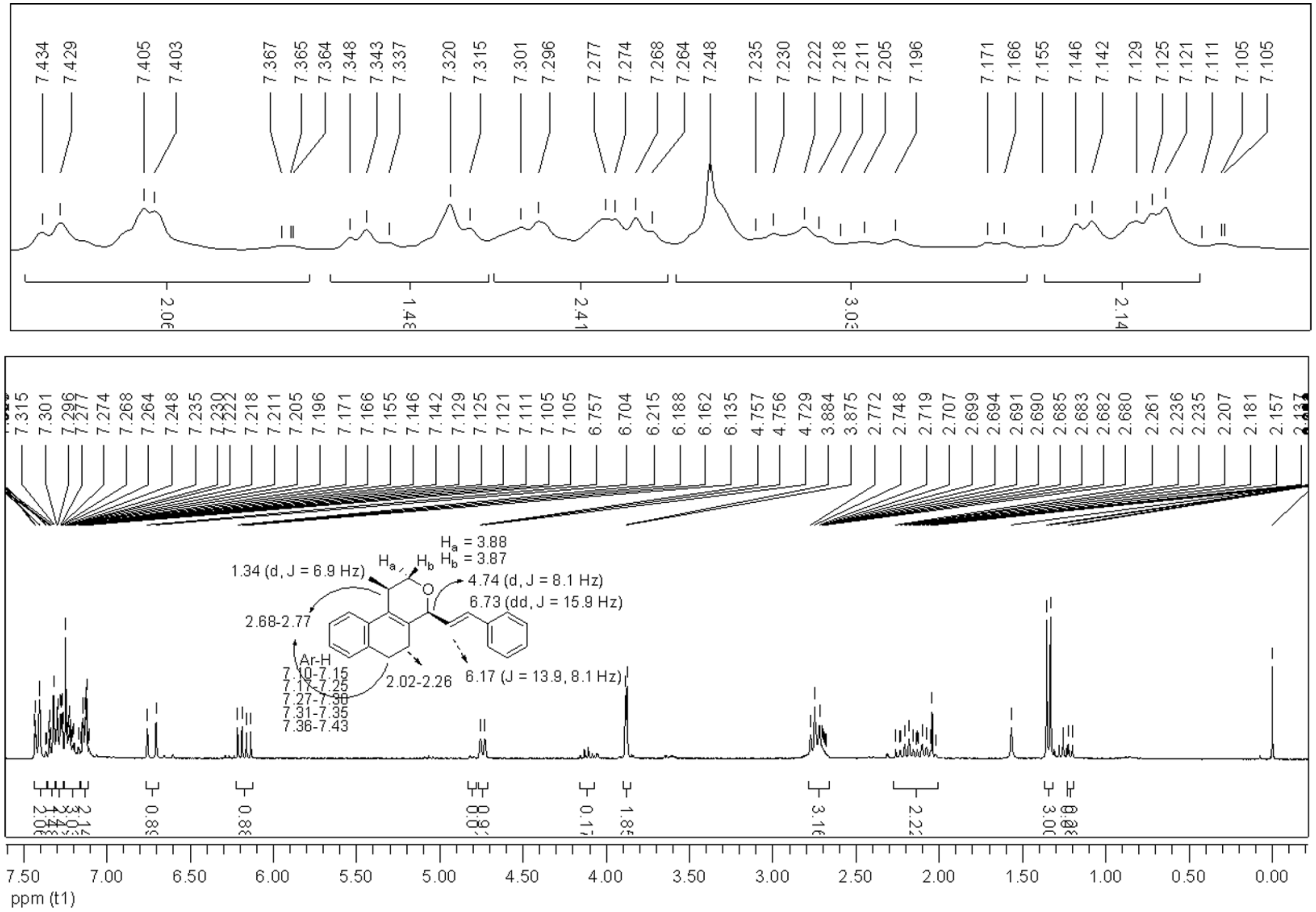

^{1}H-NMR of compound *cis*-**7u** (300 MHz, $CDCl_3$)

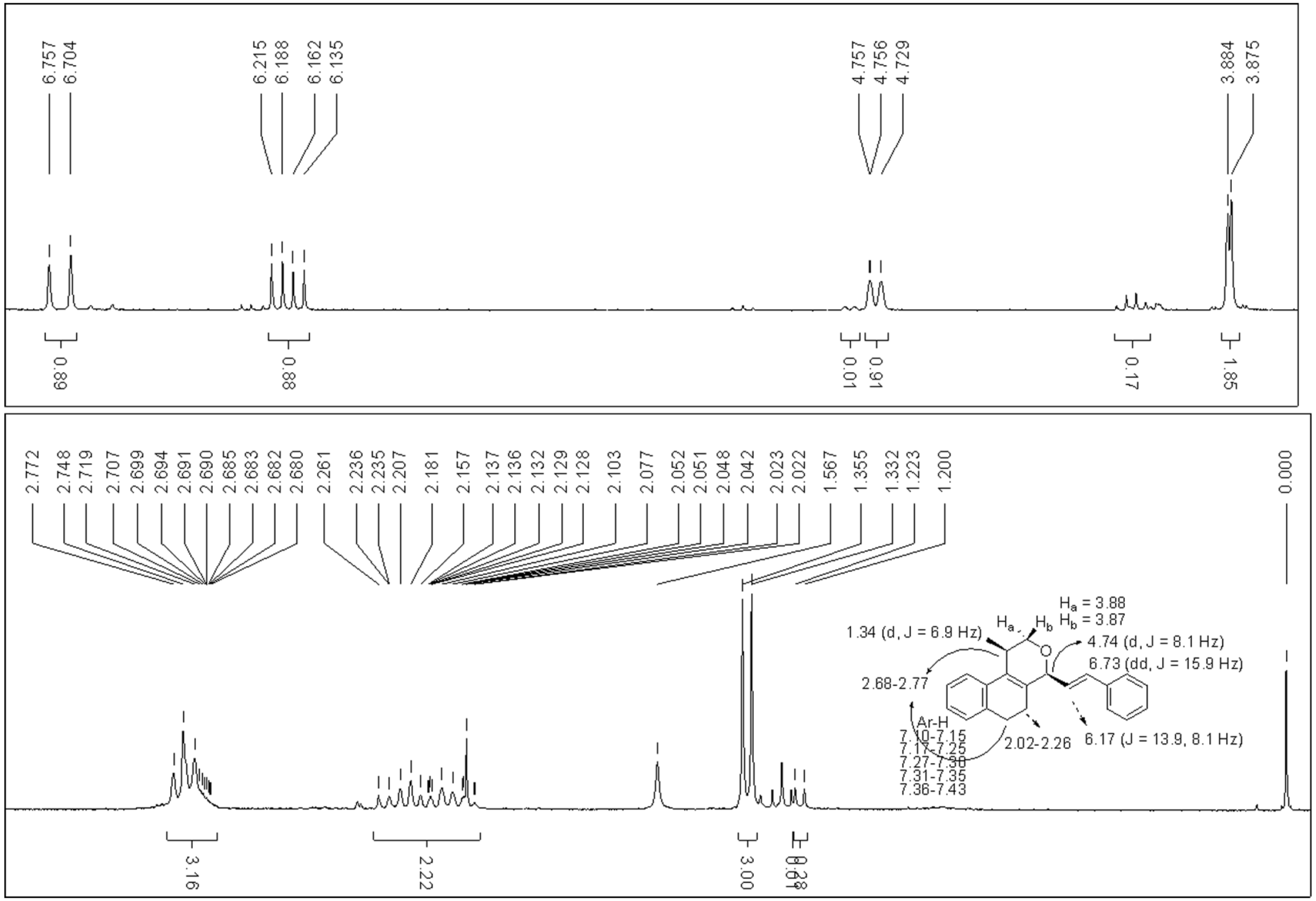

^{13}C-NMR of compound *cis*-**7u** (75 MHz, $CDCl_3$)

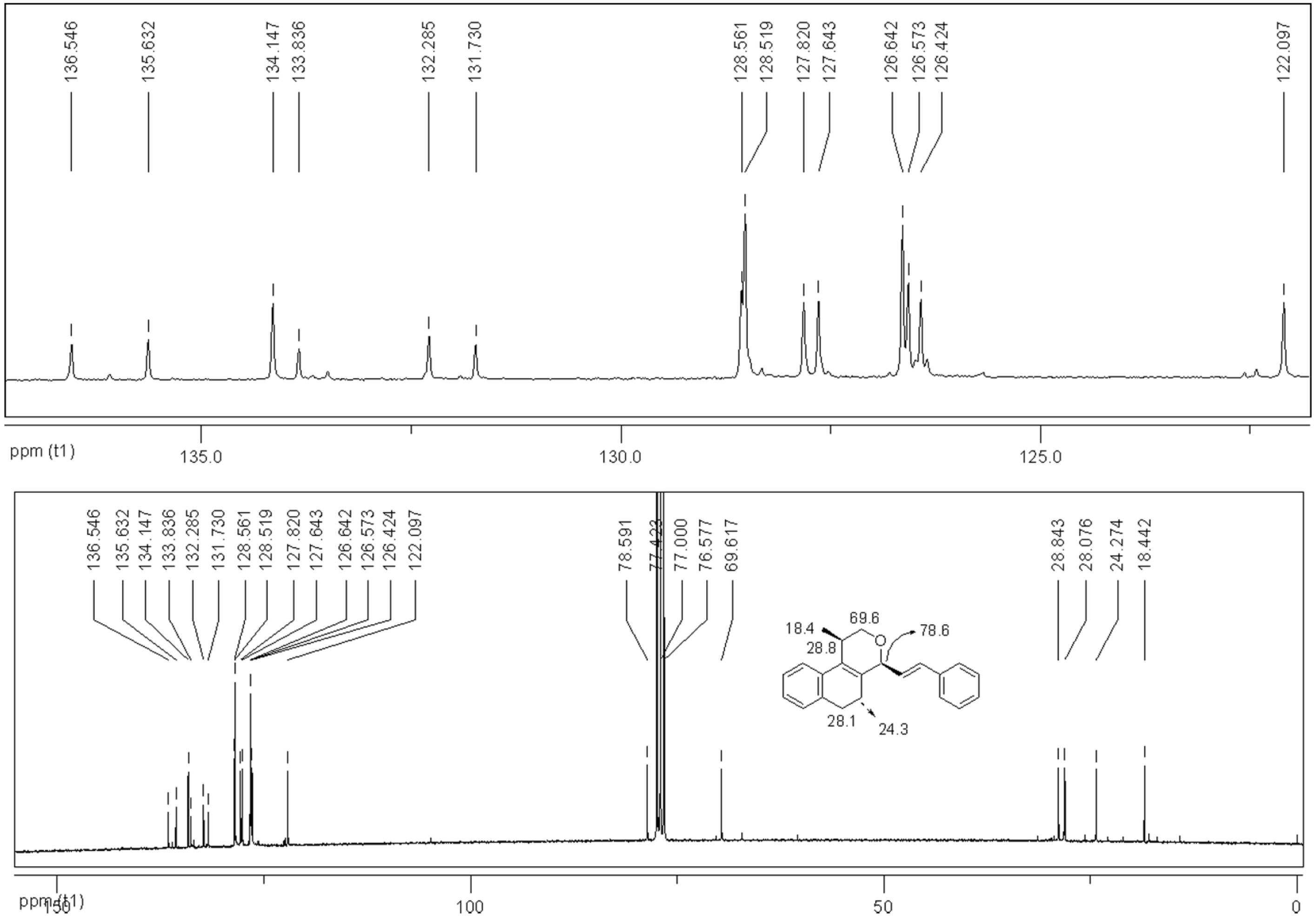

^{1}H-NMR of compound *trans*-**7u** (500 MHz, $CDCl_3$)

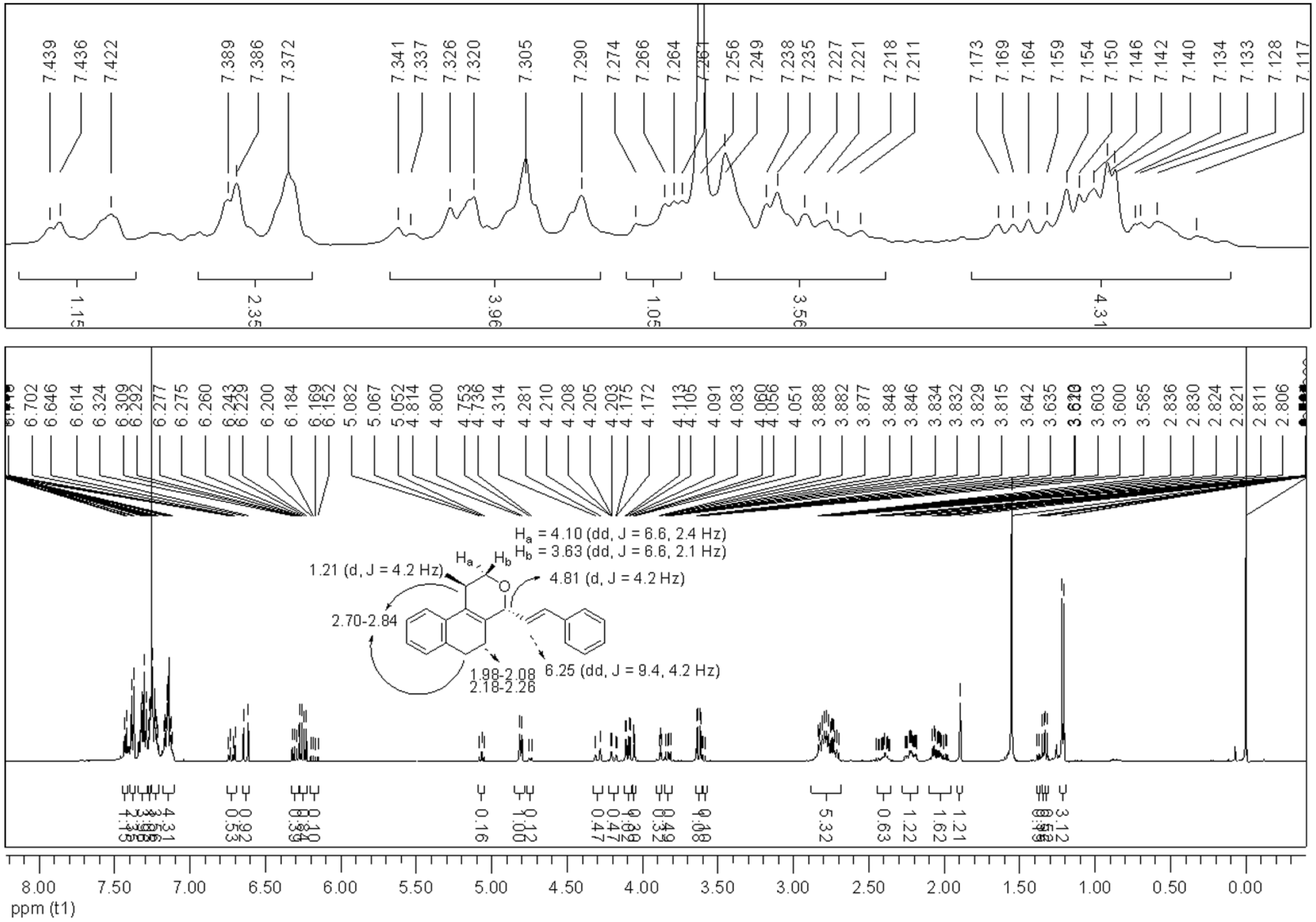

^{1}H-NMR of compound *trans*-**7u** (500 MHz, $CDCl_3$)

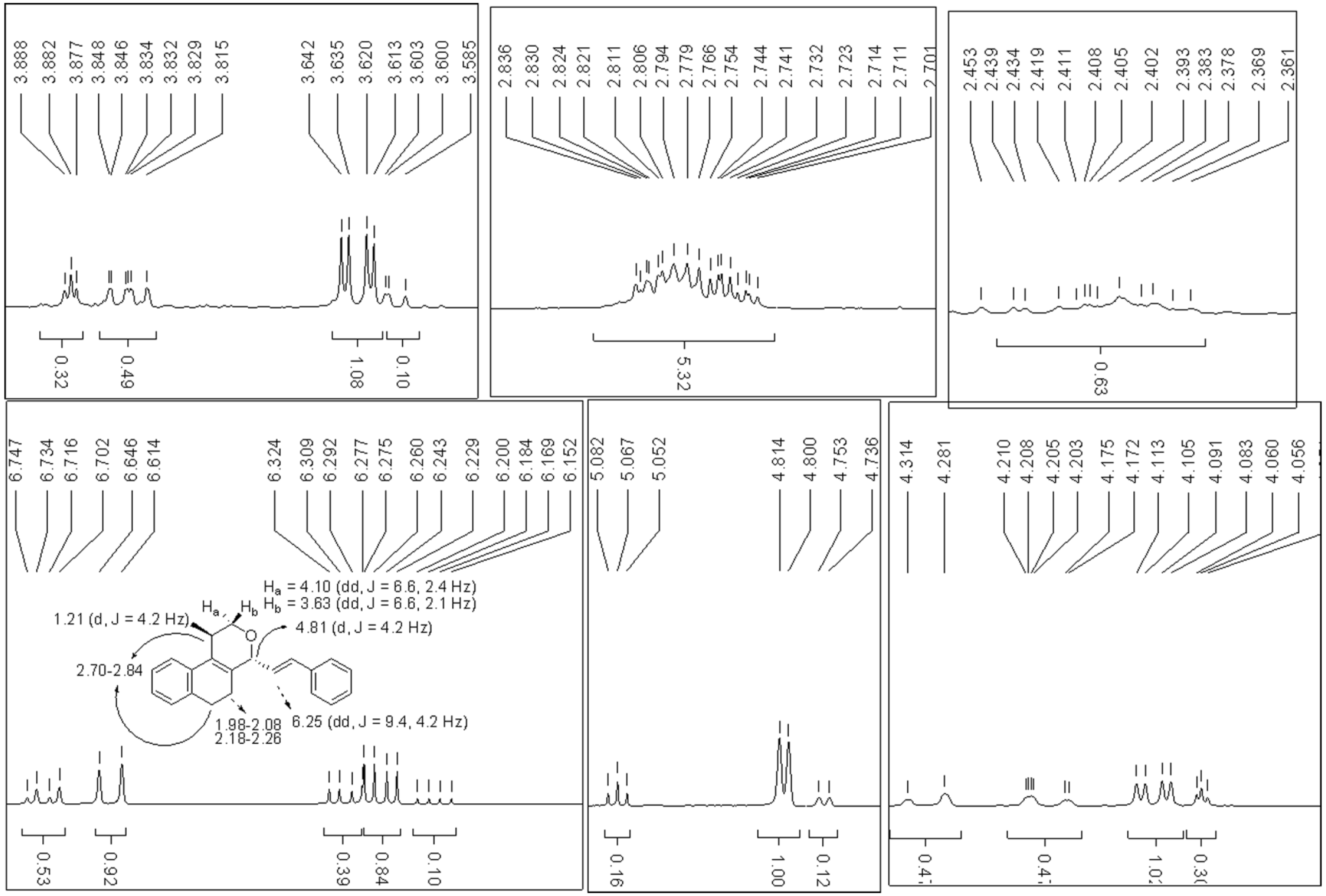

^{1}H-NMR of compound *trans*-**7u** (500 MHz, $CDCl_3$)

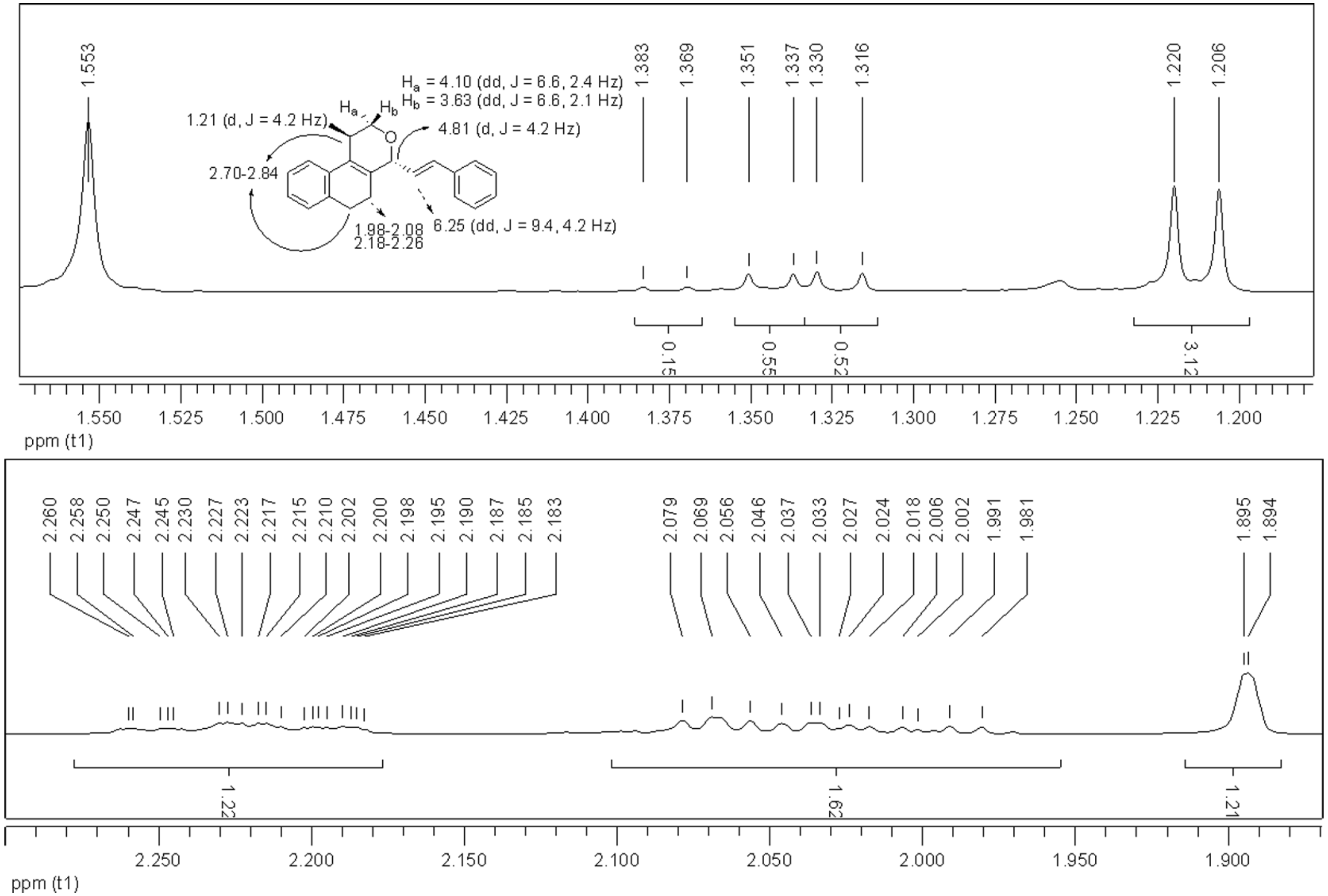

^{13}C-NMR of compound *trans*-**7u** (50 MHz, $CDCl_3$)

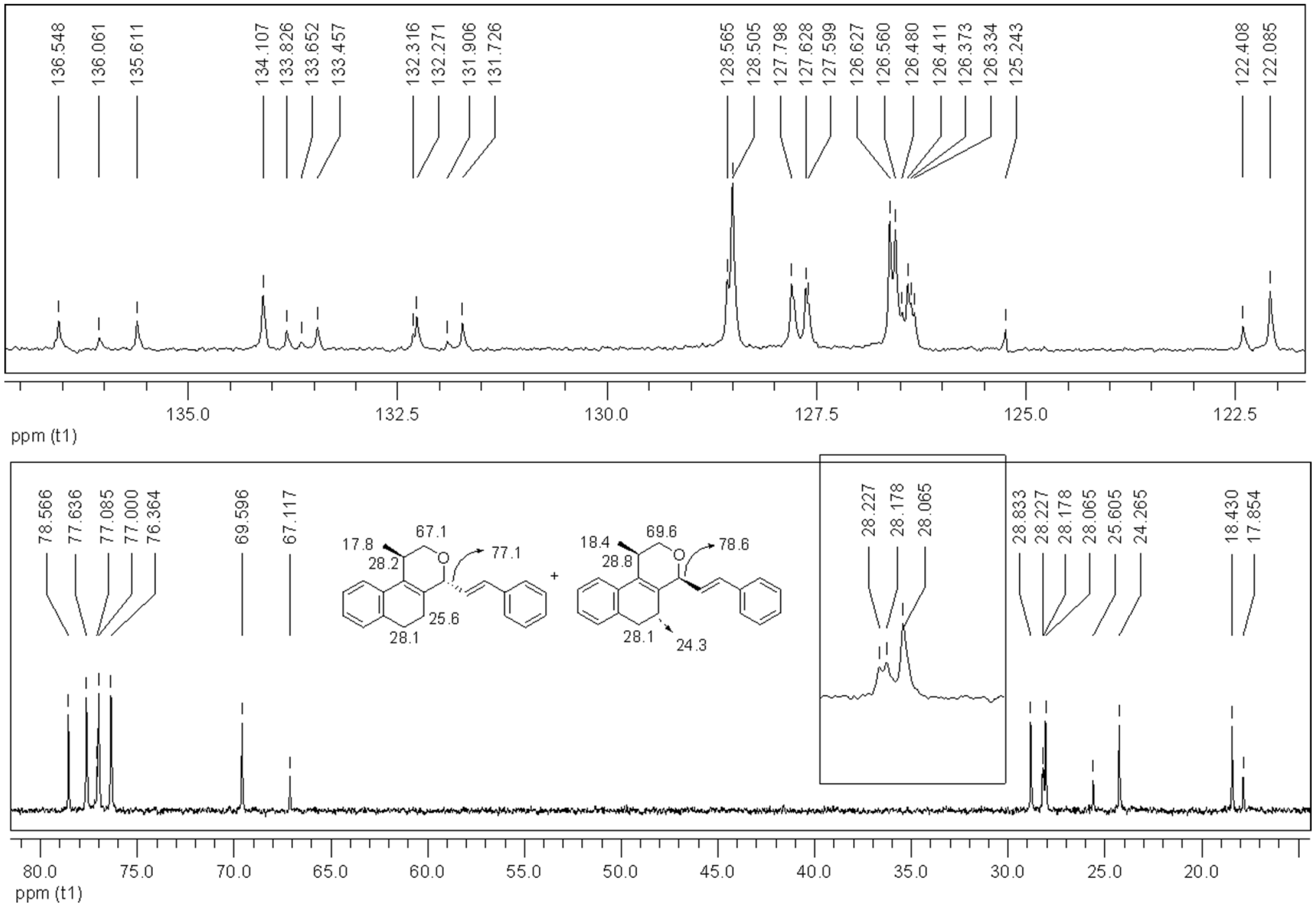

2D NOESY NMR of compound *trans*-**7u** (500 MHz, $CDCl_3$)

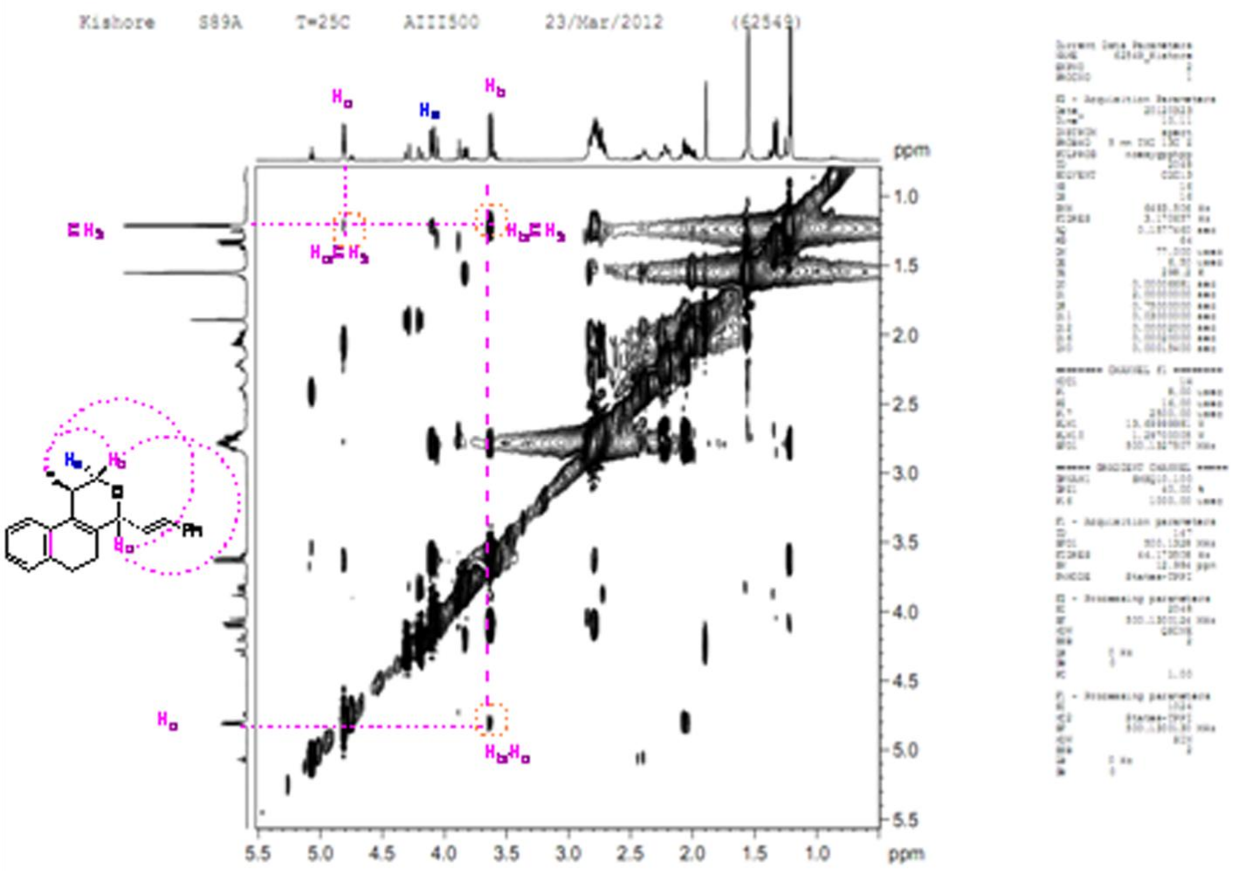

^{1}H-NMR of compounds *cis*-**13b** and *trans*-**13b** (300 MHz, $CDCl_3$)

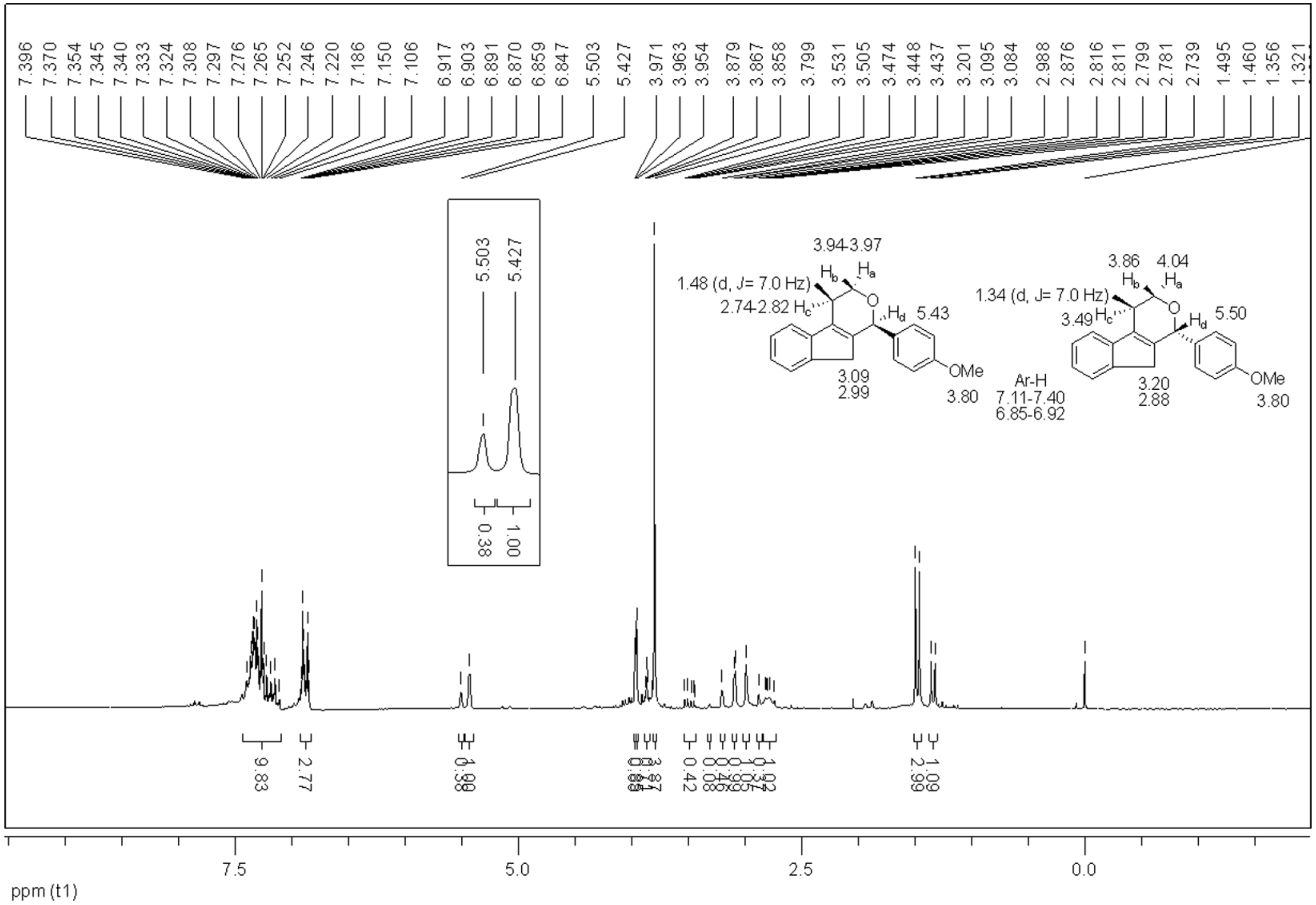

^{1}H-NMR of compounds *cis*-**13b** and *trans*-**13b** (300 MHz, $CDCl_3$)

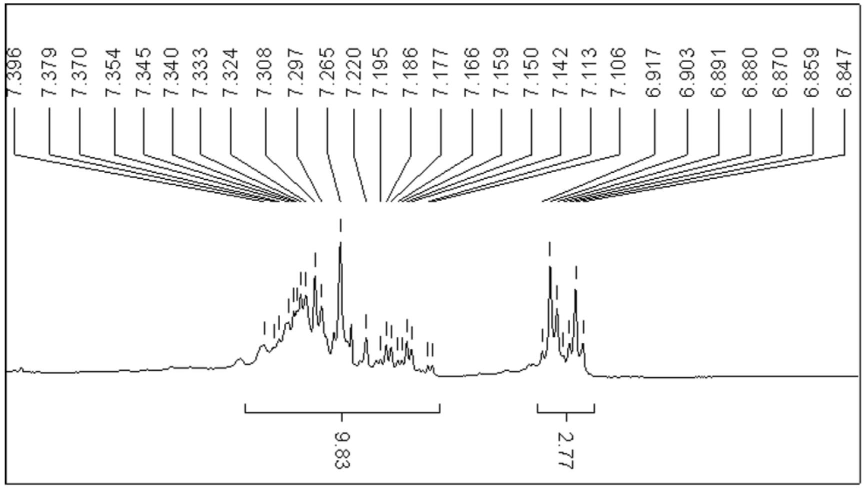

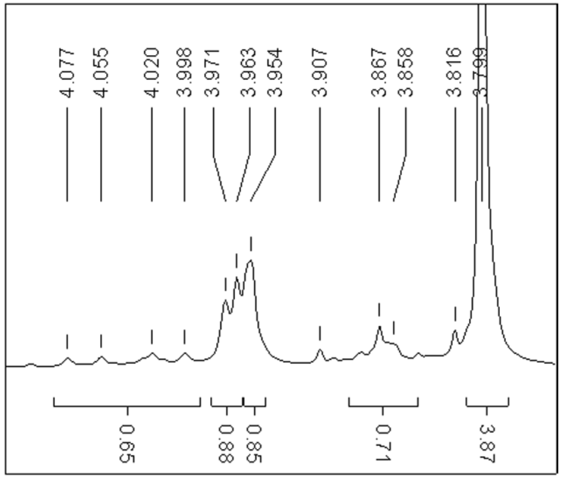

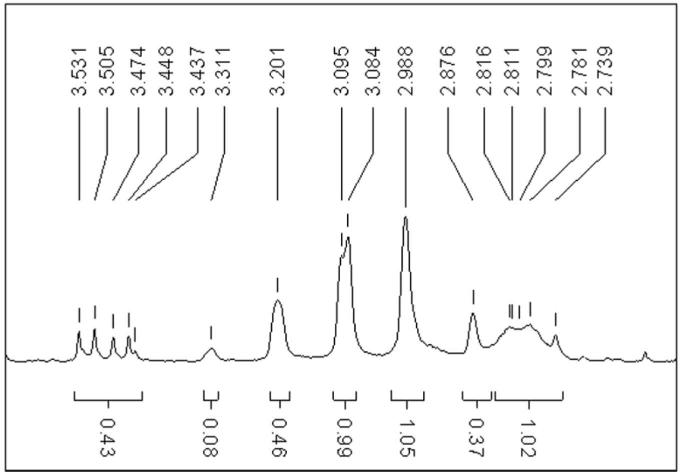

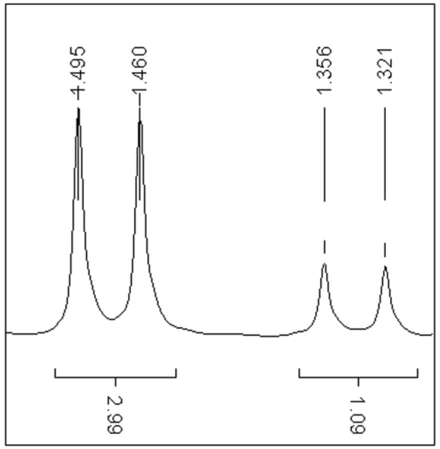

^{13}C-NMR of compounds *cis*-**13b** and *trans*-**13b** (75 MHz, $CDCl_3$)

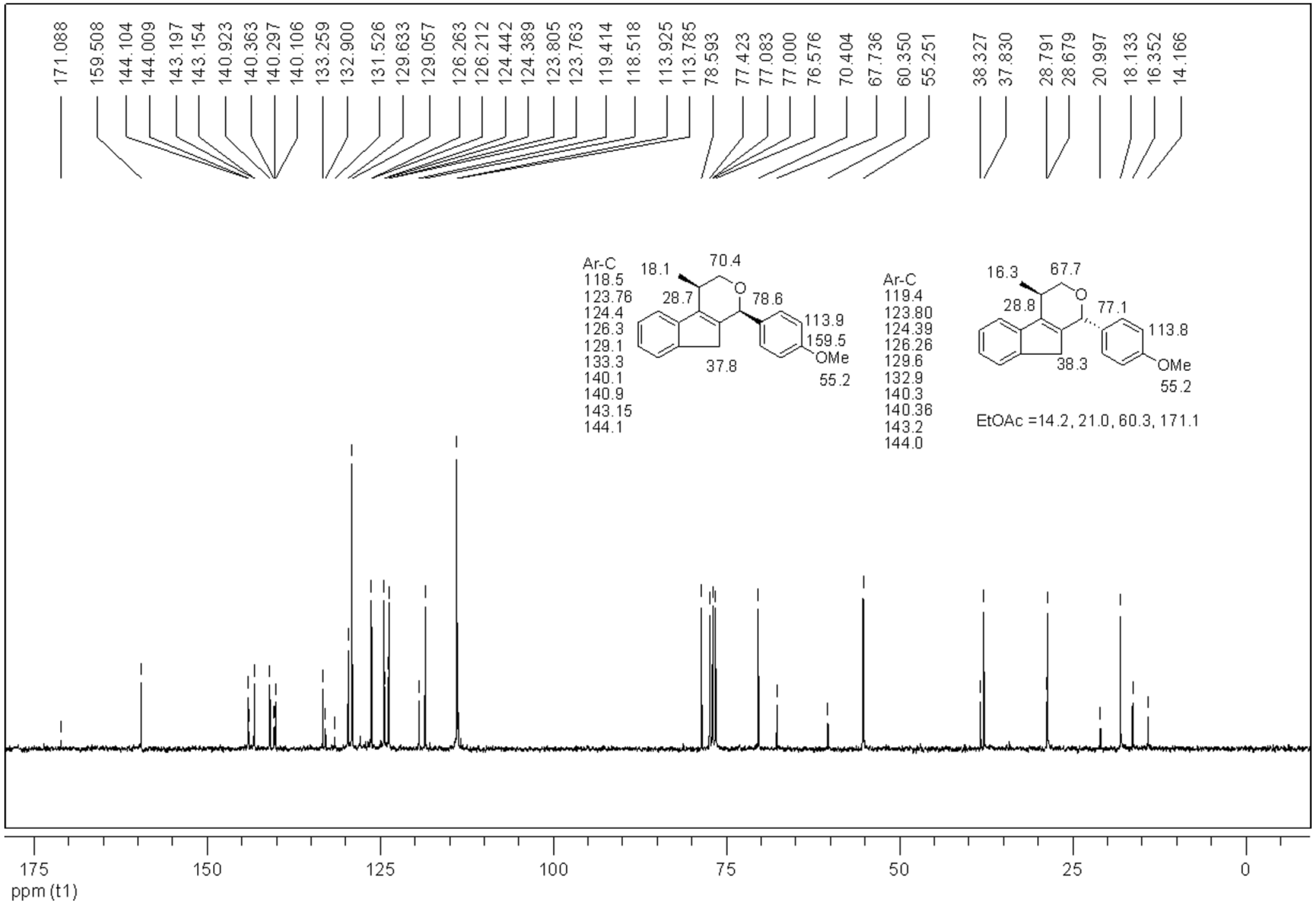

^{13}C-NMR of compounds *cis*-**13b** and *trans*-**13b** (75 MHz, $CDCl_3$)

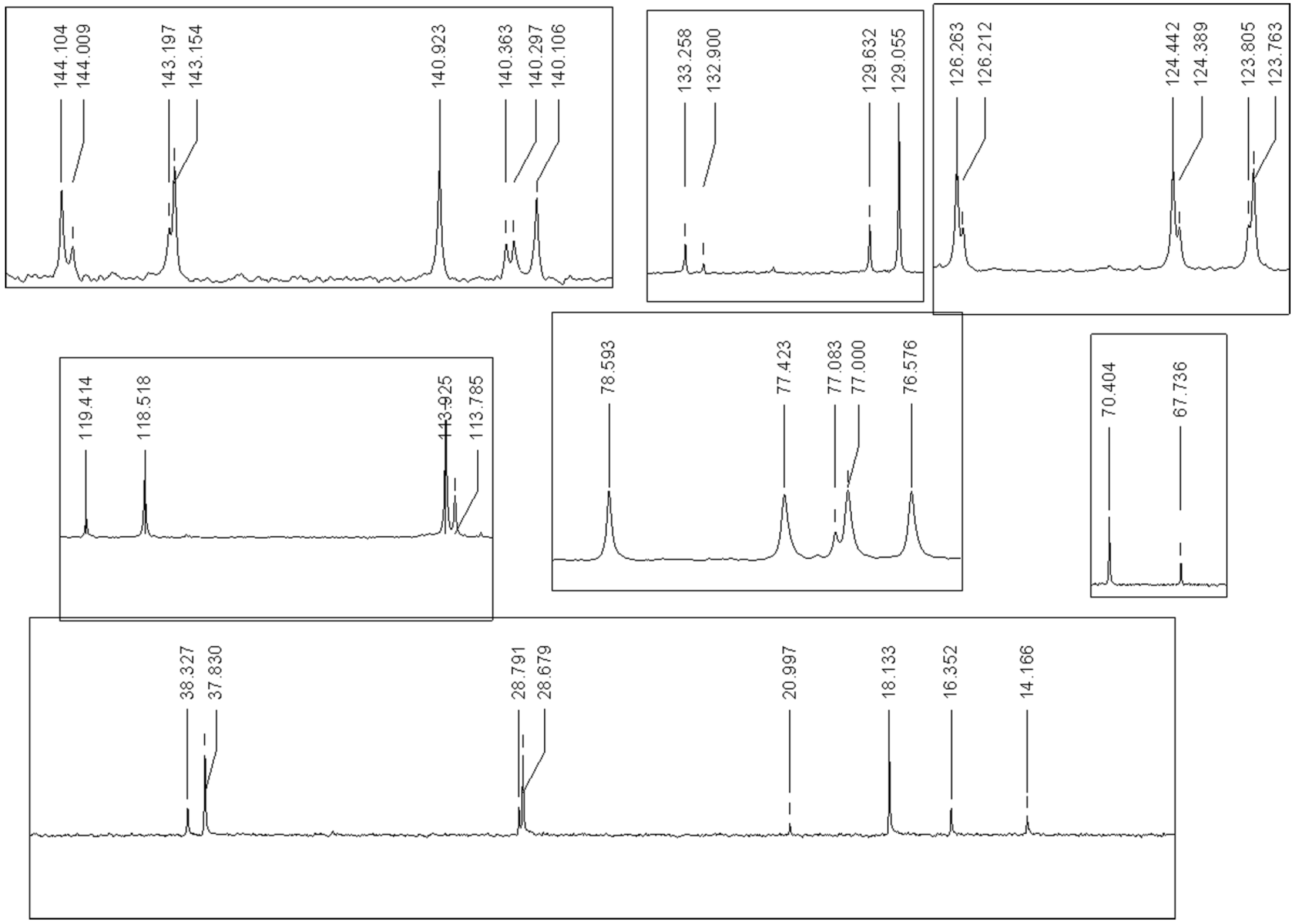

2D NOESY NMR of compound *cis*-**13b** (300 MHz, $CDCl_3$)

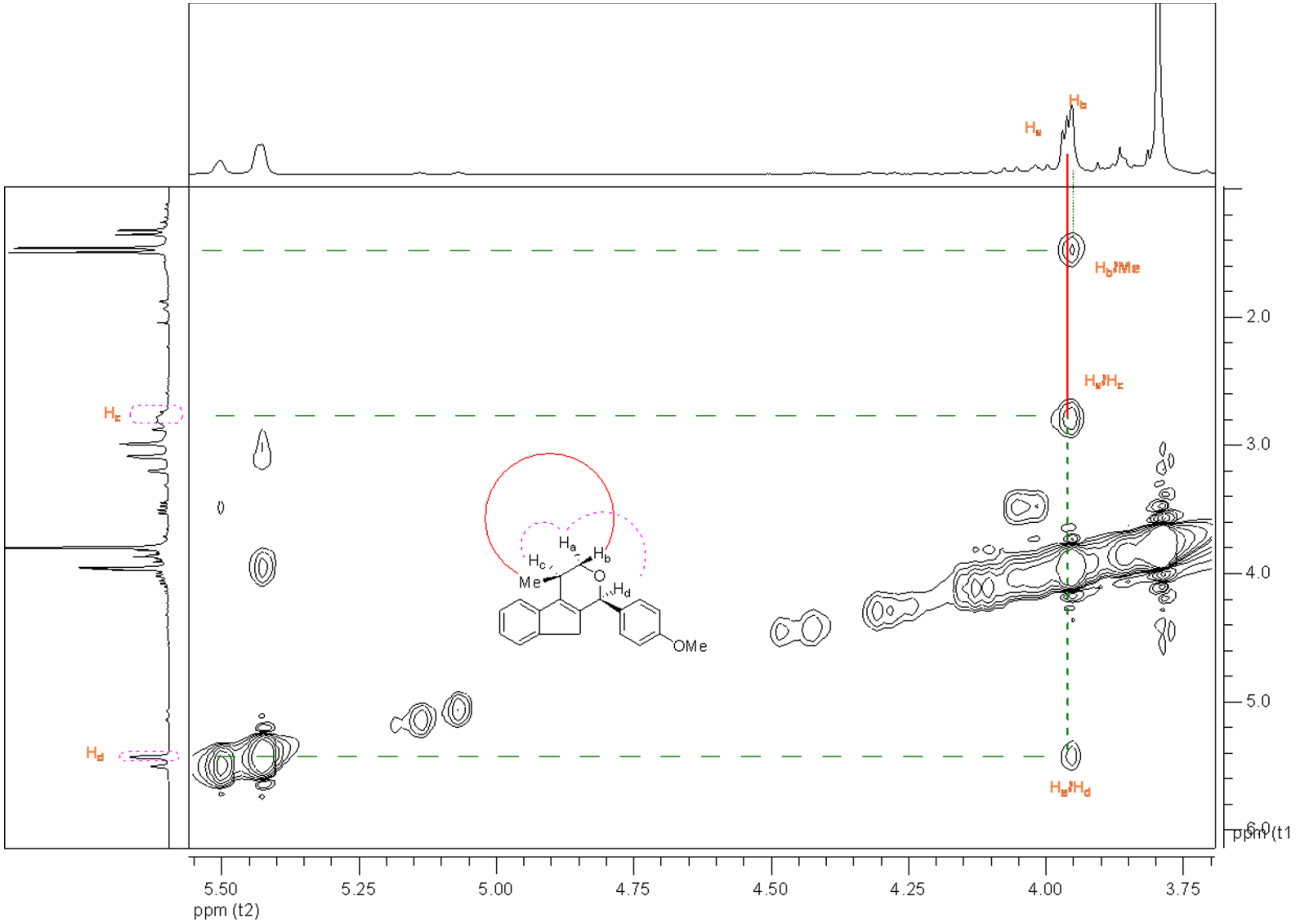

^{1}H-NMR of compound *cis*-**14c** (200 MHz, $CDCl_3$)

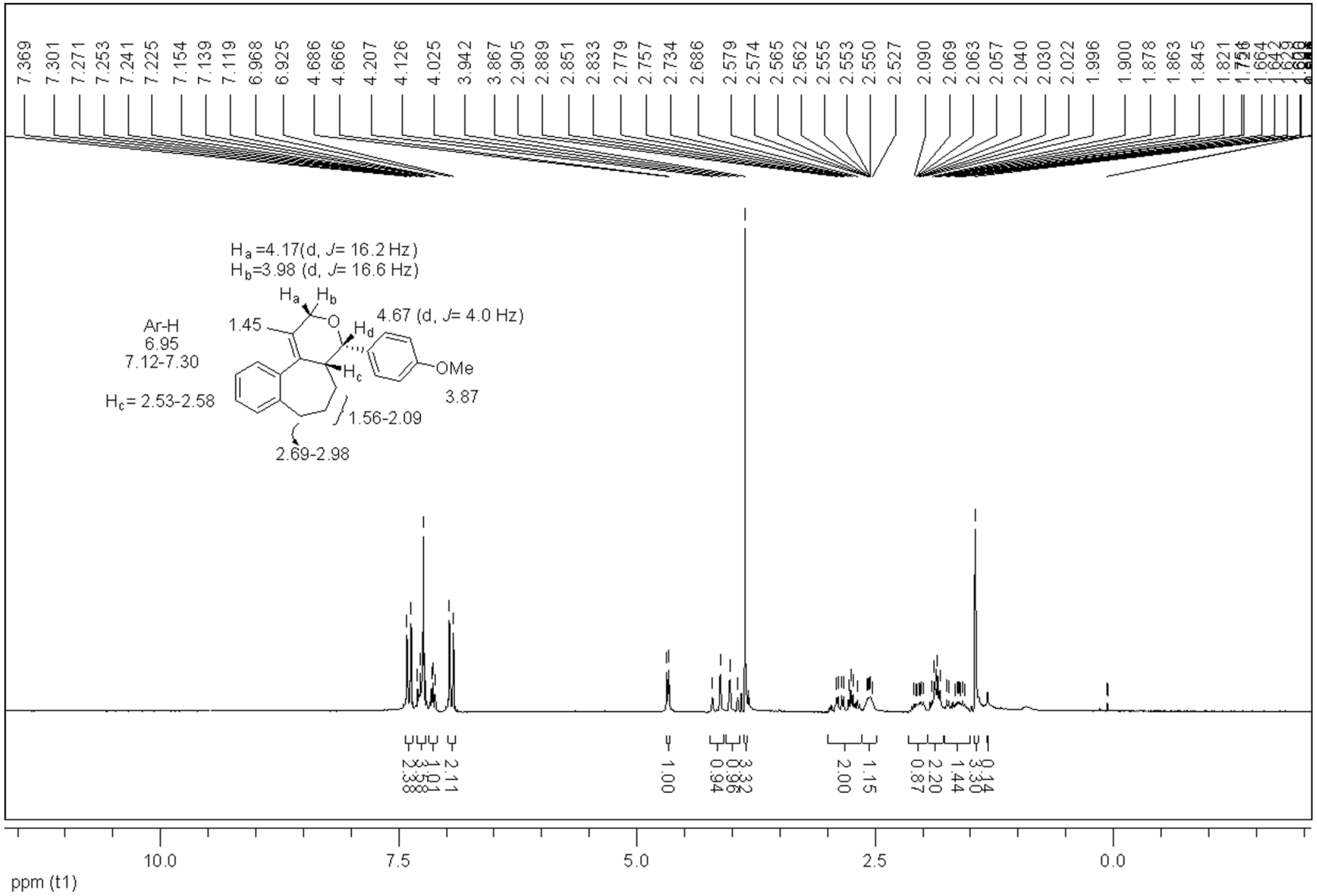

^{1}H-NMR of compound *cis*-**14c** (200 MHz, $CDCl_3$)

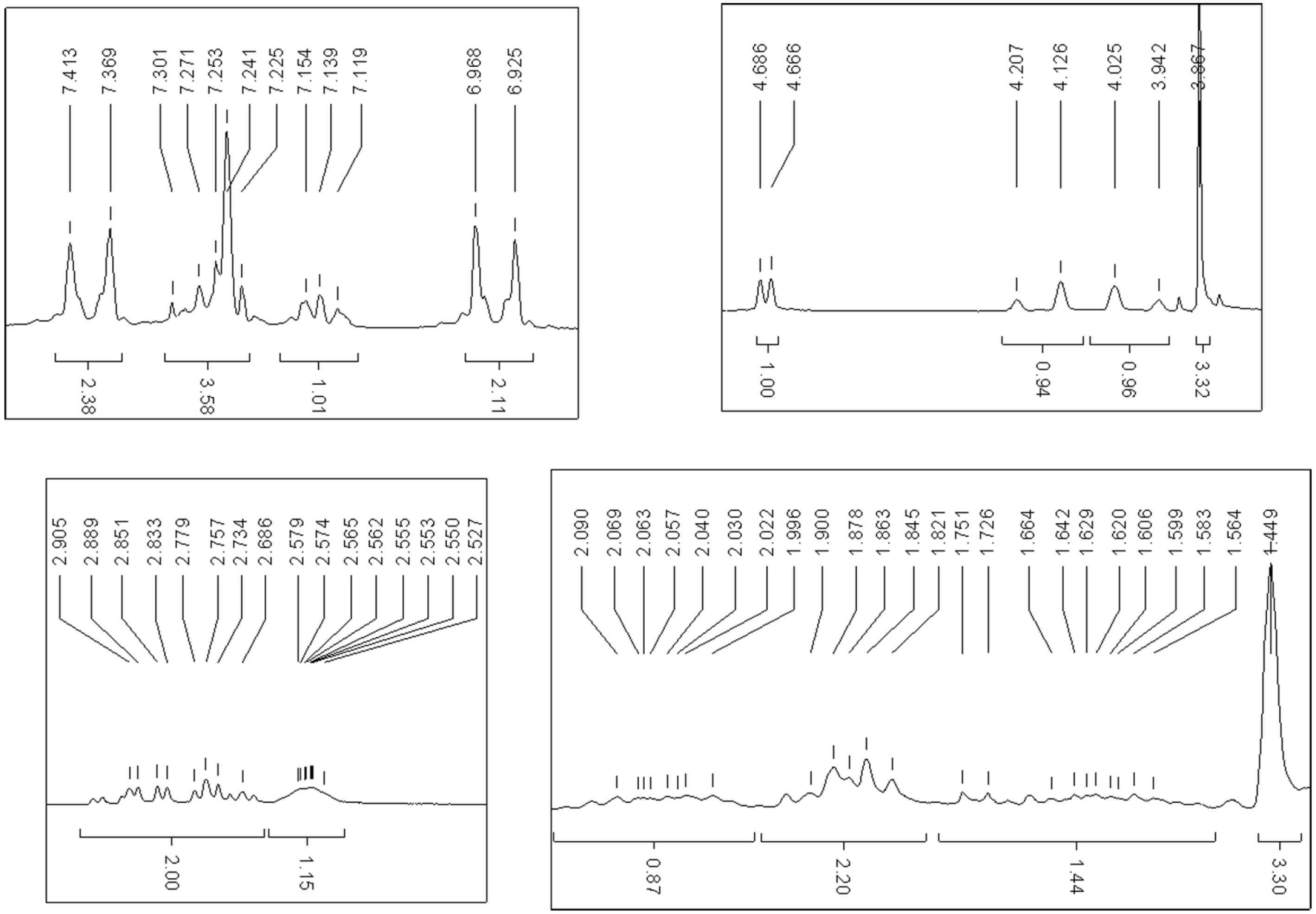

^{13}C-NMR of compound *cis*-**14c** (50 MHz, $CDCl_3$)

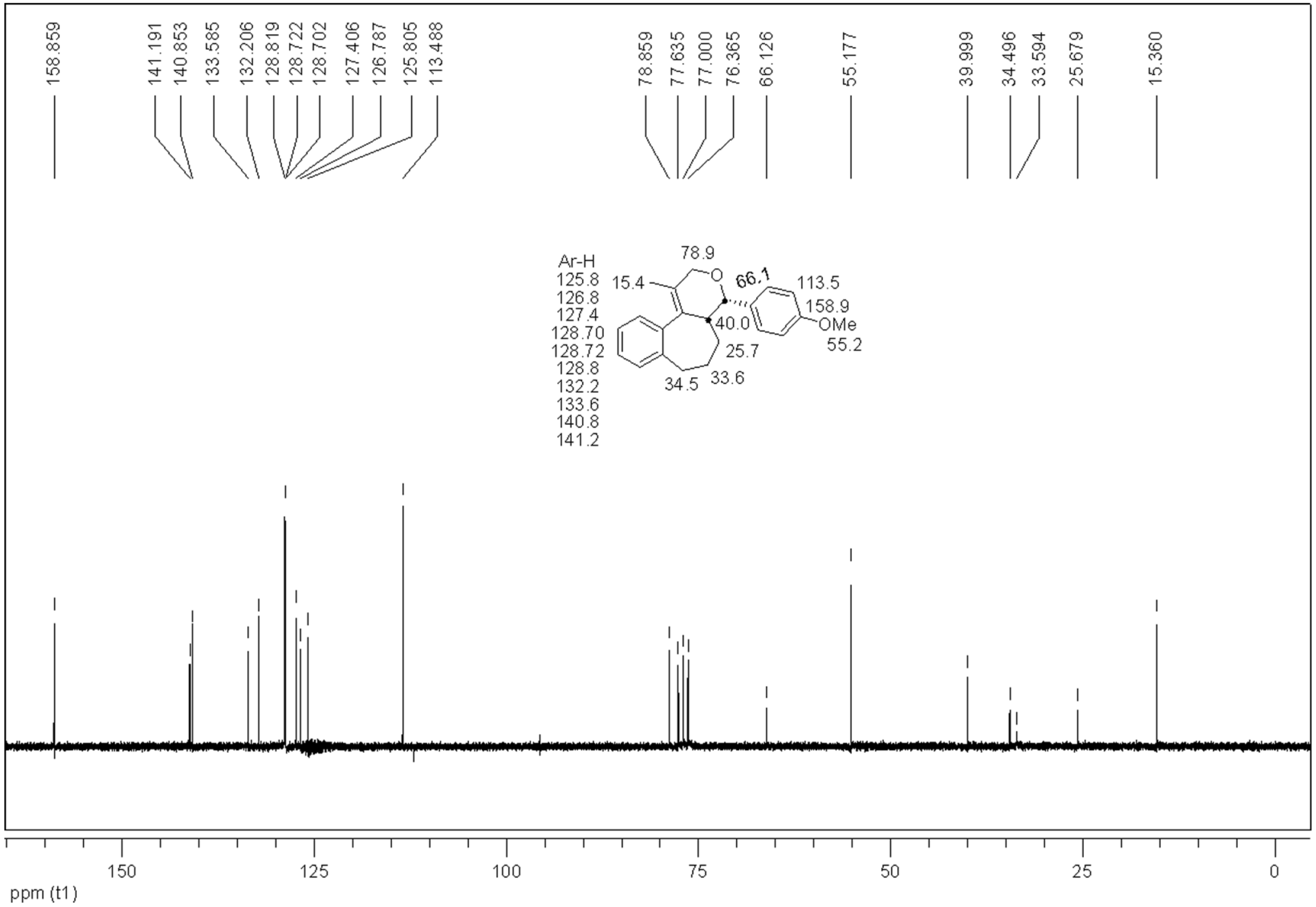

2D NOESY NMR of compound *cis*-14c (300 MHz, $CDCl_3$)

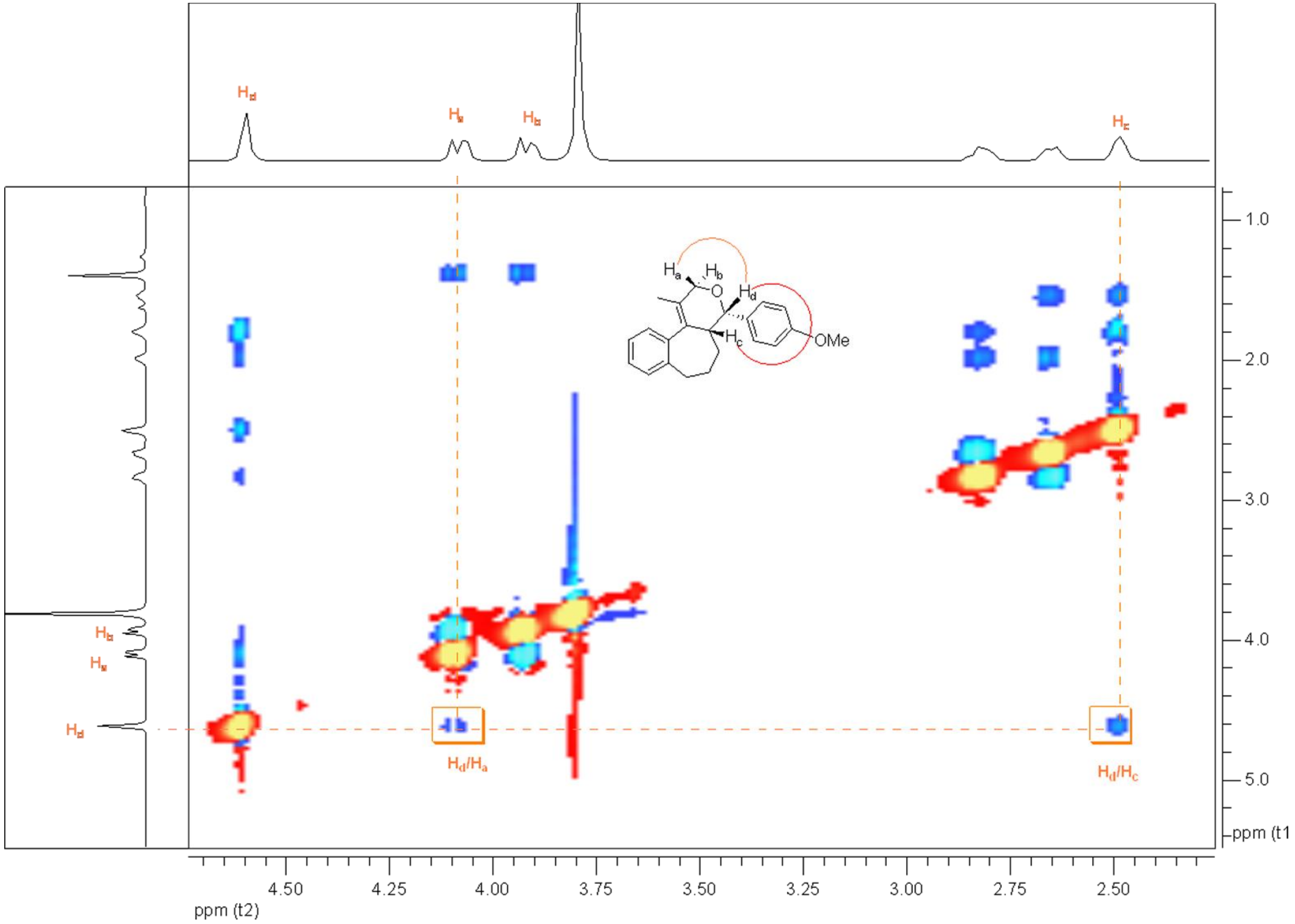

^{1}H-NMR of compound *trans*-**14c** (200 MHz, $CDCl_3$)

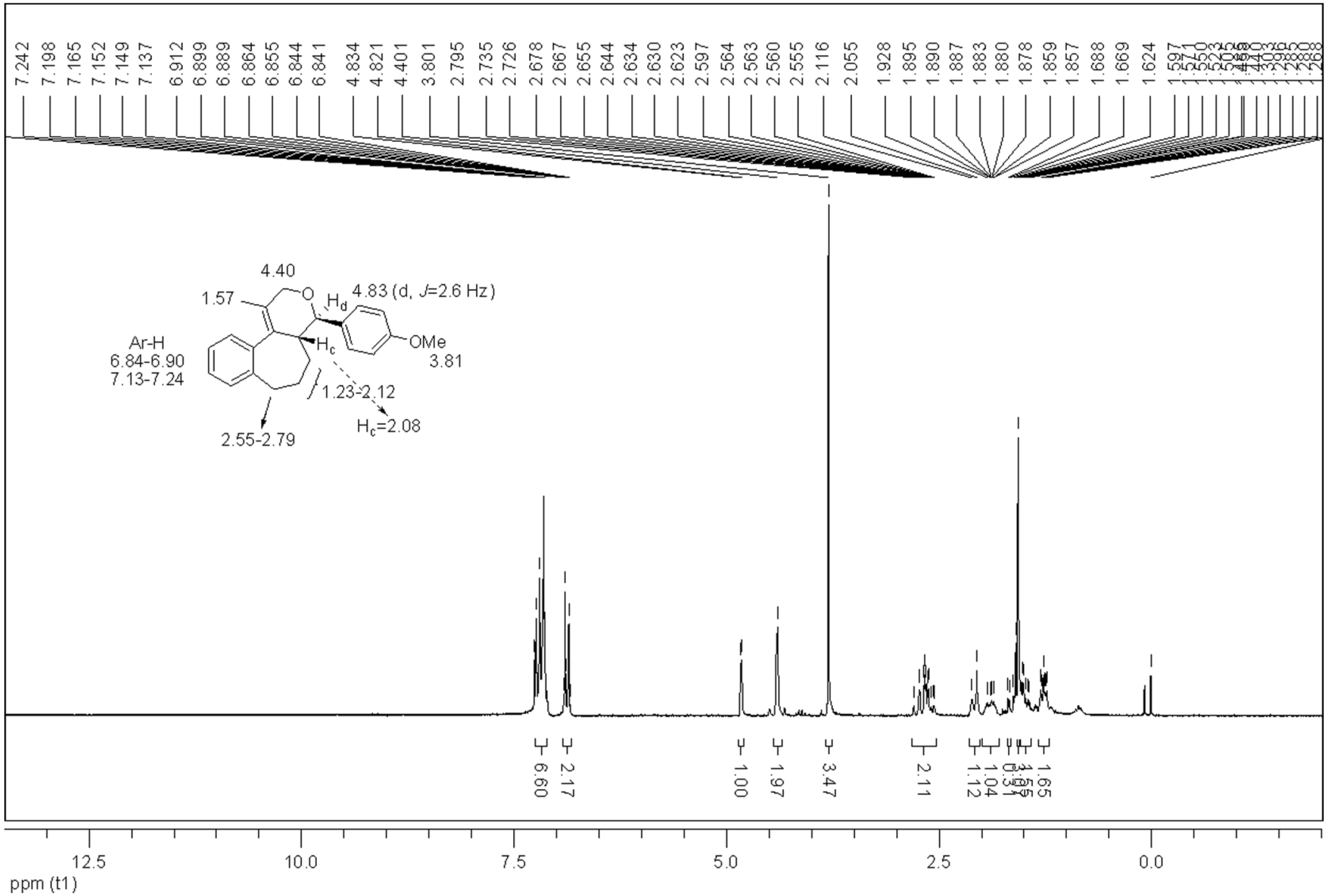

^{1}H-NMR of compound *trans*-**14c** (200 MHz, $CDCl_3$)

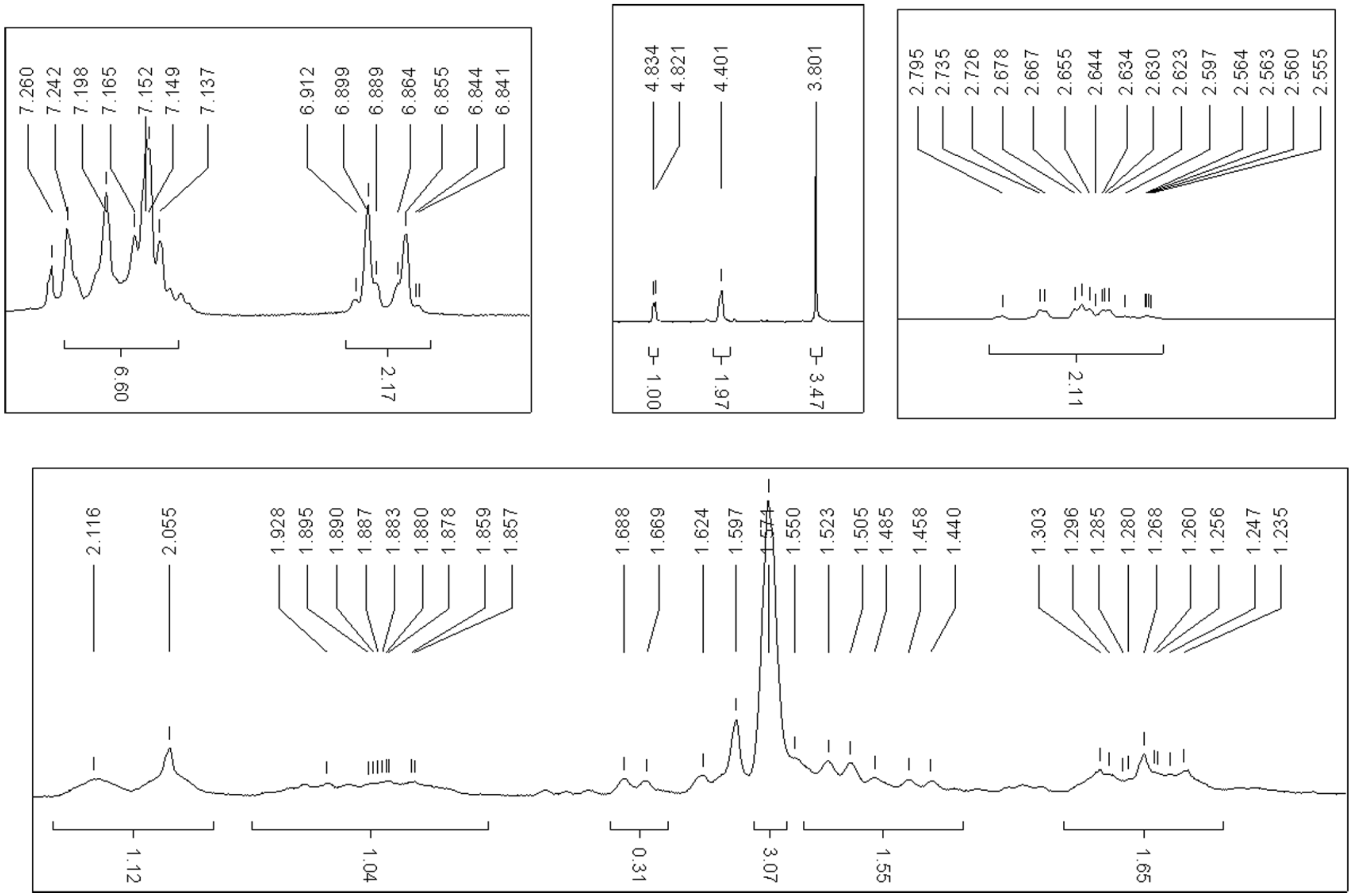

^{13}C-NMR of compound *trans*-**14c** (50 MHz, $CDCl_3$)

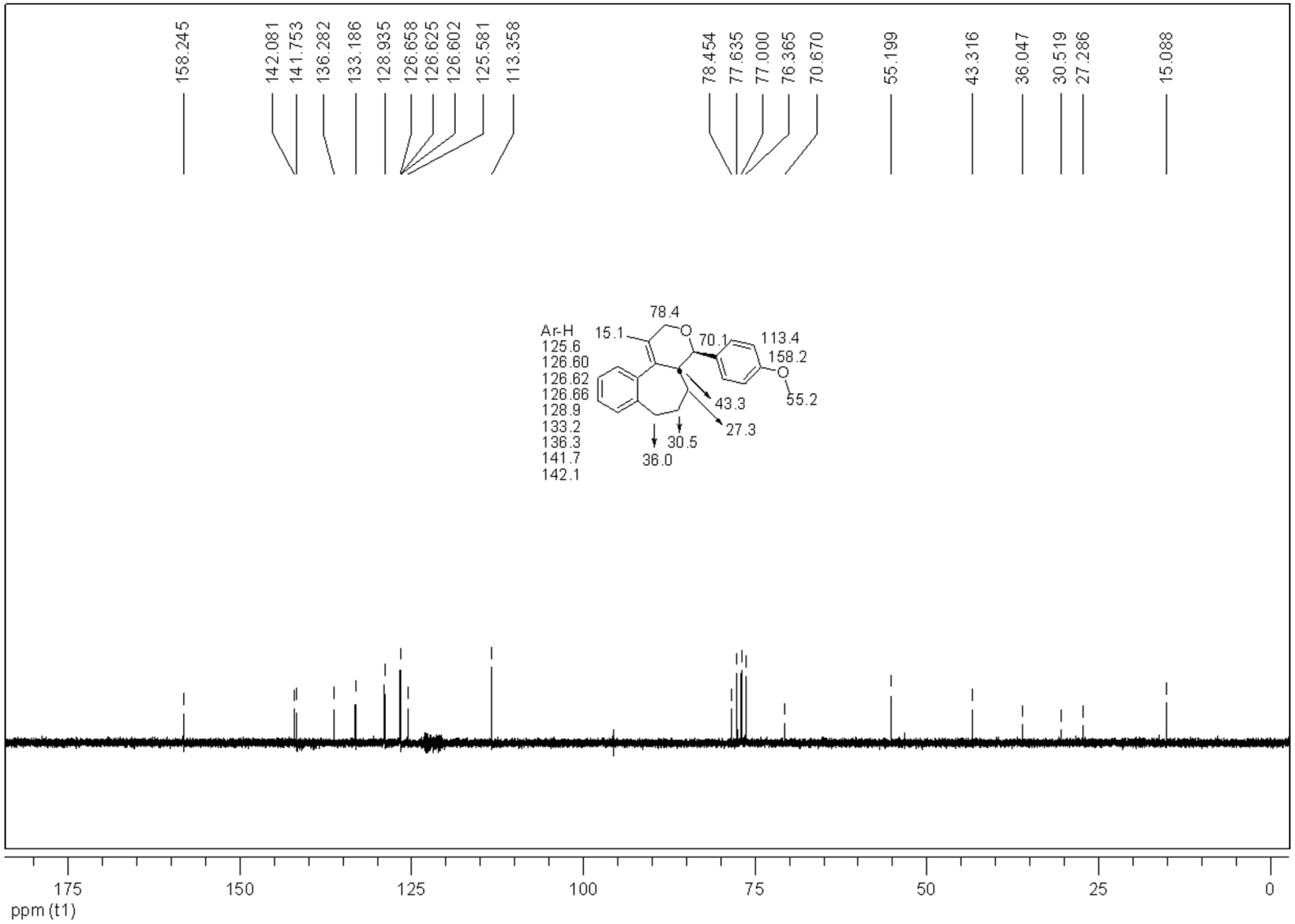

^{1}H-NMR of compound **13d** (200 MHz, $CDCl_3$)

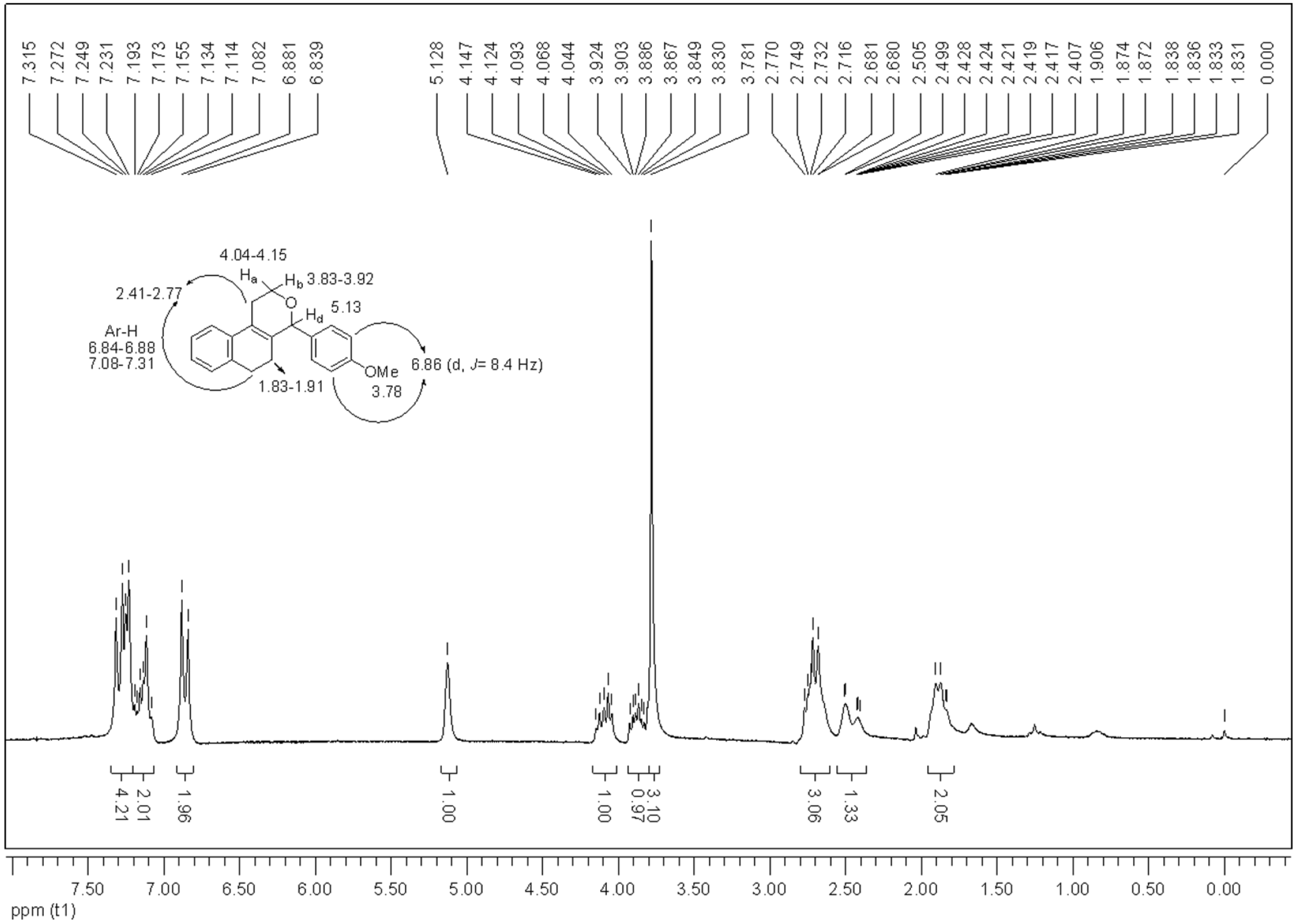

^{1}H-NMR of compound **13d** (200 MHz, $CDCl_3$)

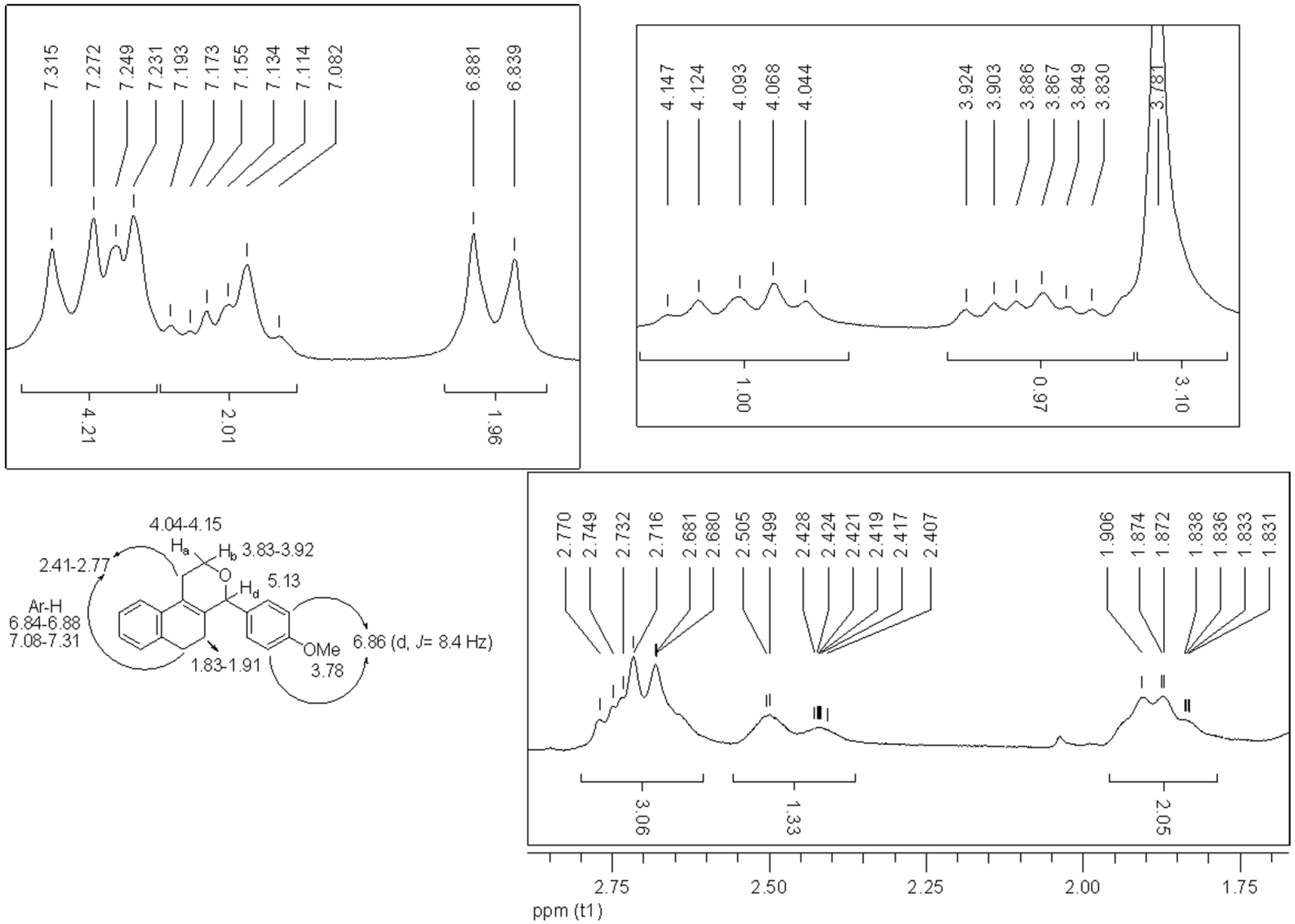

^{13}C-NMR of compound **13d** (50 MHz, $CDCl_3$)

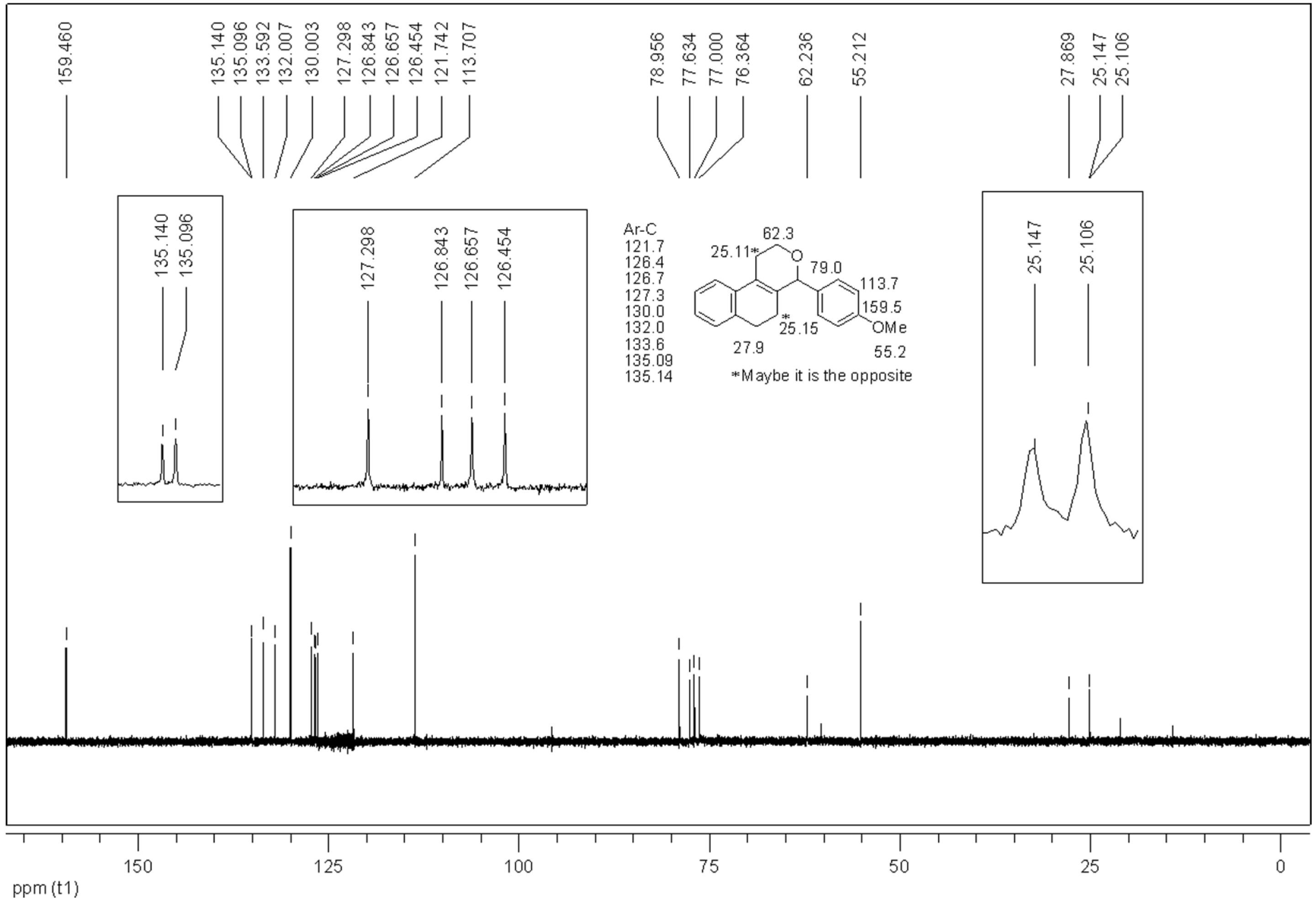

^{1}H-NMR of compound *cis*-**13e** (200 MHz, $CDCl_3$)

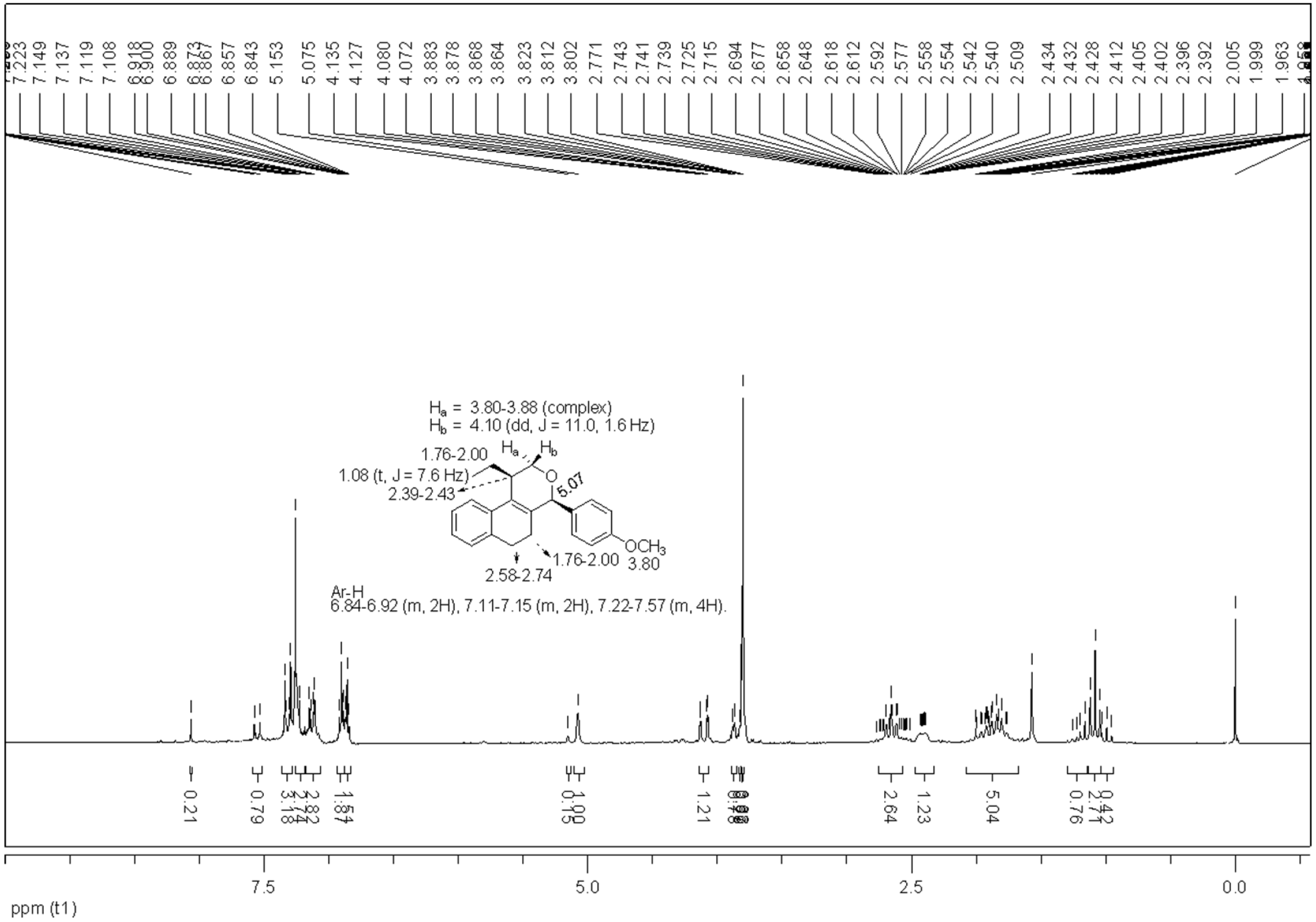

^{1}H-NMR of compound *cis*-**13e** (200 MHz, $CDCl_3$)

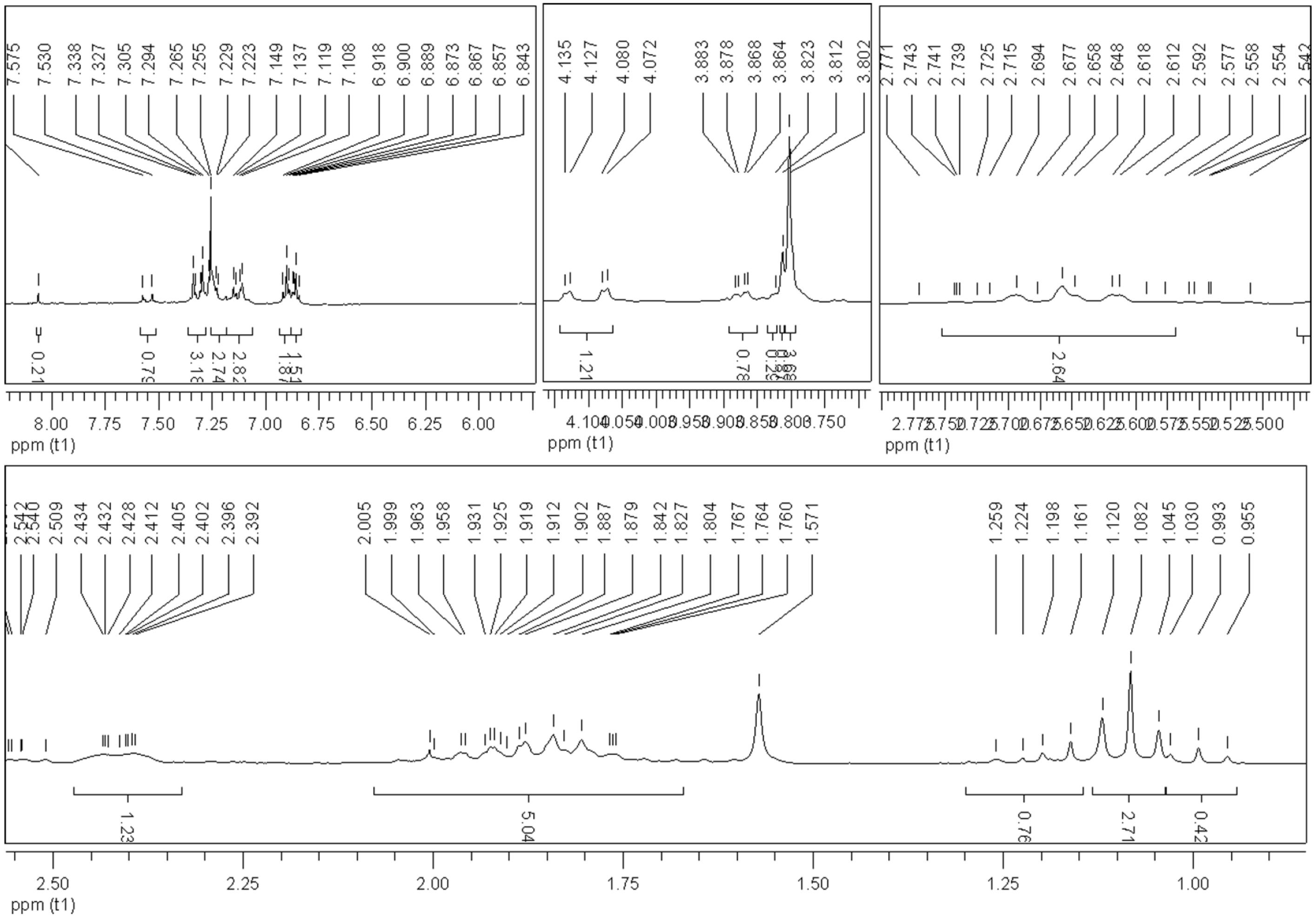

^{13}C-NMR of compound *cis*-**13e** (50 MHz, $CDCl_3$)

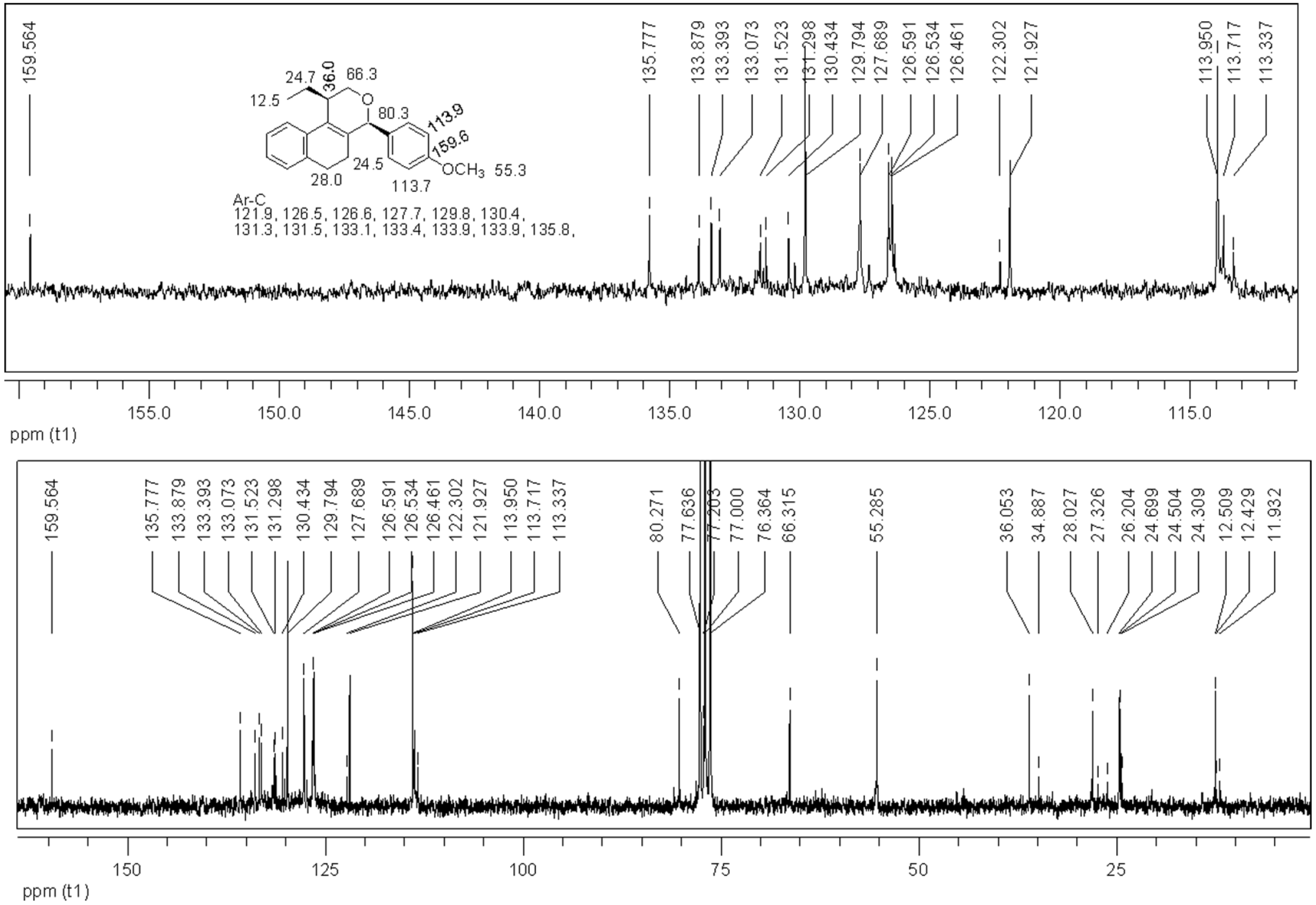

^{1}H-NMR of compound *trans*-**13e** (200 MHz, $CDCl_3$)

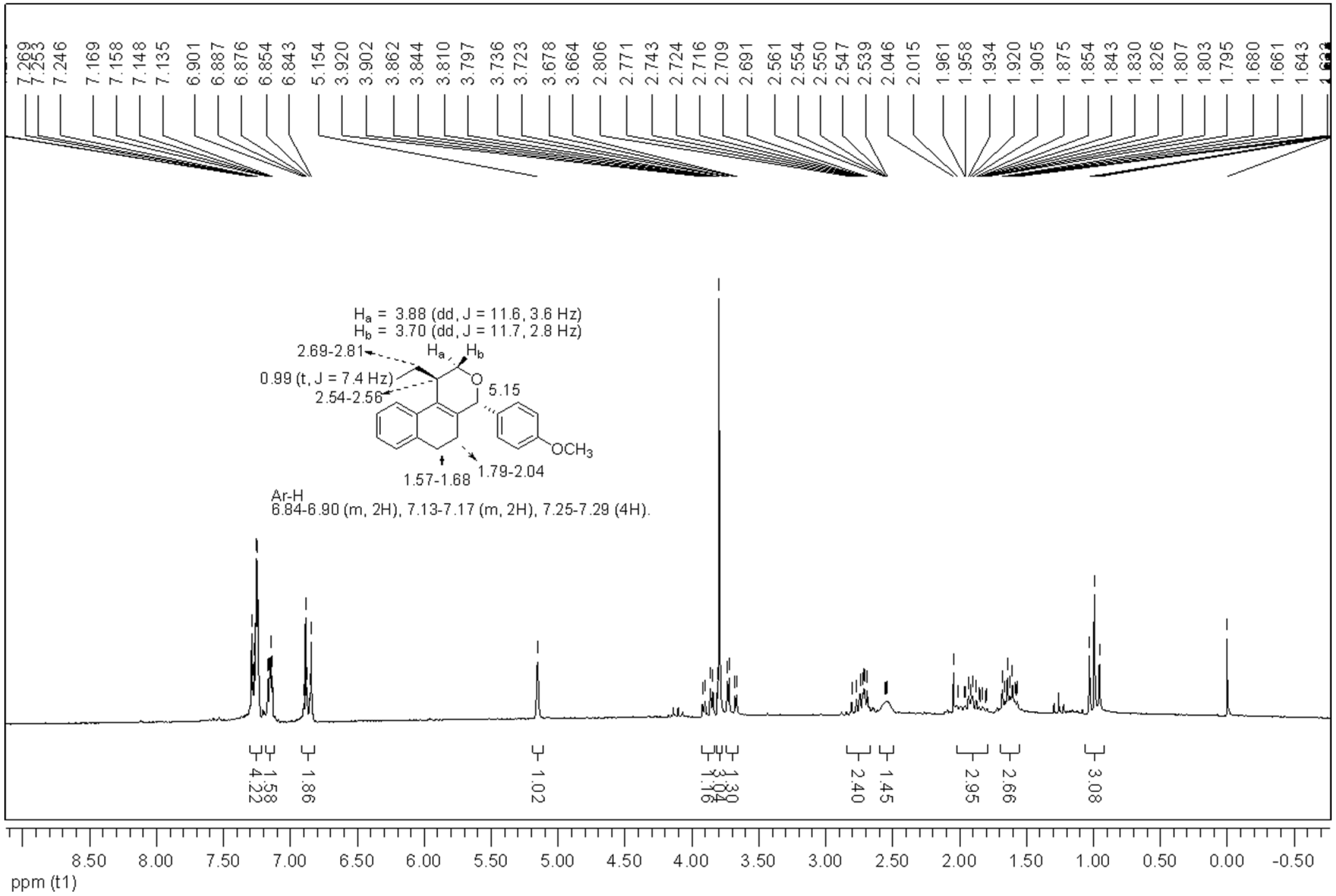

^{1}H-NMR of compound *trans*-**13e** (200 MHz, $CDCl_3$)

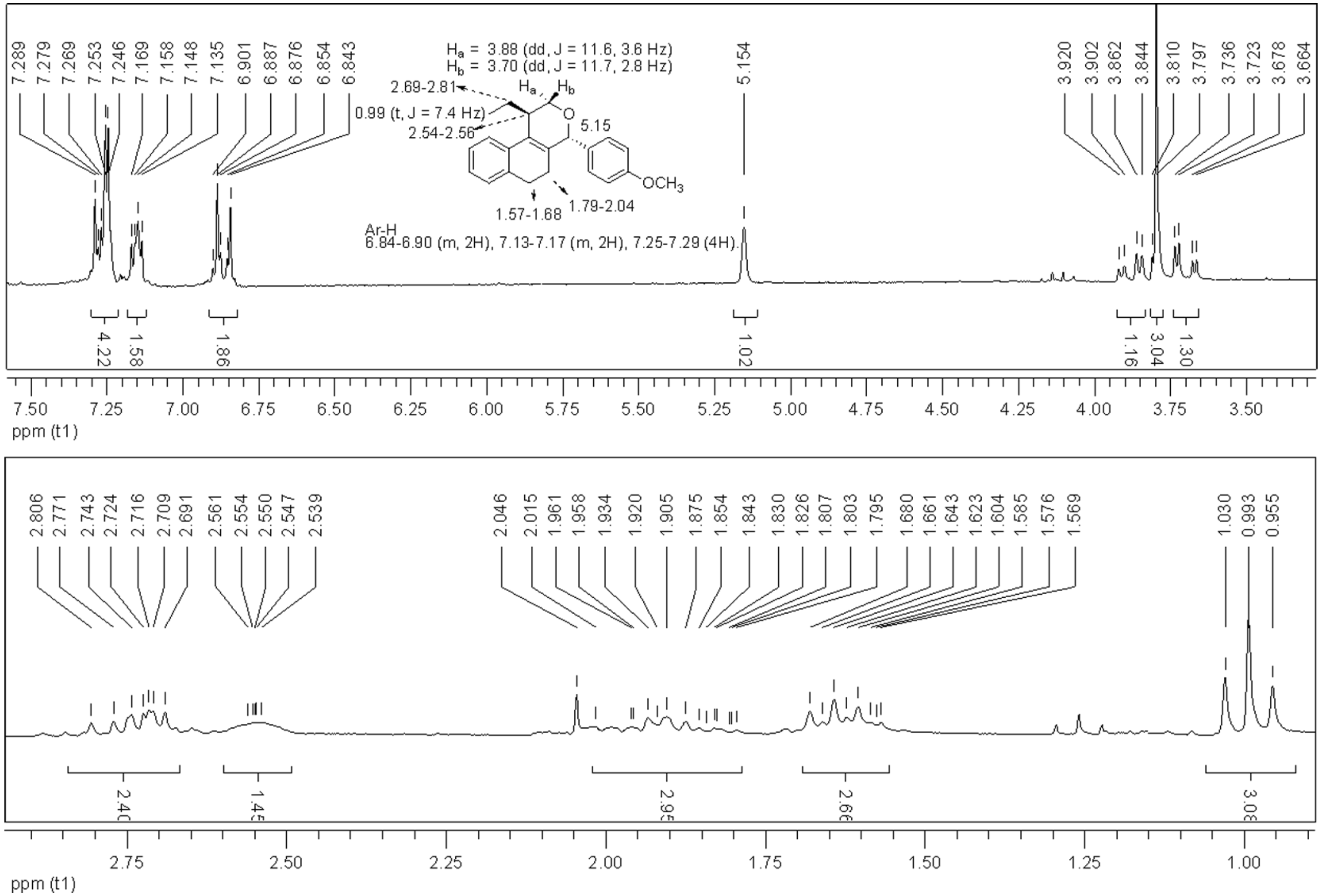

^{13}C-NMR of compound *trans*-**13e** (50 MHz, $CDCl_3$)

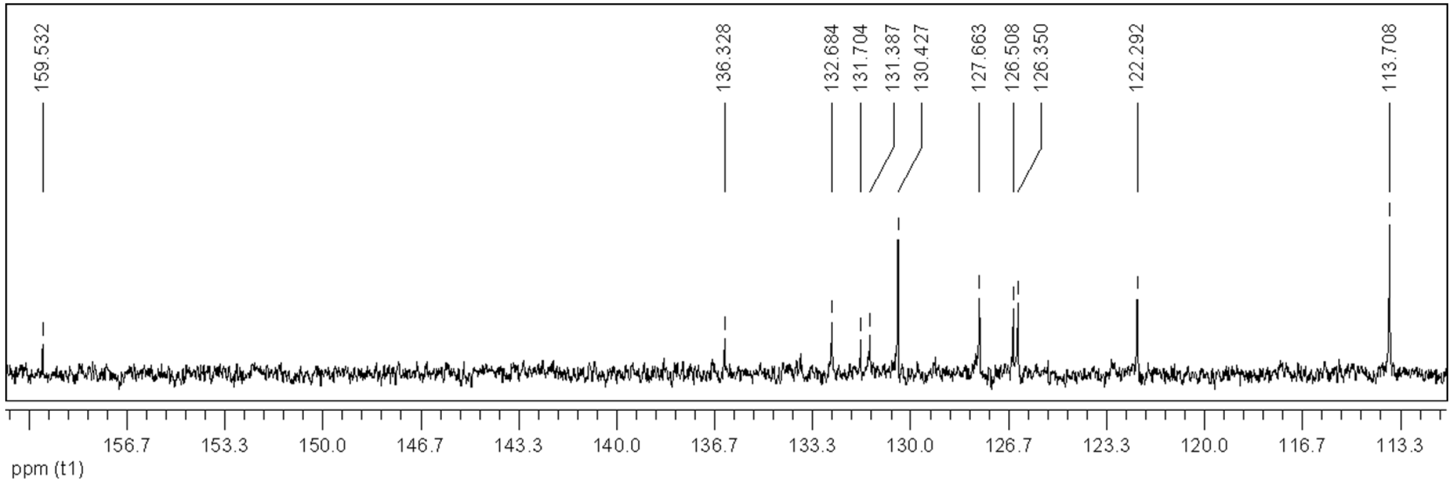

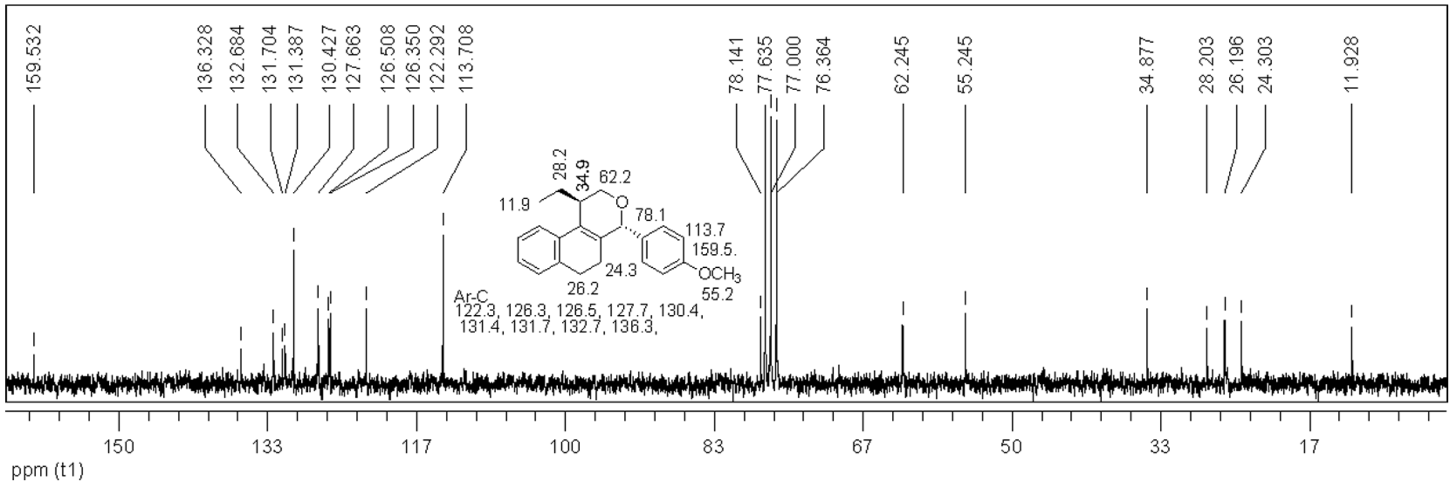

2D NOESY NMR of compound *trans*-**13e** (300 MHz, $CDCl_3$)

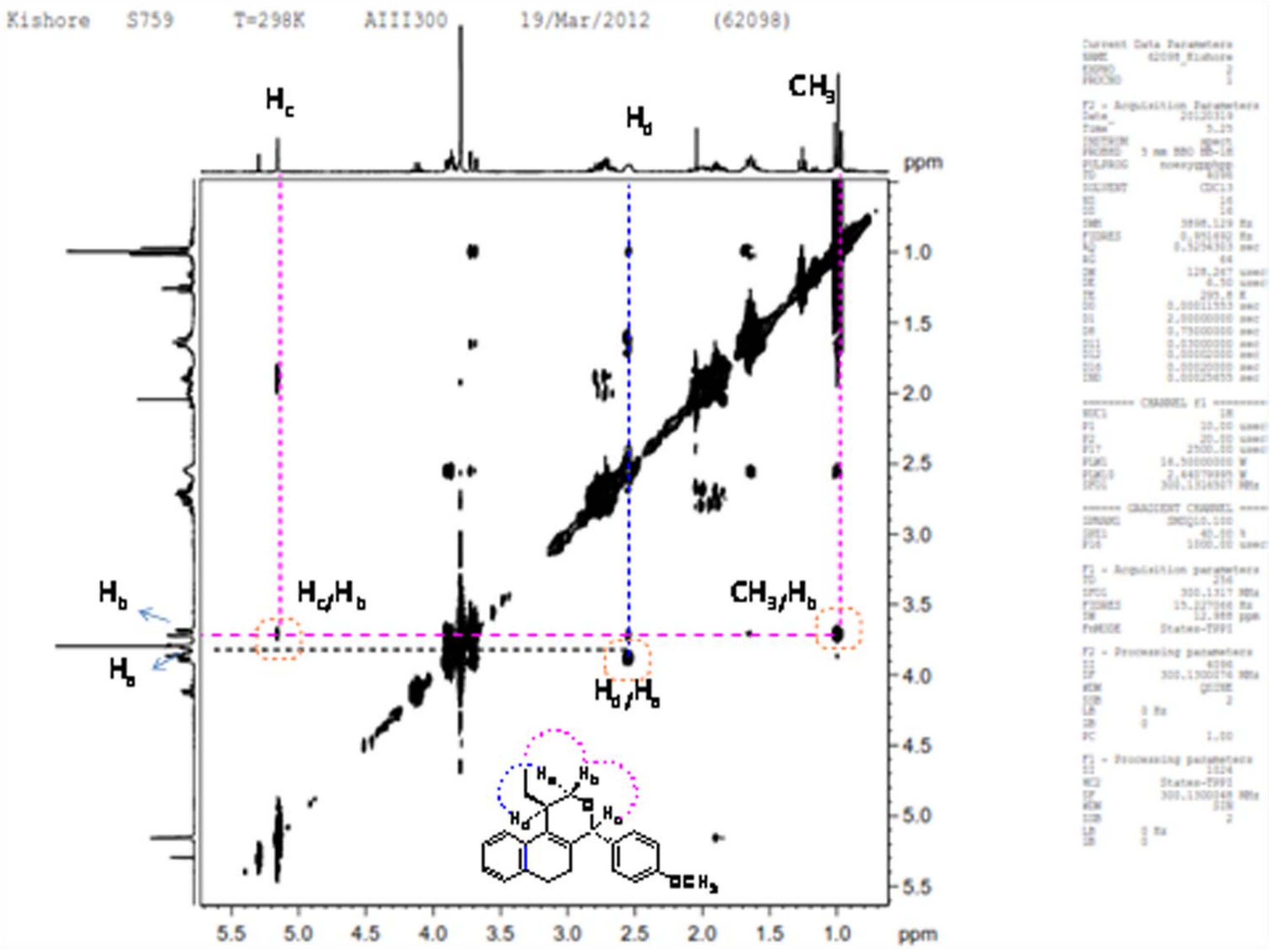

^{1}H-NMR of compound *cis*-**15e** (200 MHz, $CDCl_3$)

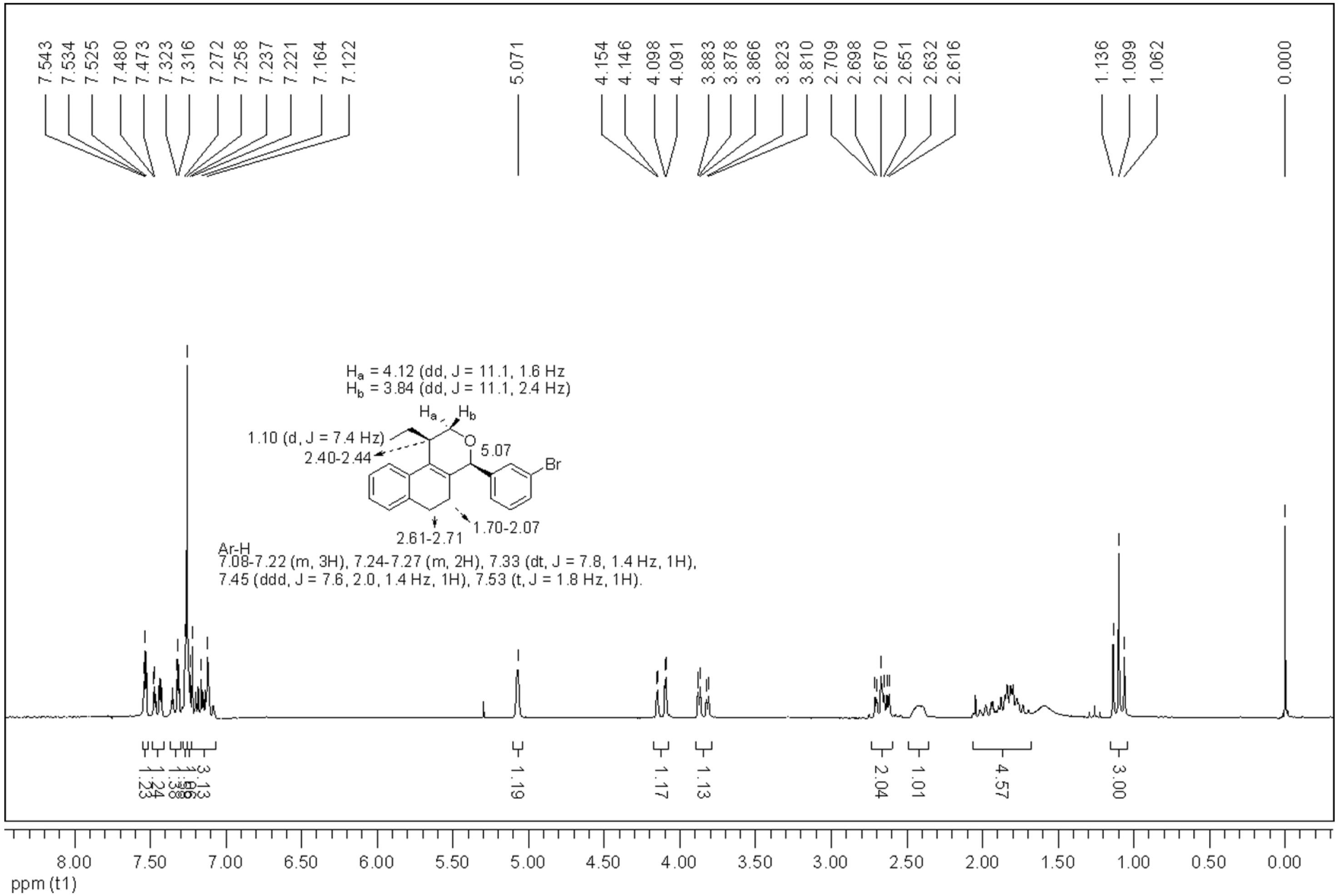

^{1}H-NMR of compound *cis*-**15e** (200 MHz, $CDCl_3$)

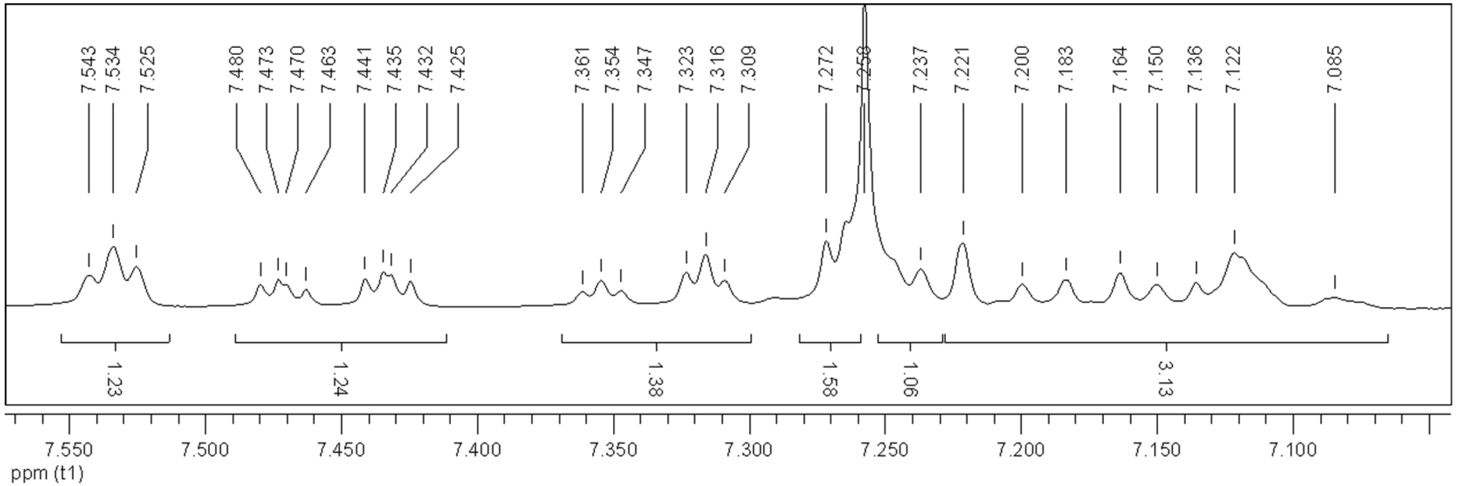

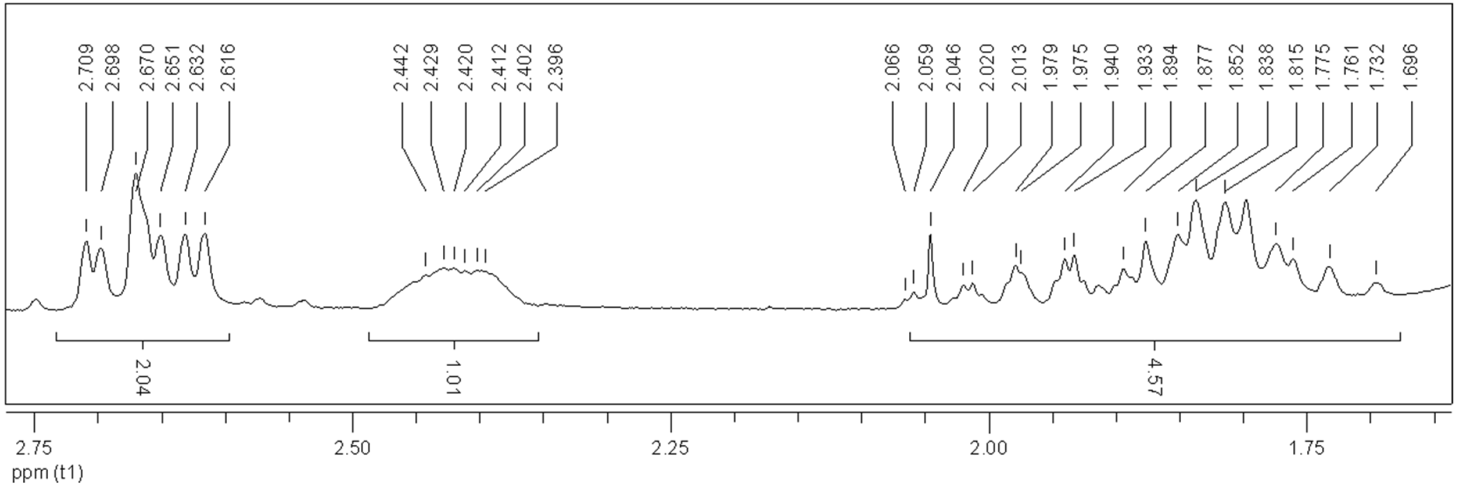

^{13}C-NMR of compound *cis*-15e (50 MHz, $CDCl_3$)

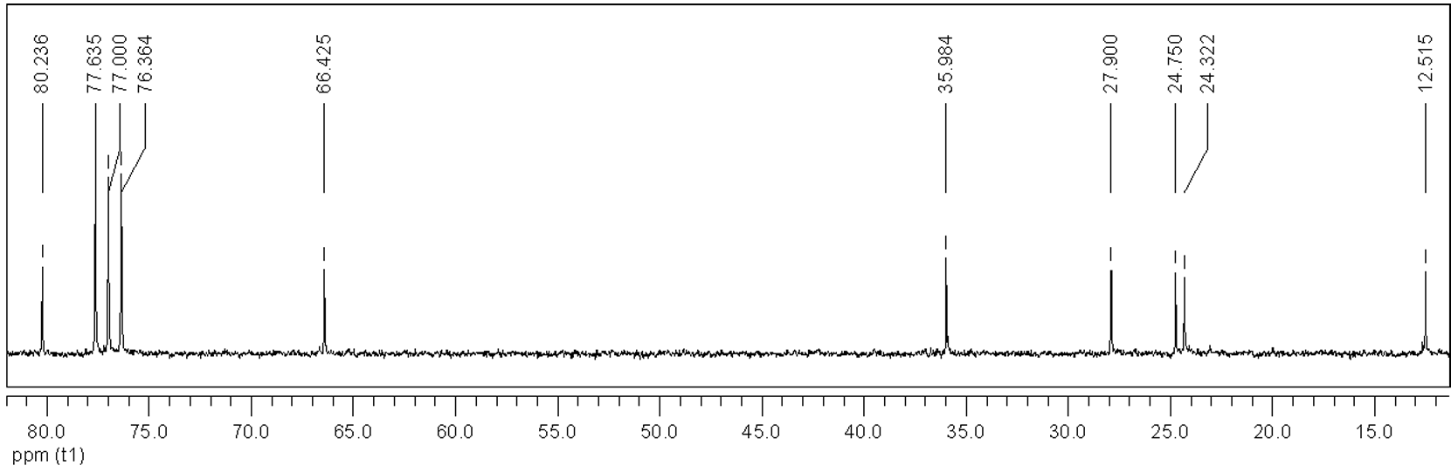

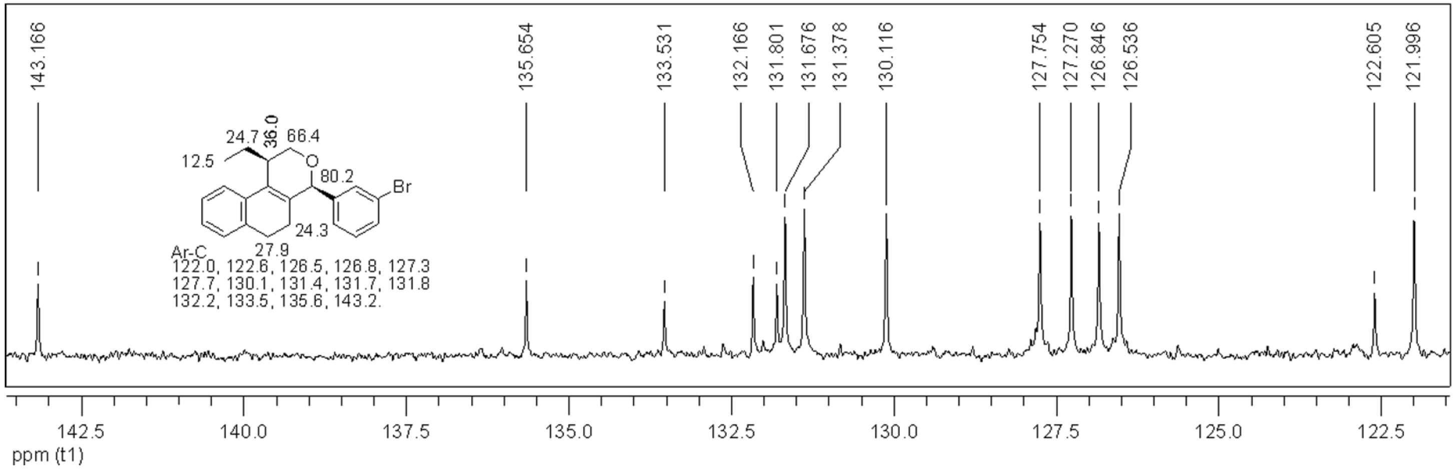

2D NOESY NMR of compound *cis*-**15e** (500 MHz, $CDCl_3$)

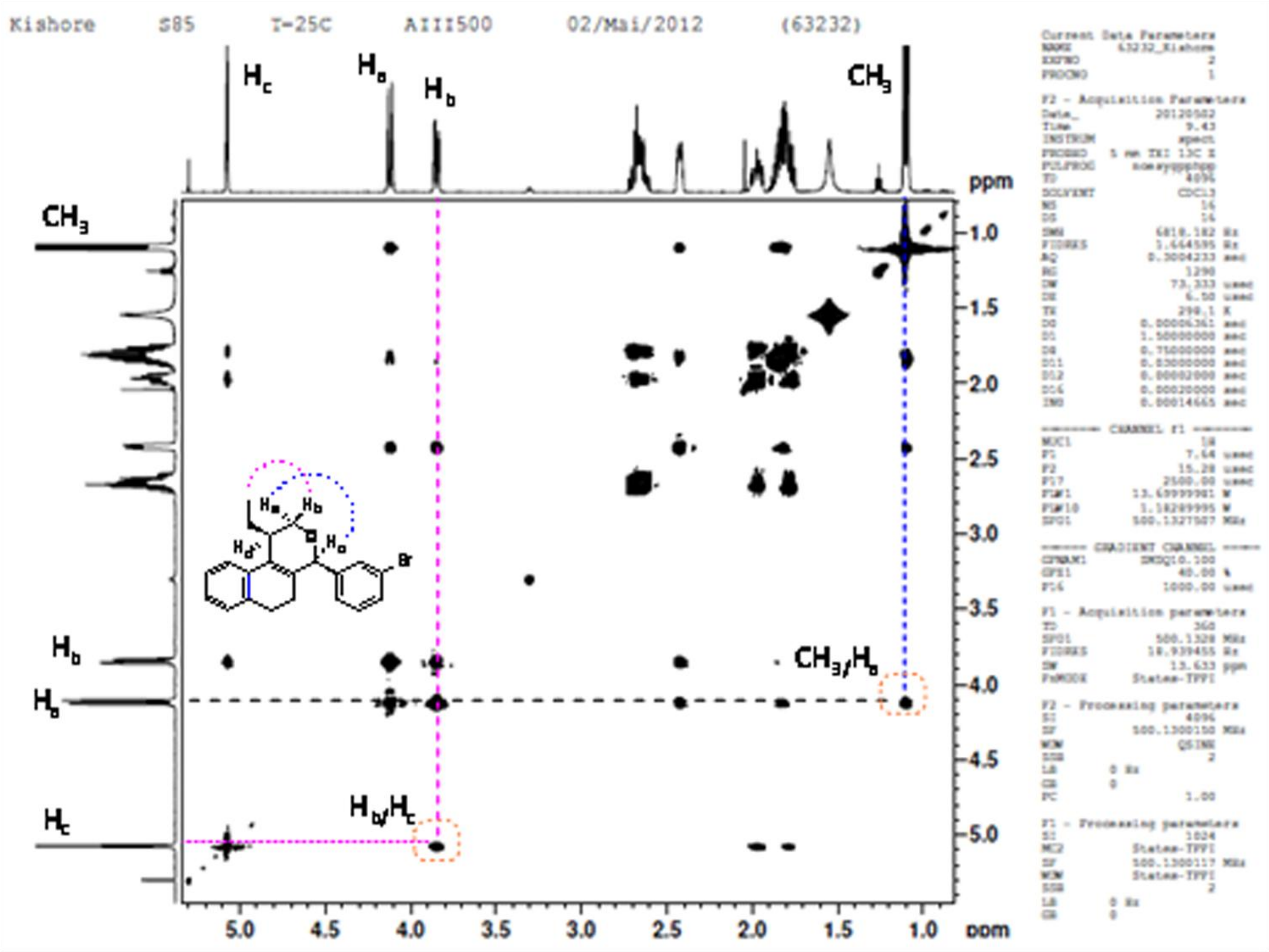

^{1}H-NMR of compounds *cis*-**13g** and *trans*-**13g** (500 MHz, $CDCl_3$)

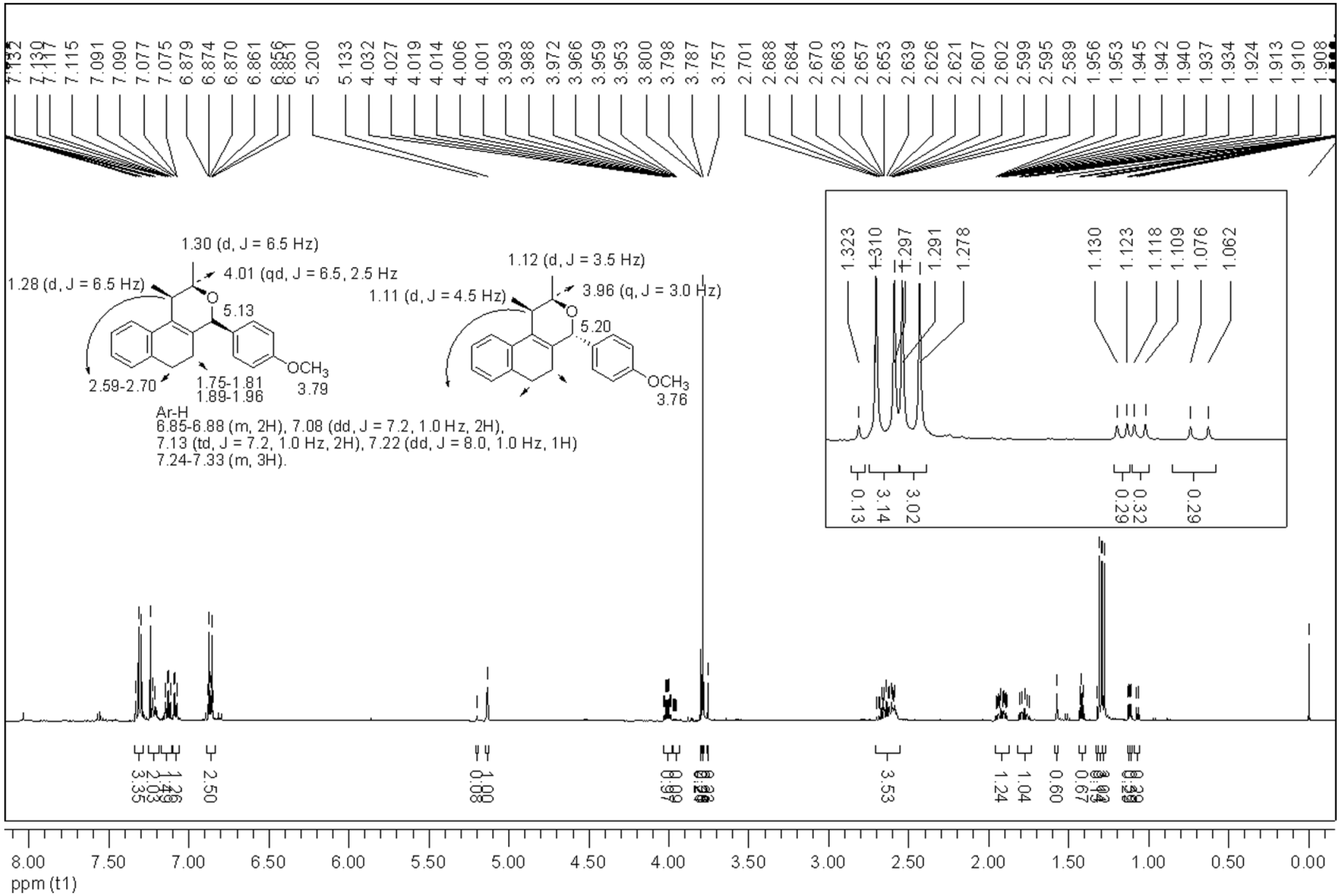

^{1}H-NMR of compounds *cis*-**13g** and *trans*-**13g** (300 MHz, $CDCl_3$)

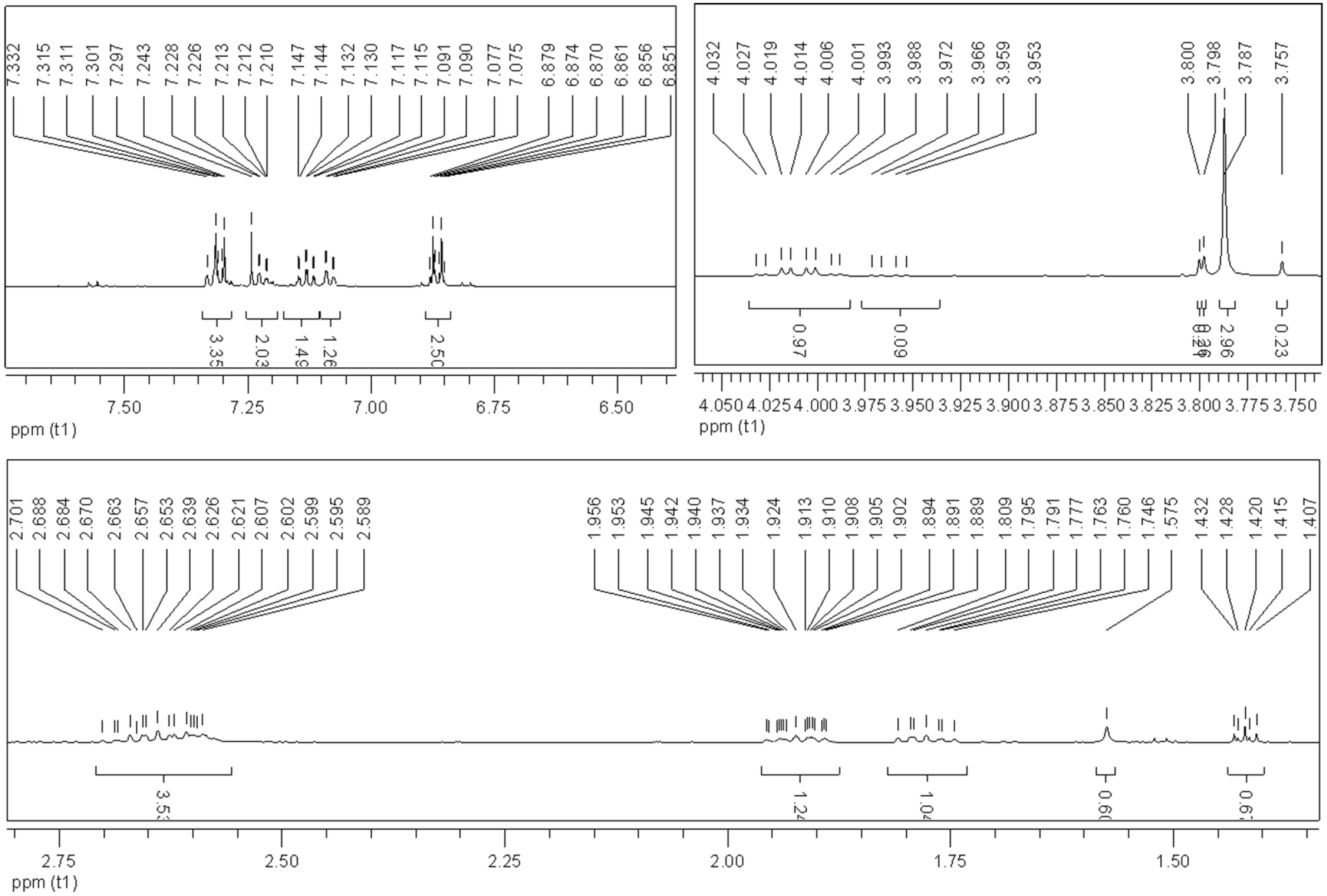

^{13}C-NMR of compounds *cis*-**13g** and *trans*-**13g** (50 MHz, $CDCl_3$)

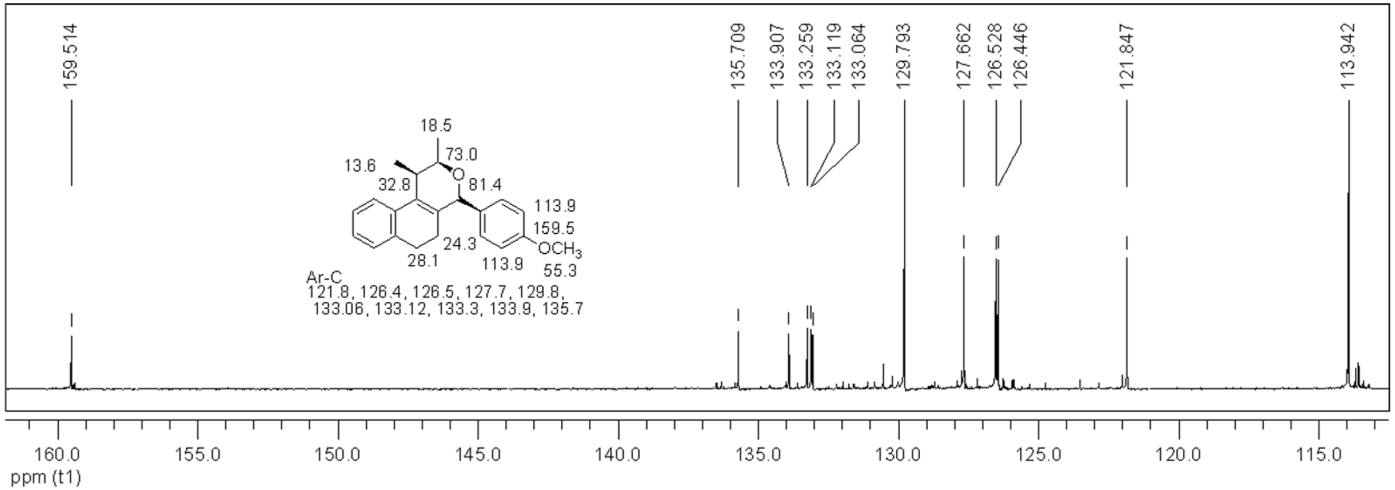

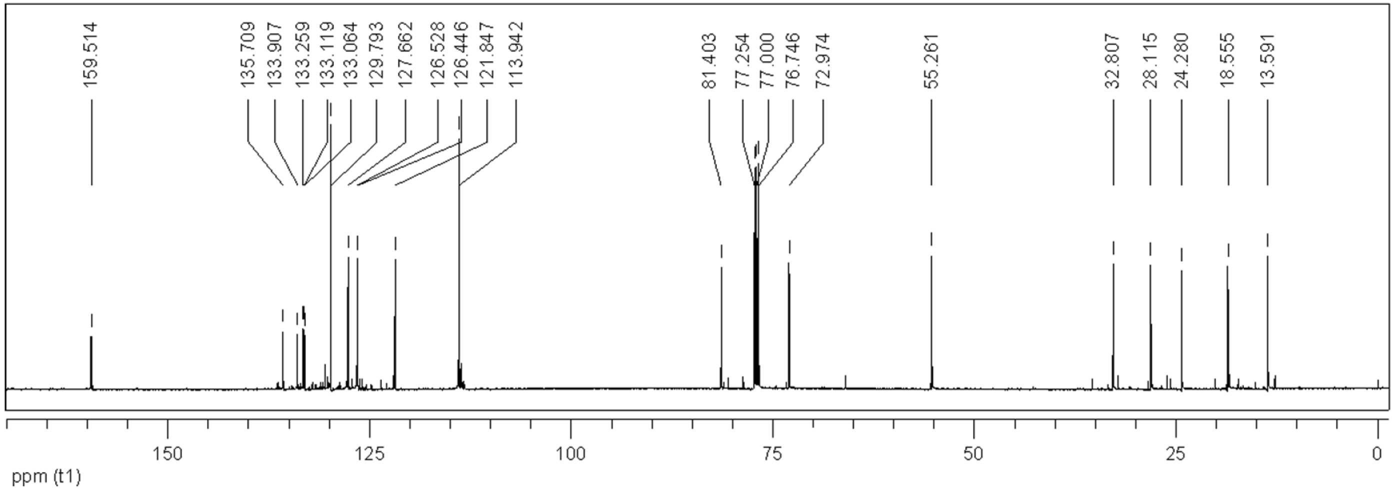

2D NOESY NMR of compound *cis*-**13g** (500 MHz, $CDCl_3$)

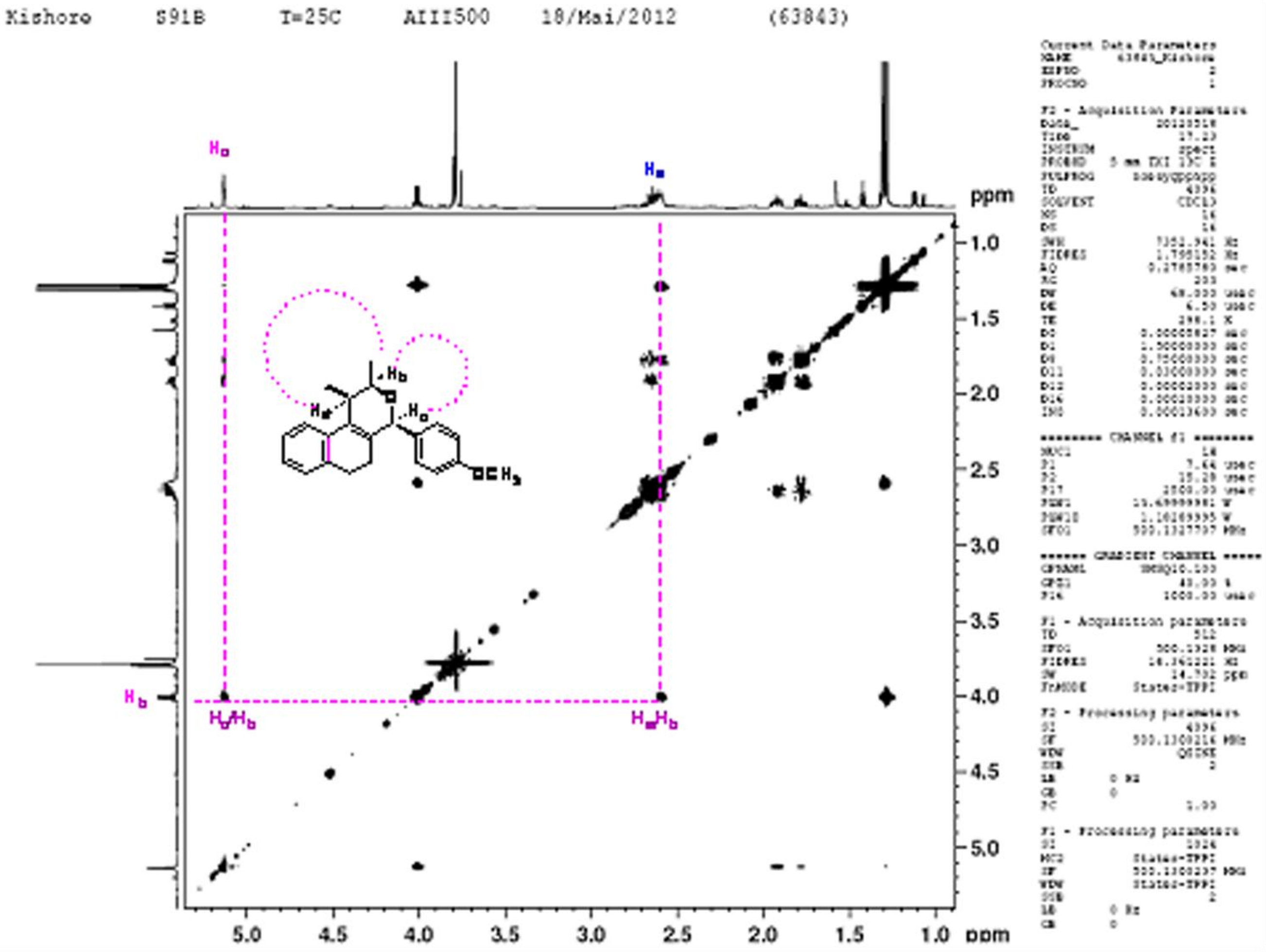

^{1}H-NMR of compound **5h** (500 MHz, $CDCl_3$)

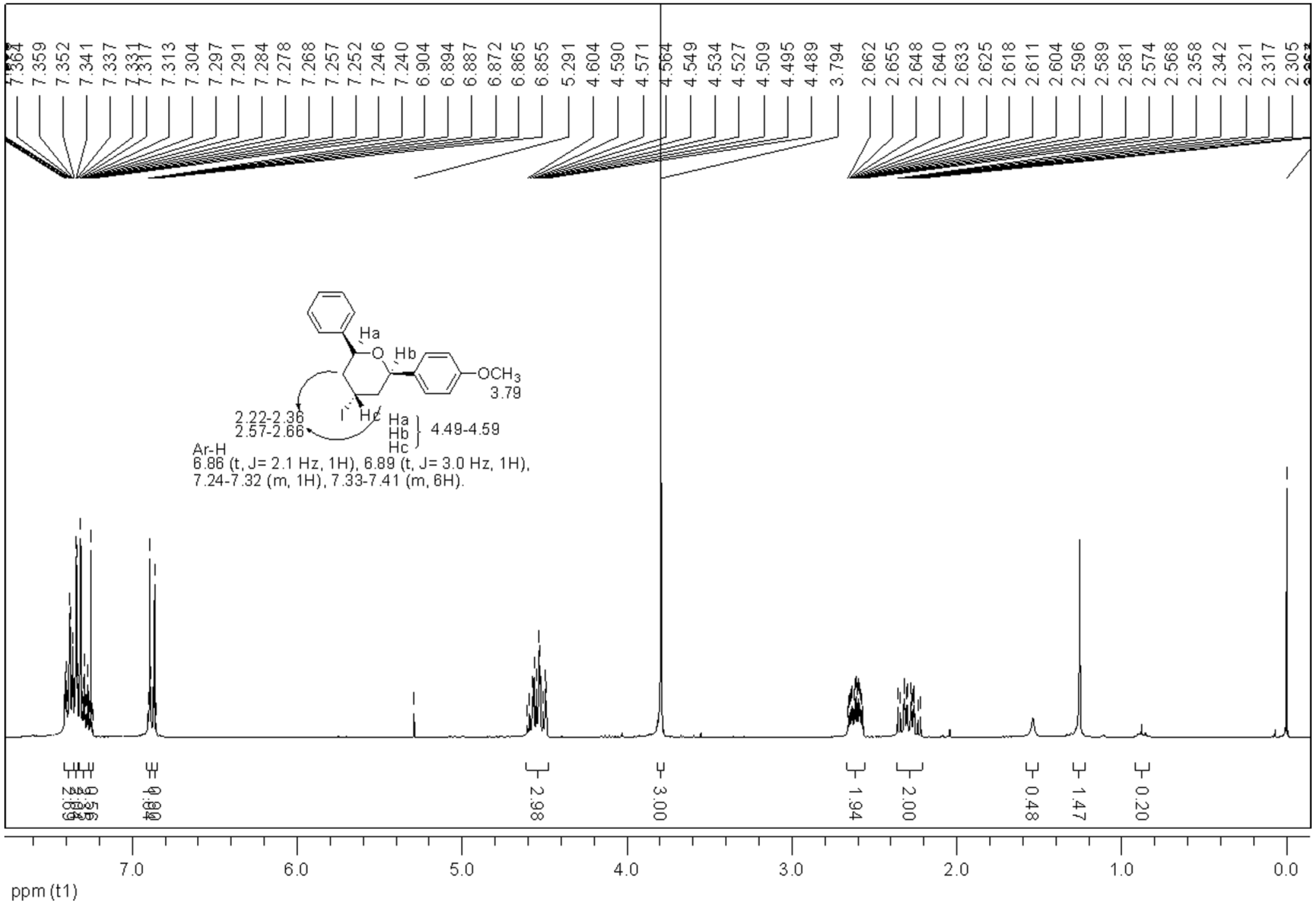

^{1}H-NMR of compound **5h** (500 MHz, $CDCl_3$)

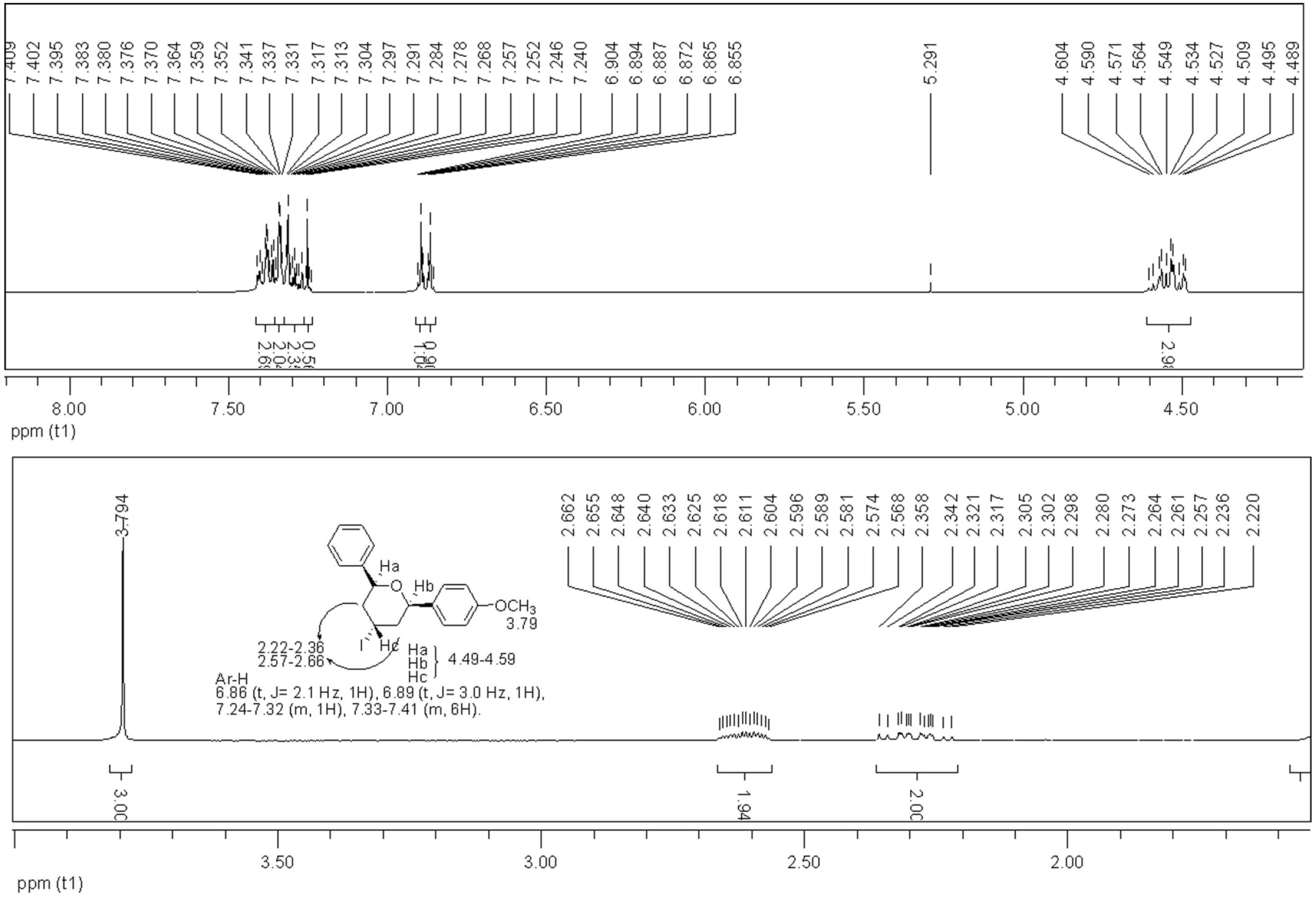

^{13}C-NMR of compound **5h** (75 MHz, $CDCl_3$)

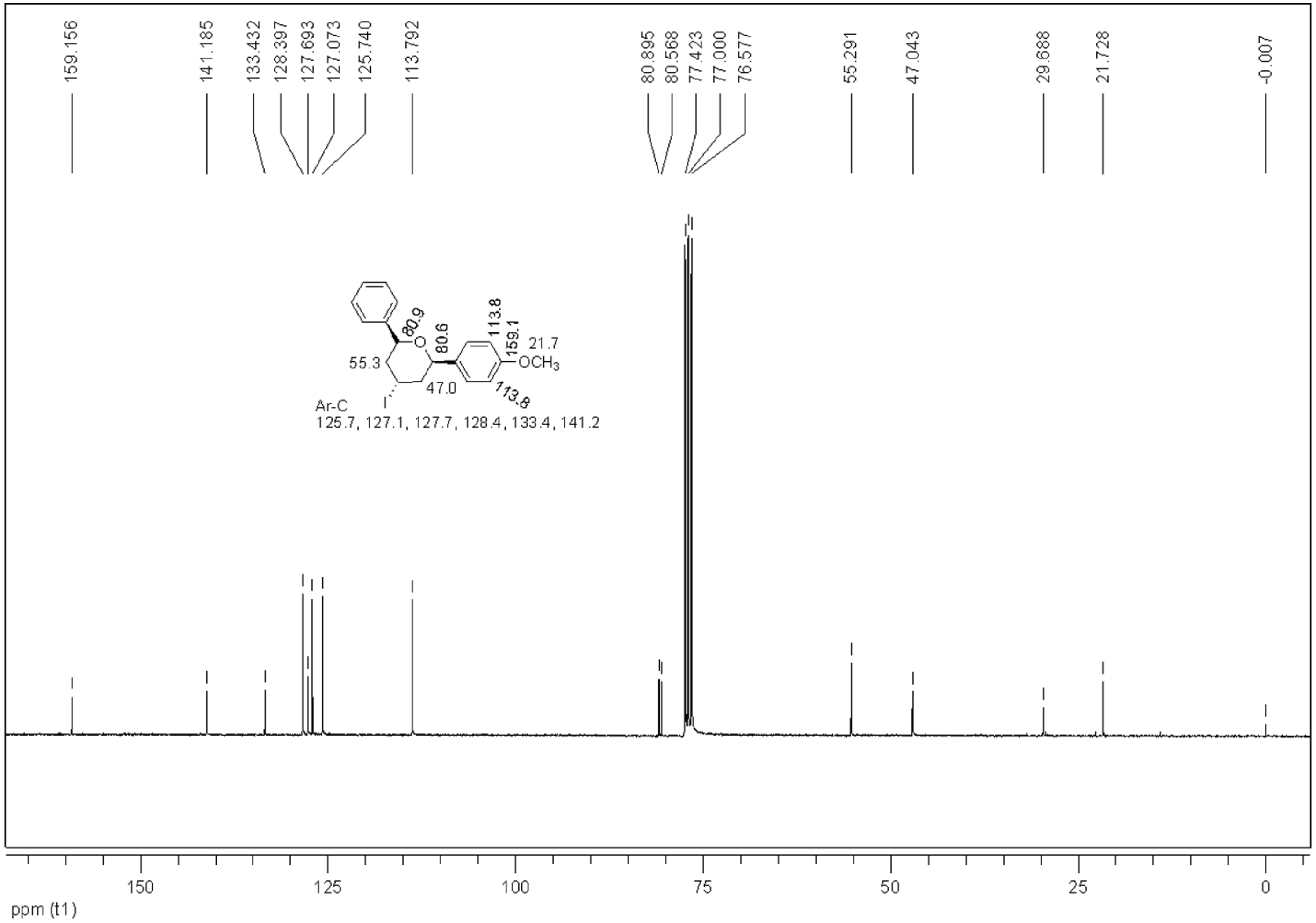

^{1}H-NMR of compound **5i** (300 MHz, $CDCl_3$)

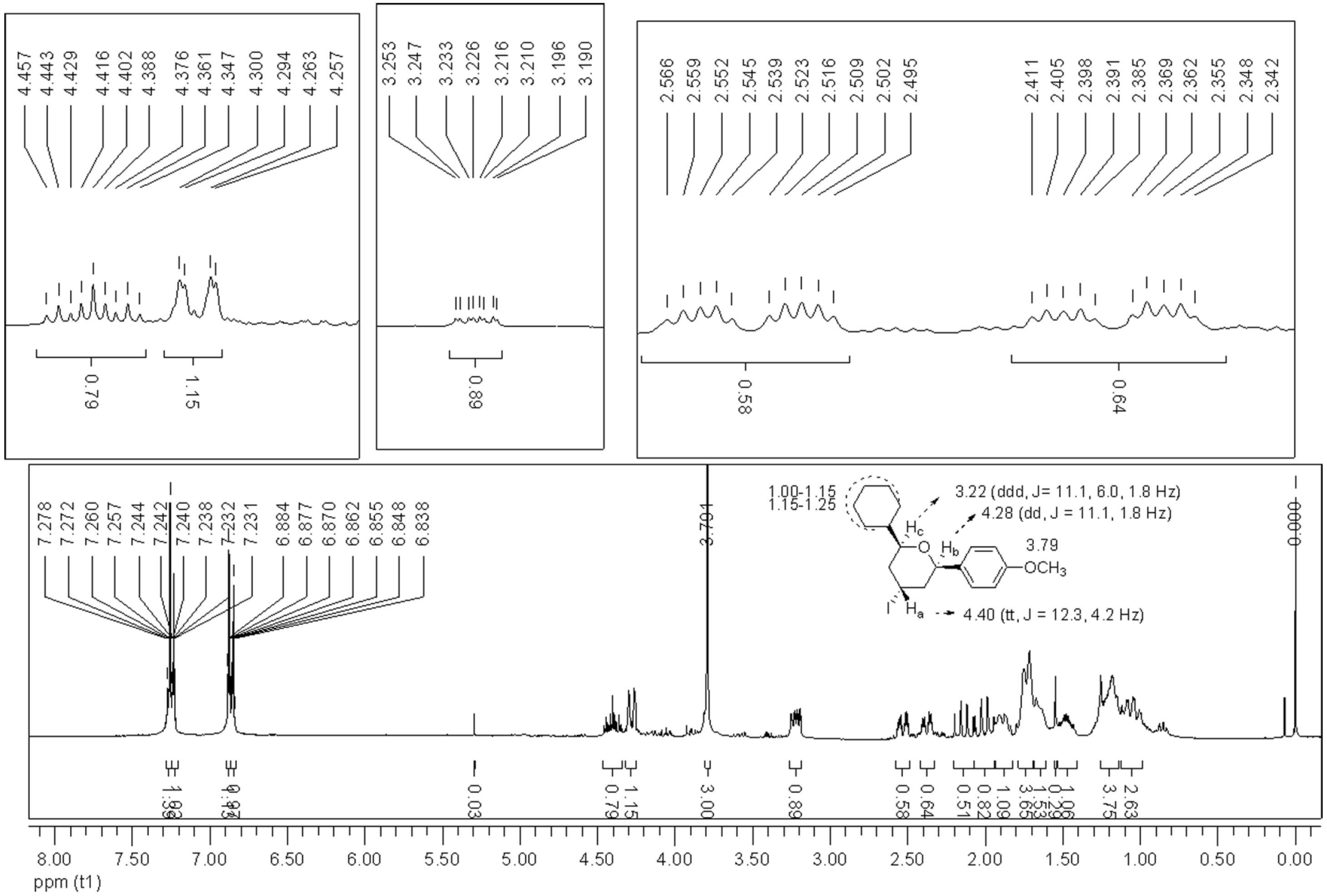

^{1}H-NMR of compound **5i** (300 MHz, $CDCl_3$)

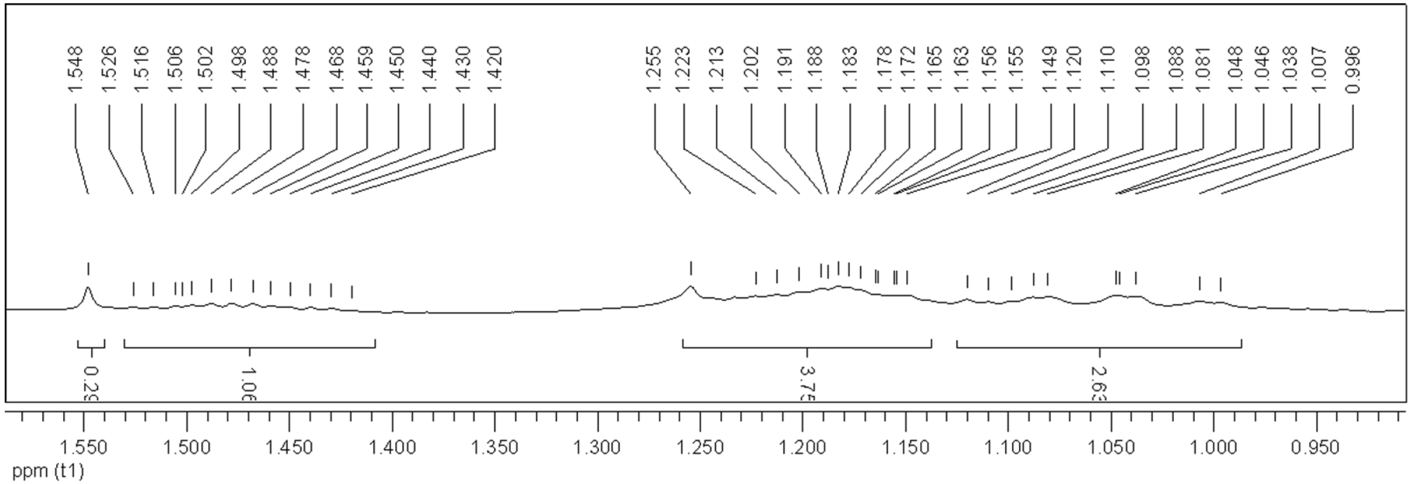

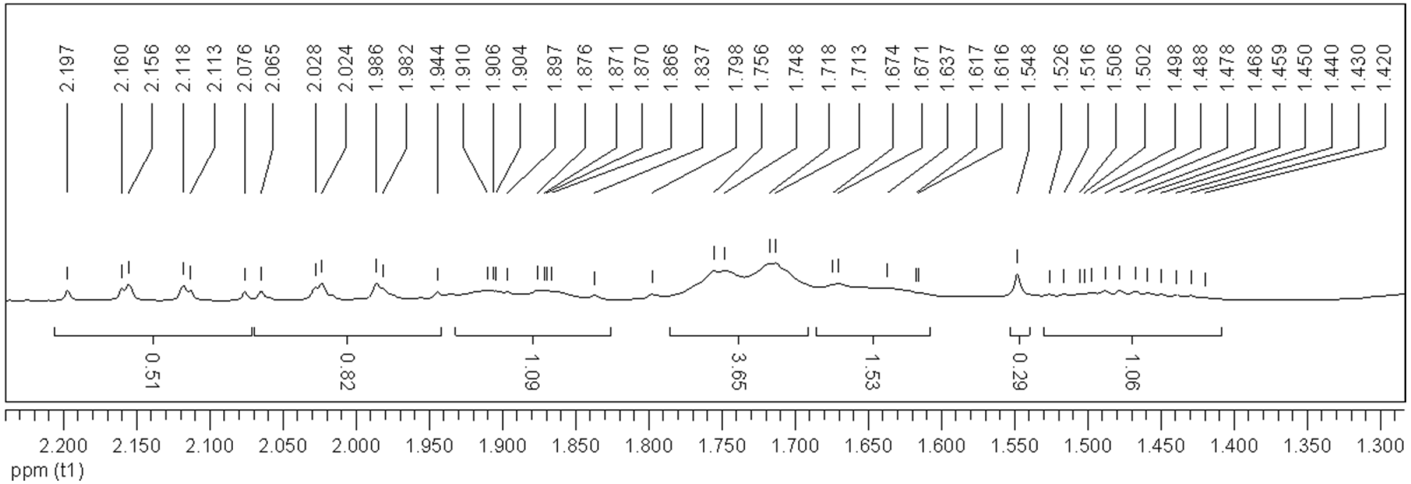

^{13}C-NMR of compound **5i** (75 MHz, $CDCl_3$)

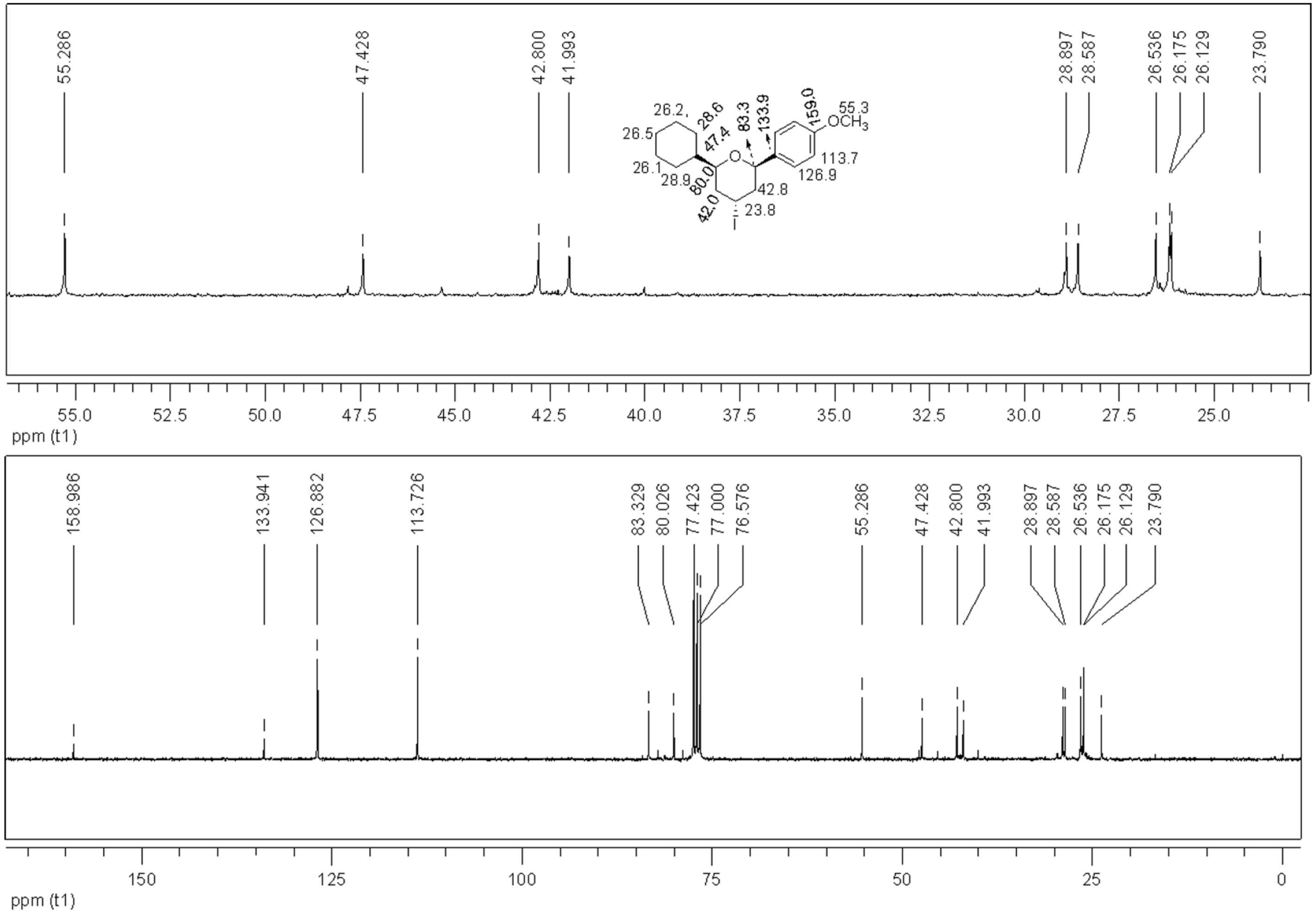

^{1}H-NMR of compound **5j** (500 MHz, $CDCl_3$)

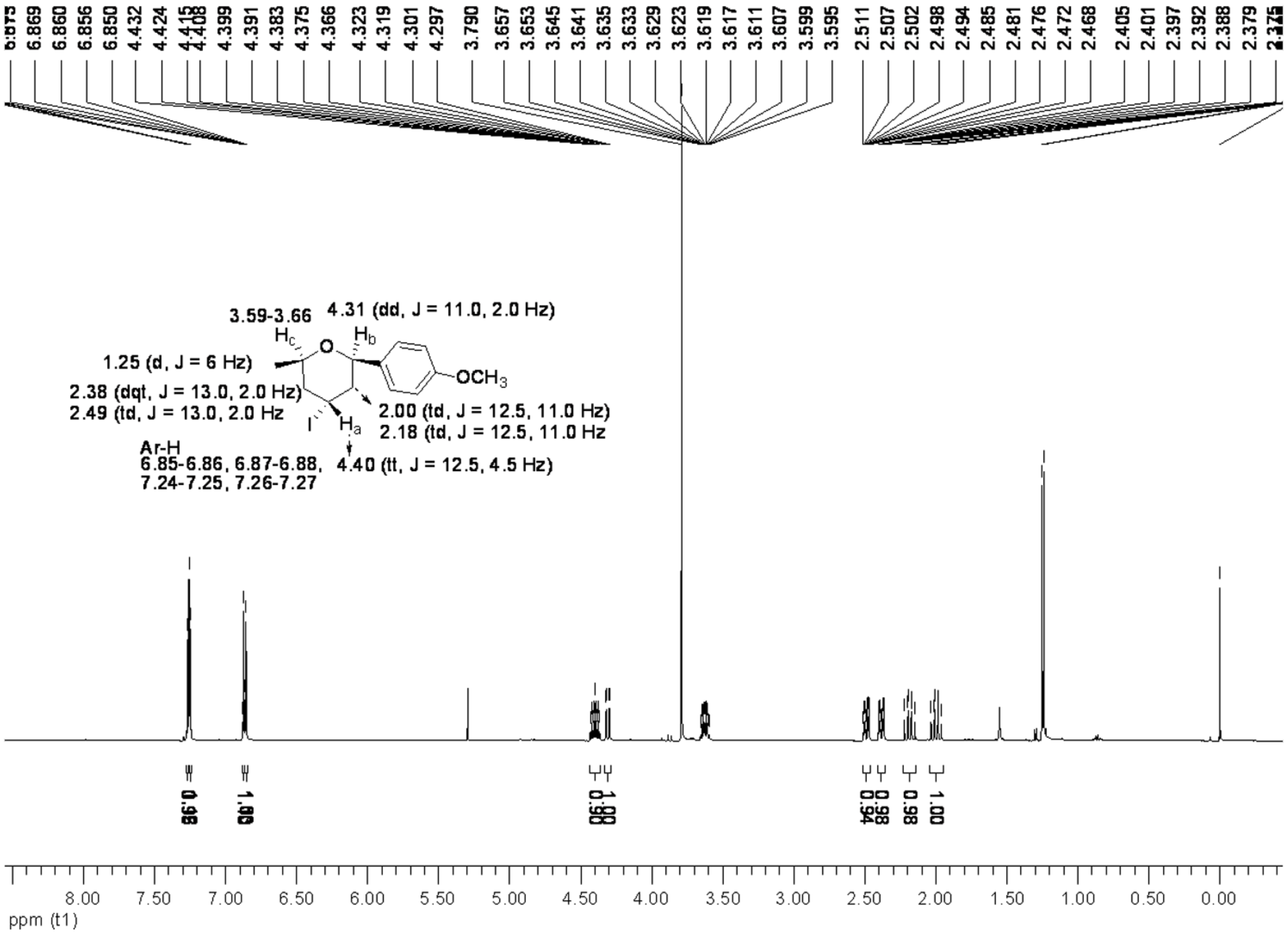

^{13}C-NMR of compound **5j** (50 MHz, $CDCl_3$)

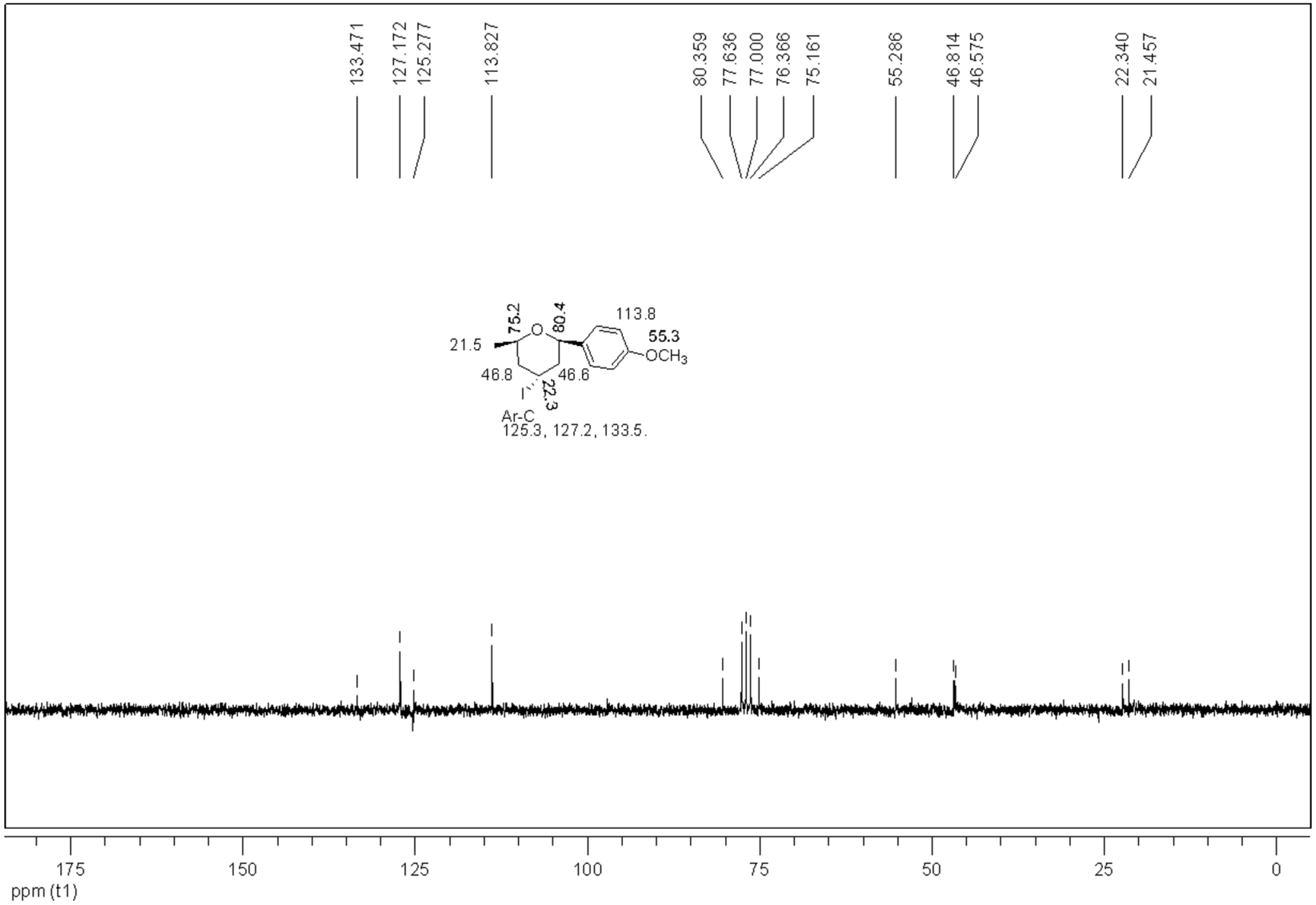

2D NOESY NMR of compound **5j** (500 MHz, $CDCl_3$)

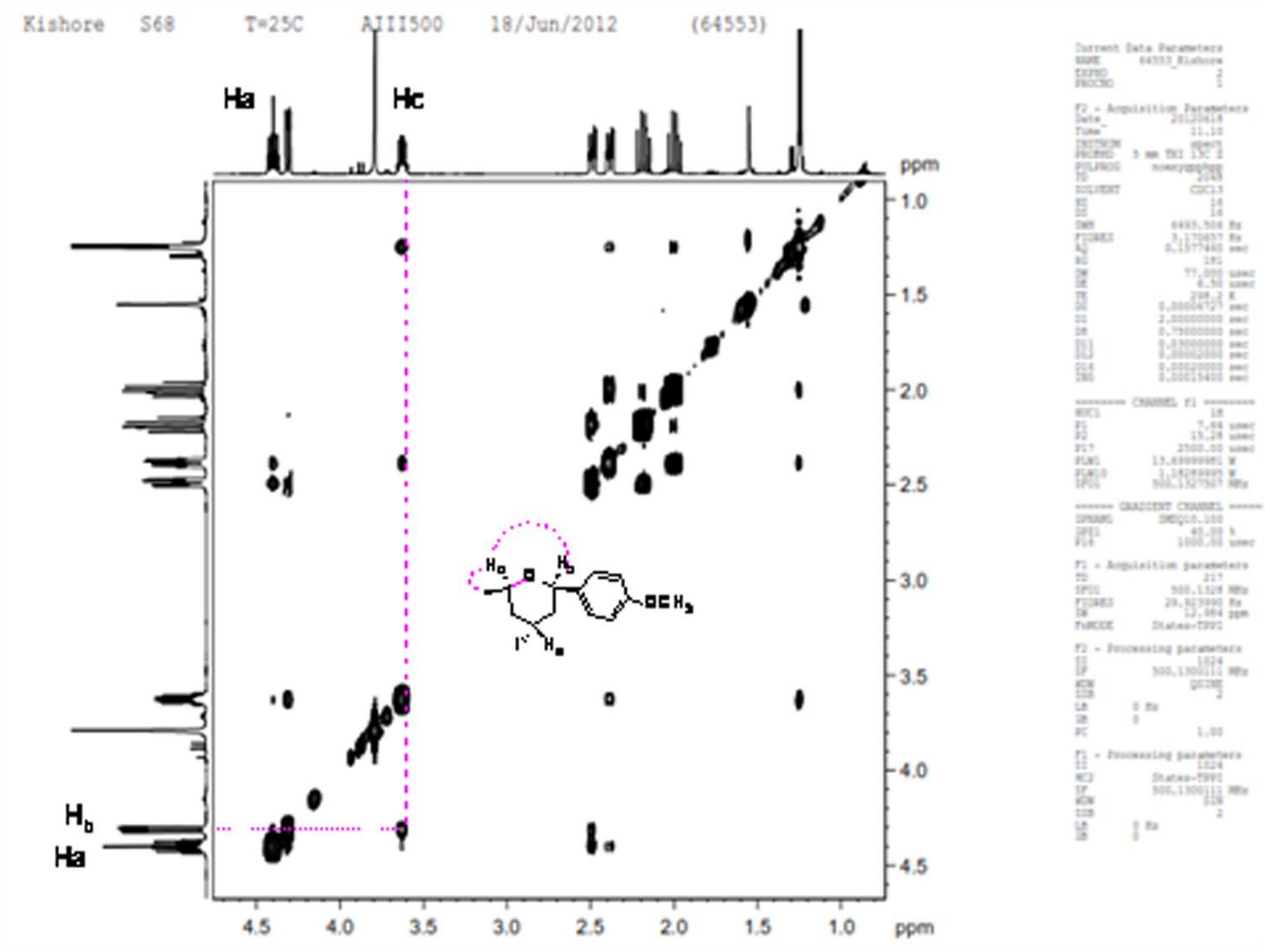

^{1}H-NMR of compound **5k** (300 MHz, $CDCl_3$)

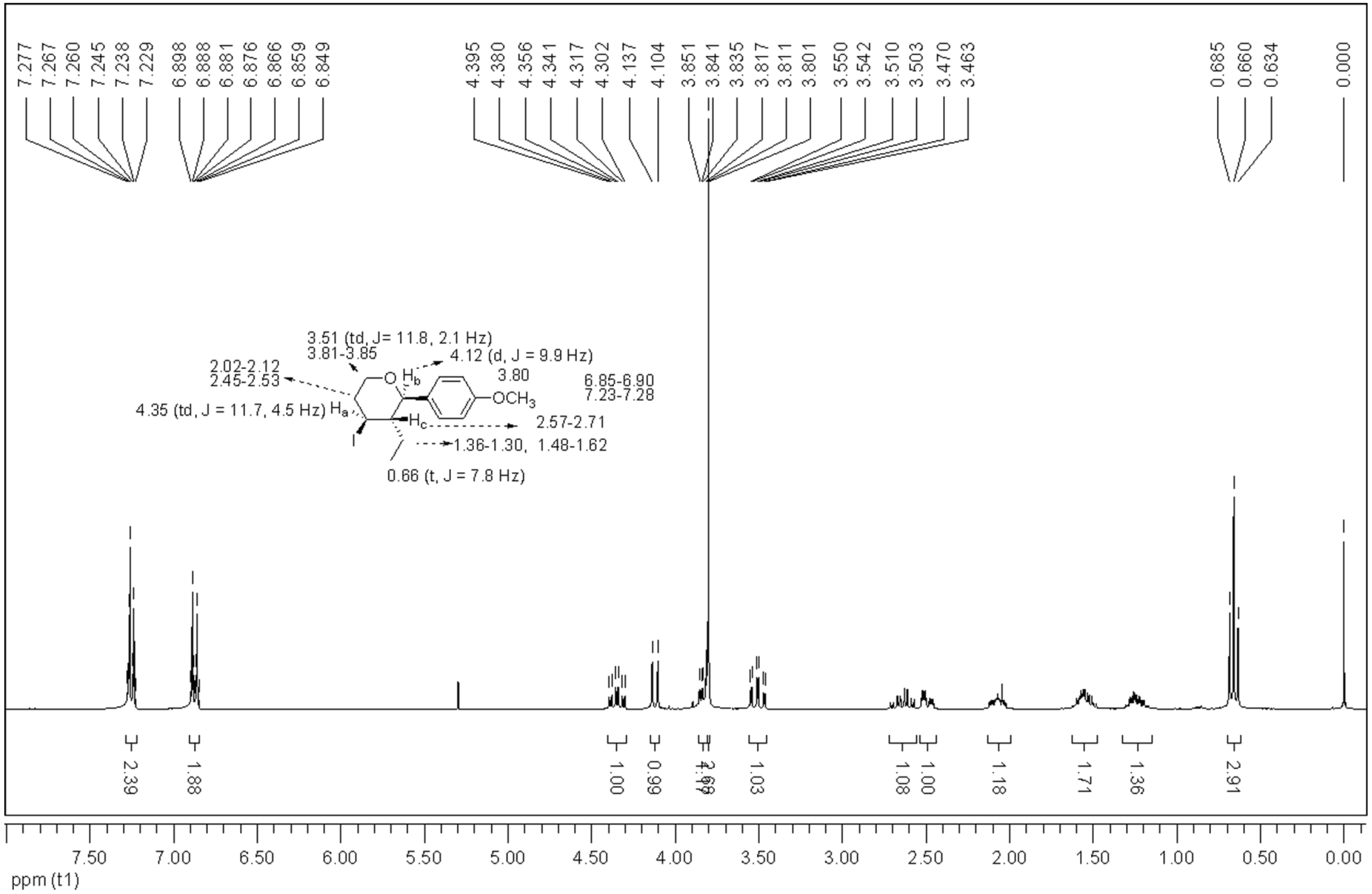

^{1}H-NMR of compound **5k** (300 MHz, $CDCl_3$)

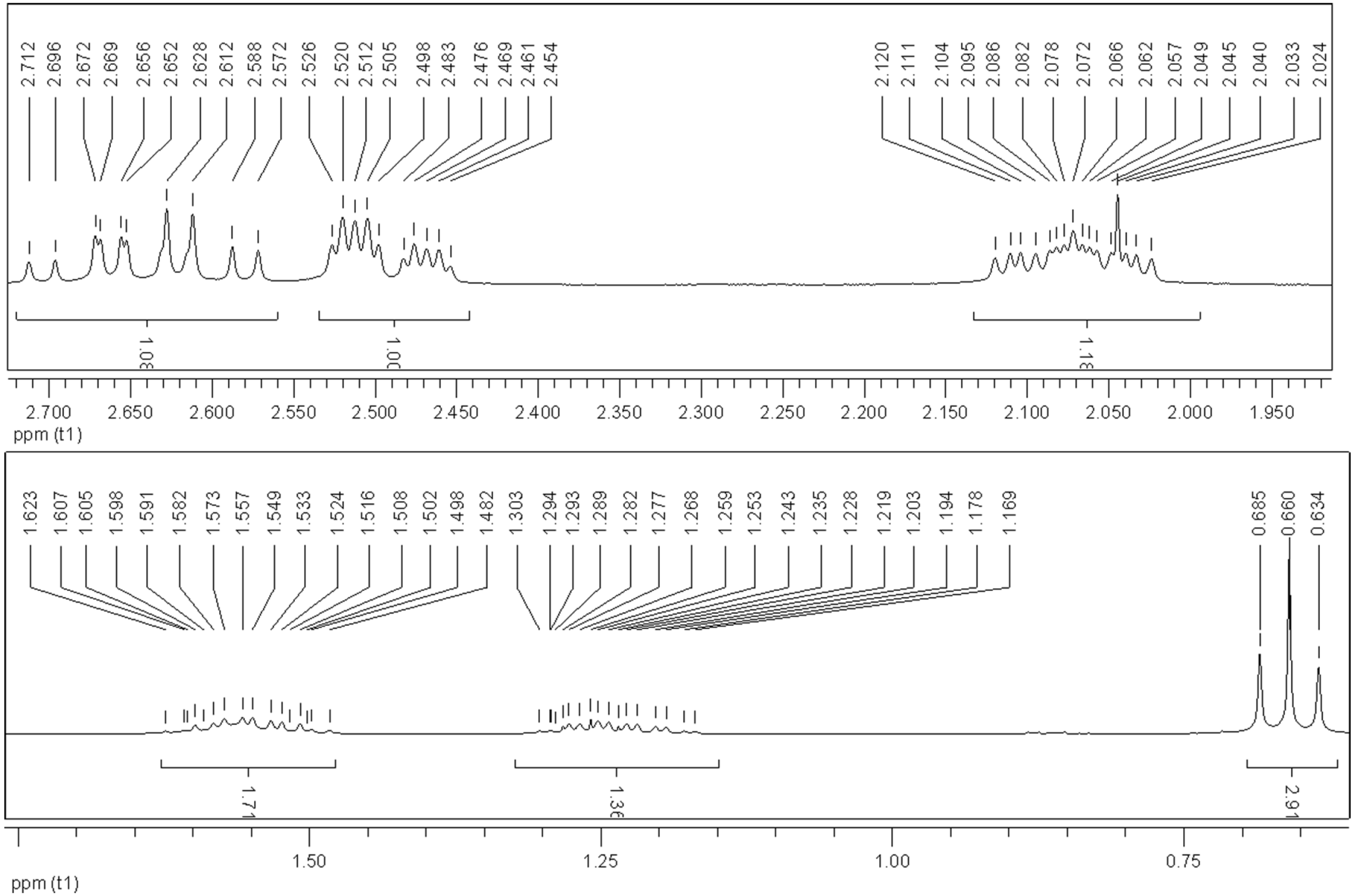

^{13}C-NMR of compound **5k** (75 MHz, $CDCl_3$)

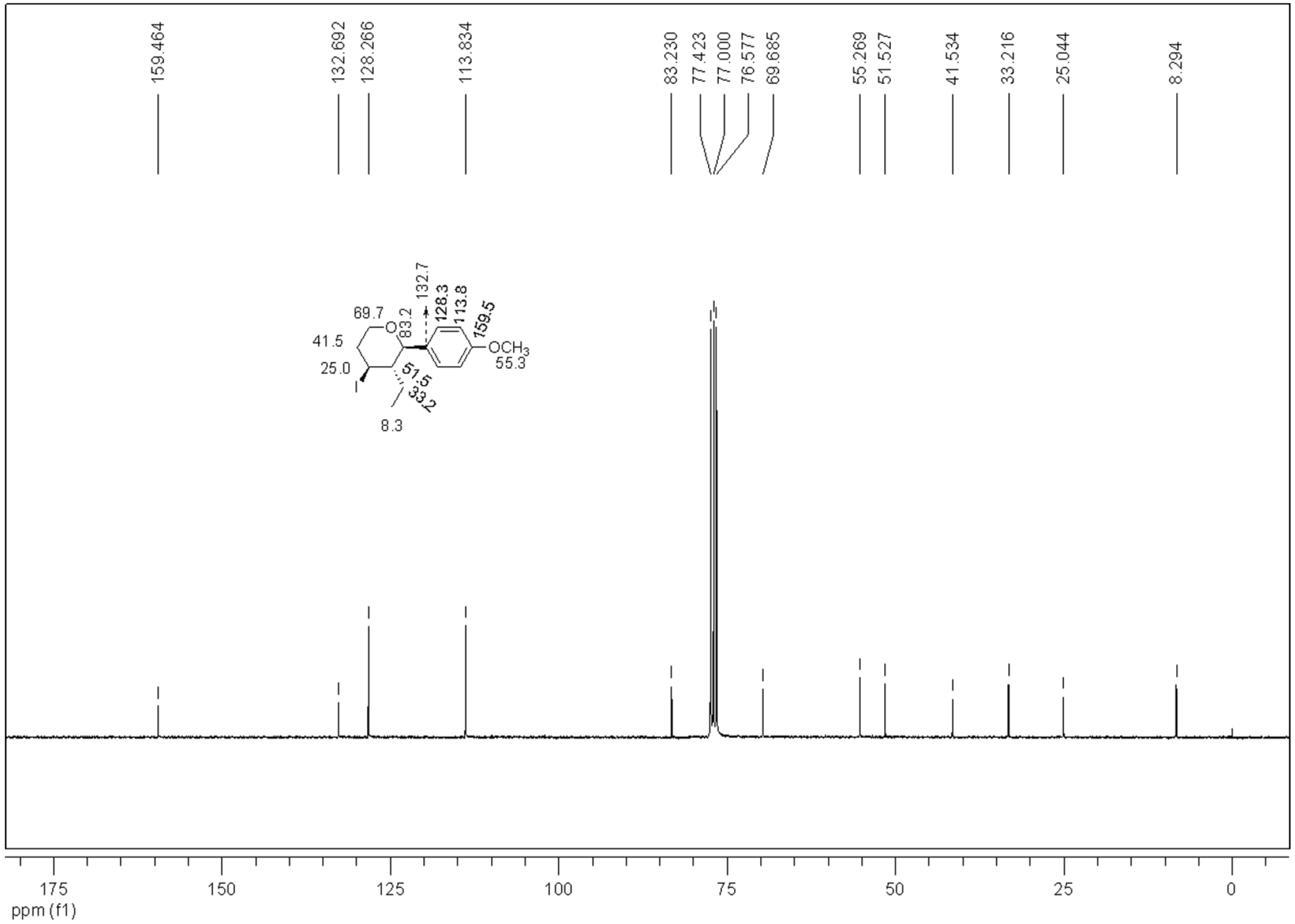

2D NOESY NMR of compound **5k** (300 MHz, $CDCl_3$)

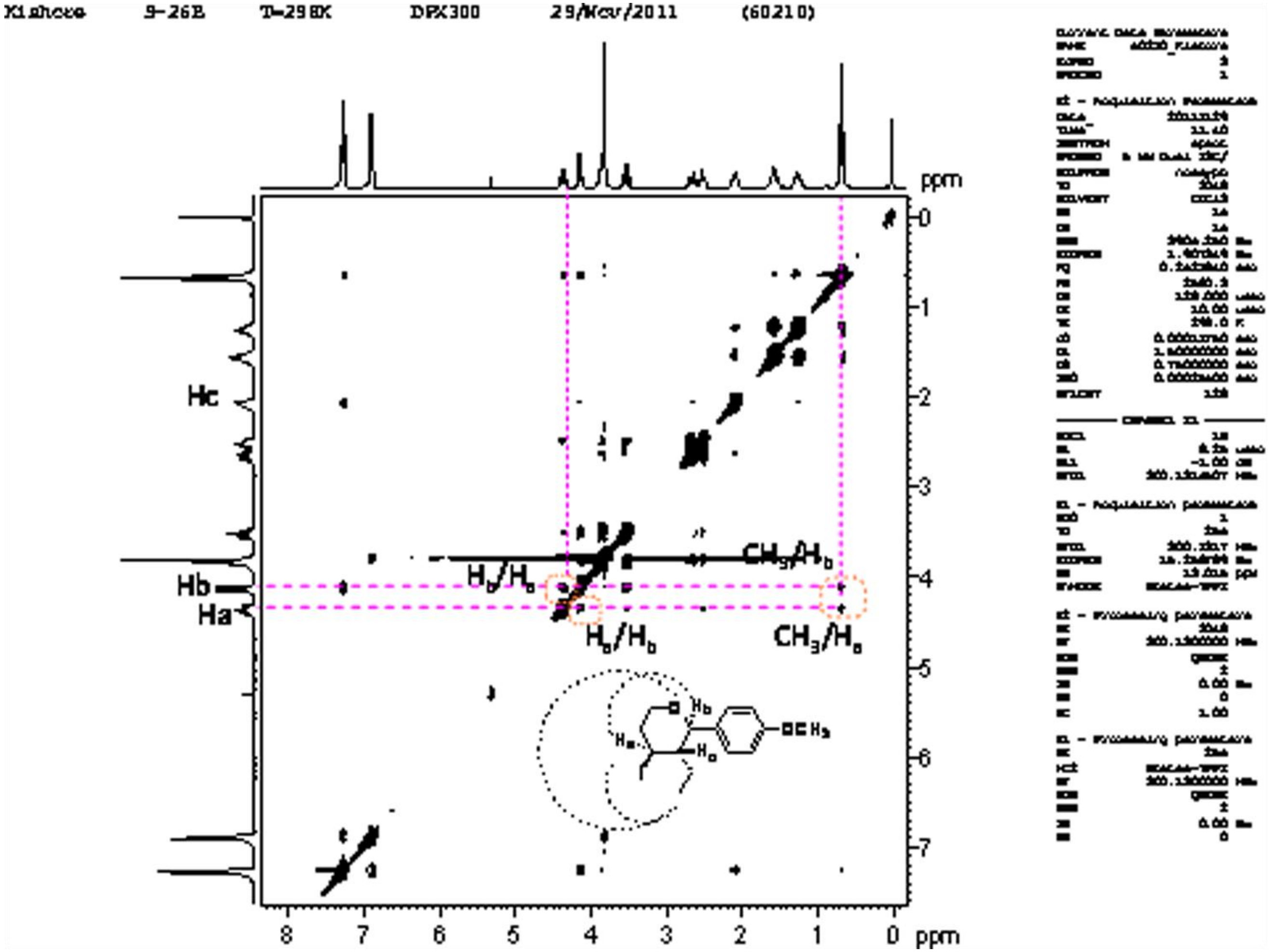

^{1}H-NMR of compound **5l** (200 MHz, $CDCl_3$)

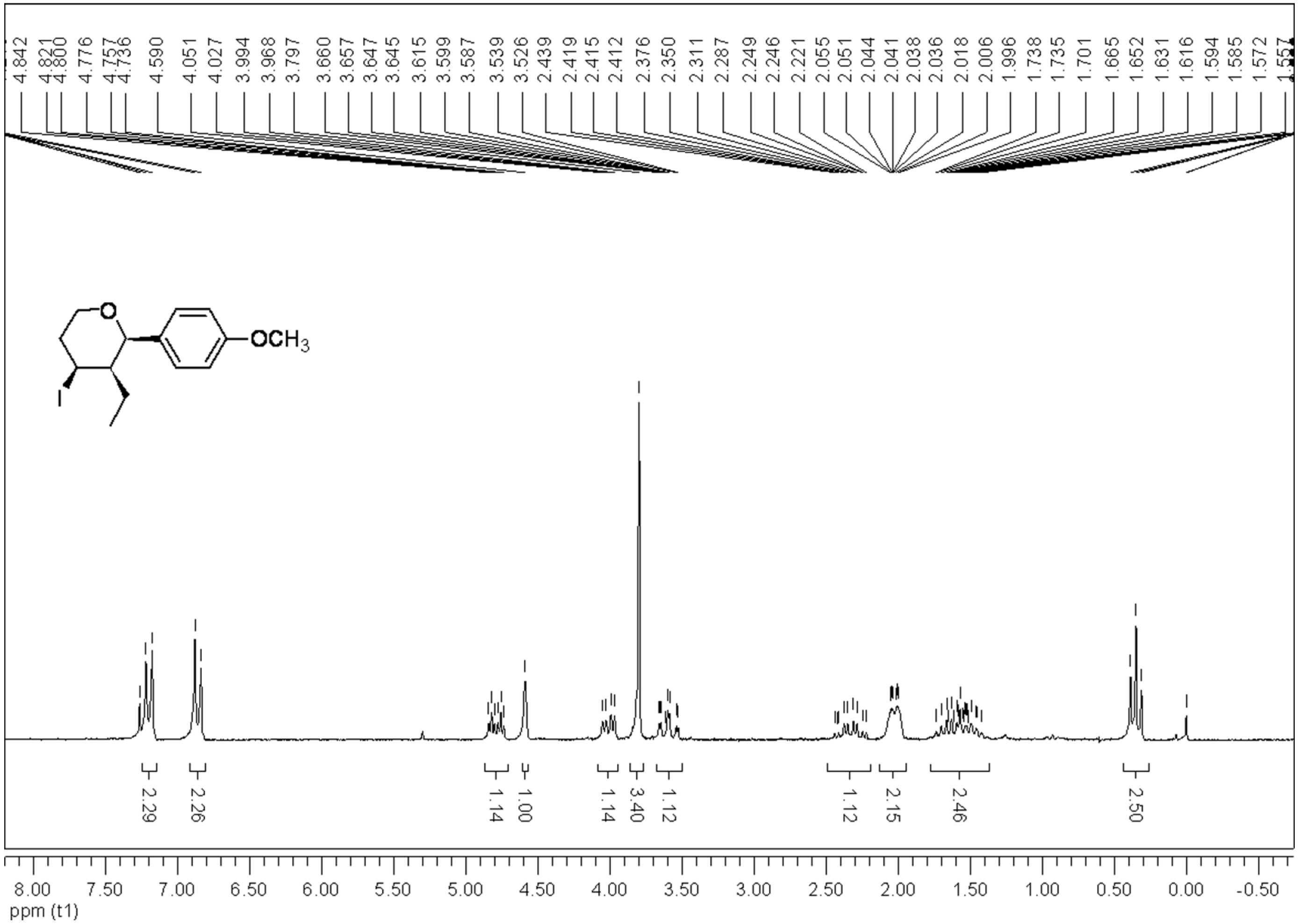

^{1}H-NMR of compound **5l** (200 MHz, $CDCl_3$)

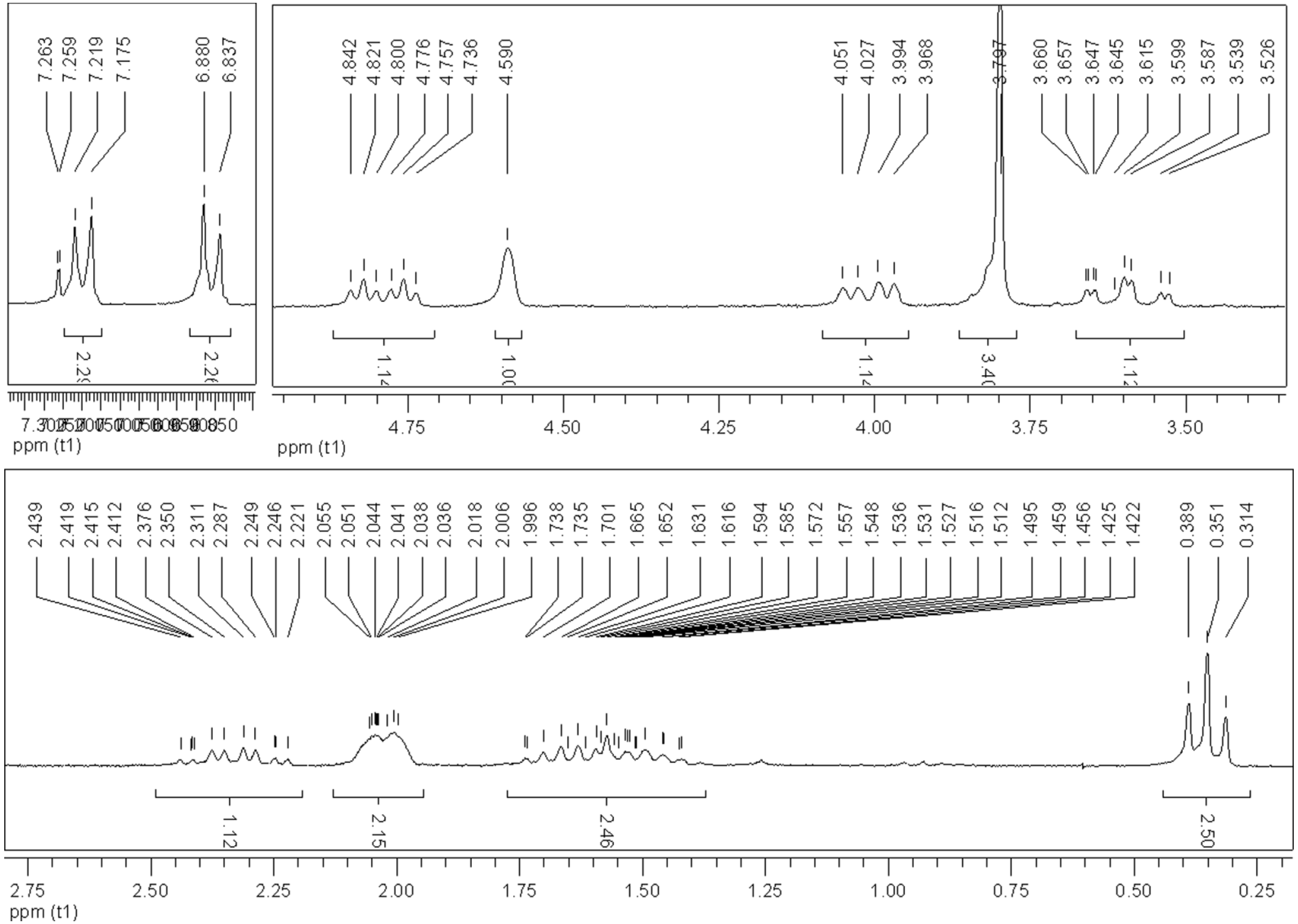

^{13}C-NMR of compound **5l** (50 MHz, $CDCl_3$)

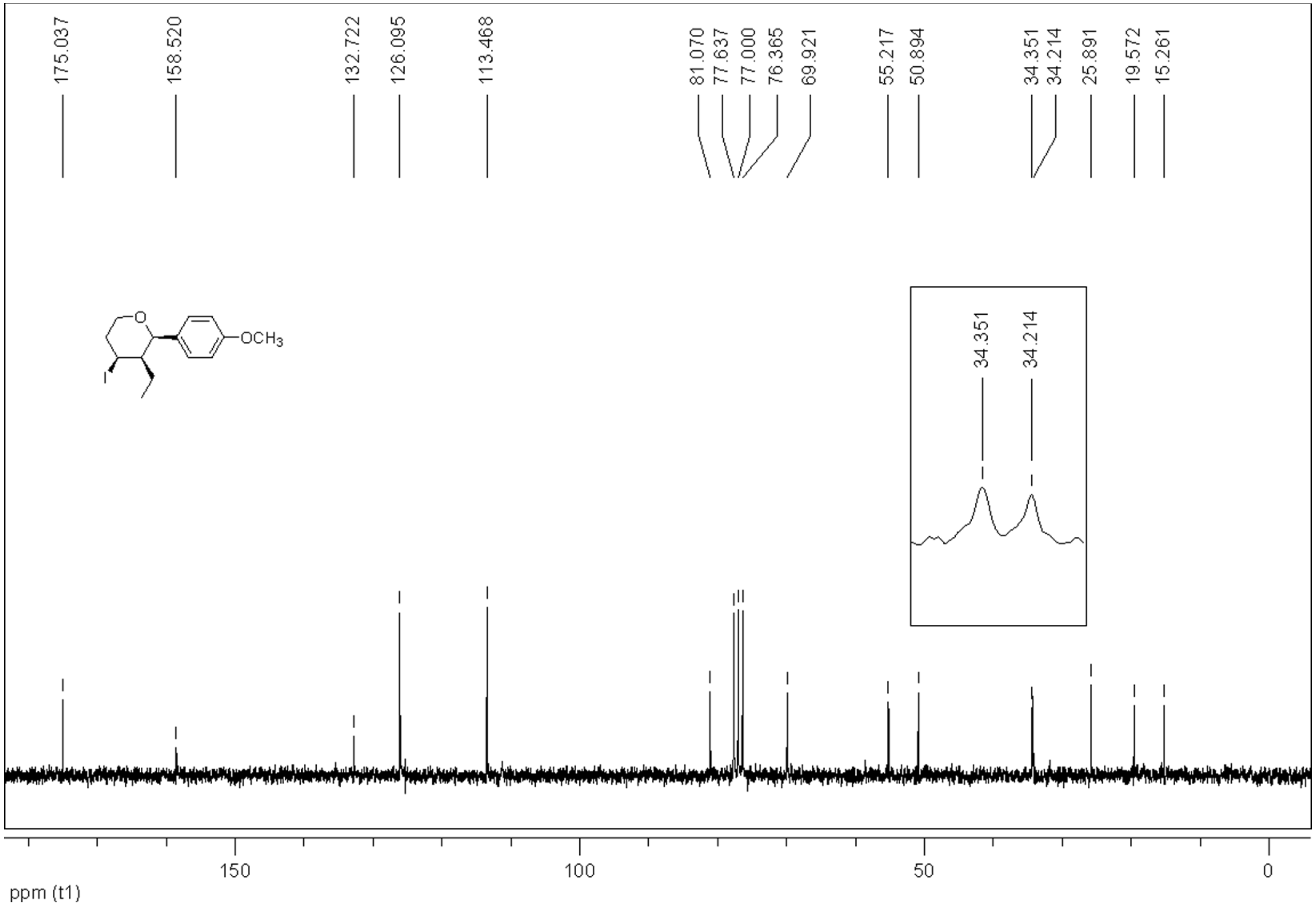

Supplement: Supplementary file 1 [file molecules-18-11100-s001.zip › molecules-39050-supplementary/molecules-39050-SM-final.pdf]
